# Pragmatic Solutions for Stroke Recovery and Improved Quality of Life in Low- and Middle-Income Countries—A Systematic Review

**DOI:** 10.3389/fneur.2020.00337

**Published:** 2020-06-25

**Authors:** Echezona Nelson Dominic Ekechukwu, Paul Olowoyo, Kingsley Obumneme Nwankwo, Olubukola A Olaleye, Veronica Ebere Ogbodo, Talhatu Kolapo Hamzat, Mayowa Ojo Owolabi

**Affiliations:** ^1^Department of Medical Rehabilitation, Faculty of Health Sciences and Technology, College of Medicine, University of Nigeria, Enugu, Nigeria; ^2^LANCET Physiotherapy and Wellness and Research Centre, Enugu, Nigeria; ^3^Department of Medicine, Federal Teaching Hospital, Ido Ekiti, Nigeria; ^4^College of Medicine and Health Sciences, Afe Babalola University, Ado Ekiti, Nigeria; ^5^Stroke Control Innovations Initiative of Nigeria, Abuja, Nigeria; ^6^Fitness Global Consult Physiotherapy Clinic, Abuja, Nigeria; ^7^Department of Physiotherapy, Faculty of Clinical Sciences, College of Medicine, University of Ibadan, Ibadan, Nigeria; ^8^Department of Physiotherapy, University College Hospital, Ibadan, Nigeria; ^9^Department of Medicine, Faculty of Clinical Sciences, College of Medicine, University of Ibadan, Ibadan, Nigeria; ^10^University College Hospital, Ibadan, Nigeria; ^11^Blossom Specialist Medical Centre, Ibadan, Nigeria

**Keywords:** pragmatic solution, stroke recovery, quality of life, low- and middle-income countries, innovatively high technology interventions, systematic review

## Abstract

**Background:** Given the limited healthcare resources in low and middle income countries (LMICs), effective rehabilitation strategies that can be realistically adopted in such settings are required.

**Objective:** A systematic review of literature was conducted to identify pragmatic solutions and outcomes capable of enhancing stroke recovery and quality of life of stroke survivors for low- and middle- income countries.

**Methods:** PubMed, HINARI, and Directory of Open Access Journals databases were searched for published Randomized Controlled Trials (RCTs) till November 2018. Only completed trials published in English with non-pharmacological interventions on adult stroke survivors were included in the review while published protocols, pilot studies and feasibility analysis of trials were excluded. Obtained data were synthesized thematically and descriptively analyzed.

**Results:** One thousand nine hundred and ninety six studies were identified while 347 (65.22% high quality) RCTs were found to be eligible for the review. The most commonly assessed variables (and outcome measure utility) were activities of daily living [75.79% of the studies, with Barthel Index (37.02%)], motor function [66.57%; with Fugl Meyer scale (71.88%)], and gait [31.12%; with 6 min walk test (38.67%)]. Majority of the innovatively high technology interventions such as robot therapy (95.24%), virtual reality (94.44%), transcranial direct current stimulation (78.95%), transcranial magnetic stimulation (88.0%) and functional electrical stimulation (85.00%) were conducted in high income countries. Several traditional and low-cost interventions such as constraint-induced movement therapy (CIMT), resistant and aerobic exercises (R&AE), task oriented therapy (TOT), body weight supported treadmill training (BWSTT) were reported to significantly contribute to the recovery of motor function, activity, participation, and improvement of quality of life after stroke.

**Conclusion:** Several pragmatic, in terms of affordability, accessibility and utility, stroke rehabilitation solutions, and outcome measures that can be used in resource-limited settings were found to be effective in facilitating and enhancing post-stroke recovery and quality of life.

## Introduction

Stroke is a major public health challenge in many Low- and Middle- Income Countries (LMICs) (1, 2). It is a leading cause of disability and premature mortality (3). Stroke is a common cause of severe financial hardship and poverty (4) and resources for stroke care and rehabilitation are sparse in LMICs (5). Rehabilitation services are typically limited and not easily affordable (6, 7). Although, there are several proven therapies and rehabilitation strategies for stroke in high income countries, these are not directly transferrable to LMICs (8). Many LMICs have minimal health care spending and any model of stroke rehabilitation for this region must not only be effective but practical and sustainable in terms of affordability, availability, accessibility and acceptability (7, 8). The global burden associated with stroke underscores the need for strategies to circumvent current trends and check the projected increase in stroke incidence in LMICs (1).

We conducted a systematic review of RCTs of interventions that addressed recovery of functioning, and enhancement of quality of life after stroke and discussed effective, cost-saving and practical rehabilitation models to improve clinical outcomes and quality of life among stroke survivors in LMICs.

The two main objectives of the review are therefore:

1. To determine effective interventions/modes of care delivery that enhances post-stroke recovery and quality of life and the outcome measures utilized.
2. To identify effective stroke rehabilitation interventions that would constitute pragmatic (cost-effective, accessible, and utilizable) solutions in lower and middle income countries.

## Methods

This systematic review of literature was based on the Preferred Reporting Items for Systematic Reviews and Meta-Analyses (PRISMA) guideline. Ethical standards necessary for the conduct of a systematic review were maintained. The study was registered with PROSPERO (CRD42020138454).

### Search Strategy

We conducted a search of PubMed, HINARI, and Directory of Open Access Journals (DOAJ) databases for articles published up to November 2018 using the Patient-Intervention-Comparison-Outcome (PICO) format with stroke (Patient Problem), non-pharmacologic stroke rehabilitation/neurorehabilitation strategies (Intervention), stroke recovery (Outcome) and quality of life (Outcome) as some of the keywords. We however did not specify comparison groups in the search strategy.

### Eligibility Criteria

Only studies that were identified as completed randomized controlled trials (RCTs), that involved adult stroke survivors (age ≥ 18 years) who underwent non-pharmacological rehabilitation in both the intervention and comparison groups, and with available full text were included in this review. However, published protocols, pilot and feasibility studies, and non-English language articles were excluded.

### Data Extraction

The titles and abstracts of articles were screened by the authors and studies that did not meet the eligibility criteria were excluded. Full texts of eligible studies were further scrutinized and the following information were obtained and recorded in prepared data extraction form: citation, number of study participants, purpose of the study (specific construct targeted), type of intervention, type of control, and outcome of intervention (between intervention and control groups difference) (see Supplementary Table).

### Quality Appraisal

The quality of the articles was assessed using JADAD scale (9). The scale also known as the Oxford quality scoring system has 7 items with a maximum score of 5 and a minimum score of 0. For the purpose of this review, studies with JADAD scores <3 were rated as low quality while those with scores ≥3 were rated as high quality studies.

### Data Synthesis

Thematic presentation of findings of the reviewed studies was done in line with the objectives of the review. Stroke recovery and their outcomes were operationalized using the broad categories of functioning based on the International Classification of Functioning, Disability and Health (ICF) conceptual framework (10). Thus, stroke rehabilitation interventions and outcomes assessed in the various studies were presented according to their effects on the recovery of body functions, activity and participation. The efficacy of trial interventions on quality of life was also presented as a separate theme. Stroke care models identified as effective in the reviewed articles were also presented as a specific theme. Summaries of the quality of studies that addressed each of the themes were presented.

## Results

**Figure 1:**
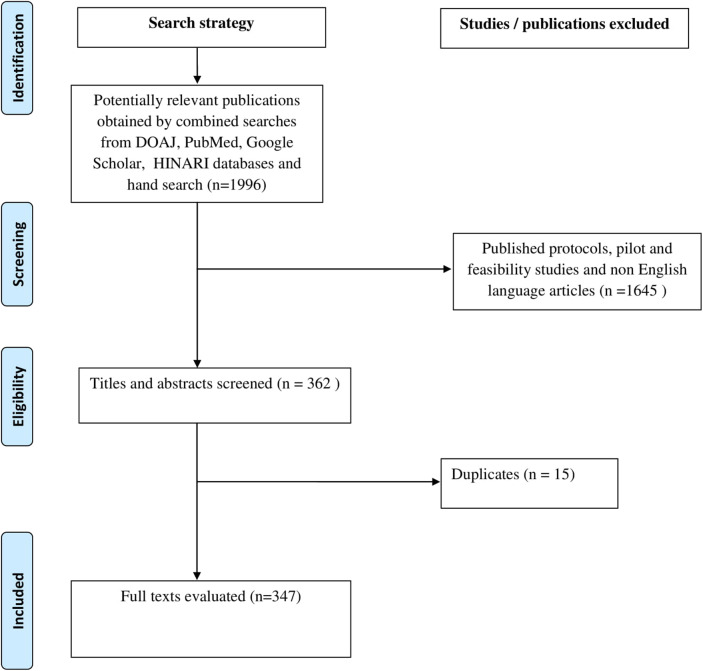
Preferred Reporting Items for Systematic Reviews and Meta-Analysis (PRISMA) flow diagram.

A total of 1996 studies were obtained from the electronic searches of the databases, while the findings of 347 studies with available full text articles were synthesized and presented. One thousand, six hundred and thirty-five articles were excluded because they did not meet with the inclusion criteria while 15 articles that contained duplicate data were also excluded. Details are presented in the PRISMA flowchart (Figure 1).

### Methodological Qualities of the Included Studies

In general, most of the studies (65.22%) included in this review were high quality trials (JEDAD Scores ≥3). Majority of the studies (>70.00%) with Transcranial Direct Current Stimulation (t-CDS), Virtual Reality (VR), Body Weight Supported Treadmill Training (BWSTT), mental practice, Task Oriented Therapy (TOT), muscle stretching exercises, speech therapy, participation based therapies, Community Based Rehabilitation (CBR), Home Based Rehabilitation (HBR), family/care-giver led therapy, and telerehabilitation were high quality trials. However, studies whose interventions hinged on robotics, Constraint Induced Movement Therapy (CIMT), Occupational Therapy (OT), Early Therapy, Cognitive Therapy, Quality of Life Centered Care were found to have an almost equal distributions in methodological quality as shown in Table 1.

**Table 1:** Summary of the methodological qualities of the included studies based on therapeutic techniques (*n* = 347).

**SN**	**Therapy**	**Low Quality**		**References**	**High Quality**		**References**	**Total**
		**f**	**%**		**f**	**%**		
1	Robotics	17	42.50	(11–27)	23	57.50	(28–47)	40
2	t-DCS	5	26.32	(48–52)	14	73.68	(30, 41, 53–61)	19
3	TMS	10	33.33	(62–71)	20	66.67	(65, 72–90)	30
4	FES	6	33.33	(91–96)	12	66.67	(97–108)	18
5	VR	7	26.92	(109–115)	19	73.08	(116–125)	26
6	Video Game	2	66.67	(126, 127)	1	33.33	(128)	3
7	BWSTT	3	27.27	(129–131)	8	72.73	(132–139)	11
8	OT	8	50.00	(17, 110, 140–143)	8	50.00	(54, 81, 144–146)	16
9	CIMT	18	47.37	(141, 147–162)	20	52.63	(36, 163–180)	38
10	Mirror Therapy	5	33.33	(19, 62, 181–183)	10	66.67	(184–193)	15
11	Mental Practice	2	28.57	(194, 195)	5	71.43	(145, 196–198)	7
12	TOT	6	25.00	(199–204)	18	75.00	(33, 37, 83, 205–216)	24
13	Muscle Strength Tr	5	35.71	(217–221)	9	64.29	(74, 222–228)	14
14	Muscle Stretching	0	0.00		3	100.00	(229–231)	3
15	Cognitive Therapy	3	42.86	(216, 232, 233)	4	57.14	(234–237)	7
16	Speech Therapy	0	0.00		4	100.00	(84, 238–240)	4
17	Aerobic Exercise/Physical Activity	18	40.91	(48, 109, 232, 241–255)	26	59.09	(205, 255–282)	44
18	Particip-Based Rx	1	20.00	(251)	4	80.00	(283–286)	5
19	QoL Centered Care	8	42.11	(287–294)	11	57.89	(225, 236, 295–303)	19
20	CBR	1	20.00	(304)	4	80.00	(305–308)	5
21	HBR	3	13.64	(309–311)	19	86.36	(190, 248, 303, 311–326)	22
22	Family/CG led Rx	1	16.67	(327)	5	83.33	(328–332)	6
23	Self-Management	1	50.00	(333)	1	50.00	(334)	2
24	Telerehabilitation	1	25.00	(335)	3	75.00	(312, 336, 337)	4
25	Early Therapy	5	55.56	(156, 338, 339)	4	44.44	(340–343)	9
	**Total**	**136**	34.78		**255**	65.22		

*t-DCS, Transcranial Direct Current Stimulation; TMS, Transcranial Magnetic Stimulation; FES, Functional Electrical Stimulation; VR, Virtual Reality; BWSTT, Body Weight-Supported Treadmill Training; OT, Occupational Therapy; CIMT, Constraint-Induced Movement Therapy; TOT, Task-Oriented Therapy; Tr, Training; Particip, Participation; QoL, Quality of Life; CBR, Community-Based Rehabilitation; HBR, Home-Based Rehabilitation; CG, Caregiver; Rx, Therapy*.

### Locations of Studies With Innovatively High Technology Interventions

**Figure 2:**
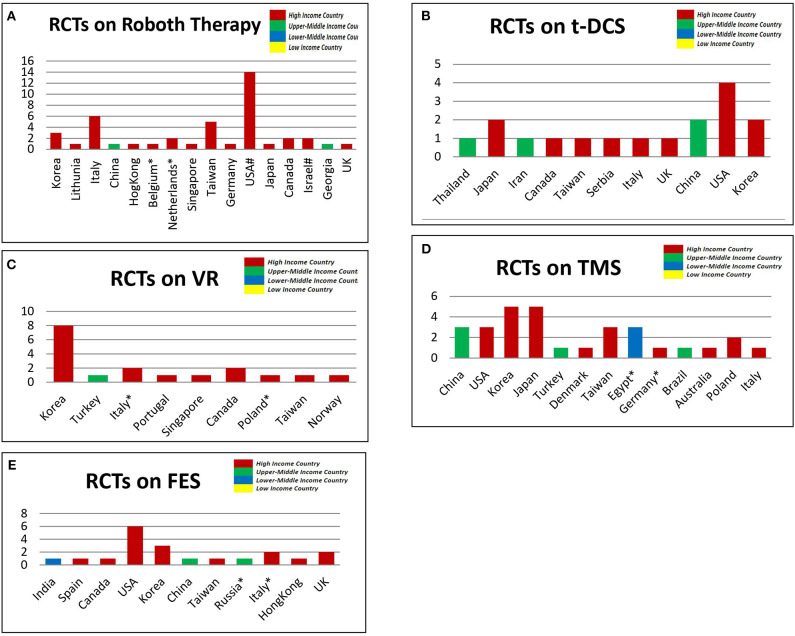
The Location of selected studies that used innovatively high technological interventions based on the June 2019 World Bank List of Economiesl **(A)**. Robot Therapy **(B)**. Virtual Reality [VR] **(C)**. Transcranial Direct Current Stimulation [t-DCS] **(D)**. Transcranial Magnetic Stimulation [TMS] **(E)**. Functional Electrical Stimulation [FES]. NB, X-axis = number of studies; Y-axis = Country, *,# each indicate a single study in multiple countries.

A total of 40 studies (11–50) conducted in 15 countries made use of Robot Therapy (RT). Majority (95.24) of these RT studies were done in high income countries such as USA (33.33%), Italy (14.29%) Taiwan (11.90%) etc. Very few studies (4.76%) were conducted in upper middle income countries (China and Georgia) while none was found in the lower middle and lower income countries. Also, of the 19 studies (16, 29, 51–64, 344) that compared the effects of transcranial direct current stimulation, 78.95% were conducted in high income countries, few (21.05%) in upper-middle-income countries, and none was found from lower-middle and lower income countries. Similarly, most of the trials on the effectiveness of virtual reality (94.44%), transcranial magnetic stimulation (88.0%) and functional electrical stimulation (85.00%) were conducted in high income countries as shown in Figure 2.

### Outcome Measures Reported and Their Utility

Using the ICF classification model, 24 themes representing constructs in the function/structure (impairment) domain were found in the included studies. A total of 160 studies (66.57%) out of the 347 reviewed studies assessed motor function. Other outcomes such as balance (19.31%), muscle strength (16.43%), spasticity (12.39%), and depression (12.39%) were among the most assessed function/structure related outcomes. Majority (71.88%) of the studies that assessed motor function utilized Fugl Meyer Assessment scale. Other frequently used tools for assessing motor function were Wolf Motor Function Test (16.25%), Action Reach Arm Test (13.75%) and Box and Block Test (12.50%) as shown in Table 2.

**Table 2:** Function- and structure-related outcome measures and their utility scores (*n* = 347).

**Construct**	**Outcome measure**	***x* + (*y*)**	***f***	**%**	**Rel. %**	**References**
Motor function	FMA	115(+0)	115	33.14	71.88	(11, 13, 14, 16, 17, 19, 24–27, 30–44, 46, 47, 49, 50, 52, 54, 56–58, 60, 61, 63–66, 73, 76, 77, 81, 83, 85, 87, 88, 92, 93, 95, 97, 98, 100, 102, 104, 105, 109, 111, 112, 116, 121, 122, 129, 132, 135, 137, 140, 144, 145, 151, 154, 155, 157, 159, 162, 163, 165–167, 178–180, 184–191, 194, 197, 200, 202, 205, 206, 209, 215, 222, 246, 260, 265, 274, 295, 311, 318, 323, 327, 337, 344–347)
	WMFT	13(+13)	26	7.49	16.25	(31, 36, 60, 75, 79, 81, 83, 98, 111, 128, 141, 147, 150, 156, 158, 160, 161, 163, 166, 173–175, 215, 344, 346)
	BBT	4(+16)	20	5.76	12.50	(30, 34, 38, 40, 51, 88, 89, 104, 105, 114, 116, 120, 185, 186, 200, 202, 214, 215, 335, 345)
	ARAT	7(+15)	22	6.34	13.75	(24, 98, 109, 116, 121, 149, 153, 163, 172, 180, 187, 189–191, 195, 205, 207, 209, 220, 268)
	MAS	12(+2)	14	4.03	8.75	(22, 144, 176, 210, 211, 231, 250, 253, 277, 280, 281, 300, 307, 312)
	MI	3(+6)	9	2.59	5.63	(44, 68, 88, 108, 116, 194, 251, 265, 282)
	(m)RS	0(+7)	7	2.02	4.38	(76, 104, 109, 116, 121, 163, 210)
	MSS	0(+3)	3	0.86	1.88	(25–27)
	EMG	0(+2)	2	0.58	1.25	(83, 87)
	RMA	2(+0)	2	0.58	1.25	(171, 204)
	Others	4(+3)	7	0.29^a^	0.63^a^	SSS (133), FIM (193), AMAT (106), STREAM (269), [RPSS (112), MFT (203), CAHAI (262)]
	**Total**	**160 (+71)**	**Σx** **=** **160**	**46.11**	**100.00**	
Muscle strength	MRC	12(+0)	12	3.46	23.08	(11, 26, 27, 34, 38, 43, 70, 76, 79, 83, 140, 144)
	MI	4(+1)	5	1.44	9.62	(28, 32, 34, 106, 270)
	MPS	2(+0)	2	0.58	3.85	(24, 25)
	Peak torque	4(+0)	4	1.15	7.69	(48, 75, 108, 267)
	Dynamometer	14(+0)	14	4.03	26.92	(55, 63, 86, 96, 207, 214, 215, 218, 219, 227, 228, 278, 296, 317)
	EMG	3(+2)	5	1.44	9.62	(107, 200, 206, 218, 226)
	MMT	3(+0)	3	0.86	5.77	(121, 137, 206)
	Virgometer	2(+0)	2	0.58	3.85	(247, 250)
	1RM	1(+1)	2	0.58	3.85	(223, 228)
	Hand grip	3(+0)	3	0.86	5.77	(59, 69, 237)
	Others	4 (+1)	5	0.29^a^	1.92^a^	HSS (80), KTPB (70), Pinch gauge (71), PGBT (120), Myometer (220)
	**Total (Σf)**	**52(+5)**	**Σx** **=** **52**	**14.99**	**100.00**	
Balance	BBS	34(+0)	34	9.80	68.00	(11, 13, 22, 23, 92, 102, 103, 110, 119, 127, 129, 134, 135, 139, 144, 217, 222, 242, 247, 250, 265, 266, 270, 273, 276, 278, 279, 299, 306, 312, 314, 318, 323, 343)
	TUG	6(+6)	12	3.46	24.00	(5, 11, 36, 49, 103, 107, 110, 119, 124, 250, 265, 348)
	ABC	0(+2)	2	0.58	4.00	(129, 135)
	FRT	3(+1)	4	1.15	8.00	(119, 271, 281, 321)
	FTSTS	1(+1)	2	0.58	4.00	(48, 244)
	Fall calendar	2(+0)	2	0.58	4.00	(248, 316)
	LoS	1(+1)	2	0.58	4.00	(199, 273)
	Others	3(+6)	9	0.29^a^	2.00^a^	BBA (102), PSV (110), COP (113), BPM (125) SQ (126), FABS (199), PASS (267), BMS (273), PPA (315)
	**Total**	**50(+17)**	**Σx** **=** **50**	**14.41**	**100.00**	
Muscle tone (spasticity)	(m)AS	37(+0)	37	10.66	90.24	(12, 14, 19, 24, 25, 27–29, 31, 32, 34, 41, 56, 63, 65, 66, 68, 73, 91, 96, 99, 112, 138, 145, 189, 192, 202, 204, 219, 230, 247, 250, 267, 312, 345, 346, 349)
	CSS	2(+0)	2	0.58	4.88	(107, 259)
	Others	2(+2)	4	0.29^a^	2.44^a^	EMG (68), H/M ratio (94), Pendulum Test (94), Myotron-3D (186)
	**Total (Σf)**	**41(+2)**	**Σx** **=** **41**	**11.82**	**100.00**	
Depression	HAD-S	16(+0)	16	4.61	38.10	(12, 216, 233, 236, 248, 260, 269, 277, 281, 291, 303, 308, 316, 328, 330, 332)
	CES-D	4(+0)	4	1.15	9.52	(21, 196, 289, 297, 319)
	BDI	4(+0)	4	1.15	9.52	(24, 89, 121, 300)
	GDS	7(+0)	7	2.02	16.67	(153, 214, 253, 283, 290, 299, 312)
	GHQ	2(+0)	2	0.58	4.76	(142, 304)
	Others	9(+1)	10	0.29^a^	2.38^a^	SADQ-H (80), IMTEQ (111), PHQ (222), ARS-D (244), STAI (299), Kessler-10 (289), MADS (292), DASS (305), Zungseas (340), SAS (216)
	**Total (Σf)**	**42(+1)**	**Σx** **=** **42**	**12.10**	**100.00**	
Pain	VAS	8(+0)	8	2.31	66.67	(28, 99, 183, 190, 231, 287, 295, 345)
	FMA	2(+0)	2	0.58	16.67	(24, 189)
	Others	2 (+1)	3	0.29^a^	8.33^a^	PNS (346), WBF (153), RAI (231)
	**Total (Σf)**	**12(+1)**	**Σx** **=** **12**	**3.46**	**100.00**	
Speech	WAB	2(+0)	2	0.58	12.50	(59, 72)
	ASRS	2(+0)	2	0.58	12.50	(80, 84)
	BDAE	2(+1)	3	0.86	18.75	(82, 84, 240)
	AAT	3(+0)	3	0.86	18.75	(67, 170, 238)
	PAS	2(+0)	2	0.58	12.50	(195, 224)
	Others	5 (+10)	14	0.29^a^	6.25^a^	TOM (239), COAST (37), CCAS (78), COM-B (350), DRS (68), [VfDS (217), HSS (80), PICA (240), BNT (82), SVPN (82), CAL (170), Milan protocol (238), FCP (240), Token Test (238), CPNT (84)]
	**Total (Σf)**	**16(+10)**	**Σx** **=** **16**	**4.61**	**100.00**	
Cognitive/Executive Fxn	ACER	2(+0)	2	0.58	7.69	(12, 118)
	TMT	4(+1)	5	1.44	19.23	(118, 234–236, 342)
	MMSES	5(+0)	5	1.44	19.23	(216, 232, 248, 293, 317)
	MCA	2(+1)	3	0.86	11.54	(216, 298, 306)
	Others	10 (11)	21	0.29^a^	3.85^a^	Token Test (138), THT (89), CL (197), SART (237), SPMSQ (289), PGCM (311), MAQ (196), CTT (198), VDS (234), CT-50CT (313), [VMIQ (197), S-CNPT (89), CFQ (235), AVLT (196), RBMT (197), Picture arrangement (118), CWST (234), BST (235), SPM (235), ESS (237), StSS (237)]
	**Total (Σf)**	**26(+13)**	**Σx** **=** **26**	**7.49**	**100.00**	
Range of motion (ROM)	Goniometer	8(+0)	8	2.31	72.73	(66, 91, 100, 105, 229–231, 345)
	Others	3(+0)	3	0.29^a^	9.09^a^	MCbA (282), 3D-MA (210); Reaching (40)
	**Total (Σf)**	**11(+0)**	**Σx** **=** **11**	**3.17**	**100.00**	
CVS, hemat and respiratory function	VO_2_ max	3(+0)	3	0.86	21.43	(44, 134, 278)
	HR	2(+1)	3	0.86	21.43	(44, 108, 275)
	MIP	2(+0)	2	0.58	14.29	(224, 249)
	PCI	2(+0)	2	0.58	14.29	(271, 286)
	Others	5(+14)	19	0.29^a^	7.14^a^	02 pulse (44), PC (255), IME (249), BP (44), MPV (255), SBMBDS (263), MEP (224), RPE (44), FVC (263), Vent Resp (44), CBF (232), FEVI (263), Borg's Scale (138), WBC (255), WHS (138), RBC (255), 2 MWT (348), Hg (255), FEV/FVC (263)
	**Total (Σf)**	**14(+15)**	**Σx** **=** **14**	**4.03**	**100.00**	
Structural dysfunction	X-ray	1(+0)	1	0.29	33.33	(99)
	fMRI	1(+0)	1	0.29	33.33	(151)
	LVM	1(+0)	1	0.29	33.33	(158)
	**Total**	**3(+0)**	**Σx** **=** **3**	**0.86**	**100.00**	
Cortical excitability	TMS	6(+0)	6	1.73	33.33	(58, 71, 90, 156, 187, 274)
	rMT	4(+2)	6	1.73	33.33	(68, 70, 80, 83, 90, 187)
	MEP	4(+4)	8	2.31	44.44	(62, 63, 70, 74, 83, 87, 90, 187)
	aMT	0(+2)	2	0.58	11.11	(70, 80)
	MMA	0(+2)	2	0.58	11.11	(68, 83)
	fMRI	4(+0)	4	1.15	22.22	(59, 210, 336, 349)
	Others	0(+2)	2	0.29^a^	5.56^a^	[SICI (67), ICF (67)]
	**Total (Σf)**	**18(+12)**	**Σx** **=** **18**	**5.19**	**100.00**	
Perception and sensation	2PD	3(+0)	3	0.86	23.08	(176, 184, 251)
	Others	10 (+1)	11	0.29^a^	7.69^a^	Ns (130) NSA (188) CBS (258) Oxford Scale (138) SCT (189) Light Trash (282) [Cutaneous Threshold (184)] NEIVEQ (243) Brush mood (183) RASP (186) AMT (245)
	**Total (Σf)**	**13(+1)**	**Σx** **=** **13**	**3.74**	**100.00**	
Posture	TCT	3(+0)	3	0.86	60.00	(106, 138, 251)
	Others	2(+1)	3	0.29^a^	20.00^a^	PASS (102), SBMS (94), [mRS (138)]
	**Total (Σf)**	**5(+1)**	**Σx** **=** **5**	**1.44**	**100.00**	
Hemineglect	BIT	1(+0)	1	0.29	50.00	(191)
	Albert Test	1(+0)	1	0.29	50.00	(138)
	**Total**	**2**(+0)	**Σx** **=** **2**	**0.58**	**100.00**	
Attitude and belief	ABC	2(+0)	2	0.58	22.22	(216, 222)
	Others	7(+0)	7	0.29^a^	11.11^a^	SEOEE (203), LSES (284), FES (336), GSES (234), CABS (351), SEQ (262), SSEQ (333)
	**Total (Σf)**	**9(+0)**	**Σx** **=** **9**	**2.59**	**100.00**	
Infection	FLUTS-Q	1(+0)	1	0.29	–	(226)
flexibility	EFT	1(+0)	1	0.29	–	(226)
fatigue/Stress	CSI	6(+0)	6	1.73	50.00	(303, 312, 317, 322, 330)
	CBS	2(+0)	2	0.58	16.67	(314, 328)
	Others	2(+2)	4	0.29^a^	8.33	CIS-F (269), [GHQ (352), SOL-f (269) RSS (350)]
	**Total (Σf)**	**12(+0)**	**Σx** **=** **12**	**3.46**	**100.00**	
Social support	PRO-85	1(+0)	1	0.29	–	(291)
Fxn	IIQ	1(+0)	1	0.29	–	(303)
COST	Fin. Acct.	1(+0)	1	0.29	50.00	(345)
	Econ. Eval	1(+0)	1	0.29	50.00	(312)
	**Total**	**2(+0)**	**Σx** **=** **2**	**0.58**	**100.00**	
Satisfaction	GAS	2(+0)	2	0.58	22.22	(185, 272)
	Others	7(+1)	8	0.29^a^	11.11^a^	VAS (269), SASC-19 (291), WHOQoL (284), Likert Scale (304), PSS (330), SSMBP (333), SSPS (336), [PoSS (330)]
	**Total (Σf)**	**9(+1)**	**Σx** **=** **9**	**2.59**	**100.00**	

n^a^, n% for each of the outcome measures; x, exclusive frequency; y, repeated frequency, f, sum of x and y; % = (f/347^*^100); Rel %, (f/Σx^*^100).

*FMA, Fugl Meyer Assessment Scale; WMFT, Wolf Motor Function Test; BBT, Box and Block Test; ARAT, Action Reach Arm Test; MAS, Motor Assessment Scale; MI, Motricity Index; (m)RS, (modified) Rankin Scale; MSS, Motor Status Scale; RMA, Rivermead Motor Assessment AMAT, Action Reach Arm Test; RPSS, Reaching Performance Scale for Stroke; SSS, Scandinavian Stroke Scale; FIM, Functional Independence Measure; MFT, Motor Function Test; CAHAI, Chedoke Arm and Hand Activity Inventory; STREAM, Stroke Rehabilitation Assessment for Movement; MRC, Medical Research Council Scale for Muscle Strength; MPS, Motor Power Scale; EMG, Electromyogram; MMT, Manual Muscle Test; 1RM, One Repetition Maximum; HSS, Hemiplegic Stroke Scale; KT PB, Keyboard Tapping and Peg Board Task; ROM, Range of Motion; BBS, Bergs Balance Scale; TUG, Time Up and Go test; ABC, Activity specific Balance Confidence scale; FRT, Functional Reach Test; FTSTS, Five Times Sit to Stand Test; LoS, Level of Support; BBA, Brunel Balance Scale; PSV, Postural Say Velocity; CoP, Center of Pressure; BPM, Balance Performance Monitor; SQ, Semistructured Questionnaire; FABS, Fullerton Advanced Balance Scale; PASS, Postural Assessment Scale for Stroke; BMS, Balance Master System; PPA, Physiological profile Assessment; (m)AS, (modified) Ashworth Scale; CSS, Composite Spasticity Scale; H-M ratio, Hoffman Reflect–Motor Response ratio; HAD-S, Hospital Anxiety and Depression Scale; CES-D, Center for Epidemiologic Studies Depression Scale; BDI, Beck's Depression Inventory; GDS, Geriatric Depression Scale; GHQ, General Health Questionnaire; SADQ-H, Stroke Aphasic Depression Questionnaire—Hospital Version; IMTEQ, Intrinsic Motivational Task Evaluation Questionnaire; PHQ, Patient Health Questionnaire; ARS-D, Aphasia Rating Scale for Depression; STAI, State Trait Anxiety Inventory; MADS, Montgomery Asberg Depression Scale; DASS, Depression Anxiety Stress Scale; SAS, Self-rating Anxiety Scale; VAS, Visual Analog Scale; PNS, Pain Numerical Scale; WBF, Wong-Baker Faces Pain Scale; RAI, Resident Assessment Instrument; WAB, Western Aphasia Battery; ASRS, Apraxia of Speech Rating Scale; BDAE, Boston Diagnostic Aphasia Examination; AAT, Aachen Aphasia Test; PAS, Penetration Aspiration Scale; TOM, Therapy Outcome Measure; COAST, Communication Outcomes After Stroke Scale; CCAS, Concise Chinese Aphasia Scale; COM-B, Capability, Opportunity, Motivation—Behavior model; VfDS, Videofluoroscopic Dysphagia Scale; HSS, Hemiplegic Stroke Scale; PICA, Porch Index of Communicative Ability; BNT, Boston Naming Test; SVPN, Solutions with Virtual Private Networks; CAL, Communicative Activity Log; FCP, Functional Communication Profile; CPNT, Computerized Picture Naming Test; DRS, Dysphagia Rating Scale; ACE, Addenbrooke's Cognitive Examination; TMT, Trail Making Test; MMSES, Mini-Mental Stroke Examination Scale; ROM, Range of Motion; MCA, Montreal Cognitive Assessment scale; THT, Tower of Hanoi Task; CL, Cognitive Log; VMIQ, Vividness of Movement Imagery Questionnaire; SART, Sustained Attention to Response Test; S-CNT, Seoul Computerized Neuropsychiatric Test; CFQ, Cognitive Failure Questionnaire; SPMSQ, Short Portable Mental Status Questionnaire; PGCM, Philadelphia Geriatric Center Morale Scale; MAQ, Meta-memory in Adulthood Questionnaire; AVLT, Auditory Verbal Learning Test; CTT, Color Test Trial; RBMT, Rivermead Behavioral Memory Test; VDS, Verbal Digital Test; CWST, Color–Word Stroop Test; BST, Block Span Test; DST, Digit Span Test; SPM, Standard Progressive Matrices; ESS, European Sleepiness Scale; StSS, Strafford Sleepiness Scale; CT-50 CT, CT-50 Cognitive Test; MCbA, Motor Club Assessment; 3D-MA, 3D Motion Analysis; CVS, Cardiovascular System; VO_2_Max, Maximal Oxygen Consumption; HR, Heart Rate; MIP, Maximum Inspiratory pressure; PCI, Physiological Cost Index; PC, Platelet Count; IME, Inspiratory Muscular Endurance; BP, Blood Pressure; MPV, Mean Platelet Volume; SBMBDS, Shortness of Breath Modified Borg Dyspnea Scale; MEP, Maximum Expiratory Pressure; RPE, Rate Perceived Exertion; FVC, Forced Vital Capacity; Vent-Resp, Ventilatory Response; CBF, Cerebral Blood Flow; FEV1, Forced Expiratory Volume in 1 s; WBC, White Blood Count; RBC, Red Blood Count; 2 MWT, 2 minute Walk Test; fMRI, functional Magnetic Resonance Imaging; LVM, Longitudinal Voxel Morphology; TMS, Transcranial Magnetic Imaging; rMT, rest Motor Threshold; MEP, Motor Evoked Potential; aMT, active Motor Threshold; MMA, Motor Map Area; SICI, Short-Interval Intracortical Inhibition; ICF, Intra-Cortical Facilitation; 2PD, Two point Discrimination; NS, Numerical Scale; RASP, Rivermead Assessment of Somatosensory Performance; NSA, Nottingham Sensory Assessment; CBS, Catherine Bergego Scale; SCT, Star Cancellation Test; NEI-VFQ, National Eye Institute Visual functioning Questionnaire; TCT, Trunk Control Test; PASS, Posture Assessment Scale for Stroke; SBMS, Smart Balance Master System; BIT, Behavioral Inattention Test; SEOEE, Short Self-efficacy and Outcomes Expectations for Exercise; LSES, Liverpool Self-Efficacy Scale; FES, Falls Efficacy Scale; GSES, General Self-Efficacy Scale; CABS, Cerebrovascular Attitudes and Beliefs Scale; SEQ, Self-Efficacy Questionnaire; SSEQ, Stroke Self-Efficacy Questionnaire; FLUTS-Q, Female Lower Urinary Tract Symptom Questionnaire; EFT, Eriksen Flanker Test; CSI, Carer Strain Index; CBS, Caregiver Burden Scale; CIS-f, Checklist Individual Strength—subscale fatigue; SOL-f, Self-Observation List—fatigue subscale; RSS, Relatives' Stress Scale; PRO-85, Personal Resource Questionnaire; IIQ, Incontinence Impact Questionnaire; Fin Acct, Financial Account, Econ. Eval, Economic Evaluation; GAS, Goal Attainment Scale; SASC, Satisfaction-With-Stroke-Care questionnaire; WHOQoL, WHO Quality of Life Scale; PSS, Patient Satisfaction with Services; SSMBP, Stroke Self-Management Behaviors Performance Scale; PoSS, Pound Satisfaction Scale*.

Table 3 summarized the utility scores of outcome measures (Activities of Daily Living [ADL], Gait, and Mobility) in the Activity domain of the ICF classification system. A total of 208 studies (75.79%) out of the 347 studies in this review assessed ADL. Majority of these studies used Barthel Index or its modification (37.02%), Motor Activity Log (20.19%) and Functional Independence Measure (17.31%). In the same vein, 75 (31.2%) and 46 (14.70%) of the included studies assessed gait and mobility outcomes, respectively. Six minutes walk test (46.67%) and 10 meters walk test (38.67%) were the most utilized tool for assessing gait outcomes, while Functional Ambulatory Capacity (26.09%) and Rivermead Mobility Index (26.09%) were the most utilized outcomes for assessing post stroke mobility.

**Table 3:** Activity-related outcome measures and their utility scores (*n* = 347).

**Construct**	**Outcome measure**	**x + (y)**	**f**	**%**	**Rel. %**	**References**
ADL	FAS	1(+2)	3	0.86	1.44	(44, 161, 195)
	FIM	30(+6)	36	10.37	17.31	(19, 26, 42, 47, 49, 65, 66, 93, 112, 122, 132, 136, 137, 149, 153–155, 157, 164, 177, 179, 191, 192, 230, 237, 242, 244, 266, 277, 283, 285, 305, 317, 320, 348)
	ABILhand	3(+3)	6	1.73	2.88	(15, 47, 114, 176, 186, 190)
	(m)BI	75(+2)	77	22.19	37.02	(11, 13, 28, 29, 34, 38, 42, 44, 53, 56, 61, 69, 70, 73, 76, 77, 88, 89, 92, 95, 100, 102, 109, 121, 138, 140, 144, 145, 152, 167, 185, 189, 197, 214, 231–233, 247, 248, 251, 253, 254, 260, 266, 267, 270, 272, 276, 282, 291, 293–295, 297, 298, 302–304, 306–308, 310, 312–314, 316, 320, 322, 328–332, 334, 340, 353)
	MAL	39(+3)	42	12.10	20.19	(15, 17, 30, 31, 33, 36, 37, 41, 43, 47, 59, 100, 110, 149, 154, 155, 157–162, 165, 167, 171–173, 176–180, 183, 186, 188, 195, 311, 345, 347)
	ARAT	8(+1)	9	2.59	4.33	(14, 33, 37–39, 50, 52, 53, 311)
	WMFT	5(+3)	8	2.31	3.85	(19, 40, 46, 52, 68, 87, 183, 251)
	JTHFT	7	7	2.02	3.37	(54, 58, 120, 145, 211, 280, 335)
	9HPT	6(+3)	9	2.59	4.33	(163, 166, 172, 214, 220, 268, 269, 299, 325)
	IADL Scale	2(+1)	3	0.86	1.44	(129, 135, 165)
	NEADL	2(+6)	8	2.31	3.85	(142, 146, 155, 157, 251, 277, 307, 328)
	MFT	3(+0)	3	0.86	1.44	(99, 104, 148)
	AMAT	2(+0)	2	0.58	0.96	(97, 98)
	FAI	3(+8)	11	3.17	5.29	(22, 23, 149, 197, 282, 292, 293, 308, 310, 329, 332)
	OAR	1(+2)	3	0.86	1.44	(247, 275, 276)
	CMSA	3(+1)	4	1.15	1.92	(45, 124, 134, 237)
	Purdue Pegbox	2(+0)	2	0.58	0.96	(55, 251)
	mRS	2(+2)	4	1.15	1.92	(238, 291, 312, 337)
	E-ADL	1(+1)	2	0.58	0.96	(304, 326)
	SIS	1(+1)	2	0.58	0.96	(103, 122)
	TEMPA	1(+1)	2	0.58	0.96	(214, 215)
	Others	11(+9)	20	0.29^a^	0.48^a^	e-keyboard (57), SVIPT (51), Pen Recrider (143), UMCIT (106), SST (281), SHFT (176), AFT (194), HAP (286), YPAS (203), TUG (317), SIADL (252), [BBT (39), CAHAL (45), PPT (237), SOE (194), RMA (353), LHS (303), NHP (293), VAS (293), SAS (348)]
	**Total (Σf)**	**208(+55)**	**Σx** **=** **208**	75.79	100.00	
Gait	5 MWT	2(+0)	2	0.58	2.67	(22, 281)
	10 mWT	29(+0)	29	8.36	38.67	(11, 29, 42, 48, 74, 92, 101, 103, 108, 119, 125, 129, 130, 136, 138, 139, 186, 241, 265, 270, 271, 277, 308, 314, 315, 323, 325, 348, 349)
	6 MWT	23(+12)	35	10.09	46.67	(22, 23, 29, 42, 96, 101, 103, 129, 130, 132, 134–138, 212, 214, 219, 221, 223, 228, 237, 241, 247, 250, 266, 269, 276, 278, 279, 286, 314, 315, 323, 349)
	FAC	3(+3)	6	1.73	8.00	(22, 44, 65, 88, 348, 349)
	GAITrite	3(+3)	6	1.73	8.00	(22, 87, 103, 123, 125, 213)
	RMI	0(+2)	2	0.58	2.67	(22, 349)
	(m)EFAP	1(+4)	5	1.44	6.67	(23, 91, 96, 101, 103)
	Camera	2(+1)	3	0.86	4.00	(175, 178, 186)
	FGS	1(+1)	2	0.58	2.67	(219, 221)
	Others	11(+7)	18	0.29^a^	1.33^a^	3 MWT (261), 50 MWT (106), Force plate (20), DMA (167), PSM (262), CGS (297), POMA (49), PMS (113), WGS (127), FSS (227), Digital Recording (181), [PAV (261), Symmetry (88), PCI (108), SAM (135), mMAS (125), RVGA (212), Paper walking print (212)]
	**Total (Σf)**	**75(+33)**	**Σx** **=** **75**	31.12	100.00	
Mobility	FAC	12(+0)	12	3.46	26.09	(115, 133, 136, 138, 139, 144, 193, 212, 261, 265, 267, 277)
	TUG	7(+0)	7	2.02	15.22	(221, 241, 242, 247, 271, 277, 280)
	(m)RMI	9(+3)	12	3.46	26.09	(227, 245, 251, 261, 265, 270, 272, 277, 298, 308, 310, 332)
	Accelerometer	6(+0)	6	1.73	13.04	(36, 40, 71, 181, 197, 262)
	STREAM	2(+0)	2	0.58	4.35	(214, 349)
	Others	10(+2)	12	0.29^a^	2.17^a^	RBCT (167), Independent walk (130), Video (203), Reaction time (182), HTM (201), MAC (258), Optotrack (215), 2 mWT (124), FQOM (324), mMAS (321), [UMT (168), PMV (182)]
	**Total (Σf)**	**46(+5)**	**Σx** **=** **46**	14.70	100.00	

n^a^, n% for each of the outcome measures; x, exclusive frequency; y, repeated frequency, f, sum of x and y; %=(f/347^*^100); Rel % =(f/Σx^*^100).

*ADL, Activities of Daily Living; FAS, Functional Assessment Scale; FIM, Functional Independence Measure; (m)BI, (modified) Barthel Index; MAL, Motor Activity Log; ARAT, Action Research Arm Test; WMFT, Wolf Motor Function Test; JTHFT, Jebsen Taylor Hand Function Test; 9HPT, Nine Hole Peg Test; IADL-Scale, Instrumental Activities of Daily Living Scale; NEADL, Nottingham Extended Activities of Daily Living Scale; MFT, Manual Function Test; AMAT, Arm Motor Ability Test; FAI, Frenchay Activities Index; OAR, Older Americans Resources and Services; CMSA, Chedoke Master Stroke Assessment; mRS, modified Rankin Scale; E-ADL, Extended Activities of Daily Scale; SIS, Stroke Impact Scale; SVIPT, Sequential Visual Isometric Pinch Task; UMCIT, Upright Motor Control Test; SST Sit-to-Stand Test; SHFT, Sollerman Hand Function Test; AFT, Arm Functional Test; HAP, Human Activity Profile; YPAS, Yale Physical Activity Survey; TUG, Time Up and Go test; SIADL, Sunnaas Index of Activity of Daily Living; BBT, Box and Block Test; CAHAL, Chedoke Arm & Hand Activity Inventory; PPT, Purdue Pegboard Test; SOE, Speed of Execution; RMA, Rivermead Motor Assessment scale; LHS, London Handicap Scale; NHP, Nottingham Health Profile; VAS, Visual Analog Scale; SAS, Stroke Activity Scale; 5 MWT, 5 minute Walk Test; 10 mWT, 10-Meter Walk Test; 6 MWT, 6 minute Walk Test; FAC, Functional Ambulatory Capacity; RMI, Rivermead Mobility Index; (m)EFAP, (modified)Emory Functional Ambulatory Profile; FGS, Fast Gait Speed; 3 MWT, 3 minute Walk Test; 50 mWT, 50-Meter Walk Test; DMA, Dartfish motion analysis software; PSM, Pressure Sensitive Mat; CGS, Comfortable Gait Speed; POMA, Performance-Oriented Mobility Assessment; PMS, Pressure Mat System; WGS, Wisconsin Gait Scale; FSS, Foot Steps Symmetry; PAV, Peak Angular Velocity; PCI, Physiological Cost Index; SAM, Step Activity Monitor; mMAS, modified Motor Assessment Scale; RVGA, Rivermead Visual Gait Assessment; STREAM, Stroke Rehabilitation Assessment of Movement; RBCT, Rhythmic Bimanual Coordination Tasks; HTM, Hand-To-Mouth task; MAC = Mobility Assessment Course; 2 mWT, 2-Meter Walk Test; FQoM, Functional Quality of Movement Scale; UMT, Unimanual Motor Task; PMV, Peak Movement Velocity*.

Quality of life (QoL), post stroke reintegration and stroke impact were the three generated themes representing outcomes in the participation domain of the ICF model. Out of the 59 studies (20.17% of the included studies) that assessed QoL, SF-36 (35.59%) and Stroke Impact Scale [SIS] (30.51%) were the most utilized outcome measures. Also, SIS (21.74%) was the most utilized outcome measure in assessing post-stroke reintegration. From the 32 studies that assessed stroke severity/recovery, NIH stroke scale (50.00%) was the most frequently used outcome measure. In the same vein, SIS (45.16%) was the most utilized tool for assessing stroke impact as shown in Table 4.

**Table 4:** Participation-related outcome measures and their utility scores (*n* = 347).

**Construct**	**Outcome measure**	**x + (y)**	**f**	**%**	**Rel. %**	**References**
QoL	SIS	18(+0)	18	5.19	30.51	(17, 18, 21, 31, 43, 129, 149, 153, 154, 159, 179, 187, 189, 299, 306, 320, 328, 346)
	EuroQol	10(+0)	10	2.88	16.95	(37, 121, 190, 196, 227, 300, 302, 304, 305, 313)
	SF-36	19(+2)	21	6.05	35.59	(23, 37, 77, 264, 277, 288–291, 294, 296, 297, 301, 303, 307, 310, 315, 317, 320, 332, 340)
	SSQoL	4(+0)	4	1.15	6.78	(66, 103, 235, 298)
	WHOQoL	0(+2)	2	0.58	3.39	(196, 296)
	NHP	4(+0)	4	1.15	6.78	(247, 248, 276, 292)
	SA-SIP	2(+0)	2	0.58	3.39	(319, 321)
	SSS	1(+2)	3	0.86	5.08	(109, 264, 294)
	Others	1(+5)	6	0.29^a^	1.69^a^	EQVAS (309), [HUI (18) RS (302), N-QoL (296), QoLI (300), GHQ (332)]
	**Total (Σf)**	**59(+11)**	**Σx** **=** **59**	20.17	100.00	
Reintegration	SIS	5(+0)	5	1.44	21.74	(42, 203, 219, 221, 314)
	AAP	2(+0)	2	0.58	8.70	(129, 315)
	COPM	3(+0)	3	0.86	13.04	(141, 145, 235)
	NLQ	2(+0)	2	0.58	8.70	(142, 146)
	RNLI	2(+0)	2	0.58	8.70	(289, 330)
	Others	7(+2)	9	0.29^a^	4.35^a^	Social support lest (196), 0.8ms-2 mobilization (220), TRIP (206), RTWQ (298), LIFE-H (300), PASIPD (278), LHS (332), [IPA (196), Pedometer (315)]
	**Total (Σf)**	**21(+2)**	**Σx** **=** **21**	6.63	100.00	
Stroke severity/Recovery	NIHSS	16(+0)	16	4.61	50.00	(22, 24, 28, 68, 69, 76, 80, 85, 86, 95, 148, 153, 187, 222, 311, 347)
	CNS	2(+0)	2	0.58	6.25	(29, 237)
	(m)RS	2(+2)	4	1.15	12.50	(187, 222, 313, 322)
	RLOC	2(+0)	2	0.58	6.25	(233, 281)
	SIAS	2(+0)	2	0.58	6.25	(64, 279)
	OPS	2(+0)	2	0.58	6.25	(320, 323)
	Others	6(+3)	9	0.29^a^	3.13^a^	fMRI (58), NDS (353), GPES (266), PSQ (297), SSS (324), SOEQ (351), [OAD (233), ESS (96), mBI (311)]
	**Total (Σf)**	**32(+3)**	**Σx** **=** **32**	9.22	100.00	
Stroke impact	SIS	14(+0)	14	4.03	45.16	(24, 46, 96, 103, 118, 135, 150, 153, 163, 166, 208, 279, 284, 289)
	SF-36	4(+0)	4	1.15	12.90	(22, 236, 242, 286)
	BRS	5(+0)	5	1.44	16.13	(65, 86, 192, 193, 230)
	NHP	3(+0)	3	0.86	9.68	(252, 322, 326)
	Death	2(+0)	2	0.58	6.45	(109, 294)
	Others	3(+0)	3	0.29^a^	9.68^a^	Complications (350), GHQ (146), SA-SIP (269)
	**Total (Σf)**	**31(+0)**	**Σx** **=** **31**	8.93	100.00	

n^a^, n% for each of the outcome measures; x, exclusive frequency; y, repeated frequency, f, sum of x and y; %=(f/347*100); Rel % =(f/Σx*100).

*SIS, Stroke Impact Scale; SF-36, 36-item Short Form Survey; NHP, Nottingham Health Profile; SA-SIP, Stroke Adapted Sickness Impact Profile; SSS, Scandinavian Stroke Scale; EQVAS, Euroquol visual analog scale; HUI, Health Utilities Index; NQoL, Nocturnal QoL Questionnaire; QoLI, Quality of Life Index; GHQ, General Health Questionnaire; AAP, Adelaide Activities Profile; COPM, Canadian Occupational Performance Measure; NLQ, Nottingham Leisure Questionnaire; RNLI, Reintegration to Normal Living Index; TRIP, Test Ride for Investigating Practical fitness to drive; RTWQ, Return to Work Questionnaire; LIFE-H, Assessment of Life Habits; PASPID, Physical Activity Scale for individuals with Physical Disabilities; LHS, London Handicap Scale; IPA, the Impact on Participation and Autonomy; NIHSS, National Institute of Health Stroke Scale; CNS, Canadian Neurological Scale; (m)RS, (modified) Ranking Scale; RLOC, Recovery Locus of Control Scale; BRS, Brunnstrom Recovery Scale; SIAS, Stroke Impairment Assessment Set; OPS, Orpington Prognostic Scale; fMRI, functional Magnetic Resonance Imaging; NDS, Neurologic Deficit Scale; PSQ, Patient Satisfaction Questionnaire; SOEQ, Stages of Exercise Questionnaire; OAD, Observer Assessed Disability; ESS, European Stroke Scale; mBI, modified Barthel Index*.

### Synthesized Themes for Stroke Intervention

#### Motor Relearning Therapy (Motor Function, Muscle Strength, Balance and Muscle Tone, Activities of Daily Living, Gait, and Mobility)

One hundred and sixty trials examined the effects of various neurorehabilitation techniques on trunk, upper and lower extremity motor function while 52, 50, and 41 studies were on muscle strength, balance and muscle tone, respectively. Also included in the motor relearning interventions were the 208 trials on Activities of Daily Living (ADL), 108 and 51 trials on gait and mobility, respectively. These neurorehabilitation techniques include innovatively high technology interventions such as robotic therapy (11–50), transcranial direct current stimulation (16, 29, 51–64, 344), transcranial magnetic stimulation (66–94), functional electrical stimulation (95–112), virtual reality (113–129), and video game (130–132). Many of these trials reported “within-group” improvement in motor functioning outcomes in both intervention and control groups (usually conventional therapy) with no “between-group differences” in these outcomes. Similarly, most of the identified traditional and relatively low-technology neurorehabilitation techniques such as body weight supported treadmill (133–143), occupational therapy (33, 56, 80, 123, 144–150), constraint induced movement therapy (23, 147, 151–184), mirror therapy (39, 68, 185–197), mental therapy (145, 198–202), task oriented training (20, 24, 83, 123, 144–150) muscle strengthening and stretching exercises (73, 221–235) had significant effects on improving motor functioning.

#### Cognitive Therapy

Eight trials (116, 236–242) on the efficacy of post-stroke cognitive rehabilitation were reviewed. Three studies utilized technology-based techniques namely virtual reality (116), lumosity brain trainer (239), and continuous positive Airway Pressure (CPAP) (232). Other trials utilized relatively low technology interventions such as comprehensive rehabilitation training (236), experential/traditional music (237), aerobic exercise (238), lifestyle course (240), and workbook based intervention (242). While virtual reality and CPAP resulted in significantly better improvement in Neurocognitive functions when compared with conventional therapy, lumosity brain trainer had no significant effect on cognitive function. Among the relatively low technology interventions, comprehensive rehabilitation training, experiential/traditional music and workbook based interventions significantly improved cognitive functions of stroke survivors more than conventional therapy.

#### Speech Therapy

Four studies (84, 243–245), on therapies for post-stroke aphasia and dysarthria were reviewed. One study (243), compared the effect of music therapy combined with Speech and Language Therapy (SLT) on aphasia with SLT alone and found that the combined therapy significantly improved speech and language functions of aphasic stroke patients. However, best practice communication therapy protocol delivered by speech and language therapist (244) and standard speech therapy (245) had no significantly different effect on functional communication ability of stroke survivors. Also, a trial that evaluated the effects of repetitive transcranial magnetic stimulation (rTMS) on aphasia found no between- group difference between recipients of the intervention and those who received sham rTMS (84).

#### Aerobic Exercise/Physical Activity Based Training

Forty four studies (48, 51, 205, 237, 246–289) evaluated the effects of a variety of aerobic exercises and physical activity based interventions on different aspects of the activity construct. Activities examined in the reviewed studies included mobility (255, 258, 261, 263, 265, 269, 270, 272, 278, 281, 282), general activities of daily living as assessed with Barthel Index or its modification (257, 261, 265, 269, 272, 277, 282, 285, 287–289), or Functional Independence Measure (51, 264, 278); and upper limb functional activities (51, 256, 257, 261, 274).

The interventions trialed included body weight supported treadmill training (274), Bobath programme (280), proprioceptive neuromuscular faccilitation (246), interval/continuous aerobic exercise (248), accelerometer mediated walking (259), intensive/regular exercises (261, 276, 277), early/late training (268), fast/slow training (263), motor imagery activities (269, 272), sit-to-stand-training (205, 273), transcranial direct current stimulation (51), hydrotherapy (247), accupunture (286), orthotic device (260) augumented physiotherapy (257, 281, 282, 284, 290).

#### Other Therapies

These include participation based therapy (290–294), quality of life centered care (240, 295–310, 345), community based rehabilitation (311–315), home based rehabilitation (132, 193, 316–335), self-management (336, 337), family or care giver-led training (340–342, 350, 353, 354), telerehabilitation (317, 343, 346, 349), and early therapy/rehabilitation (174, 338, 339, 347, 348, 351, 352, 355, 356).

## Discussion

### Interventions

#### Motor Relearning Therapy

Several motor relearning interventions have been proposed for use in stroke rehabilitation to enhance motor function, activity and participation recovery after stroke and these interventions can be broadly categorized as traditional/conventional and emerging trends. Many of the trials included in this review largely confirmed the efficacy of conventional (sometimes termed “usual care”) interventions for the improvement of upper and lower limb muscle strength, balance, and coordination. Interventions found to be effective include task-specific training (138), therapist-assisted locomotor training (144). The efficacy of other interventions that may not fit into the category of conventional therapies but which also do not necessarily require high instrumentation was also reported. These include constraint- induced movement therapy (164, 172, 178), mirror therapy (185, 196, 197), and task oriented training (209, 210, 215, 216). Although many of these interventions are not costly especially because they do not require high technology gadgets and equipments, they can however be labor intensive. In most Low and Middle Income Countries (LMICs) where gross shortage of qualified rehabilitation specialists and centers appears intractable, the utilization of effective but personnel-demanding rehabilitation strategies may not be sustainable and pragmatic. The difficulties associated with utilizing conventional and low technology therapies in LMICs are further made worse by the increasing incidence and prevalence of stroke in these settings (357). The provision of conventional rehabilitation after stroke in these resource-limited settings would therefore require an aggressive focus by all stakeholders including government of those countries, policy-makers, the rehabilitation professionals, non-governmental organization and foreign collaborators on training and employment of needed rehabilitation manpower. It might be argued that while the findings of this review support the utility of pragmatic, conventional stroke rehabilitation solutions, there is a likelihood that what is considered conventional or routine care in many of the reviewed studies may not exactly depict usual care in LMICs. However, a recent systematic review of stroke rehabilitation interventions that are currently in use in LMICs provided evidence on the efficacy of low-cost physical rehabilitation interventions in improving post-stroke functional outcomes (358). Standardization of what constitutes effective conventional stroke therapies would therefore be required in LMICs and can be achieved by ensuring that training curricula for rehabilitation disciplines and relevant clinical practice guidelines place emphasis on effective evidence-based stroke rehabilitation interventions.

It is important to note that the shortage of rehabilitation professionals in LMICs is however not solely due to the non-availability of these professionals but also results from the limited employment opportunities or openings. Also worthy of mention is the limited or outright lack of utilization of lower grade health workers that could provide basic and less-specialized stroke treatments. A typical example is that of Nigeria, the most populous country on the African continent, where physiotherapy assistants are largely not in place in the country contrary to the practice in many high-income countries (359). Another case in point is the under-utilization of post-qualification internship programme that provides a pool of fresh graduates that can augment rehabilitation personnel requirements, with many health institutions grossly rationing the employment of interns due to lack of funds for remuneration and this renders such entry-level professionals under-employed and under-utilized. The adoption of a stroke quadrangle strategy (360), that proposes pragmatic solutions on issues of rehabilitation professional shortage is therefore required. However, another strategy that has gained traction in recent times is to circumvent manpower demanding conventional therapies and adopt technology driven alternatives.

Many emerging high technology stroke rehabilitation strategies have been trialed. In this review, we found several RCTs that evaluated the effect of robotic training, virtual reality training, transcranial direct current stimulation (tDCS), transcranial magnetic stimulation, functional electrical stimulation on various aspects of physical functioning. Many of these interventions are expensive and are not affordable in settings with insufficient financial resources. Although many of the trials show that these interventions despite their high cost are not more effective than conventional therapies, a likely advantage is that automated interventions like robotic therapies require minimal input from rehabilitation professionals in terms of time and efforts. Therefore, given the efficacy of robotic therapy and the fact that its utilization in stroke rehabilitation may mitigate the labor intensive and personnel tasking nature of many conventional therapies, affordable stroke rehabilitation robotics that are feasible for use in low-resource countries are being produced, and assessed for efficacy (361).

#### Cognitive Therapy

Cognitive reserve (defined as the ability to cope with brain damage) has been postulated to influence functional ability (362), and this buttresses the need for cognitive therapy during stroke rehabilitation. Similar to what obtains with the therapies for motor relearning, interventions that address post-stroke cognitive function are available in low technology and high technology forms (363). While virtual reality was reported to result in marked improvement in post-stroke cognitive functions (116), and interactive video game a potentially beneficial treatment (249), computer-based cognitive training was neither superior to mock training nor waiting list in its effect on subjective cognitive functioning (250). Hence, the utilization of technology in post-stroke cognitive rehabilitation may not guarantee a positive outcome. The use of aerobic exercise to address post-stroke cognitive impairment as was reported (238), may be considered as a more practical approach in LMICs. There is however a dearth of studies on effective post-stroke cognitive rehabilitation strategies from LMICs (1). Given the burden of post-stroke cognitive impairment especially in terms of its prevalence (364), and its potentially negative impact on other important constructs such as activities of daily living (365), participation (366), and quality of life (367), there is an urgent need to identify effective interventions that can be easily incorporated into real-life practice in LMICs.

#### Speech Therapy

The use of regular communication mechanism was found to be more effective in promoting recovery from aphasia compared to intensive aphasia therapy (251). Similarly, the use of enhanced communication therapy (245), and rTMS (84) to address the speech function of stroke patients with aphasia did not confer any additional advantage on its recipients. Although these findings may suggest that further studies are required to identify effective therapies for post-stroke speech impairments, it is important to note that the efficacy or otherwise of therapies for post-stroke speech impairments also depends on the lesion site (368) and severity of the brain injury. Therefore, identifying pragmatic solutions for recovery of speech function after stroke in LMICs may need to be accompanied by availability of neuroimaging equipment that will aid in accurately diagnosing and identifying the site and extent of the brain injury.

#### Quality of Life Centered Care

Quality of life of stroke patients represents a broad index of stroke recovery (369) and its improvement is considered as the ultimate goal of stroke rehabilitation (360). The findings of this review which showed that many of stroke trials targeting other constructs such as motor function (367), cognition (370), and functional activity (138) also evaluated the global effect of such interventions on the post-stroke quality of life is therefore not surprising. Many of the interventions that were effective in improving motor function, activity and participation were also found to improve quality of life. This is not unexpected as several observational studies have shown that many of these specific functioning constructs significantly influence or predict the multi-dimensional construct—quality of life even in other neurological conditions (371). Hence, since many of the interventions that were found to facilitate the various components of post-stroke functioning also resulted in significant improvement in post-stroke quality of life, pragmatic solutions for stroke recovery may also represent pragmatic solutions for improved quality of life after stroke.

### Models of Stroke Rehabilitation

#### Task Shifting

Task shifting has been described as an attractive option for healthcare optimization and sustainability in LMICs (372, 373). It is a process of moving or shifting appropriate task to health workers with shorter training and fewer qualifications (371). Task shifting involves deliberate delegation of specific task(s) to the least costly health worker in order to free up specialists who are in limited supply to provide more complex care for people who critically require such care (374).

The need to explore task shifting of rehabilitation activities to non-health workers such as informal or family caregivers as a potentially sustainable alternative to conventional rehabilitation, and an affordable strategy in meeting rehabilitation demands in LMICs has also been identified (375–377). The trials included in this review however did not find sufficient evidence and justification for the adoption of such a task shifting model in stroke rehabilitation. The ATTEND trial in India (a middle-income country) examined the effectiveness of a family-led stroke rehabilitation model in improving clinical outcomes with the conclusion that the model was not superior to usual care in terms of important outcomes such as death, dependency and re-hospitalization, and potentially constitutes a waste of already limited resources (378). Similarly, the TRACS trial found no significant difference in stroke patients' recovery, mood and quality of life, and caregivers' burden and perceived cost-effectiveness of a stroke caregivers training programmes (379). In line with the suggestions of the authors of the ATTEND trial, future studies will be required to examine if task-shifting in stroke rehabilitation to healthcare assistants would yield better clinical outcomes. For example, the findings of a previous study in Nigeria showed that non-neurologist healthcare workers were receptive to, and substantially assimilated stroke-specific knowledge disseminated at a task shifting training workshop (380).

#### Community-/Home-Based Rehabilitation

Community rehabilitation may constitute a cost-effective and pragmatic model of stroke rehabilitation in LMICs. Traditionally, rehabilitation services for stroke patients are offered in hospitals which are largely urban-based and inaccessible to many stroke survivors, especially those in rural areas. Improving accessibility to rehabilitation services requires implementation of existing public health programmes developed by the World Health Organization for stroke prevention and treatment (381). These include primary health care and its community-based rehabilitation counterpart (382), and home-based rehabilitation. One of the trials we reviewed, the Locomotor Experience Applied Post-Stroke (LEAPS) trial, showed that home-administered strength and balance training resulted in improvement in functional walking among community-dwelling stroke survivors. Furthermore, the home-based exercise protocol utilized in the LEAPS trial was found to be as effective as the more expensive institutional-based body-weight-supported treadmill training and hence can be considered practical and feasible for adoption in LMICs (138).

An intervention programme comprising task-specific exercises was similarly associated with improvement in motor function, postural balance, community reintegration, quality of life, and walking speed among stroke survivors treated at a primary health center in Nigeria (383). Furthermore, the Nigerian study showed that physiotherapy services delivered at primary health centers in the community resulted in similar outcomes as home-based physiotherapy services (367). Thus, home exercise interventions seem a more pragmatic form of therapy for stroke survivors with a higher likelihood of compliance (138). Community-/home-based rehabilitation can therefore be regarded as effective models for improving access to stroke care, care efficiency, coordination, and continuity in LMICs.

#### Self-Management

Though rarely used in the context of stroke (384), application of self-management interventions for stroke rehabilitation has stimulated research interest in recent years (337), Despite the fact that stroke is an acute event, stroke survivors experience physical and psychosocial challenges in the recovery trajectory which renders stroke a chronic condition (385). Challenges faced include depression, functional and mobility disability, reduction in life roles, and a lack of social support (386). Yet, rehabilitation for stroke survivors are targeted at improving physical function, while minimal attention is given to the psychosocial consequences of stroke (385, 386). To overcome these challenges, rehabilitation strategies that support stroke survivors to manage their health and lives and maximize their full potentials are necessary (337). Self-management is an emerging strategy for engaging stroke survivors in their own care. Evidence suggests that self-management programmes can impact on clinical outcomes and psychological health of patients with a range of long-term conditions (387, 388). It could influence an individual's ability to cope with their condition, and enhance quality of life (387). Self-management in stroke rehabilitation requires conscious effort by survivors themselves to deal with stroke-related disabilities, prevent stroke recurrence, and overcome challenges of long-term recovery (111). However, evidence base for its effectiveness in stroke care is still emerging (337, 389).

#### Tele-Rehabilitation

Tele-rehabilitation entails remote delivery and supervision of rehabilitation services (390). It can be considered as a viable rehabilitation alternative for stroke patients with limited access to usual rehabilitation services resulting from logistical, financial, and geographical barriers to rehabilitation centers (391). The studies included in this review showed that telerehabilitation was effective in improving falls efficacy (349), quality of life (390) and reducing depression (390), and carer stress (317) after stroke. Translation of these budding opportunities and existing evidence-based interventions into pragmatic and cost-effective solutions in LMICs remains a huge challenge. Research efforts are needed to develop cost-effective robotic devices that can perform the above functions in harsher environments characterized by extreme economic hardship (per country), intermittent electricity supply and limited expert supervisors (361). Technology assisted rehabilitation as a viable option to task-shifting is the subject of current trials (392). The feasibility and acceptability of using smart phone for self-management of stroke patients has been evaluated (393).

### Limitation

A major perceived limitation of this study is the loose thematic inclusion of some constructs such as quality of life, stroke severity, recovery, and impact under the participation component of ICF.

## Conclusion

This review showed that various approaches to stroke rehabilitation that may be adopted in LMICs exist. These however must be considered within the context and framework of the health system and available resources. Studies on how to adapt existing approaches and to develop novel ones for stroke rehabilitation in LMICs are needed. However, since many of the expensive innovative stroke therapies obtained in the review lack comparative advantage over low-cost traditional ones in terms of efficacy, the emphasis in LMICs should be the strengthening and expansion of the rehabilitation workforce, and provision of adequate rehabilitation centers to ensure access to effective conventional stroke rehabilitation solutions in those settings. Efforts at designing and producing low-cost versions of the expensive innovative stroke rehabilitation solution that will be compatible with the socio-economic, built and energy environment of LMICs should however also be encouraged, supported and funded.

## Author Contributions

EE contributed in the conceptualization of this study, sorting and extraction of data, quantitative analysis, and editing of the final manuscript. PO contributed in the conceptualization, data sorting and extraction, and qualitative analysis and draft preparation. KN took part in the conceptualization of the study, data sorting and extraction, and editing of the manuscript. OO contributed in the literature search and writing of the discussion and conclusion. VO took part in the data sorting phase and in writing the introductory section. TH was involved in the conceptualization of study and consultation and mentoring. MO was involved with the conceptualization, organization of the team, consultation and mentoring, editing and final approval of the final version of the manuscript.

## Conflict of Interest

The authors declare that the research was conducted in the absence of any commercial or financial relationships that could be construed as a potential conflict of interest.
