# Supplementary material for: Pragmatic Solutions for Stroke Recovery and Improved Quality of Life in Low- and Middle-Income Countries—A Systematic Review
Source: Front Neurol. 2020 Jun 25;11:337. doi: 10.3389/fneur.2020.00337 (PMC7336355; doi:10.3389/fneur.2020.00337)
Supplement: Supplementary file 1 [file Data_Sheet_1.pdf]

Robot Therapy

Ref	Study (Authors, year	Country	Participants	Interventions/ Model of care	Control/ comparison	Outcomes assessed/ outcome measures	Results	Conclusion	Quality score	
**11**	Park and Chung. (2018)	Korea	40 patients<br>Age range:<br>VRGT=55.58±10.42<br>ARGT=56.66± 4.39<br>Control=57.50± 9.90<br>PSD= **6 months**<br><br>**TOS= haemorrhagic/ ischaemic combined**<br><br>VRGT=3/9<br>ARGT=8/4<br>Control=9/7	Virtual reality robot assisted gait training (VRGT), Auditory stimulation robot-assisted gait training (ARGT)	General gait training	- Medical Research Council (MRC) for the grading of muscle power<br>- Berg balance scale (BBS) used to evaluate balance abilities and fall risk.<br>- Timed up and go test (TUG) used to evaluate activities related to walking including dynamic stability<br>- 10-meter walk test (10MWT) usedto investigate the extent of gait ability.<br>- Fugl-Myer Assessment (FMA) for assessing motor impairment<br>- Modified Barthel Index (MBI) to evaluate functional outcome	BBS, TUG, and 10MWT scores significantly improved post-intervention ($p < 0.05$), and the control group also had significantly improvement in all areas post-treatment ($p < 0.05$). VRGT had significantly improved MRC and FMA scores when compared with the auditory stimulation. Also, VRGT significantly improved MRC, BBS, TUG, 10MWT and FMA when compared with control group ($p < 0.05$).	The results of this study showed improve balance and gait abilities after VRGT compared with general physical therapy and were found to be effective in enhancing the functional activity of persons with stroke.	1	
**12**	Daunoraviciene et al, (2018)	Lithuania	Thirty four stroke patients<br>Age range:<br>Experimental group= 65.88 ± 4.87years<br>Control group = 65.47 ± 4.05years<br>PSD: NR	Robotic training	Conventional therapy	Hamilton Rating Scale for Depression (HAD) and the Hamilton Rating Scale for Anxiety, Addenbrooke's Cognitive Examination-Revised (ACE-R) of global cognitive function recovery after stroke, the Modified Ashworth Scale (MAS) as a	Patients assigned to the EG showed a statistically significant improvement in upper extremity motor function when compared to the CG by FIM ($P < 0.05$) and ACER ($P < 0.05$). No improvement s with the other outcomes	Task-oriented robotic training in rehabilitation setting facilitates better recovery not	1	

			TOS= both ischaemic and haemorrhagic (I/H) Experimental group=13/4 Control group= 15/2			measure of muscle tone and ROM assessment of the shoulder, elbow and wrist.		only of the motor function of the paretic arm but also of the cognitive abilities in stroke patients compared to conventional therapy.		
**13**	Villafañe et al, (2018)	Italy	Thirty-two patients<br>- Age range: Experimental group= 67 (11)yrs Control group=70 (12)yrs<br>- PSD=between 0.5 and 12 months<br>TOS(ischaemic/ haemorrhagic) Experimental group=12/4 Control group=12/4	30 minutes of passive mobilization of the hand through the robotic device Gloreha	30 minutes of physiotherapy (PT) and occupational therapy (OT)	- National Institutes of Health Stroke Scale (NIHSS), for stroke severity<br>- Modified Ashworth Scale (MAS), for Grading Spasticity<br>- Barthel Index (BI), for functional outcome<br>- Motricity Index (MI) was usedto measure motor strength<br>- Short version of the Disabilities of the Arm, Shoulder and Hand (QuickDASH)<br>- Visual analog scale (VAS) measurement was used to assess intensity of hand pain	A significant effect of time interaction existed for NIHSS, BI, MI, and QuickDASH, after stroke immediately after the interventions (all, P < .001)	Robot-assisted mobilization performed in conjunction with traditional PT and OT is as effective as traditional rehabilitation	4	
**14**	Han et al (2016)	Korea	60 patients<br>- Age range (years) Intervention group=67.89± 14.96 Control group=63.2± 10.62<br>- PSD(days): Intervention	Robot-assisted gait therapy and 30 minutes of conventional RT	Conventional rehabilitation therapy (RT)	- Brachial–ankle pulse wave velocity<br>- Peak aerobic capacity<br>- Modified Barthel Index (K-MBI) was used to assess performance in basic ADL<br>- Berg balance scale (BBS) was used to assess the balance function	Peak aerobic capacity (VO2peak), HRpeak, and ETT duration were significantly increased in the RAGT group, whereas the control group did not exhibit any significant changes after 4 weeks. Repeated-measure ANOVA revealed that participants in the RAGT had a higher peak aerobic	Robot-assisted gait therapy combined with conventional rehabilitation therapy represents an	2	

			group=21.56± 7.98<br>Control group=18.10± 9.78<br>- TOS: (ischemic/hemorrhage)<br>Intervention group= 17/13<br>Control group=16/10			- Fugl–Meyer Assessment (FMA-LL) was used to assess motor function of both lower extremities including hip, knee, and ankle	capacity, as determined by measurements of peak VO2peak, than the control group after 4 weeks of intervention (P= 0.009)	effective method for reversing arterial stiffness and improving peak aerobic capacity in subacute stroke patients with totally dependent ambulation.		
**15**	Moroneet al. (2016)	Italy	Forty-four patients<br>-**Age range [years]**:<br>Intervention group= 61.50 ± 10.97<br>Control group= 64.09 ± 16.27<br>-**PSD**:<br>Intervention group=69.20 ± 28.93<br>Control group=59.68 ± 36.03<br>**-TOS** :Ischaemic/Haemorrhagic<br>Experimental group=18/3<br>Control group=14/7	Servo-assistive robotic rollator (i-Walker) training	Conventional walking oriented therapy	10-m and 6-min timed walking tests were used to measure the walking capacity.<br><br>Spasticity assessed with a modified version of the Ashworth scale<br><br>Global ability measured with the Barthel Index (BI)<br><br>Global impairment assessed with the Canadian Neurological scale.	Treatment effectiveness was higher in the iWG group in terms of balance improvement (Tinetti: 68.4 ± 27.6 %<br>vs. 48.1 ± 33.9 %, p = 0.033) and 10-m and 6-min timed walking tests (significant interaction between group and time: F(1,40) = 14.252, p = 0.001; and F(1,40) = 7.883, p = 0.008, respectively). When measured, latero-lateral upper body accelerations were reduced in iWG (F = 4.727, p = 0.036),<br>Results were only reported of walking performance.	A robotic servo-assisted i-Walker improved walking performance and balance in patients affected by mild/moderate stroke, leading to increased gait stability and reduced falls in the community.	4	
**16**	Straudi et	Italy	**Age (years)**<br>Mean (SD):<br>Experimental	tDCS combined with upper	RAT + sham-tDCS.	Fugl-Meyer Assessment-Upper Extremity (FMA-UE) to assess arm motor recovery	recovery stage in sham-tDCS group (= 9.20, df = 1,9; < 0.05; adjusted span style="font-	The additional use of	3	

	al, (2016)		group= 52.7 (16.0)<br>Control group=64.3 (9.7)<br>**PSD (SD)days**:<br>Experimental group= 40.7 (35.1)<br>Control group=78.2 (61.9)<br>**TOS (ischaemic/ haemorrhagic**<br>Experimental group=10/2<br>Control group=9/2	extremity robot-assisted therapy (RAT)		Box and Block Test (BBT) was used to evaluate gross motor function<br><br>Motor Activity Log (MAL) was administered to quantify real-world arm use in activities of daily living	family: MinionMath-Capt;font-size:7pt;color:rgb(35,31,32);font-style:normal;font-variant:normal;"R2 = 0.45) and stroke location in real-tDCS group (= 8.48, df = 1,10; < 0.05; adjusted span style="font-family: MinionMath-Capt;font-size:7pt;color:rgb(35,31,32);font-style:normal;font-variant:normal;R2 = 0.40)	bilateral tDCS to RAT seems to have a significant beneficial effect depending on the duration and type of stroke.		
**17**	Lee et al, 2015	China	P: patients with chronic stroke between 2012-2014<br>Sample size=39 (E=20, C=19),<br>Mean age (Yrs)= (E= 54.07±11.85, C= 53.75±9.11);<br>Age range=20to 80 years;<br>PSD(months): E = 25.40±17.09, C = 27.95±16.20;<br>TOS(ischemic/hemorrhagic):<br>E = 10/10, C =11/8	Robot Assisted Therapy (RT) + Neuromuscular Electrical Stimulation (RT+ES)	RT + Sham	-Motor Impairment (UE-FMA),<br>- Spasticity (mAS),<br>- Motor Function (WMFT),<br>- Daily Function (MAL), and<br>- QoL (SIS).	Compared with the RT + Sham group, the RT + ES group demonstrated greater improvements in wrist flexor MAS score, WMFT quality of movement, and the hand function domain of the SIS. For other outcome measures, both groups improved significantly after the interventions, but no group differences were found.	RT + ES induced significant benefits in reducing wrist flexor spasticity and in hand movement quality in patients with chronic stroke.	5	
**18**	Hu et.al, 2015	Honkon g	P: hemiplegic subjects with chronic stroke<br>Sample size=26 (E=11, C=15),<br>Mean age (Years): E= 45.6 ± 11.4,C= 49.2 ± 14.7<br>PSD (Years): E=	EMG-driven NMES Robot (NMES Robot Group)	EMG-Driven Robot (Robot Group)	- UL Motor impairment (UE-FMA),<br>- UL Spasticity (mAS),<br>- UL Function (ARAT)	- The improvement in UE-FMA (shoulder/elbow, wrist/hand) obtained in the NMES robot group was significantly more than the robot group (P <0.05).<br>- Significant improvement in ARAT was achieved in the NMES robot group (P < 0.05) but absent in the robot group.	The NMES robot–assisted wrist training was more effective than the pure robot.<br>The	2	

			4.7 ± 5.2 (1.5-10),<br>C= 4.2 ± 3.6 (2-8)<br>TOS(ischemic/hemorrhagic):<br>E = 9/6<br>C =8/3				- NMES robot–assisted training showed better performance in releasing muscle co-contraction than the robot-assisted across the training sessions ($P < 0.05$).	additional NMES application in the treatment could bring more improvements in the distal motor functions and faster rehabilitation progress.		
19	Sale et al., 2014a	Italy	**Sample size:** I= 26 C= 27<br><br>**Age Range:** NR<br><br>**Mean Age:**<br>I=67.7 ±14.2<br>C= 67.7 ±14.2<br><br>**PSD:** NR<br><br>**TOS(ischemic/hemorrhagic):**<br>I = 25/1<br>C =21/6	Standard therapy with additional 30 sessions of robot-assisted therapy (EG)	Standard therapy with additional 30 sessions of usual therapy were (CG)	Primary: Motor impairment (Fugl-Meyer Test (FM)); spasticity (MAS for shoulder [MAS-S] and elbow [MAS-E] joint)<br><br>Secondary: passive range of motion [pROM]; impairment (Motoricity index [MI])	There was a statistically significant improvements in MAS-S ($p = 0.004$) and MAS-E ($p = 0.018$) in the intervention group. A non statistically significant decreasing trend of MAS-S and increasing trend of MAS-E was found in the CG. FM improved significantly in both groups (EG: $p <0.0001$, CG: $p < 0.0001$). There was also a significant difference in mean FM favoring the intervention group at both post 15 session and post 30 sessions ($p < 0.0001$ and $p < 0.001$, respectively).<br>- The secondary outcome showed statistically significant improvements in pROM ($p < 0.0001$) and MI ($p < 0.0001$) in the EG, whereas the CG showed statistically significant improvements in MI ($p < 0.0001$) and a not statistically significant increase in pROM. There was an increase in pROM just after the first 15 sessions ($p < 0.0001$) in	Robot-assisted upper limb rehabilitation treatment can contribute to increasing motor recovery in subacute stroke patients	3	

							the EG whilst pROM did not change in the CG. As regards MI, a greater improvement in the EG than the CG after the first 15 sessions ($p < 0.0001$ and $p = 0.008$, respectively) was observed.			
**20**	Lemmens et al., 2014	Belgium & Netherland	**Sample size:** I= 8<br>C= 8<br>**Age Range:** NR<br>**Mean Age:**<br>I=63.5<br>C= 55.0<br>**PSD(months):**<br>I=12.5<br>C=25.5<br>**TOS(ischemic/hemorrhagic):**NR	task-oriented robot-supported arm-hand training	task-oriented arm-hand training without robot support	Amount of arm use (Fugl-Meyer Motor Assessment [FMMA], Action Research Arm Test [ARAT] and Motor Activity Log [MAL])	Both groups showed no significant improvement in the amount of arm use ($p > 0.05$) from all the measured outcomes	There is no significant changes in actual amount of arm-hand use after task-oriented training, with or without robot-support	3	N
**21**	Salea et al., 2014b	Italy	**Sample size:** I= 11<br>C= 9<br>**Age Range:** NR<br>**Mean Age:**<br>I=67.0±12.4<br>C= 72.56±8.98<br>**PSD(days):** 30±7<br>**TOS(ischemic/hemorrhagic):**<br>I = 8/3<br>C = 7/2	intensive robot-assisted hand therapy performed using the Amadeo Robotic System	Intensive occupational therapy executed by a trained physiotherapist	Primary; Fugl-Meyer Scale (FM); Box and Block Test (BB);<br><br>Secondary: Medical Research Council Scale for Muscle Strength (hand flexor and extensor muscles) (MRC); Motricity Index (MI); modified Ashworth Scale for wrist and hand muscles (MAS); Barthel Index	The intervention group showed a statistically significant improvement in the FM (P=0.0039), the BB (P=0.0185), MI (P<0.0001), MRC (P<0.0001) and MAS (P=0.0025). the control group also showed a statistically significant improvements in the FM (P<0.0001), BB (P=0.0086), MI (P=0.0303), and MRC (P=0.001)	The robotic rehabilitation treatment may contribute toward the recovery of hand motor function in acute stroke patients. The effect it however comparable with intensive occupational therapy	3	
**22**	Ang et al., 2015	Singapore	**Sample size:** I= 11<br>C= 15<br>**Age Range:** NR<br>**Mean Age:**<br>I=48.5 ± 13.5	EEG-based motor imagery (MI) brain-computer	Manus robotically guided shoulder and elbow	Motor impairment (FMMA)	both groups demonstrated significant gains in the primary outcome, total FMMA score ($p < 0.05$); However, there were no significant intergroup differences	BCI-Manus therapy is effective and safe for arm rehabilitation	3	

			C= 53.6 ± 9.5 **PSD(days):** I=383.0 ± 290.8 C=234.7 ± 183.8 **TOS(ischemic/hemorrhagic):** I = 5/6 C = 5/10	interface (BCI) with Manus robotic feedback (BCI-Manus)	reaching exercises		at any time point during the study (*P* > .05)	after severe poststroke hemiparesis. Motor gains were comparable to the control intervention		
**23**	Hsieh et al., 2014	Taiwan	**Sample size:** I: G1 = 16 G2= 16 C= 16 **Age Range:** NR **Mean Age:** I: G1 = 54.41 ±7.77 G2= 52.34 ±13.20 C= 54.12 ±9.98 **PSD(months):** I: G1 = 20.56 ±13.95 G2= 23.56 ±15.43 C= 27.81 ±19.07 **TOS(ischemic/hemorrhagic):** I: G1 = 7/9 G2= 12/4 C= 8/8	G1 = Robot assisted therapy (RT) with the distributed form of constraint induced therapy (CIT) (RT + dCIT)<br><br>G2 = Robot assisted therapy (RT)	Conventional rehabilitation (CR)	Primary: motor impairment (FMA) and motor function (Wolf Motor Function Test (WMFT) with subscales: Functonial Ability subscale [WMFT-FAS] and performance time subscales [WMFT-TIME]).<br><br>Secondary: ADL (Motor Activity Log (MAL)) and amount of arm movement (accelerometers)	The three treatment groups showed significant within group improvements over time on the FMA total, distal, and proximal scores (all P <0.05). A significant difference in mean FMA was also found between the three groups (p < 0.01). Post hoc analyses showed that the RT + dCIT group had significantly greater gains on the FMA total score than the RT (P = 0.05) and CR (P <0.01) groups, and the RT group had significantly larger gains than the CR group (P = 0.04).The 3 groups demonstrated significant within-group improvements on the WMFT (all P <0.05). In addition, between-group comparisons showed the three groups differed significantly on the WMFT-FAS (P = 0.01). Post hoc analyses revealed that the RT + dCIT group improved significantly more than the RT and CR groups on WMFT-FAS (both P = 0.01). Moreover, there were nosignificant between-group differences among the three groups on the WMFT-TIME (P = 0.28). The three groups had significant within-group	RT in sequential combination with CIT led to additive effects on participants' motor ability and functional ability to perform motor tasks after stroke.	3	

							improvements over time on the MAL-AOU and MAL-QOM (all P<0.05) The improvements on the MAL and accelerometers were not significantly different among the three groups.			
**24**	Timmermans et al., 2014	Netherlands	**Sample size:** I= 11 C= 11 **Age Range:** NR **Mean Age:** I=61.8±6.8 C= 56.8±6.4 **PSD(years):** I=2.8±2.9 C= 3.7±3.0 **TOS(ischemic/hemorrhagic):**NR	task-oriented robot-assisted arm-hand training	task-oriented non-robotic arm-hand training	Arm hand function (Fugl Meyer Motor Assessment (FMMA)), upper limb activity (Action Research Arm Test (ARAT)); arm hand activity (Motor Activity Log (MAL)); quality of life (EuroQol-5D and SF-36).	No between group differences were found with regard to arm hand function (FMMA), arm hand capacity (ARAT), and self-perceived arm-hand performance (MAL). Both groups did not show any significant improvement in FMMA. With regard to the ARAT, a significant post treatment improvement occurred in the experimental group (p = 0.008); There was no difference found in ARAT results between cessation of the training and the 6-month follow-up measurement. With regard to the MAL (total test result), there was a significant improvement in the control group (p = 0.008), and the experimental group (p = 0.005). No significant differences were found between cessation of training and the follow up measurement for any of the primary outcome measures. A significant difference was found between the control and experimental group with regard to the delta physical health scores of the SF-36 T2-T3 (p = 0.002). In the control group deterioration in perceived physical health scores was observed, while in the experimental group physical health scores improved after	Arm hand performance improved in chronic stroke patients, after eight weeks of task oriented training with or without robot assistance.	3	

							cessation of the training. No other between group differences were found between the control and the experimental group with regard to quality of life, neither for EQ-5D results, nor for SF-36 results.			
**25**	Hesse, et al, 2014	Germany	**Sample size:**<br>I= 25<br>C= 25<br>**Age Range:**<br>I= 43-85<br>C= 34-85<br>**Mean Age:**<br>I= 71.4 ±15.5<br>C= 69.7 ±16.6<br>**PSD(weeks):**<br>I= 4.5 ±1.7<br>C=4.5 ±1.4<br>**TOS(ischemic/hemorrhagic):**<br>I = 22/3<br>C =19/6	Practiced 30 minutes of robot-assisted arm group therapy (RAGT) Plus 30 minutes of individual arm therapy (IAT)	Practiced 2x30 minutes of individual arm therapy (IAT) only	Primary: reflexes and motor tasks (Fugl-Meyer Score)<br>Secondary: disability (ARAT); gross dexterity (BBT); upper limb muscle strength (Medical Research Council grades); ADL (BI)	Both groups showed significant improvement in FMA (p<0.05), however, the improvements did not differ between the groups (p > 0.05). Among the secondary outcomes, the Action Research Arm Test, Box & Block Test, Medical Research Council scores and the Barthel Index improved in both groups over time (p<0.05), but there were no group differences between four week and three-month assessment. For the Action Research Arm Test there was a a significant difference between the two groups (*p*=0.044) in favour of the control group at three month assessment .	RAGT in combination with IAT was equally effective as a double session of IAT regarding the restoration of upper limb motor functions in moderate to severely affected subacute patients with stroke	3	
**26**	Brokaw, et al, 2013	USA	**Sample size:**<br>1 = 7 C = 5<br><br>**Age Range:** NR<br><br>**Mean Age:** NR<br><br>**PSD(years):**NR<br><br>**TOS(ischemic/hemorrhagic):** NR	Robotics therapy then conventional rehabilitation after a 1 month washout period<br><br>NB: Randomised crossover trial	Conventional rehabilitation then robotic therapy after a 1 month washout period.	Motor impairment (Fugl-Meyer), functional limitation (Action Research Arm Test); gross functional ability (BBT)	Both groups showed significant improvements in the Fugl-Meyer (*P* = .013) and Box and Blocks tests (*P* = .028). The robotic intervention periods produced significantly greater improvements in the Action Research Arm Test than conventional therapy (*P* = .033). Gains in the Box and Blocks test from conventional therapy were larger than from robotic therapy in subjects who received conventional therapy after robotic therapy (*P* = .044).	Robotic therapy can elicit improvements in arm function that are distinct from conventional therapy and supplements conventional methods to improve outcomes	3	

**27**	Abdollahi et al., 2013	USA	**Sample size:** NR<br><br>**Age Range:** NR<br><br>**Mean Age:** NR<br><br>**PSD(months):** NR<br><br>**TOS(ischemic/hemorrhagic):**NR	Trained with haptic distortions via robot-rendered forces<br><br>NB: Randomized cross-over trial	Trained with graphic distortions via a virtual environment	Primary: Impairments (The arm motor section of the Fugl-Meyer (AMFM))<br><br>Secondary: functional ability (the Wolf Motor Function Test (WMFT)); manual dexterity (Box and Blocks); ROM (reaching test)	At post intervention, the intervention group showed greater significant increase in AMFM compared with control (*P* < .05). The WMFT also showed an overall significant improvement among all the participants (*P* < .012) and the intervention group showed a significant higher improvement in WMFT between the groups (p <0.05). However, no significant change was noted in manual dexterity nor ROM in both groups	Both interventions provided significant benefit to the measured outcomes with the intervention providing higher effect in post stroke patients.	3	
**28**	Wu, et al, 2013	Taiwan	**Sample size:**<br>I:G1 = 18<br>G2=18<br>C=17<br><br>**Age Range:** I=NR<br>C= NR<br><br>**Mean Age:**<br>I:G1 = 54.95 ±9.90<br>G2=52.21 ±12.20<br>C=54.22 ±9.78<br><br>**PSD(months):**<br>I:G1 = 19.00 ±15.51<br>G2=23.28 ±15.37<br>C=23.41 ±15.24<br><br>**TOS: ischemic/hemorrhagic : NR**	G1: unilateral robot-assisted arm training (URT)<br><br>G2: bilateral robot-assisted arm training (BRT)	Conventional rehabilitation (CT)	Primary: Normalized movement time [NMT], normalized movement unit [NMU], (kinematic analysis)<br><br>Secondary: UE motor function (WolfMotor Function Test (WMFT)); ADL (Motor Activity Log (MAL) and the ABILHAND Questionnaire)	For kinematic analysis, Arm motor control did not differ significantly in NMT and NMU variables among the 3 groups. BRT and the CT groups demonstrated a larger value of trunk contribution slope for the start part than the URT group (BRT vs. URT, P = .027; CT vs. URT, P = .032), Furthermore, no significant difference was found between URT and BRT for the middle part, and URT produced significantly a greater slope value than CT (P = .001), meaning that less trunk compensation was observed in the URT group than in the CT group. The 3 groups did not differ significantly in the WMFTFAS, MAL, and ABILHAND scores. However, significant differences were found in WMFT-Time (P = .035). The URT group, but not the CT group, showed less WMFT-Time (P = .011) than the BRT group	BRT and URT resulted in differential improvements in specific UE/trunk performance in patients with stroke. BRT elicited larger benefits than URT on reducing compensatory trunk movements at the beginning of reaching. In contrast, URT produced better improvements in UE	2	

								temporal efficiency		
**29**	Ochi et al, 2013	Japan	**Sample size:** NR<br><br>**Age Range:** NR<br><br>**Mean Age:** NR<br><br>**PSD:**<br>NR<br>**TOS: ischemic/hemorrhagic :** NR	Anodal transcranial direct current stimulation (tDCS) with robot-assisted arm training (AT) (tDCS(a) + AT)<br><br>NB: Randomized crossover trial	Cathodaltranscranial direct current stimulation (tDCS) with robot-assisted arm training (tDCS(c) + AT)	Sensory and motor impairment of the upper limb (Fugl-Meyer Assessment for the upper limb (FMUl) ; ADL (MAL); spasticity (MAS)	Both interventions showed significant improvements in FMUL and MAS, but not in MAL. Distal spasticity was significantly improved with tDCSc + AT compared with tDCS(a) + AT for right hemispheric lesions , but not for left hemispheric lesions. There was no effect of stimulation order on FMUI (ie. No significant difference between the two groups on FMUI). Both grous showed no significant improvement in ADL.	Combined therapy could achieve limited effects in the hemiplegic arm of chronic stroke patients.	4	
**30**	Kelley, et al, 2013	USA	**Sample size:** I=11 C=10<br><br>**Age Range:** NR<br><br>**Mean Age**:<br>I=66.91±8.50<br>C=64.33±10.91<br><br>**PSD** (years):<br>I=3.71<br>C=1.44<br><br>**TOS:** NR	Robot assisted body weight supported treadmill training	Over-ground gait training	PO: gait velocity (10meter walk test) and endurance (6 minutes walk test)<br>SO: changes in body structure and function (Fugl –Meyer Lower Extremity Motor Score (FM-LE), ADL (Functional Independence measure locomotion (FIM-L) and Barthel Index (BI)), Participation (Stroke Impact Scale (SIS))	Significant differences were found within the groups in the FM-LE score and BI between baseline and post intervention and between baseline and 3 months follow-up testing ($p < 0.05$). However, no other within group differences and no between<br>group differences were observed	Robot assisted body weight treadmill training may provide a better improvement in motor function and ADL than over-ground training	3	
**31**	Hseih et al., 2012	Taiwan	**Sample size:**<br>I: G1 = 18<br>G2=30= 18<br>C=18<br><br>**Age Range:** I=NR	G1 = high intensity robot-assisted therapy (high	Occupational therapy (CT)	PO: motor impairment (Fugl-Meyer Assessment Scale [FMA])<br>SO: Muscle power (Medical Research Council Scale), motor abilities (Motor	There is a significant group x time interaction effects (P = 0.01) on the FMA score. All 3 groups showed significant within-group gains on the FMA total score (P<0.05) and there is a significant	The 3 treatment groups in this trial had significant within group	3	

			C=NR<br><br>**Mean Age**:<br>I: G1= 56.51+10.03<br>G2 = 52.21+12.21<br>C=54.83 +9.84<br><br>**PSD** (months):<br>I: G1= 28.6+13.67<br>G2 = 23.28+15.37<br>C=22.44+15.34<br><br>**TOS:** **(**Ischemic/hemorrhagic/subarachnoid):<br>G1 = 12/6/0<br>G2 = 11/7/0<br>C = 9/8/1	intensity RT)<br><br>G2 = lower intensity robot assisted therapy (low intensity RT)		Activity Log [MAL]), function and QOL (Stroke Impact Scale [SIS])	differences among the 3 groups at mid-term (P = 0.002) and at post treatment (P = 0.002). The higher-intensity RT group had significantly greater improvements on the FMA total score than the lower-intensity RT and CT groups at midterm (*P*=0.003 and *P*=0.02) and at post treatment (*P*=0.04 and *P*=0.02; respectively. No significant differences were found between the lower intensity RT and CT groups at midterm and at post treatment. Within-group gains on the secondary outcomes were significant in all the groups, but the differences among the 3 groups did not reach significance	gains on the FMA, Medical Research Council, and Motor Activity Log, indicating that the patients benefited from the intervention in motor ability, muscle power, and self-perceived performance in daily activities.		
**32**	Kim, et a;l., 2012	USA	**Sample size:**<br>I=30<br>C=30<br><br>**Age Range:** NR<br><br>**Mean Age** (Years):<br>I= 67±10<br>C=66±11<br><br>**PSD** (Days):<br>I=10±1 1.7<br>C=10.9±2.3<br><br>**TOS:** Ischemic	G1 = bilateral robot-assisted rehabilitation<br><br>G2 = unilateral robotic assisted rehabilitation	Usual care	Upper limb function (Fugl-Meyer (FM) assessment**)**	Both patient groups of the intervention(G1 & G2), compared with the control group, showed a better average of four point improvement with the gains ranging from 10.7% to 31.3% for the bilateral group and 7.4%–26.3% improvement for the unilateral group. However, at 95% significance level, there is no significant difference between two training groups (P = 0.7937).	Robot assisted rehabilitation, irrespective of unilateral or bilateral, is more efficient in improving upper limb function than usual care	2	
**33**	Wu, et al.,	Taiwan	**Sample size:** I:<br>G1=14 G2 = 14<br>C=14	G1 = Occupational therapy	Occupation and physical	**PO:** motor impairment (The Upper Limb subscale of the Fugl-Mayer Assessment),	Large and significant effects were found in the kinematic variables, distal part of upper-limb motor	Compared with CT, TBAT and	2	

	2012		**Age Range:** NR<br><br>**Mean Age** (Years):<br>I: G1= 55.13 ±12.72<br>G2 = 57.04 ±8.78<br>C=51.30 ±6.23<br><br>**PSD** (months):<br>I: G1=18.00±8.65<br>G2 = 17.29 ±13.29<br>C=17.57 ±9.80<br><br>**TOS:**Ischemic/hemorrhagic: NR	with robot-assisted bilateral arm training(RBAT)<br><br>G2 = Occupational therapy with therapist-based bilateral arm training(TBAT)	therapy	daily functions (The Motor Activity Log [MAL]), quality of life (The Stroke Impact Scale [SIS] version3). Kinematics: temporal efficiency and smoothness, trunk motion, trunk compensation, shoulder flexion (Kinematic analysis)	impairment, and certain aspects of quality of life in favor of TBAT or RBAT. Specifically, the TBAT group demonstrated significantly better temporal efficiency and smoothness, straighter trunk motion, and less trunk compensation compared with the CT and RBAT groups. The RBAT group had increased shoulder flexion compared with the CT and TBAT groups. No group effect on the overall FMA score, proximal part score of the FMA, and AOU and QOM of the MAL was documented. However, on the FMA, the TBAT group showed higher distal part scores than the CT group. On the SIS, the RBAT group had better strength subscale, physical function domain, and total scores than the CT group.	RBAT exhibited differential effects on outcome measures. Therapist-based BAT may improve temporal efficiency, smoothness, trunk control, and motor impairment of the distal upper limb. Robotassisted BAT may improve shoulder flexion and quality of life		
**34**	Chang et al, 2012	Korea	**Sample size:** I = 20<br>C= 17<br><br>**Age Range:**<br>I= 27–6 C= 37-79<br><br>**Mean Age** (Years):<br>I = 55.5 ± 12.0<br>C= 59.7 ± 12.1<br><br>**PSD** : NR<br><br>**TOS (ischemic/hemorr**	Robot-assisted gait training: 40 minutes of gait training with Lokomat and 60 minutes of conventional physical therapy each day,	100 minutes of conventional physical therapy daily.	Aerobic capacity (peak VO2 and respiratory exchange ratio at peak exercise); Cardiovascular response (oxygen pulse, peak heart rate, systolic and diastolic blood pressure, and rate of perceived exertion at peak exercise). Ventilatory response (minute ventilation (*V*E) at peak exercise and ventilatory efficiency. Ventilatory efficiency). Motor and gait functional status (the lower extremity score of the Fugl-	Compared with the control group, the robot group showed 12.8% improvement in peak VO2 after training ($P < .05$). Compared with the control group, the robot group also improved in FMA-L score ($P < .05$).	Patients can be trained to increase their VO2 and lower-extremity strength using a robotic device for stepping during inpatient rehabilitation.	3	

			**hagic)**<br>c: I = 12/8<br>C= 11/6			Meyer assessment scale (FMA-L), the Motricity Index (MI-L), and the FAC)				
**35**	Abdullah et al, 2011	Canada	**Sample size:** I = 8<br>C = 11<br><br>**Age Range:**<br>I=65 – 86<br>C= 41 – 83<br><br>**Mean Age** (Years):<br>I = 75.7<br>C = 70.4<br><br>**PSD** (weeks):<br>I= 4.3<br>C= 4.3<br><br>**TOS**: I = NR<br>C = NR	Robotic arm therapy	Conventional therapy	Upper limb function (Chedoke Arm & Hand Activity Inventory (CAHAI-7));<br>Secondary: motor impairment of the arm (Chedoke McMaster Stroke Assessment (CMSA))	Both showed significant improvement in CAHAI-7. Furthermore, the degree of shoulder pain, as measured by the CMSA pain inventory scale, did not worsen for either group over the course of treatment.	Findings indicated that robotic arm therapy alone, without additional physical therapy interventions tailored to the paretic arm, was as effective as standard physiotherapy treatment for all responses and more effective than conventional treatment	3	
**36**	Conroy et al., 2011	Canada	**Sample size:** I: G1 = 20 G2 = 21 C = 21<br><br>**Age Range:** 3±2<br><br>**Mean Age** (Years):<br>I: G1 = 57 ± 12<br>G2 = 60 ± 13<br>C = 53 ± 47<br><br>**PSD** (years):<br>I: G1 = 3±2	G1 = Robot-Assisted Planar Reaching<br><br>G2 = Robot-assisted planar reaching (gravity compensat	Intensive conventional arm exercise program.	Upper extremity function (UE Fugl-Meyer Assessment (FMA)); Secondary: arm function (Wolf Motor Function Test (WMFT)) and stroke recovery (the Stroke Impact Scale (SIS), version 3)	For the FMA score, no significant within group nor between group difference was noted. The WMFT scores showed no significant differences (*P*=.58 for the group main effect) between the groups for speed of motor task performance.<br>However, the planar with vertical group had a larger change with significance in SIS ADLs scores compared with the intensive conventional arm exercise group.	Chronic upper extremity deficits because of stroke are responsive to intensive motor task training. However, training outside the	3	

			G2 = 5 + 8<br>C = 4 + 6<br><br>**TOS**: I: G1 = 16/4, G2 = 16/2<br>C = 19/0	ed), combined planar with vertical robot-assisted reaching,			No other significant within/between group difference was found in the secondary outcomes.	horizontal plane in a gravity present environment using a combination of vertical with planar robots was not superior to training with the planar robot alone		
**37**	Liao et al, 2012	Taiwan	**Sample size:** I = 10 C = 10<br><br>**Age Range:** NR<br><br>**Mean Age**:<br>I = 55.51+11.17<br>C = 54.56+8.20<br><br>**PSD** (days):<br>I= 23.90+13.39<br>C= 22.20+17.47<br><br>**TOS**: NR	Robot-assisted therapy	Dose-matched active control therapy	arm activity ratio of the accelerometer data (accelerometer); upper extremity function (Fugl-Meyer Assessment Scale); activity of daily living (the Functional Independence Measure (FIM)); motor activity (the Motor Activity Log); and bimanual ability (ABILHAND)	The robot-assisted therapy group significantly increased motor function, hemiplegic arm activity and bilateral arm coordination (Fugl-Meyer Assessment Scale: P=0.002; mean arm activity ratio: P=0.026; ABILHAND questionnaire: P=0.043) compared with the dose matched active control group	Symmetrical and bilateral robotic practice, combined with functional task training, can significantly improve motor function, arm activity, and self-perceived bilateral arm ability in patients late after stroke.	5	
**38**	Wagner et al., 201	USA	**Sample size:** I : G1= 49, G2 = 50<br>C = 28<br><br>**Age Range:** NR	G1 = usual care plus robot therapy<br>G2 = usual	usual care alone	Quality of life assessed using the Health Utilities Index, and the Stroke Impact Scale.	Changes in quality of life were modest and not statistically different, except for the Stroke Impact Scale, in which the robot group had a significantly higher	Usual care, complemented with robot therapy provides	2	

	1		**Mean Age**: NR<br><br>**PSD**: NR<br><br>**TOS**: NR	care plus intensive comparison therapy			score than the usual care group at 12 weeks (*P*=0.04). This difference decreased over time and was not significant at 24 or 36 weeks.	increased quality of life assessed with SIS in ost stroke patients.		
**39**	Burgar et al., (2011)	USA	**Sample size: I:**<br>G1= 19 G2 = 17<br>C=18<br><br>**Age Range:** NR<br><br>**Mean Age I:**<br>G1= 62.5 ± 2.0<br>G2 = 58.6 ± 2.3<br>C=68.1 ± 3.3<br><br>**PSD(days): I:**<br>G1= 17.3 ± 2.7<br>G2 = 16.6 ± 2.4<br>C=10.6 ± 1.2<br><br>**TOS:** I =NR<br>C = NR	G1 = 15 hours robot-assisted (RA) upper-limb therapy with the Mirror Image Movement Enabler (MIME)<br><br>G2 = 30 hours robot-assisted (RA) upper-limb therapy with the Mirror Image Movement Enabler (MIME)	15 hours of additional conventional therapy in addition to usual care	The primary outcome: upper extremity functions (Fugl-Meyer Assessment (FMA)). Secondary outcomes: ADL (Functional Independence Measure (FIM)), spasticity (Modified Ashworth Scale), and upper limb functions (Wolf Motor Test)	Gains in the primary outcome measure were not significantly different between groups at 6 months. The high-dose group had greater FIM gains than controls at discharge and greater tone but no significantdifference in FIM changes compared with low-dose subjects at 6 months. As used during acute rehabilitation, motor-control changes at follow-up were no less with MIME than with additional conventional therapy.	Improved shoulder and elbow function and impairment measures following RA training can be at least equivalent to conventional, labor-intensive methods.	2	
**40**	Morone et al., (2011)	Italy	**Sample size:**<br>**I:** RG_{HM} = 12<br>RG_{LM} = 12<br>C: CG_{HM} = 12<br>CG_{LM} = 12	robotic-assisted gait training with convention	Conventional training only.<br><br>subgroup:	The primary outcome measure was walking ability (as measured by Functional Ambulatory Category [FAC]).<br>secondary outcomes:	The lower motoricity subgroup of the intervention group significantly improved in FAC (*P*<.001), RMI (*P* =.001), and walking distance (*P* =.029). Conventional and robotic	Robotic therapy combined with conventional therapy may	3	

			**Age Range:** I= C=<br>**Mean Age**<br>RG_{HM} = 68.33+9.11<br>RG_{LM} = 55.58 +13.35<br>CG_{HM} = 62.92 +7.43<br>CG_{LM} = 60.17 +9.59<br><br>**PSD(days):**<br>RG_{HM} = 21.92+10.72<br>RG_{LM} = 16.25 +11.33<br>CG_{HM} = 20.00+15.68<br>CG_{LM} = 20.00+12.76<br><br>**TOS(ischemic/hemohragic):**<br>**I:** RG_{HM} = 9/3<br>RG_{LM} = 9/3<br>C: CG_{HM} = 12/0<br>CG_{LM} = 11/1	al training.<br><br>subgroup: high motor impairment (RG_{HM}) and low motor impairment (RG_{LM})	high motor impairment (CG_{HM}) and low motor impairment (CG_{LM})	spasticity (lower-leg Ashworth); mobility functions (Rivermead mobility index [RMI]); walking endurance (6-minute walk test on a 20-m path (6MWT), walking speed (10-m walk test (10MWT))	therapies were equivalent in the higher motricity arm. At the end of the 4 weeks of therapy, the mean FAC value was similar among RG_{LM}, RG_{HM}, and CG_{HM}, and lower for CG_{LM} . Significant difference was also see between the froup groups (P = .005), and post hoc analysis revealed differences only between the 2 $_{LM}$ subgroups (*P* = .002) favouring RG_{LM} but not between the 2 $_{HM}$ subgroups (*P* = .429). At discharge, FAC values were higher in the intervention groups than in the control (p .001). the RG_{HM} and CG_{HM} did not show any between group difference in any of the measured outcomes. While the MI and 10MWT did not differ between the RG_{LM} and CG_{LM}. The RG_{LM} showed higher improvement in 6MWT compared with CG_{LM}	be more effective in improving mobilitythan conventional therapy alone in patients with greater motor impairment during inpatient stroke rehabilitation.		
**41**	Albert et al., 2010	USA	**Sample size:**<br>**I: G1 = 49 G2 =** 50 C= 28<br><br>**Age Range:**<br>I: G1 =44-95<br>G2 = 228-86<br>C=2-88<br><br>**Mean Age:**<br>I: G1 =66 + 11	G1 = robot assisted therapy<br><br>G2 = intensive comparison therapy	Usual care	PO: motor functions (Fugl Meyer Assessment scale)<br>SO: motor control (Wolf Motor Function Tests[WMFT]), function and quality of life (Stroke Impact Scale [SIS]).<br>Others: adverse effect and measures of pain (numerical scale [0-10]), spasticity (Modified Ashworth Scale	At 12 weeks, A significant mean change in motor function and social participation of SIS was found between G1 and control (p = 0.009) and also between G1 and G2 in pain scale (p = 0.03) there is no other significant difference between the groups in other primary outcomes. At 36 weeks, G1 has a significantly more improvement in Fugl-Meyer	In patients with long term upper limb deficits after stroke, robot-assisted therapy did not significantly improve	3	

			G2 = 64 ± 11<br>C=63 ± 12<br><br>**PSD(days):**<br>I: G1 =3.6±4.0<br>G2 = 4.8 ± 4.0<br>C=6.2 ± 5.0<br><br>**TOS:** NR			[MAS])	scores and Wolf Motor Function Tests than control, however, there is no significant difference between G1 and G2	motor function at 12 weeks, as compared with usual care or intensive therapy. At 36 weeks, G1 improved outcomes as compared with usual care but not with G2		
**42**	Mirelman, et al 2010	Israel & USA	**Sample size:** NR<br><br>**Age Range:** NR<br>**Mean Age:** 62<br><br>**PSD:** NR<br><br>**TOS(ischemic/hemorrhagic):** NR	trained on a six-degree of freedom force-feedback robot interfaced with a virtual reality [VR] simulation	trained on a six-degree of freedom force-feedback robot interfaced without a virtual reality [VR] simulation	force, speed and excursion performances (the robotic system); Kinematic and kinetic gait parameters (an eight-camera motion capture system incorporating two embedded force platforms in a 7 m walkway)	Self-selected walking speed (SSWS) for the VR group improved significantly ($p = 0.003$) and the improvements were sustained at follow-up ($p = 0.013$) but no improvement was seen in the control group; both at post intervention and follow-up ($p = 0.97$); for Gait kinetics, Ankle kinetics were significantly different between groups favoring the intervention group: in ankle power at push-off ($p = 0.036$). For Gait kinematics, ankle ROM in the barefoot condition, increased significantly for both groups with the intervention group having higher increases. During ambulation with shoes, there were no significant differences between groups in kinematics and also neither group had differences in ankle ROM	There is potential for recovery of force and power of the lower extremity for individuals with chronic hemiparesis with training on a six-degree of freedom force-feedback robot interfaced with a virtual reality [VR] simulation	2	
**43**	Kutner,	Georg	**Sample size: I**=7<br>C=10	repetitive task	therapist-supervised	PO: Health related quality of life (Stroke Impact Scale	The mean SIS within the two groups did not show any	Though from this study, it	2	

	et al., 2010	ia	**Age Range:** I=NR C= NR<br>**Mean Age:**<br>**I=** 51.0 + 11.3<br>C= 61.9 + 13.4<br><br>**PSD(days):**<br>I= 184.1 + 126.5<br>C= 269.6 + 111.1<br><br>**TOS(ischemic/hemorrhagic):** I = 5/2 C = 7/3	practice (RTP) combined with 30 hours of robotic-assisted therapy	repetitive task practice (RTP)	(SIS) version 3.0),depression (20-item Center for Epidemiologic Studies–Depression [CES-D] Scale)	significant difference from baseline to post-intervention but showed a significant difference from baseline to follow-up (0.008). The average change in mood ratings in the combined therapy group was greater from pre-intervention to post-intervention (*P*=.03). There is an improvement in mood in the control group from baseline to post intervention (p = 0.02). There is also an improvement in both groups in ADL domain of SIS from baseline to post intervention (p = 0.04) and there is an improvement in both groups in hand function from baseline to post intervention and through the follow-up (p <0.05)	can't be considered a better therapy, Robotic-assisted therapy may be an effective alternative or adjunct to the delivery of intensive task practice interventions to enhance mood and ADL in patients with stroke.		
**44**	Schwartz et al., 2009	Israel	**Sample size:** I=37 C=30<br><br>**Age Range**: I=NR C= NR<br><br>Mean Age**:**<br>I=62 + 8.5<br>C=65 + 7.5<br><br>**PSD(days):**<br>I=21.6 + 8.7<br>C=23.6 + 10.1<br><br>**TOS**:<br>I = 23/14 C = 26/4	robotic-assisted gait training (RAGT)	regular physiotherapy	Primary: ability to walk independently (FAC scale)<br><br>Secondary: neurological status (NIHSS); ADL (FIM); Functional motor assessment (disability stroke activity scale (SAS) for stroke patients). Gait velocity (10-MWT test); basic mobility and transfer (TUG test); exercise tolerance (2 minutes walk test)	RAGT group showed a significant improvement in FAC. Both groups also showed significant improvement in the SAS score at the end of the 6-week treatment without significant difference between groups (*P*=0.22). Both groups showed significant improvement in NIHSS, with the RAGT group showing significantly lower NIHSS score than the control group (*P* < .01); both groups also showed significant improvement in the FIM cognitive subscore, with no significant differences between groups (*P* = .73); however, the motor FIM score showed a significant advantage in the	at the end of a 6-week trial, that locomotor therapy with the use of RAGT combined with regular physiotherapy produced promising effects on functional and motor outcomes in patients after subacute stroke as compared	3	

							RAGT group as compared with the control group (*P* = .05). no significant difference in mean 10MWT and TUG was also noted between the groups	with regular physiotherapy alone.		
**45**	Hidler et al., 2009	USA	**Sample size:** I=33 C=30<br><br>**Age Range:**<br>I=30-79<br>C= 36-78<br><br>**Mean Age:**<br>I=59.9 +11.3<br>C=54.6 +9.4<br><br>**PSD(days):**<br>I=28.9 + 12<br>C=26.1 + 10.9<br><br>**TOS(ischemic/hemorrhagic):**<br>I = 26/7<br>C = 21/9	robotic-assisted gait training with the Lokomat	conventional gait training	Primary outcomes: walking velocity (5 meters walk test) and walking distance (6-minute walk test)<br>Secondary outcomes: balance (Berg Balance Test), mobility functions (Functional Ambulation Category (FAC)), stroke severity (National Institutes of Neurological Disorders and Stroke, National Institutes of Health, US NIH Stroke Scale), motor impairment (Motor Assessment Scale), performance (Rivermead Mobility Index), perception of instrumental ADL participation (Frenchay Activities Index), and stroke impact (SF-36 Health Survey).<br>cadence (Gait Rite)	Control group experienced significantly greater gains in walking speed (P = .002) and distance (P = .03) than the intervention group. These differences were maintained at the 3-month follow-up evaluation. Secondary measures were not different between the 2 groups, although a 2-fold greater improvement in cadence was observed in the conventional versus Lokomat group.	For subacute stroke participants with moderate to severe gait impairments, the conventional gait training interventions appears to be more effective than robotic-assisted gait training for facilitating returns in walking ability.	1	
**46**	Hornby et al., 2008	USA	**Sample size:** I= 24 C= 24<br><br>**Age Range:** NR<br><br>**Mean Age:**<br>I=57+10<br>C= 57+11<br>**PSD(months):**<br>I=50+51<br>C= 73+87	Robot assisted locomotor training	Therapist assisted locomotor training	gait speed (instrumented walkway); walking capacity (6 minute walk test); functional ambulation (modified Emory Functional Ambulation Profile (mEFAP)); balance (Berg Balance Scale); perception of instrumental ADL participation (Frenchay Activities Index); heath	The control group showed greater improvements in speed and single limb stance time on the impaired leg, with larger speed improvements in those with less severe gait deficits. Perceived rating of the effects of physical limitations on quality of life improved only in subjects with severe gait deficits who received therapist-assisted LT.	Therapist-assisted lomotor training facilitates greater improvements in walking ability in stroke survivors as	1	

			**TOS(ischemic/hemorrhagic):**<br>I = 12/12<br>C =10/14			components (Short Form 36 (physical SF36)		compared to a similar dosage of robotic-assisted locomotor training.		
**47**	Volpe et al., 2008	USA / UK	**Sample size:** I= 11 C= 10<br><br>**Age Range:** NR<br><br>**Mean Age:**<br>I= 62 ± 3<br>C= 60 ± 3<br><br>**PSD(months):**<br>I= 35 ± 7<br>C= 40 ± 11<br><br>**TOS(ischemic/hemorrhagic):**<br>I = 10/1<br>C = 10/0	Robot assisted intensive movement-based rehabilitation	Therapist assisted intensive movement-based rehabilitation	Primary: Elbow/shoulder function (Fugl-Meyer Scale for Shoulder/Elbow (FM Sh/El);<br>Secondary: Wrist/Hand function (Fugl-Meyer Scale for Wrist/Hand (FM W/H); motor ability (Motor Power Scale for Shoulder/Elbow (MP)); resistance to passive movement (Modified Ashworth Scale); Disability (Stroke Impact Scale (SIS)); motor impairment (Action Research Arm Test (ARAT)); pain (pain scale from the Fugl-Meyer scale); depression (Beck Depression Scale); stroke severity (NIH Stroke Scale)	Both groups showed significant change over time for the primary outcome measures, shoulder (*P* < .007) and elbow(*P* < .003),<br>& was maintained in a 3-month follow-up. There was no change in the FM W/H measure over time. There was no significant difference in mean of all the outcome measures found between the groups. There were no significant improvements in disability outcome measures (Ashworth Scale, the Joint Stability Scale, pain assessment, and Beck Depression Scale) or differences across groups.	Robot assisted intensive movement-based rehabilitation did not demonstrate specific efficacy over Therapist assisted intensive movement-based rehabilitation in improving motor functions. Both interventions did not show any therapeutic effect on disability outcomes	1	
**48**	Lum, 2006	USA	**Sample size:**<br>I: G = 9<br>G = 5<br>G = 10<br>C= 6	G1 = Robot unilateral group: performed exercise	Conventional rehabilitation (neurodevelopmental	Spasticity (MAS); ADL (FIM); motor impairment (upper limb section of Fugl-Meyer [FM] Scale and Motor Status Score [MSS); amount of time spend performing	There was a significant difference in the amount of time spend performing movement in the robot between the intervention groups (p <0.05). When compared with the control group, the robot	Both robot-combined and robot-unilateral training provide	1	

			**Age Range:** NR<br><br>**Mean Age:**<br>I: G = 69.8 + 4.0<br>G = 72.2 ± 11.7<br>G = 62.3 ± 2.8<br>C= 59.9 ± 5.5<br><br>**PSD(weeks):**<br>I: G = 10.0 ± 1.9<br>G = 6.2± 1.0<br>G = 13.0 ± 2.1<br>C= 10.6 ± 2.7<br><br>**TOS(ischemic/ hemorrhagic):**NR	that progressed from the easiest exercise mode (psive) to the most challenging (constraint induced)<br><br>G2 = robot-bilateral group: practiced the same 12 reaching movements but only in bilateral mode<br><br>G3 = Robot-combined group: spent approximately half of the treatment time in the unilateral mode and the other half in	therapy [NDT])	movement in the robot (mirror image movement enabler [MIME] system); muscle strength (motor power examination)	combined group has higher significant improvement in the proximal FM and MSS synergy scale ($p < 0.05$), however, both of the differences were lost at 6 months follow up. Significant improvements were noted in both robot unilateral and robot combined group in both proximal and distal FM, motor power examination and FIM ($p < 0.05$).	better effective improvement in functional ability and motor functions compared to conventional rehabilitation. These effects appears to be temporary		

				bilateral mode.						
**49**	Fasoli, et al 2004	USA	**Sample size:** I= 30 C= 26<br><br>**Age Range:** NR<br><br>**Mean Age:**<br>I= 62 ± 2.4<br>C= 67 ± 2.3<br><br>**PSD(days):**<br>I= 14 ± 0.9<br>C= 16 ± 1.3<br><br>**TOS(hemorrhagic /non-hemorrhagic):**<br>I= 4/26<br>C= 3/23	Robot therapy : participated in at least 25 sessions of sensorimotor robotic training for the paretic arm assisted by the robot	robot exposure therapy: performed the same planar reaching tasks as the robot therapy group but assisted by the patient using the non paretic arm or the therapist	upper extremity function (Fugl-Meyer test); upper-limb isolated movement (Motor Status Score); motor power (Medical Research Council Scale [MRC]) and functional motor abilities (FIM)	There were statistically significant group by time interactions for the Fugl-Meyer test, Motor Status Score for shoulder and elbow, and the MRC test of motor power. No significant group by time interactions was found on the Motor Status Score for wrist and hand. Both groups showed significant improvement on the Fugl-Meyer Test. Control group also showed Statistically significant improvements on the Motor Status Score for shoulder and elbow and on the MRC test of motor power, but this improvement only lasted for a short time. Patients in both groups made significant improvements in FIM scores from admission to discharge (*P* < 0.0001).	Robot therapy provided better improvement in motor functions	2	
**50**	Fasoli, et al, 2003	USA	**Sample size:** NR<br><br>**Age Range:** NR<br><br>**Mean Age:** NR<br><br>**PSD:** NR<br>**TOS(ischemic/hemorrhagic):**NR	sensorimotor robotic therapy: the robot provided movement assistance when the person was unable to reach the targets independently	progressive-resistive robotic therapy: the robot provided movement resistance to targeted motions	Spasticity Modified Ashworth Scale (MAS); synergistic and isolated movement patterns and grasp (Fugl-Meyer test of upper-extremity function); muscle strength (MRC test of motor power); complete and discrete measure of upper-limb isolated movement and motor function (The Motor Status Scale (MSS) score)	A statistically significant between group difference was found for the MSS wrist and hand score (*P*=.006). Subjects who received progressive-resistive therapy showed substantial improvements in wrist and hand movement that were not seen in the sensorimotor group. No significant group differences were found for the other clinical evaluation scores	Both Robotic therapy shows reduction in motor impairment in persons with moderate to severe chronic impairments	1	

Transcranial Direct Current Stimulation (t-DCS)

Ref	Study (Authors, year	Country	Participants	Interventions/ Model of care	Control/ comparison	Outcomes assessed/ outcome measures	Results	Conclusion	Quality score
**51**	Klomjai,et al. (2018)	Thailand	**19 stroke patients**<br><br>**Age range of all patients: 20–74 years**<br><br>**PSD= less than 6 months**<br><br>**TOS= ischaemic stroke**	Transcranial direct current stimulation	Conventional physical therapy	Lower-limb performance was assessed by the Timed Up and Go (TUG) and Five-Times-Sit-To-Stand (FTSTS) tests and muscle strength was assessed by peak knee torque of extension.	For TUG, there was a significant difference between PRE (24.0 [4.7 s] and POST (18.5 [3.0 s],P = 0.004 for only the real group, with no difference for the sham group (P > 0.005). For FTSTS, paired t-test revealed a significant difference between PRE (18.3 [1.2 s] and POST (15.1 [1.2 s], for only the real group (P = 0.003), with no difference for the sham group (P > 0.005). no significant difference between the two groups.	A single session of dual-tDCS before PT in people with sub-acute stroke immediately improved lower-limb function but not strength.	2
**52**	Manji et al,. (2018)	Japan	Thirty stroke inpatients<br>**Age** :(years), mean (SD)<br>E=62.2 (10.1)<br>C= 63.7(11.0)<br>**PSD**:mean (SD)<br>E=134.5 (55.7days)<br>C=149.7 (24.2)days<br>**TOS:** (ischemic/ hemorrhagic)<br>E=9/6<br>C= 8/7	Combined therapy body weight-supported treadmill training (BWSTT) and tDCS	Sham stimulation	-10 m Walk Test (10MWT)<br>-Timed Up and Go (TUG) test<br>-Fugl-Meyer Assessment, lower limb,<br>-Trunk Control Test (TCT):,<br>-Performance Oriented Mobility Assessment (POMA):<br>- Functional Independence Measure(FIM)<br>*[uses not supplied]*	tDCS with BWSTT significantly improved gait speed (10MWT) and applicative walking ability (TUG), compared with BWSTT + sham stimulation periods (p < 0.05)	The facilitative effects of tDCS on supplementary motor area improved postural control during BWSTT.	2

**53**	Oveisgharan et al,. 2017	Iran	20 patients<br>**Age** range:<br>Control= 65.3 ± 16.5yrs<br>Experimental= 52.1 ± 12.8yrs<br><br>**PSD**= N/A<br><br>**TOS**: ischaemic	Transcranial direct current stimulation (tDCS)	Sham stimulation	Fugl-Meyer upper extremity assessment (FM) and Action Research Arm Test (ARAT.<br><br>*[Uses were not stated]*.	During the first days of stimulations, the sham group's FM scores increased by 1.2 per day, while the real group's scores increased by 1.7 per day (p = .003).	left dorsolateral prefrontal cortex (DLPFC) stimulation in conjunction with M1 stimulation resulted in better motor recovery than real motor cortex (M1) stimulation alone	1
**54**	Fan et al, 2017	Canada	30 right-handed subjects<br><br>mean age: 22.1 ± 3.3 years<br><br>PSD: N/A<br><br>TOS: haemorrhagic or ischaemic N/S	Transcranial direct current stimulation (tDCS) applied over the primary motor cortex (M1)	Sham stimulation	Metronome-assisted sequential visual isometric pinch task (SVIPT) [N/S]	The anodal tDCS group showed significantly greater improvement in performance (39.28 15.92%) than the sham tDCS group (24.06 16.35%) on the metronome-assisted task, t(28) = 2.583, P = 0.015 (effect size d = 0.94)	Anodal tDCS is effective in promoting grip motor learning in both post stroke patients and healthy individuals.	2
**55**	Koh et al 2017	Taiwan	25 participants<br>**Age** range:<br>(tDCS-SM)= 55.3±11.5 (n=14)<br>Control= 13 56.9±13.5 (n=11)<br>**PSD:**<br>Intervention group= 15.8 (8.1) months<br>Control group= 13.4 (9.4)months<br>**TOS:** Both ischaemic and haemorrhagic conmbined.<br>Intervention group= ischaemic 10, haemorrhagic 4<br>Control group= ischaemic 8,	Transcranial direct current stimulation with sensory modulation (tDCS-SM)	High repetitions of passive movements on the paretic hand	The Action Research Arm Test (ARAT) was used to assess UE function.<br><br>The Barthel Index (BI) assesses an individual's level of independence in basic activities of daily living.	Compared with the control group, the tDCS-SM group had a trend of a small immediate effect ($\eta p2$ = 0.02 – 0.04) on reducing spasticity but no long-term effect. A trend of small immediate and long-term effects in favor of tDCS-SM was found on UE function and daily function recovery ($\eta p2$= 0.02 - 0.09)	tDCS-SM had a nonsignificant trend of having immediate and longitudinal effects on voluntary UE movement recovery in patients with severe to moderate UE paresis after stroke,	4

			haemorrhagic 3						
56	Ilić et al,. 2016	Serbia	26 stroke patients<br>**Age** range(years), mean (SD):<br>Experimental group=58.3 (7.7)<br>Control group=62.0 (3.9)<br>PSD (months):<br>Experimental group= 41.0 (24.4)<br>Control group= 37.3 (20.9) 0.68<br>TOS: ischaemic	Anodal transcranial direct stimulation (tDCS) with occupational therapy	Sham stimulation with occupational therapy	Modified Jebsen Taylor Hand Function Test (mJTHFT) was used for functional assessment that is scored by using the summed times required to complete seven individual tasks.<br><br>Upper limb Fugl-Meyer (ULFM) [N/S]	mJTHFT showed significant differences between active and sham group i.e. main effect of group F[1,23] = 18.978, p < 0.001, p2 = 0.452,as well as the effect of covariate F[1,23] = 88.191, p < 0.001, p2 = 0.793; and no within-subject effect of time F[2,46] = 0.709, p = 0.497, p2 = 0.030 i.e. between-group differences were preserved across time without change of slope	Fine motor skill deficits in chronic stroke survivors can be improved when intensive OT is primed with anodal tDCS over the ipsilesional hemisphere.	4
16	Straudi et al, (2016)	Italy	**Age** (years)<br>Mean (SD):<br>Experimental group= 52.7 (16.0)<br>Control group=64.3 (9.7)<br>**PSD** (SD)days:<br>Experimental group= 40.7 (35.1) Control group=78.2 (61.9)<br>**TOS** (ischaemic/ haemorrhagic)<br>Experimental group=10/2<br>Control group=9/2	tDCS combined with upper extremity robot-assisted therapy (RAT)	RAT + sham-tDCS.	-Fugl-Meyer Assessment-Upper Extremity (FMA-UE) to assess arm motor recovery<br>-Box and Block Test (BBT) was used to evaluate gross motor function-Motor Activity Log (MAL) was administered to quantify real-world arm use in activities of daily living	recovery stage in sham-tDCS group (= 9.20, df = 1,9; <0.05; adjusted span style="font-family: MinionMath-Capt;font-size:7pt;color:rgb(35,31,32);font-style:normal;font-variant:normal;"R2 = 0.45) and stroke location in real-tDCS group (= 8.48, df = 1,10; < 0.05; adjusted span style="font-family: MinionMath-Capt;font-size:7pt;color:rgb(35,31,32);font-style:normal;font-variant:normal;R2 = 0.40)	The additional use of bilateral tDCS to RAT seems to have a significant beneficial effect depending on the duration and type of stroke.	3
57	Allman et al,. (2016)	UK	24 patients<br>**Age** range, years:<br>Experimental group= 59.5(12.1)<br>Control group=66.8 (10.4)<br>**PSD:**	Anodal transcranial direct current stimulation (tDCS)	Sham treatment	Action Research Arm Test (ARAT)<br><br>Wolf Motor Function Test (WMFT)	There were higher scores for the anodal tDCS group compared to the sham group for both ARAT [c2(8) = 28.88, P = 0.031, corrected for the three measures considered] and WMFT [c2(8) = 27.91, corrected P = 0.037] but not	The addition of ipsilesional anodal tDCS to a 9-day motor training program improved long-term clinical outcomes relative to	2

			Experimental group= 51.2 (33.4)<br>Control group= 56.6 (39.8)<br>**TOS:** both ischaemic and haemorrhagic [N/S]			Upper Extremity Fugl-Meyer (UEFM) score.<br><br>[uses were not stated]	for UEFM [c2(8) = 14.22, corrected P = 0.329].	sham treatment in patients after stroke	
**58**	Au-Yeung, 2014	China	**Sample size:** NR<br><br>**Age Range:** NR<br><br>**Mean Age:** NR<br><br>**PSD(years):** NR<br><br>**TOS(ischemic/hemorrhagic):** NR	-G1 = anodal transcranial direct current stimulation (a-tDCS) targeting the primary motor area of the lesioned hemisphere (M1 lesioned)<br>-G2 = cathodal transcranial direct current stimulation (c-tDCS) applied to the contralateral hemisphere (M1-nonlesioned)	Sham transcranial direct current stimulation (tDCS)<br><br>NB: Randomized crossover trial with a washout period of at least 5 days between sessions	Primary: hand dexterity (The Purdue pegboard); selective attention (A color-word Stroop test (Stroop) levels 1, 2, and 3); Pinch grip strength (dynamometer)	There was a significant improvement in hand dexterity after c-tDCS (P = 0.014), but after a-tDCS or sham stimulation, there was no significant improvement. However, there was no significant difference in hand dexterity across the three interventions. The Stroop test levels 1 and 2 did not show any significant change with the three tDCS interventions. On level 3, however, response time was significantly shortened by 0.6 secs after c-tDCS (P = 0.017) only. The improvement in Stroop level 3 was significantly different across the three tDCS sessions (P = 0.041). Pinch strength was not changed significantly with any of the three tDCS conditions	Twenty minutes of cathodal tDCS to M1nonlesioned can promote both paretic hand dexterity and selective attention in people with chronic stroke	3
**29**	Ochi, et al, 2013	Japan	**Sample size:** NR<br><br>**Age Range:** NR<br><br>**Mean Age:** NR<br><br>**PSD:**<br>NR<br>**TOS: ischemic/hemorrhagic :** NR	Anodal transcranial direct current stimulation (tDCS) with robot-assisted arm training (AT) (tDCS(a) + AT)<br><br>NB: Randomized crossover trial	Cathodal transcranial direct current stimulation (tDCS) with robot-assisted arm training	Sensory and motor impairment of the upper limb (Fugl-Meyer Assessment for the upper limb (FMUl) ; ADL (MAL); spasticity (MAS)	Both interventions showed significant improvements in FMUL and MAS, but not in MAL. Distal spasticity was significantly improved with tDCSc + AT compared with tDCS(a) + AT for right hemispheric lesions , but not for left hemispheric lesions. There was no effect of stimulation order on FMUI (ie. No significant difference between the two groups on FMUI). Both grous showed no significant	Combined therapy could achieve limited effects in the hemiplegic arm of chronic stroke patients.	4

					(tDCS(c) + AT)		improvement in ADL.		
59	Wu, et al., 2013	China	**Sample size:** I=45 C=45<br><br>**Age Range:** NR<br><br>**Mean Age**: I= 45.9±11.2<br>C = 49.3±12.6<br><br>**PSD** (months):<br>I=4.9±3.0<br>C = 4.9±2.9<br><br>**TOS: (**Ischemic/ hemorrhagic):<br>I = 27/18<br>C = 26/10	Transcranial direct current stimulation (tDCS) to the primary sensorimotor cortex of the affected side with cathodal stimulation,<br>and conventional physical therapy	Sham stimulation (same area as the tDCS group) and conventional physical therapy.	Muscle tone (MAS), ADL (BI); Motor function (UL motor component of the Fugl-Meyer Assessment)	Both groups significantly improved in all the measured outcomes at post intervention and at 4 weeks follow; however, for MAS score, the control group only showed a significant improvement at follow-up. Compared with the sham tDCS group, UL muscle tone was significantly decreased and UL motor function and ADL assessment significantly improved in the active tDCS group after tDCS and at follow-up.	Cathodal tDCS combined with conventional physical therapy decreases muscle tone and improves motor function and ADL significantly more than conventional physical therapy alone. But both therapies provide improvement in muscle tone, upper limb function and ADL	4
60	Zimerman, et al, 2012	USA	**Sample size:** NR<br><br>**Age Range:** NR<br><br>**Mean Age** (Years): NR<br><br>**PSD**: NR<br><br>**TOS:** NR	Cathodal transcranial direct current stimulation (tDCS). (Cross over design)	Sham transcranial direct current stimulation (tDCS)	Motor performance recorded with an ergonomic 4-button electronic keyboard connected to a computer	tDCS facilitated the acquisition of a new motor skill compared with sham stimulation (*P*=0.04) yielding better task retention results.	These results indicate that tDCS is a promising tool to improve not only motor behavior, but also procedural learning.	3
61	Nair, et al, 2011	USA	**Sample size:**<br>I = 7<br>C = 7<br>**Age Range:** I=NR C=NR<br><br>**Mean Age** (months):<br>I = 61±12<br>C = 56±15	Cathodal transcranial Direct Current Stimulation (tDCS) and simultaneous occupational therapy	Sham tDCS+OT.<br>and simultaneous occupational therapy	Range-Of-Motion (ROM)<br>Upper limb function (Upper-Extremity Fugl-Meyer Assessment (UE-FMA)), and therapeutic response (functional	Cathodal tDCS+OT resulted in significantly more improvement in ROM in multiple joints of the paretic UE and in the UE-FMA scores than sham tDCS+OT, and that the effects lasted at least one week post-stimulation.<br>-The magnitude of the activation (beta values) in the contralesional motor region	This suggests that cathodal tDCS combined with OT leads to significant motor improvement after stroke	3

			**PSD** (days):<br>**I =** 33±20<br>C = 28±28<br><br>**TOS**: NR			magnetic resonance imaging [FMRI])	decreased in the majority of patients (5 out of 7) treated with cathodal tDCS to the contralesional motor region, while only 3 out of 6 patients in the sham group showed slight decreases.		
**62**	Bolognini et al, 2011	Italy, Malaysia, USa	**Sample size:** I = 7 C = 7<br><br>**Age Range:** NR<br><br>**Mean Age**: NR<br><br>**PSD**: NR<br><br>**TOS**: NR	Active bihemispheric transcranial direct current stimulation (tDCS) combined with constraint-induced movement therapy (CIMT).	Sham bihemispheric transcranial direct current stimulation (tDCS) combined with constraint-induced movement therapy (CIMT).	-Hand function (JHFT); -Handgrip strength (Handgrip Strength (HS);<br>-ADL (MAL);<br>- Selective movement (UE-FMA);<br>-Cortical excitability (transmagnetic stimulation)	-Patients in both groups demonstrated significant gains on all primary outcome measures,(JHFT, HS, MAL, and UE-FMA). Gains were larger in the active tDCS group.<br>-Neurophysiological measurements showed a reduction in transcallosal inhibition from the intact to the affected hemisphere and increased corticospinal excitability in the affected hemisphere only in the active tDCS/CIMT group. Both groups showed a reduction in corticospinal excitability of the unaffected hemisphere.	CIMT alone appears effective in modulating local excitability but not in removing the imbalance in transcallosal inhibition.<br><br>Bihemispheric tDCS may achieve this goal and foster greater functional recovery.	4
**63**	You et al. 2011	Korea	**Sample size:** I: G1= 14 G2 = 7 C= 21<br><br>**Age Range:** NR<br><br>**Mean Age:**<br>I: G1 = 65.86<br>G2 = 70.43<br>C= 63.43<br><br>**PSD:**<br>I= NR<br><br>**TOS:** NR	-G1 = cathodal transcranial direct current stimulation (tDCS) applied to the right superior temporal gyrus, both with conventional speech and language therapy<br>-G2 = anodal transcranial direct current stimulation (tDCS) applied to the left superior temporal gyrus	Sham transcranial direct current stimulation (tDCS) with conventional speech and language therapy	Korean-Western Aphasia Battery (K-WAB) for assessment of aphasia	All patients had significant improvements in aphasia quotients, spontaneous speech, and auditory verbal comprehension. However, auditory verbal comprehension improved significantly more in patients treated with a cathode, as compared to patients in the other groups.	These results are consistent with the role of Wernicke's area in language comprehension and the therapeutic effect that cathodal tDCS has on aphasia patients with subacute stroke, suggesting that tDCS may be an adjuvant treatment approach for aphasia rehabilitation therapy in patients in an early stage of	4

								stroke.	
**64**	Lindenberg, et al, (2010)	USA	**Sample size: I=** 10 C= 10<br><br>**Age Range:** I= NR C=NR<br><br>**Mean Age:** **I=** 61.7 + 14.7 C= 55.8 + 12.9<br><br>**PSD(months):** NR<br><br>**TOS(ischemic/hemorrhagic):** I = NR C = NR	Real bihemispheric transcranial direct current stimulation (tDCS) with simultaneous physical/occupational therapy	Sham stimulation with simultaneous physical/occupational therapy.	Motor impairment (Fugl-Meyer-UE); motor function (Wolf Motor Function Test); cortical excitability (functional imaging)	The improvement of motor function was significantly greater in the real stimulation group when compared to the sham group. The effects outlasted the stimulation by at least 1 week. In the real-stimulation group, stronger activation of intact ipsilesional motor regions during paced movements of the affected limb were found postintervention whereas no significant activation changes were seen in the control group.	The combination of bihemispheric tDCS and peripheral sensorimotor activities improved motor functions in chronic stroke patients that outlasted the intervention period. This novel approach may potentiate cerebral adaptive processes that facilitate motor recovery after stroke.	3
**65**	Kim et al., (2010)	Korea	**Sample size:** I: G1= 6 G2 = 5 C= 7<br>**Age Range:** NR<br>**Mean Age:** I : G1= 55.3 + 16.4 G2 = 53.6 + 14.9 C= 62.9 +9.2<br>**PSD(days):** I: G1 = 34.0 +27.1 G2 = 19.4 +9.3 C= 22.9+7.5<br>**TOS(ischemic/hemorrhagic):** NR	G1: anodal transcranial direct current stimulation over the affected motor cortex,<br>G2: cathodal transcranial direct current stimulation over the unaffected motor cortex,.	C: sham stimulation	upper limb motor impairment with FMA score and global functional state with mBI	G2 led to a greater improvement in FMA than C (P < 0.05). The mBI score indicated that time (*P* <0.001) and the intervention x time interaction (*P* < 0.010) were significant, but that intervention alone was not significant. Post hoc analysis indicated no significant differences in MBI scores among the three groups (*P* > 0.05) at the 6-month follow-up	There is a potentially beneficial effect of noninvasive cortical stimulation during rehabilitative motor training of patients who have suffered from subacute strokes.	3
**66**	Figlewski et al. (2017)	Denmark	44 patients<br>**Mean age**, (yrs): E=60±11; C= 61±10<br>**PSD** median (range) months: E=9 (3–35); C=7 (3–36)<br>**TOS** (ischaemic /haemorrhagic)	Constraint-induced movement therapy with anodal transcranial direct current stimulation (CIMT+tDCS)	CIMT + sham tDCS	Wolf Motor Function Test used for function and strength Functional Ability Scale (WMFT-FAS; range, 0–75), was used to assess each of the 15 tasks	After adjustment for WMFT-FAS baseline scores, age, sex, and dominant hand, the between-group difference was still signifcant (coeffiient of β=2.8; 95% confience interval, 0.05–5.5;P=0.046). Coeffiients for the other variables in the model were not signifiant: WMFT-FAS baseline score (β=0.09;	CIMT+TDCS resulted in improvement of functional ability of the paretic upper limb compared with CIMT alone.	**4**

			E= 19/3; C= 18/4			on a 6-point scale, ranging from 0	P=0.2), age (β=0.1; P=0.15), sex (β=−0.72; P=0.6), and dominant hand (β=1.4; P=0.3).		

Transcranial Magnetic Stimulation (TMS)

Ref	**Study (Authors, year**	**Country**	**Participants**	**Interventions/ Model of care**	**Control/ comparison**	**Outcomes assessed/ outcome measures**	**Results**	**Conclusion**	**Quality score**
**67**	Hu et al, (2018)	USA	Forty post-stroke **Age range** (Yrs): E1 =46.5 ± 12.1 E2 =48.5 ± 11.2 E3= 50.7 ± 10.4 C= 47.3 ± 9.8 **PSD:** >1month **TOS**[I/H] E1 =5/5, E2 =6/4, E3 = 5/5, C=4/6	E1: High-frequency rTMS (HF-rTMS) group (10 Hz) E2: Low-frequency rTMS (LF-rTMS) group (1 Hz) E3: A sham stimulation group	C: Standard treatment	Chinese version of the Western Aphasia Battery (WAB) was applied to assess the language ability of patients.	The LF-rTMS group exhibited a more marked improvement than the HF-rTMS group in spontaneous speech, auditory comprehension, and aphasia quotients (AQ). Compared to the control group, the HF-rTMS cohort exhibited significant improvement at 2-months post-treatment in repetition and AQ.	LF-rTMS and HF-rTMS are both beneficial to the recovery of linguistic function in patients with post-stroke non-fluent aphasia.	3
**68**	Long, et al, 2018	China	Sixty-two patients **Age** range (years): HF-rTMS= 55.90 ± 8.89;LF-rTMS= 57 ± 11.78; Control= 56.85 ± 5.48 **PSD** (days) HF-rTMS= 19.05	Combining high-frequency repetitive transcranial magnetic stimulation (HF-rTMS) and low-frequency rTMS (LF-rTMS)	Unilateral use of LF-rTMS alone.	The upper limb motor function was evaluated using the Fugl-Meyer Assessment (FMA) and the Wolf Motor Function Test (WMFT)	LF-rTMS and LF-HF rTMS groups exhibited significant improvements in terms of both the FMA scores (P = 0.018 in the LFrTMS group and P = 0.001 in the LF-HF rTMS group) and the WMFT time (both P < 0.001) relative to the LF-rTMS group.	LF- and LF-HF rTMS were effective in promoting upper limb motor recovery in patients with acute stroke	1

			± 2.74;LF-rTMS= 19.57 ± 2.34;<br>Control=19.81 ± 2.98<br>**TOS**(ischaemic/haemorrhagic)<br>HF-rTMS= 10/10<br>LF-rTMS= 11/10<br>Control=11/10						
**69**	Kim and Yim (2018)	Korea	Twenty stroke patients<br>**Age** range:<br>Experimental group=51+/-2.98<br>Control group= 74.11+/-2.88<br>**PSD**= <3 months<br>**TOS** (ischaemic /haemorrhagic)<br>Experimental=5/3<br>Control=7/5	high-frequency repetitive transcranial magnetic stimulation (HF-rTMS) combined with task-oriented mirror therapy (TOMT)	HF-rTMS	-Corticospinal and motor capacity recovery(Motor evoked potential MEP) -Pinch grip and pich grip tests (JAMAR® Hand Evaluation Kit a hydraulic dynamometer and a hydraulic pinch gauge)<br>- Hand motor function (Box and block test)	Significant improvements in the MEP and hand function variables were observed in both groups (p<0.05). Inparticular, hand functions (pinch grip and box and block test) were significantly different between the 2 groups<br>(p<0.05).	HF-rTMS combined with TOMT had a positive effect on hand function and can be used for the rehabilitation of precise hand movements in acute stroke patients.	1
**70**	Chervyak et al, 2018	U.S.A	42 patients<br>**Age** range:<br>Sham=61.4 +/-11.4<br>LF=54.2 6+/-11.1<br>HF=58.6 +/- 10.4<br>H-LF=60.7 6+/-9.6<br>**PSD**(months)<br>Sham= 7.9 ± 8.4<br>LF= 5.1 ± 4.8<br>HF= 5.8 ± 4.6<br>H-LF= 7.37 ± 5.9<br>**TOS**=both ischaemic and haemorrhagic	Repetitive transcranial magnetic stimulation (rTMS)<br>-Low-Frequency (LF)<br>-High-Frequency (HF)<br>-High–Low Frequency (H-LF)	Sham stimulation	Fugl–Meyer Assessment Scale,<br><br>modified Ashworth Scale of Muscle Spasticity,<br><br>Barthel Index of Activities of Daily Living<br><br>[uses not supplied]	-The LF group improved their FM scores by 11.3 points or 35% from the pretest to posttest (t-test, P< 0.001),<br>-the HF group by 7.6 points or 23% (P <0.000), and<br>-the H-LF group by 9.7 points or 33% (P <0.018),<br>-There was no difference in the sham group (P <0.164)	rTMS positively influenced motor and functional recovery in study participants, demonstrating the clinical potential of the method.	3

			[N/S]						
**71**	Watanabe et al, (2018)	Japan	21 patients<br>**Age** range:, mean (SD) years:<br>iTBS =72.5 (6.5)<br>1-Hz =67.6 (6.4)<br>Sham= 75.2 (5.5)<br>**PSD**= first 7 days<br>**TOS**=ischaemic stroke	intermittent theta-burst stimulation (iTBS) of the affected motor cortex and 1-Hz stimulation of the unaffected hemisphere	Sham stimulation	-FuglMeyer Assessment (FMA),<br>-Stroke Impairment Assessment Set (SIAS),<br>-Modified Ashworth Scale (MAS),<br>-Motor evoked potential (MEP) amplitude<br>-	The MAS wrist and finger score tended to increase in the sham stimulation group (P = 0.078) and in the iTBS group but not in 1-Hz stimulation group	ipsilesional iTBS improves movement of the affected limb, whereas contralesional 1-Hz stimulation reduces spasticity of the affected limb.	1
72	Aşkın et al. (2017)	Turkey	40 participants<br>**Age** range (years):<br>E= 58.80 ± 12.02;<br>C= 56.75 ± 11.46<br>**PSD** (Months):<br>E= 24.35 ± 15.39<br>C= 28.35 ± 15.34<br>**TOS**= ischaemic	Experimental Group (E):low-frequency (LF) rTMS to the primary motor cortex of the unaffected side + physical therapy (PT)	Control Group (C): Physical therapy	-Brunnstrom Recovery Stages.<br>- Fugl–Meyer Assessment,<br>-Box and Block test,<br>-Functional Independence Measurement (FIM)<br>-Functional Ambulation Scale (FAS) scores<br>-Modified Ashworth Scale scores<br>*[The uses were not supplied]*	-FIM cognitive scores and standardized mini-mental test scores were significantly increased and<br>-distal and hand Modified Ashworth Scale scores were significantly decreased only in the rTMS group (p < .05).	LF-rTMS can safely facilitate upper extremity motor recovery in patients with chronic ischemic stroke.	**4**
73	Cho et al. (2017)	Korea	Thirty subacute stroke patients<br>**Age** range (years):<br>E= 60.73 (9.28);<br>C=58.13 (12.52)<br>PSD (days):<br>E=13.8 ± 6.2<br>C=13.6 ± 5.2<br>TOS(ischaemic /haemorrhagic<br>E=12 / 3	Experimental Group (E): Dual-mode stimulation using repetitive transcranial magnetic stimulation (rTMS) and (tDCS)	Control Group (C): Single stimulation using transcranial direct current stimulation	Total Fugl-Meyer (FMA-T), upper limb (FMA-U) and<br>Lower limb (FMA-LL) scores	FMA-T (F = 4.42, df = 2, P = 0.02), FMA-UL (F = 4.13, df = 2, P = 0.02), and FMA-LL (F = 3.80, df = 2, P = 0.03), were significantly improved over time in the Dual stimulation group.	Dual-mode was safe and superior to 10 Hz rTMS alone for improving motor function in subacute stroke patients.	**2**

			C=13 / 2		(rTMS)				
74	Cha and Kim. (2017)	Korea	Thirty subacute stroke<br>**Age** range (years):<br>E1= 55.80±16.40;<br>E2=53.80±13.28 ;<br>C=53.70±9.67.<br>**PSD** (months):<br>E1= 4.20±1.22;<br>E2=3.90±1.59;<br>C= 4.10±1.19<br>**TOS**: both ischaemic and haemorrhagic<br>[N/S]	E1:Ankle strengthening exercise group<br><br>E2:Ankle strengthening exercise integrated group rTMS	C:rTMS group	Motor-evoked potential testing (MEP)<br><br>Peak torque at the ankle joint<br><br>10 m walk test (10MWT)<br><br>[Uses were not stated]	E2 showed significantly higher amplitude of MEP, plantarflexor and dorsiflexor of peak torque, 10 m walk test than E1 and C ($p < 0.05$).<br>E1 and E2 differed significantly in the pre- and post-test for all variables, ($p < 0.05$).<br>In C, the pre- and post-test scores for the amplitude of MEP, dorsiflexor, and 10MWT differed significantly ($p < 0.05$).	Strengthening exercise integrated rTMS has positive effects on motor function recovery in subacute stroke patients.	**4**
**75**	Du et al. (2016)	China	69 stroke patients<br>**Age** range:(years)<br>E1=56.78 ± 8.47<br>E2=56.78± 12.4<br>C=53.61± 13.55<br>**PSD** (days):<br>E1=7 (4–16)<br>E2= 6 (5–12)<br>C=8 (3–24)<br>**TOS**= ischaemic	E1= 3-Hz ipsilesional repetitive transcranial magnetic stimulation (rTMS),<br>E2= 1-Hz contralesional rTMS	C= Sham rTMS	**Motor Impairment:**<br>Lower limb score of Fugl–Meyer Assessment (LE-FMA); Upper limb score of Fugl–Meyer Assessment (UE-FMA); Lower limb score of Medical Research Council score (LE-MRC); andUpper limb score of Medical Research Council score (UE-MRC)<br><br>**Stroke Severity:**<br>National Institutes of Health Stroke Scale (NIHSS); Modified Rankin Scale (mRS); Barthel Index (BI) scale	There was a substantial positive correlation between motor improvement of UE-FMA and increase in the corticospinal excitability (as indexed by rMT) in the affected hemisphere ($r = 0.615$, $P < 0.001$)<br>Nevertheless, there was no correlation between motor function improvement and rMT in the unaffected hemisphere ($r = 0.062$, $P = 0.721$)	Repetitive transcranial magnetic stimulation is a beneficial neurorehabilitative strategy for enhancing motor recovery in the acute and subacute phase after stroke.	4

76	Zheng et.al, 2015	China	P: Poststroke patients with hemiplegia; **Sample size**=108 (E=55, C=53), **Age** (Years): E= 65.4±13.5, C= 66.2 ±13.1; **PSD** (days): E= 19.3±7.3, C= 18.7±8.1 **TOS**(ischemic/hemorrhagic): E = 34/21;C =35/18	E: Low-Frequency, repetitive Transcranial Magnetic Stimulation (rTMS) + Virtual reality (VR) training	C: Sham rTMS + VR	UL Function (UE-FMA, WMFT), -Living Activity (mBI) , - QoL ((SF-36)	- UE-FMA scores [MD=13.2, CI=3.6-22.7, *P*<0.01], - WMFT scores (MD=2.9, CI=2.7-12.3, *P*<0.01), -mBI scores (MD=16.1, CI=3.8-9.4, *P*<0.05) were significantly increased in the experimental group as compared with the control group	The combined use of Low frequency rTMS with VR training could effectively improve the UL function, the living activity, and the QoL in hemiplegic stroke patients	5
77	Wang et al, 2014	Taiwan	P: Patients with stroke who presented with non-fluent aphasia; Sample size= 43 (E1=15, E2=14, C=14); **Mean age** (yrs): E1= 61.3±13.2, E2= 62.1± 12.7, C= 60.4±11.9; **PSD** (months): E1 = 16.8±6.4, E2= 15.7± 8.5, C = 16.1±7.3; **TOS**(ischemic/hemorrhagic): NA	E1 = Transcranial Magnetic Stimulation (TMS) + Synchronous Naming Training (TMSSync)<br><br>E2 = TMS with subsequent Naming Training (TMSSub)	C = Sham TMS + Synchronous Naming Training (TMSSham)	Language Function Domains: (conversation, describing a family picnic picture, and naming objects and their use) assessed using Concise Chinese Aphasia Test (CCAT)	- E1 showed significantly superior results in CCAT total (P<0.001), expression & description subtests (P<0.001), action (P=0.02) and object naming activity (P=0.008).<br><br>- The superior results lasted for 3 months (P=0.005), in comparison with the E2 and C groups.	TMS protocol and language training can be combined to achieve outcomes superior to those obtained when used separately.	4
78	Wang et al, 2014	Taiwan	P: Chronic Stroke Patients; Sample size= 44 (E1=16, E2=14, C=14);	E1= rTMS of contralesional primary motor cortex (cM1)	C= Sham rTMS of cM1	- Hand grip strength and shoulder anterior flexion strength (MRC Scale)	- cPMd modulation yielded significant improvements in distal (p= 0.011) and proximal (p=0.013)MRC, FMA (p= 0.003), and WMFT (p= 0.004) scores compared with sham stimulation;	In patients with chronic stroke, the cPMd can fulfill a role	4

			**Mean age** (yrs): E1= 62.38±12.09, E2= 63.07±12.89, C= 68.00±12.51; **PSD** (months): E1= 5.59±3.86, E2= 8.79±7.55, C = 7.41±8.81; **TOS**(ischemic/hemorrhagic): E1 = 10/6; E2= 8/6; C =11/3	E2=rTMS of contralesional premotor cortex (cPMd)		- UL Motor Funcion Function (UE-FMA),<br>- Joint Segment/ Integrative Movements (WMFT)<br><br>a cortical excitability	- A significant effect on cortical excitability suppression equivalent to that of cM1 modulation, but engendered effects on motor improvement inferior to those of cM1 modulation.	similar to that of the cM1 in interhemispheric imbalance, which can be ameliorated by applying inhibitory rTMS to achieve substantial motor restoration	
**79**	Khedr et al., 2014	Egypt & Germany	**Sample size:** I= 19 C= 10<br><br>**Age Range:** NR<br><br>**Mean Age:**<br>**I=** 61.0 ± 9.8<br>C= 57.4 ± 9.6<br><br>**PSD(weeks):**<br>I= 5.8 ± 4.08<br>C=4.0 ± 2.6<br><br>**TOS(ischemic/hemorrhagic):**NR	Real dual-hemisphere repetitive transcranial magnetic stimulation (rTMS)	Sham dual-hemisphere repetitive transcranial magnetic stimulation (rTMS)	Hand strength and the language assessment section of Hemispheric Stroke Scale (HSS); depression (Stroke Aphasic Depression Questionnaire–Hospital Version (SADQ-H)); functional disability (National Institutes of Health Stroke Scale (NIHSS)); Cortical excitability: resting motor threshold [rMT] and active motor threshold [aMT]) (monophasic magnetic stimulator); aphasia severity (Aphasia Severity Rating Scale (ASRS))	ASRS (*P* = 0.018), HSS (P = 0.004) improved significantly in the real rTMS group compared with the sham group.<br>Also, there was a significant increase in cortical excitability of the affected hemisphere demonstrated by reduced rMT and aMT in the TMS group (*P* = 0.015 and 0.008 respectively) while no such changes were recorded in the sham group (*P* = 0.30). Both groups also showed significant differences for measures of both the rMT and the aMT in the affected hemisphere (*P* = 0.008 and 0.018 respectively).<br>SADQ-H showed a significant Time × Group interaction (*P* = 0.02) with the intervention group having significant differences in scores at post-treatment session and 1-month but not at 2 months after the treatment session (*P*= 0.006, *P*=0 .04, and *P*= 0.05, respectively). There was also a significant Time × Group interaction for the overall NIHSS (*P* = 0.03); however, there were no differences in	Combining dual-hemisphere rTMS with language training might be a feasible treatment for nonfluent aphasia	4

							NIHSS posttreatment and the significant interaction was driven mostly by baseline differences in scores		
**80**	Galvao, et al, 2014	Brazil	**Sample size:**<br>I= 10<br>C= 10<br><br>**Age Range:** NR<br><br>**Mean Age:**<br>I=57.4±12.0<br>C= 64.6±6.8<br><br>**PSD(months):**<br>I=47.8±43.2<br>C=58.9±27.2<br><br>**TOS(ischemic/hemorrhagic):**<br>I = 9/1<br>C =8/2	Repetitive transcranial magnetic stimulation (rTMS) and physical therapy (PT).	Sham Repetitive transcranial magnetic stimulation (rTMS) and physical therapy (PT).	Primary: Spasticity (MAS)<br><br>Secondary: motor function recovery(FMA); Maximum passive range of motion of the paretic wrist Joint (goniometer); The functional independence (the Brazilian-version of FIM); health-related quality of life (The stroke-specific quality-of-life scale (SSQOL))	There was a significant difference in mean MAS scores between the two groups ($P < 0.01$). There was a decrease in the MAS score between the baseline and postintervention ($P<.001$) and between the baseline and follow-up ($P=.03$) for the experimental group, whereas no significant difference was observed in the control group (baseline vs postintervention, $P=.102$; baseline vs followup, ($P=.157$). There were no differences between the groups at any time for any of the secondary outcome measures. At postintervention, when compared with baseline, the participants of both groups achieved an improvement in the UE-FMA and SSQOL total scores. However, only participants in the experimental group showed improved FIM scores and wrist range of movement. At the follow-up examination, a significant improvement in both groups was observed only on the UE-FMA.	rTMS associated with PT can be beneficial in reducing poststroke spasticity	2
**81**	Abo et al., 2013	Japan	**Sample size:** I= 44 C=22<br><br>**Age Range:** NR<br><br>**Mean Age:**<br>I= 57·7 ± 12·7<br>C= 60·3 ± 10·6<br><br>**PSD(months):**<br>I= 62·1 ± 47·7	Low-frequency repetitive transcranial magnetic stimulation Plus occupational therapy (LF-rTMS-OT)	Constraint induced movement therapy (CIMT)	Impairment (FMA); motor functions (WMFT)	FMA score increased significantly in both groups (LF-rTMS-OT: $P < 0·001$; CIMT: $P < 0·005$). Similarly, the decrease in WMFT log performance time was significant in both groups (LF-rTMS-OT, $P < 0·001$; CIMT, $P < 0·005$). In addition, FAS of WMFT also increase significantly in both groups (LF-rTMS-OT $P < 0·001$; CIMT, $P < 0·005$). A significantly larger increase	both intervention provided improved motor and functional outcomes, with Low-frequency repetitive transcranial	3

			C=68·0 ± 53·1<br><br>**TOS(ischemic/hemorrhagic):**<br>I = 18/26<br>C =11/11				in FAS of WMFT was significantly improved in the intervention group compared with the CIMT group (*P* < 0·05).	magnetic stimulation plus occupational therapy (LF-rTMS-OT) providing better effect	
**82**	Barwood et al., 2013	Australia	**Sample size:** I= **6** C= 6<br><br>**Age Range:**<br>I= 54 – 67<br>C= 51 – 85<br><br>**Mean Age:**<br>I= 60.8+5.98<br>C= 67 +13.11<br><br>**PSD(years):**<br>I= 3.49 +1.27<br>C= 3.46 +1.53<br><br>**TOS:** NR	Active Low-frequency repetitive transcranial magnetic stimulation (rTMS)	Sham Low-frequency repetitive transcranial magnetic stimulation (rTMS)	behavioural language function (standard form of the Boston Naming Test [BNT] and selected subtests of the Boston Diagnostic Aphasia Examination [BDAE]); picture naming (Snodgrass and Vanderwart picture-naming)	Significant group x time interaction were identified for the following subtests: BDAE naming actions (P < 0.01), BDAE naming tools and instruments (P < 0.05), BDAE repetition of sentences (P < 0.05), Cookie Theft picture description complexity index (P < 0.05), Cookie Theft picture description longest words per phrase (P < 0.01), Commands (P < 0.05), the overall score calculated from the BDAE subtests administered (P < 0.01), The BNT (P < 0.05), Snodgrass and Vanderwart picture-naming latency (P < 0.05) and Snodgrass and Vanderwart picturenaming accuracy (P < 0.05). Significant differences between the two groups were found in favour of the intervention group for a number of language subtests: BDAE naming actions (P < 0.01), BDAE naming tools and instruments (P < 0.05), Cookie Theft picture description complexity index (P < 0.05), BDAE overall score (P < 0.05) and picture-naming accuracy (P < 0.05). However, The real stimulation group performed significantly lower on the Snodgrass and Vanderwart picture-naming latency (P < 0.05). The intervention group also showed	These findings provide considerable evidence to support the theory of rTMS modulating mechanisms of transcallosal disinhibition in the aphasic brain and highlight the potential clinical applications for language rehabilitation post-stroke.	3

							significant improvement in BDAE naming actions (P < 0.05), BDAE naming of tools and instruments (P < 0.01), BDAE overall score (P < 0.01), Commands (P < 0.05) and picture naming accuracy (P < 0.05). Picture-naming latency was significantly lower (P < 0.05). No significant results within the sham group across time were found.		
**83**	Thiel et al., 2013	NY	**Sample size:**<br>I= 13 C= 11<br><br>**Age Range:** NR<br><br>**Mean Age:**<br>I= 69.8±7.96<br>C= 71.2 ±7.78<br><br>**PSD(days):**<br>I=37.5 ±18.52<br>C= 50.6 ±22.63<br><br>**TOS:**NR	Inhibitory repetitive transcranial magnetic stimulation (rTMS) over the right triangular part of the posterior inferior frontal gyrus followed by 45 minutes of speech and language therapy	Sham repetitive transcranial magnetic stimulation (rTMS) over the right triangular part of the posterior inferior frontal gyrus followed by 45 minutes of speech and language therapy	Language (Aachener Aphasia Test (AAT))	Global AAT score was significantly higher in the TMS group than in the sham group (*P*=0.003). A highly significant overall treatment effect (*P*=0.003) was also found in the subtest analysis. There was no significant interaction between treatment and subtest. The mean increase in subtest scores was higher for the TMS group in all subtests.	Inhibitory repetitive transcranial magnetic stimulation (rTMS) over the right triangular part of the posterior inferior frontal gyrus followed by 45 minutes of speech and language therapy significantly improve language recovery in sub-acute ischemic stroke.	2

**84**	Sung et al., 2013	Taiwan	**Sample size:**<br>I=15<br>C1 = 12<br>C2=13<br>C3=14<br><br>**Age Range:** NR<br><br>**Mean Age:**<br>I= 62.3 + 12.2<br>C1 = 64.2 + 11.9<br>C2= 63.3 + 12.8<br>C3=63.1 + 12.8<br><br>**PSD(months):**<br>I = 7.8 + 1.7<br>C1 = 8.1 + 1.5<br>C2 = 7.9 + 2.0<br>C3 = 8.2 + 1.6<br><br>**TOS: ischemic/hemorrhagic :**<br>I = 10/5<br>C1 = 8/4<br>C2 = 8/5<br>C3 = 9/5	G1= 1 Hz Repetitive transcranial magnetic stimulation (rTMS) over the controlesional primary motor cortex (cMI) and then intermittent theta burst stimulation (ITBS) over the ipsilateral MI (iM1)	C1= 1 Hz Sham rTMS over cM1and ITBS over iM1<br><br>C2= 1 Hz rTMS over cM1 and sham ITBS over iM1<br><br>C3= 1 Hz sham rTMS over cM1 and then sham ITBS over iM1	Motor function (Wolf Motor Function Test [WMFT]); motor impairment (upper extremity FuglMeyer Assessment [FMA]); finger flexors strength (Medical Research Council (MRC) Scale); muscle activities (simple reaction time task (RT), and index finger tapping task (FT) assessed using Electromyography); Electrophysiological measures: Bilateral corticomotor excitability (monophasic magstim200); probing rMT, maximal amplitude, latency of MEP, and motor map area were measured using Dantec Keypoint electromyography (EMG)	Th intervention group showed a higher MRC score compared with group C1 (p = 0.003) and C2. (p = 0.001). For the FMA, the intervention group also showed greater significant improvement compared with groups C1 (p =0.001) and C2 (p =0.001). For WMFT, the intervention group also showed higher significant improvement compared with group C1 (p = 0001) and C2 (p = 0.001). For RT the intervention group showed higher significant improvement compared with groups C1 (p = 0.001) and C2 (p = 0.003). For cortical excitability, the motor map area measurements in the contralesional hemisphere showed significant decrements in the intervention group (p = 0.002), Group C1 (p = 0.0012) and C2 (p = 0.004). In addition, the intervention group showed significant change in contalesional MEP amplitude (p = 0.03) and latency (p = 0.021). The ipsilateral motor map area of the intervention group and group C1 were significantly enlarged compared with C3 (p = 0.002 and 0.004 respectively)	Coupling inhibitory-facilitatory TMS enhances motor recovery of chronic stroke patients	5
**85**	Waldowski, et al, 2013	Poland	**Sample size:** I=8<br>C=10<br>**Age Range:** NR<br>**Mean Age (years):**<br>**I**=63 ±12.07<br>C= 58.7 ±11.38<br>**PSD(years):**<br>I= 13.3 ±4.06<br>C= 11.1 ±2.08<br>**TOS: ischemic/ hemorrhagic :**	Speech and language therapy (SLT) + real low-frequency repetitive transcranial magnetic stimulation (rTMS)	SLT + sham rTMS	Naming assessment: accuracy of naming and reaction time (RT) (Computerized Picture Naming Test (CPNT)); language abilities (Boston Diagnostic Aphasia Test (BDAE)); aphasia severity (Aphasia Severity Rating Scale (ASRS))	Both groups showed significant improvement in their naming abilities, however, there was no significant difference in average test scores between groups at any time. The experimental group showed better scores in average RT immediately after rTMS treatment (p = 0.048). no other significant between group difference were seen in all the other measured outcomes.	SLT+rTMS do not appear to be more effective in improving naming in early-stroke aphasia patients than SLT+sham rTMS	5

			NR						
86	Senió w, et al., 2012	Poland	**Sample size:**<br>I=20 C=20<br>**Age Range:**<br>I=48-79<br>C=46-78<br>**Mean Age**: I= 63.5±8.9<br>C=63.4 ±9.2<br>**PSD** (Days):<br>I=41.7 ±21.3<br>C=38.0±26.6<br>**TOS:**<br>Ischemic	Physiotherapy (PT) with Low-frequency repetitive transcranial magnetic stimulation (rTMS) to the contralesional primary motor cortex (cM1)	PT + sham-rTMS to cM1	PO: Functional assessment of the paretic hand (Wolf Motor Function Test [WMFT-FAS and WMFT-TIME]),<br>SO: level of neurological deficit (National Institutes of Health Stroke Scale [NIHSS]), motor impairment (the upper extremity part of the Fugl-Meyer Motor Assessment (FMA))	In post-treatment assessment, the results obtained by both groups in all 3 outcome measures were significantly higher than baseline scores. However, there were no significant between-group differences in WMFT–FAS (*P* = .92), WMFT–TIME (*P* = .29), FMA (*P* = .68), or NIHSS (*P* = .82).	The study revealed that type of treatment had no significant impact on the clinical or functional state of our patients	4
87	Sasaki, et al, 2013	Japan	**Sample size:**<br>I : G1 = 9<br>G2 = 9<br>C= 11<br>**Age Range:**<br>I=NR<br>C=NR<br>**Mean Age** (Years):<br>I : G1 = 63.0 ± 9.3<br>G2 = 65.7 ±<br>C= 68.6 ± 8.7<br>**PSD** (days):<br>I : G1 = 15.4<br>G2 = 18.4<br>C= 17<br>**TOS**: cerebral infarction/ICH, intracerebral hemorrhage I :<br>G1 = 4/5<br>G2 = 4/5<br>C= 5/6	G1 = high frequency repetitive transcranial magnetic Stimulation (HF-rTMS: 10 Hz rTMS) to the lesional hemisphere (LH), + conventional Rehabilitation (Rhb)<br><br>G2 = low frequency-1Hz (LF-rTMS) to the non-LH, + Rhb	The sham stimulation and conventional rehabilitation	Grip strength (standard Jamar dynamometer); tapping frequency (National Institute of Health Stroke Scale [NIHSS] score and the finger-hand and upper-limb subitems of the Brunnstrom Recovery Stage [BRS])	Both the HF-rTMS and LF-rTMS groups had significant increases in both grip strength and tapping frequency. Comparison of the extent of improvement showed a more significant increase in grip strength and tapping frequency in the HF-rTMS group compared to the sham stimulation group (each P <.05), and no difference between the LF-rTMS group and the sham stimulation group. .	HF-rTMS applied to the lesional hemisphere in the early phase of stroke was more beneficial for motor improvement of the affected upper limb than LF-rTMS	3

**88**	Wang et al., 2012	Taiwan	**Sample size:** I = 12 C = 12<br>**Age Range:** NR<br>**Mean Age** (Years):<br>I = 64.90 + 12.37<br>C = 62.98 + 10.88<br>**PSD** (days): I= 1.84 + 1.16<br>C= 2.00 + 1.23<br>**TOS**: NR	Repetitive transcranial magnetic stimulation (rTMS) followed by task-oriented training (TOT) 30 minutes for 10 sessions over 2 weeks.	Sham rTMS followed by TOT (30 minutes) for 10 sessions over 2 weeks.	The primary outcomes: corticomotor excitability symmetry (Motor evoke potential, MEP) and gait symmetry.<br>The secondary outcomes: motor control (the Fugl-Meyer assessment score (FMA)) and spatial and temporal parameters of gait (GAITRite system)	Decreased interhemispheric asymmetry of the amplitude of the MEP was noted after rTMS and taskoriented training. Improvement in spatial asymmetry of gait was comparable with increased symmetry in interhemispheric excitability in the intervention group. Motor control and walking ability were also significantly improved after rTMS and task-oriented training.	rTMS enhances the effect of TOT in those with chronic stroke, especially by increasing gait spatial symmetry and corticomotor excitability symmetry.	5
89	Marconi, et al., (2011)	Italy	**Sample size: I=** 15 C= 15<br><br>**Age Range:** NR<br><br>**Mean Age:**<br>**I=** 63.6 + 7.6<br>C= 66.3 + 11.0<br><br>**PSD(days):**<br>I= 39.9 + 28.8<br>C= 40.6 + 25.1<br><br>**TOS(thromboemboli/hemorrhagic** NR	Physiotherapy with repeated muscle vibration (rMV) using transcranial magnetic stimulation (TMS),	Physiotherapy alone	Primary outcomes: resting motor threshold (RMT), map area, map volume, short-interval intracortical inhibition (SICI), and intracortical facilitation (ICF), muscle tonus and motor function were measured using electromyography secondary outcome: Spasticity (Modified Ashworth Scale [MAS]), motor function was assessed by the Motricity Index [MI] for the upper limbs), and functional ability (Wolf Motor Function Test–Functional Ability (WMFT-FA).	Pre–post analysis revealed a reduction in RMT and and increase in motor map areas occurred in the vibrated muscles only in the rMV + PT group, with an increase in map volumes of all muscles. Moreover, SICI increased in the flexors and decreased in the extensor. These neurophysiological changes lasted for at least 2 weeks after the end of rMV + PT and paralleled the reduction in spasticity and increase in motor function in the intervention group. the iintervention group also showed significant improvement in MI and WMFT-FA.	rMV with PT may be used as a nonpharmacological intervention in the neurorehabilitation of mild to moderate hemiparesis.	1
90	Chang, et al., (201	Korea	**Sample size:** I=18 C= 10<br><br>**Age Range:** I= NR C= NR	Real Repetitive transcranial magnetic stimulation (rTMS) with	Control Repetitive transcranial	Motor function of the affected upper limb (Motricity Index [MI], the upper limb score in the Fugl-Meyer	Motor function improved in both groups after treatment; however, patients who received real rTMS experienced additional improvement in motor function of the affected upper	Positive long-term effects on motor recovery could be	5

	0)		**Mean Age:**<br>I= 56. +11.2<br>C= 57.0+14.5<br><br>**PSD(days):**<br>I= 12.9+5.2<br>C= 14.4+5.9<br><br>**TOS(thromboembloli/hemorrhagic):** NR	conventional, physical, and occupational therapy,	magnetic stimulation (rTMS) with conventional, physical, and occupational therapy,	assessment [FMA-UL], and the Box and Block test (BBT)). Motor function of the affected lower limb (leg score in MI [MI-L] and the lower limb score in FMA (FMA-LL). Mobility (Functional Ambulatory Category [FAC], ADL (modified Barthel index)	limb. Over 3 months after the stroke, the time and type of intervention for the Motoricity Index of the affected upper extremity showed significant interaction. Both groups showed significantimprovement in MI-L ($p <$ 0.0001) and FMA-LL ($p < 0.0001$). Also, Both groups demonstrated significant improvement in FAC and MBI over time ($p < 0.0001$); however, there was no interaction effect between group and time	achieved after 10 daily sessions of high-frequency rTMS in conjunction with motor practice during the subacute period of stroke.	
**91**	Kim, et al., 2010	Korea	**Sample size:**<br>I: G1 =6<br>G2=6<br>C=6<br>**Age Range:**<br>I=NR C= NR<br>**Mean Age: I: g1** = 68.3 + 7.4<br>G2=53.5 + 16.9<br>C=66.8 +17.2<br>**PSD(days):**<br>I: G1 =404.4 + 71.7<br>G2 = 241.2 + 42.5 C=69.7 + 39.0<br>**TOS(ischemic/others):** G1= 5/1 G2 = 4/2 C = 5/1	-G1 = low frequency(1 Hz) repetitive transcranial magnetic stimulation [rTMS] applied over the left dorsolateral prefrontal cortex (DLPFC)<br>-G2 = high frequency (10 Hz) rTMS applied over the left DLPFC	Sham rTMS applied over the left DLPFC.<br><br>NB: all the groups received conventional cognitive rehabilitation	Cognition (The Seoul Computerized Neuropsychological Test), executive function (Tower of Hanoi task), ADL (Modified Barthel Index[MBI]), mood (Beck Depression Inventory[BDI)	There was no significant change in post treatment mean scores between the groups in all the measured outcomes. G2 showed a significant improvement in BDI. There is a a significant improvement in MBI for all the three groups ($P = 0.04$; $P =$ 0.04; and $P = 0.03$, for the low-frequency, high-frequency, and sham groups, respectively)	There was a positive effect of all the interventions on mood, but no measurable effect on cognition. and none has a higher efficacy than the other	3
**92**	Khedr & Abo-Elfetoh, 2010	Egypt	**Sample size:** I = 11<br>C= 11<br><br>**Age Range:** NR	Traditional rehabilitation plus Active repetitive transcranial magnetic	Traditional rehabilitation plus Sham repetitive	Dysphagia (Dysphagia rating scale); Grip strength (according to the Hemispheric Stroke Scale). Stroke severity (The National Institutes	Active rTMS improved dysphagia compared with sham rTMS in both groups (p = 0.001 for both); NIHSS and BI improved in all patients during the course of follow-up, with this being significantly larger in the active	rTMS could be a useful adjuvant strategy in neurorehabilitation of	1

			**Mean Age**:<br>NR<br><br>**PSD** : NR<br><br>**TOS:** NR	stimulation (rTMS)	transcranial magnetic stimulation (rTMS)	of Health Stroke Scale [NIHSS]) and ADL (Barthel index scale (BI))	rTMS group for the BI but not NIHSS. There is also a significant interaction (group X time) for the BI measure (p = 0.001). Although hand grip strength and NIHSS were improved over period of study in both patient groups, there was no significant difference between active and sham rTMS treatment (p = 0.67 and p = 0.52, respectively). All improvements were maintained over 2 months of follow-up (p= 0.001).	dysphagia, although further assessment is necessary in multicentre clinical trials.	
**93**	Khedr, et al, 2009	Egypt	**Sample size:** I =<br>G1 = 12<br>G2 = 12<br>C= 12<br><br>**Age Range:**<br>I=NR<br>C=NR<br>**Mean Age** (Years):<br>I: G1 = 54.7 ± 9.7<br>G2 = 59.0 ± 13.5<br>C= 60.0 ± 9.5<br><br>**PSD** (days):<br>**I: G1 =** 16.3 ± 3.6<br>G2 = 17.2 ± 3.6<br>C = 17.7 ± 3.8<br><br>**TOS:** ischemic	G1 = 1 Hz repetitive transcranial magnetic stimulation (1Hz-rTMS) over the unaffected hemisphere<br><br>G2 = 3 Hz repetitive transcranial magnetic stimulation (3Hz-rTMS) over the affected hemisphere	Sham repetitive transcranial magnetic stimulation (Sham-rTMS) over the affected hemisphere	Strength of hand grip (Medical Research Council 1986, Keyboard Tapping and Pegboard Task); Severity of stroke (National Institutes of Health Stroke Scale (NIHSS); functional ability (Barthel index scale (BI). Cortical excitability [resting and active motor threshold (AMT) and motor evoked potentials (MEPs)] were assessed using electromyography	At post intervention, There was significantly higher improvement in the 1 Hz group in comparison with the 3 Hz group in NIHSS and pegboard scales. Significant improvements were noticed in keyboard tapping and pegboard collection in each real group separately in comparison with the sham (P = 0.001 for all). On the other hand, the improvement was significantly more pronounced in 1 Hz group in comparison with the 3 Hz group for both tasks (P = 0.001 for both rating scales). Whilst no significant differences in hand grip were detected. All groups of patients were improved in both the NIHSS and BI scales at 3 months with the intervention group significantly more than control	These results confirm that five daily sessions of rTMS over motor cortex using either 1 Hz over the unaffected hemisphere or 3 Hz over the affected hemisphere can enhance recovery. At 3 months, the improvement was more pronounced in 1 Hz group.	2
**94**	Takeuchi et al, 2008	Japan	**Sample size:** I= 10 C= 10<br><br>**Age Range:** NR	sub-threshold repetitive transcranial magnetic	sham repetitive transcranial	The maximum pinch force of the affected hand (a pinch gauge); Movement acceleration	Compared with sham stimulation, rTMS induced a higher increase in the excitability of the affected motor cortex ($p < 0.001$) and an	rTMS improved the motor learning of	2

			**Mean Age:**<br>**I=** 61.2+9.7<br>C= 63.4+7.4<br><br>**PSD(months):**<br>I= 25.4+20.8<br>C= 34.4+38.6<br><br>**TOS(ischemic/hemorrhagic):**<br>I = NR<br>C =NR	stimulation [rTMS] over the unaffected hemisphere	magnetic stimulation [rTMS] over the unaffected hemisphere	(an accelerometer); cortical excitability (transcranial magnetic stimulation (TMS).	improvement in acceleration of the affected hand (*p* = 0.006). Moreover, the effect of motor training on pinch force was enhanced by rTMS (*p* < 0.001). These improvement in the motor function lasted for one week after rTMS and motor training (*p* < 0.001). a significant<br>Interaction was shown between time and condition with respect to acceleration (*p* = 0.033) and pinch force; *p* = 0.013). It also showed a significant effect<br>of time on both acceleration (*p* < 0.001) and pinch force (*p* < 0.001).	the affected hand in patients after stroke; thus, it can apply as a new rehabilitation strategy for patients after stroke	
**95**	Takeuchi et al, 2005	Japan	**Sample size:** I= 10 C= 10<br>**Age Range:** NR<br>**Mean Age:**<br>**I=** 58.4+6.6<br>C= 59.6+12.3<br>**PSD(months):**<br>I= 25.2+18.4<br>C= 28.7+16.7<br>**TOS(ischemic/hemorrhagic/unidentified):**<br>1 = 51/5/0<br>C = 49/10/3	subthreshold repetitive transcranial magnetic stimulation rTMS	Sham repetitive transcranial magnetic stimulation rTMS	pinch force and acceleration, rest motor threshold (rMT), amplitude of the motorevoked potentials (MEPs), and transcallosal inhibition (TCI) duration were assessed using TMS	When compared with sham stimulation, rTMS reduced the amplitude of motor-evoked potentials in contralesional motor cortex (M1), and the TCI duration. Also, the intervention group immediately induced an improvement in pinch acceleration of the affected hand, although a plateau in motor performance had been reached by the previous motor training.	subthreshold repetitive transcranial magnetic stimulation rTMS provided a significant improvement in all the measured outcomes.	4

Key: PSD - poststroke duration; TOS. Quality score: assessed with Jadad quality assessment scale for rating randomized controlled trials; N/A = Not Available or Applicable

PFMT = Pelvic Floor Muscle Training; LUTS = Lower Urinary Tract Symptoms; E = Experimental; C = Comparison/Control; I = Ischaemic Stroke; H = Haemorrhagic Stroke; ES = Electrical Stimulation; VDS = Videofluoroscopy Dysphagia Scale; PAS = Penetration–Aspiration Scale; CT = Conventional Treatment; NFT = Neuromuscular Faccilitation; BMD = Bone Mineral Density, ALP = Alkaline phosphatase; BGP = Bone gla protein; IL-6 = Interleukin-6; PNS = Peripheral Nerve Stimulation; mCIT = modified Constraint Induced Movement Therapy; WMFT = Wolf Motor Function Test; FMA = Fugl-Meyer Assessment Scale; ARAT = Action Reach ArmTest; UE = Upper Extremity; mAS = Modified Ashworth Scale; MBEST = Mini Balance Evaluation Systems Test, TUG = Time up and Go; 6MWT = Six minutes walk test;ABC =Activity Specific Balance; FAI = Frenchai Activity Index; CHIEF = Craig Hospital Inventory of Environmental Factors; SIS- = Stroke Impact Scale ; mCIMT = Modified Contraint Induced Movement Therapy; rTMS = Repetitive transcranial magnetic stimulation; BI = Barthel Index; HADS-Thai = Thai version of the Hospital Anxiety and Depression Scale; DEXA = Dual energy-ray

Absorptometry; LWBV = Low intensity Whole Body Vibration; HWBV = High intensity Whole Body Vibration; MFT = Manual Function Test; VD = Vertical Displacement (Shoulder Subluxation); UL = Upper Limb; MAL = Motor Activity Log; VO2 peak, 6MWD = 6-minute walk distance, 30WT = 30-ft Walk Times; 48SC = 48-hr step counts; TDx = Treadmill; SAM = Step Activity Monitoring; BCI-FES = Brain–Computer Interface controlled Functional Electrical Stimulation; MAL = Motor Activity Log; mBI = modified Berthel Index; AOT = Action Observational Training; SMA = Stride Management Assist; FTST = Functional Task Specific Training; m = Median;

Functional Electrical Stimulation (FES)

Ref	Study (Authors, year	Country	Participants	Interventions / Model of care	Control/ comparison	Outcomes assessed/ outcome measures	Results	Conclusion	Quality score
**96**	Ganesh et al, (2018)	India	Eighty-three patients<br>**Age** range: mean age of 57.12 years<br>**PSD**=N/A<br>**TOS**= (Ischaemic/Haemorrhagic)<br>C=19/7, E1=24/3 E2=23/7	E1= Faradic current for 10 min andtask-oriented exercises<br>E2= Russian current for 10 min and task-oriented exercises	C= Task-oriented exercises	- Soleus and Gastrocnemius muscles spasticity [Modified Ashworth scale];<br>- Passive range of movement ROM [Goniometer]<br>- Functional Ambulation [Modified Emory Functional Ambulation Profile]	Both the types of stimulation and exercises were not associated with improvements in modified Emory Functional Ambulation Profile ($p > 0.05$).<br>- The results showed that all the groups were effective in improving passive ankle ROM ($p < 0.05$) and reducing soleus and gastrocnemius muscles spasticity ($p < 0.05$).	Electrical stimulation plus exercises are associated with low to medium effect sizes in reducing spasticity and improving ankle ROM	1
**97**	Dujović et al, (2017)	Spain	16 participants<br><br>Age range: N/A<br><br>PSD: N/A<br><br>TOS= both ischaemic and haemorrhagic combined [N/S]	Functional electrical stimulation (FES) plus conventional rehabilitation program (FES+Rhb)	Conventional rehabilitation program (Rhb)	-10 Meter Walk Test (10 MWT)<br>-Fugl-Meyer Assessment (FMA), used to measure motor impairment.<br>-Berg Balance Scale (BBS) used to measure static and dynamic balance function<br>-Modified Barthel Index (mBI) used for activities of daily living (ADL), functional mobility and gait	Results showed a significant increase in gait speed in FES group ($p < 0.001$)	FES+Rhb is more effective on walking speed, mobility of the lower extremity, balance disability and ADLcompared to Rhb only.	1
98	Marquez-	Canada	21 participants<br>**Age** range:<br>FES= 32–74yrs	Functional electrical stimulation	Conventional therapy	-Functional Independence Measure Self-Care evaluates	Functional Independence Measure Self-Care subscores increased by 22.8±6.7 points in the intervention group and 9±6.5	FES therapy results in a significantly	**1**

	Chin et al, 2017.		Control= 29–82yrs<br>**PSD**= 15 to 57 days<br>**TOS**: Both ischaemic and haemorrhagic (N/S)	therapy (FES)		eating, grooming, bathing, dressing upper body, dressing lower body, and toileting<br>-Fugl-Meyer Assessment-Upper Extremity [FMA-UE]	in the control group, following 40 hr of equal-intensity therapy.<br><br>FMA-UE score changes were 27.2±13.5 and 5.3±11.0 for the intervention and control groups, respectively.	greater recovery when added to standard physical and occupational therapy.	
**99**	Knutson et al,. (2016)	USA	80 stroke patients<br>**Age** range:<br>E=55.4 (17.0)yrs<br>C=56.3 (12.7)yrs<br>**PSD:**<br>E=1.8 (2.5)yrs<br>C=1.6 (4.9)yrs<br>**TOS:** (ischaemic/ haemorrhagic)<br>E=35/5; C= 32/8	E:Contralaterally controlled functional electrical stimulation (CCFES)	C: Cyclic neuromuscular electrical stimulation (cNMES).	Box and Block Test (BBT)- manual dexterity<br><br>UE-FMA- upper limb impairment<br>Arm Motor Abilities Test- functional ability	CCFES group had greater improvement on the BBT, 4.6 (95% confidence interval [CI], 2.2–7.0), than the cNMES group, 1.8 (95% CI, 0.6–3.0), between-group difference of 2.8 (95% CI, 0.1–5.5), P=0.045. No signifiant between-group difference was found for the upper extremity Fugl–Meyer (P=0.888) or Arm Motor Abilities Test (P=0.096).	CCFES improved hand dexterity more than cNMES in chronic stroke survivors.	4
**100**	Carrico et al, 2016	USA	Sample size= 19,<br>**Mean age**=N/A<br>Age range= 35-66yrs; **PSD**: N/A<br>**TOS**(ischemic/hemorrhagic):<br>E = 9/1; C =8/1	Active Peripheral Nerve Stimulation (PNS) + modified Constraint Induced Movement Therapy (mCIT)	Sham PNS + mCIT	- Wolf Motor Function Test WMFT (UL Motor Capacity)<br>- Fugl-Meyer Assessment Scale FMA (UL Motor Function)<br>- Action Reach ArmTest ARAT (UL Motor Capacity)	Active PNS enhanced mCIT more than sham PNS for<br>- WMFT-timed score (P=0.006) and lift score (P=0.001);<br>- FMA (p=0.022); and<br>- ARAT (p=0.007)	Pairing PNS with mCIT can improve UE movement function more than mCIT alone	5
**101**	Jang et al, 2016	Korea	P: patients who were diagnosed with hemiparetic stroke.<br>Sample size =20<br>E/C=10/10;<br>**Mean age** (years):<br>E= 61.10 (13.77),<br>C= 61.70 (12.09);<br>**PSD** (years): E= 4.40±0.97, C=	Brain–Computer Interface controlled Functional Electrical Stimulation (BCI-FES)	Functional Electrical Stimulation (FES) only	-Shoulder Subluxation (Pre and post Rehabilitation X-ray)<br>-Pain Intensity (10cm VAS)<br>-Spasticity (MAS)<br>Upper limb function (MFT)	-Significant differences between groups on the three outcome measures, VD (p = 0.004) and two items of the MFT (shoulder flexion, p = 0.029, and shoulder abduction, p = 0.016), compared with the FES group.<br>-Significant reduction in pain within both groups, but not between groups.<br>-No significant differences in spasticity within or between groups.	-BCI-FES training with conventional therapy may be effective in enhancing improvement in shoulder subluxation of patients with stroke	3

			4.10±0.74;<br>**TOS**(ischemic/hemorrhagic):<br>E = 6/4<br>C =6/4					by facilitating motor recovery.	
**1 0 2**	Kim et al, 2016	Korea	P: Sample size=30 ,<br>Mean age=NA (E= 59.07± 8.07, C=59.93 ± 9.79);<br>**Age** range=NA;<br>**PSD**: 12 months ;<br>**TOS**(ischemic/hemorrhagic):<br>E = 8/7; C =6/9	Action Observational Training (AOT)+BCI-FES + Conventional Therapy	Conventional therapy	-UL Motor Function (Fugyl Myer Upper Extremity subscale-UE-FMA), -ADL Quality of movement (Motor Activity Log - MAL),<br>-ADL (modified Barthel Index - mBI) and<br>-Wrist joint ROM (Goniometer)	After 4 weeks, the UE-FMAs (total, shoulder and wrist), MAL (MAL-Activity of Use and Quality of Movement), mBI and wrist flexion ROM were significantly higher in the BCI-FES group ($p<0.05$).	AOT+BCI-FES is effective in paretic arm rehabilitation by improving the UE performance. AOT+BCI-FES can be used as a therapeutic tool for stroke rehabilitation.	3
**1 0 3**	Bethoux et.al, 2015	USA	P: patients with chronic stroke<br>Sample size=495 (E=242, C=253),<br>**Mean age** (years): E=63.87 ± 11.33, C=64.30 ± 12.01<br>**PSD**: E= 6.90 ± 6.43, C= 6.86 ± 6.64<br>**TOS:** NA	FES device [WalkAide (WA)]	Ankle Foot Orthotics (AFO)	-Gait speed (10MWT),<br>-Walking endurance (6MWT),<br>- Gait quality (FAP), and<br>- Performance of functional ambulation (mEFAP)	- FES proved non-inferior to AFOs for all primary endpoints.<br>-Both FES and AFO groups showed statistically and clinically significant improvement for 10MWT compared with initial measurement.<br>-No statistically significant between-group differences were found for primary or secondary endpoints.<br>-The FES group demonstrated statistically significant improvements for 6MWT and mEFAP Stair-time subscore.	-long-term FES use may lead to additional improvements in walking endurance and functional ambulation	3
**1 0 4**	Chen et.al, 2014	China	P: subjects with early stroke<br>Sample size=48 (E1=18, E2=15,C=15),<br>**Mean age** NA<br>**PSD:** NA	E1=.four-channel FES (based on normal walking pattern) + General	C=comfort stimulation<br>+<br>GM&SR	- Motor impairment (FMA),<br>- posture (PASS),<br>- Dynamic balance(BBA),<br>- Static balance(BBS),<br>- ADL (mBI).	- For intra-group comparisons versus pre-treatment, at week 1, 2 and 3, the scores of PASS, BBA, BBS, FMA and mBI had statistically significant differences ($P < 0.05$);<br>- At week 3 post-treatment, when four-channel and double-channel FES groups	Compared with traditional dual-channel FES, functional electrical	3

			**TOS**(ischemic/hemorrhagic):<br>E = 14/11<br>C =12/13	medication & standard rehabilitation (GM&SR)<br>E2= dual-channel FES + GM&SR			were compared versus pre-treatment, the scores of ipsilateral FMA had statistically significant differences (P < 0.05).<br>- At week 1 post-treatment, mBI had statistically significant difference among 3 groups (P = 0.037) in favour of Four channel FES	stimulation based on human walking patterns is more efficacious. and it helps to restore brain structure and function and promote motor function recovery in patients with early stroke	
**105**	Bethoux , et al., 2014	USA	**Sample size:** I= 242 C= 253<br><br>**Age Range:** NR<br><br>**Mean Age:**<br>I=63.87 ± 11.33<br>C= 64.30 ± 12.01<br><br>**PSD(years):**<br>I=6.90 ± 6.43<br>C=6.86 ± 6.64<br><br>**TOS(ischemic/hemorrhagic):**NR	Wore peroneal nerve functional electrical stimulator (FES) for 6 months	Wore ankle-foot orthoses (AFO) for 6 months	Primary: walking ability and gait velocity (10MWT); mobility, ADL/IADL and Social Participation (domains of SIS).<br>Secondary: walking endurance (6-Minute Walk Test [6MWT]); general ambulation ability (GaitRite Functional Ambulation Profile [FAP]); peformance in functional mobility tasks Modified Emory Functional Ambulation Profile (mEFAP), balance (Berg Balance Scale [BBS] and Timed Up and Go [TUG] test), quality of	There were no statistically significant between-group differences in mean gait velocity or SIS composite score. Both groups demonstrated statistically significant improvement in gait velocity (*P* < .001). for the secondary outcomes, Significant within-group differences between baseline and 6 months were noted in the intervention group for GaitRite FAP score (*P* = .001), total mEFAP time (*P* < .001), and the mEFAP subtasks of Floor time (*P* = .001) and Obstacle Course time (*P* = .001). No significant between-group differences were found for these variables. Statistically significant improvement on BBS score was noted for the intervention group (*P*= .001). Neither the intervention group nor control group exhibited statistically significant improvement in time to complete the TUG. No significant between groups differences were found	Use of FES is equivalent to the AFO in the improvement of gait and quality of life in post stroke individuals	3

						life (Stroke-Specific Quality of Life [SSQoL]), and stroke impact (SIS)	for these variables. Neither group demonstrated any significant within group improvement for the individual domains of the SIS or for SSQoL score from baseline to 6-month follow-up. No significant between-group differences were found for these variables.		
**106**	Kim et al, 2013	Korea.	**Sample size:** I= 12 C=11<br><br>**Age Range:** NR<br><br>**Mean Age:**<br>I= 55.92 $\pm$ 11.75<br>C= 55.64 $\pm$ 12.61<br><br>**PSD(days):**<br>I= 34.06 $\pm$ 1.65<br>C=35.00 $\pm$ 15.05<br><br>**TOS(ischemic/hemorrhagic):**<br>I = 7/5<br>C = 7/4	Functional electrical stimulation (FES) with mirror therapy (MT)	Functional electrical stimulation (FES) without mirror therapy (MT)	Motor recovery (FMA and Brunnstrom's motor recovery stage [BMRS]); degree of motor skill disability (The manual function test); gross manual dexterity (BBT);	In the FMA, scores for shoulders, lower arms, wrists, hands, and upper limb co-ordination increased significantly after intervention in both groups ($P < .05$). On the other hand, the motor skills of the wrist and hand increased significantly more in the experimental group than in the control group ($P < .05$). For BMRS, both groups showed a significant increase in upper limb and hand scores after intervention ($P < .05$), but the experimental group showed significantly better hand recovery than the control group ($P < .05$). The manual function test showed significant increases in functions of the shoulders and hands after intervention in both groups after intervention ($P < .05$), but hand function increased significantly more in the experimental group ($P < .05$). For BBT, both groups showed a significant increase after intervention ($P < .05$). However, in this case, no significant intergroup differences were observed before or after testing.	FES with MT during poststroke rehabilitation may effectively improve motor functions of the upper extremity	3
**107**	Lo et al, 2012	Taiwan	**Sample size:** I = 10<br>C= 10<br><br>**Age Range:** I=NR C=NR<br>**Mean Age** (Years):<br>I : 47.62 $\pm$ 3.28	Cycling training with Functional electrical stimulation (FES-CG)	Cycling training without Functional electrical stimulation (CG)	Postural control (The Smart Balance Master system) and muscle tone (Hoffmann's reflex/motor response ratio, H/M ratio and pendulum test)	In the balance test, some parameters in all directions exhibited significant intervention effects between the two groups. Both groups showed significant improvement in the H/M ratio (FES-CG: $p = .014$, and CG ; $p = .005$) and relaxation index (FES-CG : $p = .005$; and CG: p = 047) revealed significant	Cycling training, with or without FES may reduce spasticity in stroke patients. The	1

			C= 51.64 ± 3.41<br><br>**PSD** (months):<br>I : 25.54 ± 12.95<br>C= 29.64 ± 10.36<br><br>**TOS**: ischemic/ hemorrhagic I : 4/6<br>C= 5/5				difference between FES-CG and CG group. The change ratios of directional control in the forward direction and H/M ratio revealed significant difference (p = .022) between FES-CG and CG among subjects with higher muscle tone. The stroke subjects' postural control was improved while their muscle tone was reduced after the 20 min cycling training program both with and without FES.	application of FES in cycling exercise was shown to be more effective in stroke patients with higher muscle tone.	
**108**	Solopova et al, 2011	Russia/Italy	**Sample size:** I = 32 C = 29<br>**Age Range:** I=NR C=NR<br>**Mean Age:** NR<br>**PSD** (months):<br>I = 8.2 ± 4.3<br>C = 9.3 ± 4.5<br>**TOS**: I = 27/5<br>C = 26/3	step-synchronized functional electrical stimulation (FES) with conventional therapy	Conventional therapy only	Motor function of lower limb with the Fugl-Meyer scale. Stroke severity (National Institutes of Health Stroke Scale (NIHSS) and European Stroke Scale (ESS)); ADLBarthel Index (BI))	After treatment, there was an improvement of the motor function and ADL in both groups, however, significantly higher in the experimental group. Active rhythmic. No significant between group difference was noted for severity	The developed FES and leg displacement-assisted therapy facilitates improvement in motor and functional abilities.	1
**109**	Knutson et al, 2012	USA	**Sample size:** I = 9 C = 8<br>**Age Range:** I=NR C=NR<br>**Mean Age** (Years):<br>I = 54.4 ± 13.5<br>C = 51.9 ± 7.9<br>**PSD** (days):<br>I= 95 ± 58<br>C= 109 ± 47<br>**TOS: ischemic/hemorrhagic**: I = 8/1<br>C = 7/1	6 weeks Contralaterally controlled functional electrical stimulation (CCFES)	6 weeks cyclic neuromuscular electrical stimulation (NMES)	maximum voluntary finger extension angle and finger movement tracking error (custom-built electrogoniometer); upper extremity function (Fugl-Meyer motor assessment (FMA); dexterity (the Box and Blocks Test (BBT)); motor functions (Arm Motor Abilities Test); Activity limitation (Functional Ability component (0 to 5) ordinal scale) of the	At all post-treatment, CCFES produced larger improvements than cyclic NMES on every outcome measure. Maximum voluntary finger extension showed the largest treatment effect, with a mean group difference across the posttreatment time points of 28° more finger extension for CCFES.	The results favor CCFES over cyclic NMES for the improvement in mtor functions	3

						Arm Motor Abilities Test (AMAT).			
110	Ambrosini et al, (2011)	Italy	**Sample size:** I= 15 C = 15 **Age Range:** NR **Mean Age:** I=59+10 C=56 +14 **PSD(days):** I= 48 + 43 C= 48 + 36 **TOS**(ischemic/hemorrhagic/ traumatic brain injury)**:** I = 11/3/1 C 8/5/2 =	cycling induced by functional electrical stimulation (FES)	passive cycling with placebo stimulation	Primary: Motricity Index (MI), for motor power and timing a walk of 50 meters with a stopwatch for walking speed<br><br>secondary: Trunk Control Test (TCT) for trunk Control and the Upright Motor Control Test (UMCT) for functional abilities	Both groups showed no significant differences between assessments after training and at follow-up. A main effect favoring FES-treated patients was demonstrated by repeated-measures ANCOVA for Motricity Index (*P*<0.001), Trunk Control Test (*P*=0.001), Upright Motor Control Test (*P*=0.005), and pedaling unbalance (*P*=0.038). No outcome measures demonstrated significant improvements after training in the placebo group.	The study demonstrated that 20 sessions of FES cycling training significantly improved lower extremity motor functions and accelerated the recovery of overground locomotion in postacute hemiparetic patients. Improvements were maintained at follow-up.	4
111	Embrey et al, 2010	USA	**Sample size: I=**15 C=13 **Age Range:** I=34 - 73 C= 41-75 **Mean Age:** I=62.1 + 11.6 C=57.5 + 10.0 **PSD(years):** I=4.3 + 3.4 C=5.6 + 4.4 **TOS:** NR	3 months of wearing functional electrical stimulation (FES) that activates automatically while walking then crossover to 3 months of walking without FES	3 months of walking without FES then cross over to 3 months of wearing functional electrical stimulation (FES) that	walking speed (6 minutes walk test[6MWT]); ambulation (Emory functional Ambulatory Profile [EFAP]), isometric strength (Dynamometer), spasticity (Modified Ashworth Scale [MAS]), and the impact of stroke (Stroke Impact Scale	Change in scores yielded higher scores for the intervention group in all the measured outcomes. There is a significant improvement in 6MWT for both interventions (p = 0.02). There is no significant difference between the groups in EFAP, same with SI; however, both groups measured significant improvement. There were no statistically significant change in plantar flexor strength in both groups while the control group has more significant improvement in strength of dorsi-flexor. MAS yielded	FES system that stimulates dorsiflexors and planter flexors similar to the timing of typical adult gait combined with daily walking, can	2

					activates automatically while walking	[SIS])	no statistically significant changes between the groups	improve the walking ability of adults with hemiplegia	
**112**	Yan et al, 2005	Hong Kong	**Sample size:**<br>I: G1 = 13<br>G2 = 15<br>C= 13<br>**Age Range:** NR<br>**Mean Age:**<br>I: G1 = 68.2+7.7<br>G2 = 73.3+8.1<br>C= 70.4+7.6<br>**PSD(days):**<br>I: G1 = 8.7+5.8<br>G2 = 10.1+2.8<br>C= 9.1+3.5<br>**TOS(ischemic/hemorrhagic):**<br>I: G1 = 11/2<br>G2 = 13/2<br>C= 11/2	G1 = standard rehabilitation with functional electrical stimulation (FES) or placebo stimulation or alone<br><br>G2 = standard rehabilitation with placebo functional electrical stimulation (placebo)	standard rehabilitation (SR)	ankle plantar-flexor tone (composite spasticity scale (CSS)); Maximum isometric voluntary contraction (MIVC) (joint torque and surface (EMG); Walking (timed "Up & Go" (TUG))	Raw CSS scores of the affected plantar-flexors in the 3 groups were similar at the different assessment intervals. However, the percentage increases of CSS scores in the placebo and control groups at week 3 were significantly greater than that in the FES group (*P*<0.05). In contrast, no difference was found between placebo and control groups at all times. Percentage increases in MIVC torques and IEMG of the FES group were significantly larger than those of the control group from week 1 onward (*P*<0.01), and larger than the placebo group at week 3 (*P*=0.032). In ankle plantar-flexion, a significant effect was found only at week 3 between the FES and the other 2 groups (*P*<0.01), Furthermore, the EMG cocontraction ratio during dorsiflexion of the affected ankle was significantly more reduced in the FES than the other 2 groups from week 1 or 2 onward (*P*=0.001 to 0.042) No differences were found in the TUG score among groups at any time	Fifteen sessions of FES, applied to subjects with acute stroke plus standard rehabilitation, improved their motor and walking ability to the degree that more subjects were able to return to home	3
**113**	Burridge, et al, 1997	UK	**Sample size:** I= 16<br>C= 16<br>**Age Range:** NR<br>**Mean Age:**<br>I= 52.3+14.3<br>C= 61.3 + 8.6<br><br>**PSD(years):**<br>I= 3.7<br>C= 4.11	functional electrical stimulation (FES): used the stimulator and received a course of physiotherapy	Physiotherapy alone.	Walking speed (10MWT); walking efficacy (The Physiological Cost Index (PCI)); heart rate (polar heart rate monitor)	The intervention group showed an increase in walking speed; No significant change was measured in walking parameters in the control group. There was also a statistically significant difference between the change in PCI between the groups (p = 0.048); no other between group change was noted.	Physiotherapy alone, in this group of subjects with established stroke, did not improve walking.	3

			**TOS(ischemic/hemorrhagic):**NR						

Key: PSD - poststroke duration; TOS. Quality score: assessed with Jadad quality assessment scale for rating randomized controlled trials; N/A = Not Available or Applicable PFMT = Pelvic Floor Muscle Training; LUTS = Lower Urinary Tract Symptoms; E = Experimental; C = Comparison/Control; I = Ischaemic Stroke; H = Haemorrhagic Stroke; ES = Electrical Stimulation; VDS = Videofluoroscopy Dysphagia Scale; PAS = Penetration–Aspiration Scale; CT = Conventional Treatment; NFT = Neuromuscular Faccilitation; BMD = Bone Mineral Density, ALP = Alkaline phosphatase; BGP = Bone gla protein; IL-6 = Interleukin-6; PNS = Peripheral Nerve Stimulation; mCIT = modified Constraint Induced Movement Therapy; WMFT = Wolf Motor Function Test; FMA = Fugl-Meyer Assessment Scale; ARAT = Action Reach ArmTest; UE = Upper Extremity; mAS = Modified Ashworth Scale; MBEST = Mini Balance Evaluation Systems Test, TUG = Time up and Go; 6MWT = Six minutes walk test;ABC =Activity Specific Balance; FAI = Frenchai Activity Index; CHIEF = Craig Hospital Inventory of Environmental Factors; SIS- = Stroke Impact Scale ; mCIMT = Modified Contraint Induced Movement Therapy; rTMS = Repetitive transcranial magnetic stimulation; BI = Barthel Index; HADS-Thai = Thai version of the Hospital Anxiety and Depression Scale; DEXA = Dual energy-ray Absorptometry; LWBV = Low intensity Whole Body Vibration; HWBV = High intensity Whole Body Vibration; MFT = Manual Function Test; VD = Vertical Displacement (Shoulder Subluxation); UL = Upper Limb; MAL = Motor Activity Log; VO2 peak, 6MWD = 6-minute walk distance, 30WT = 30-ft Walk Times; 48SC = 48-hr step counts; TDx = Treadmill; SAM = Step Activity Monitoring; BCI-FES = Brain–Computer Interface controlled Functional Electrical Stimulation; MAL = Motor Activity Log; mBI = modified Berthel Index; AOT = Action Observational Training; SMA = Stride Management Assist; FTST = Functional Task Specific Training; m = Median;

Virtual Reality

	Study (Authors, year	Country	Participants	Interventions / Model of care	Control/ comparison	Outcomes assessed/ outcome measures	Results	Conclusion	Quality score
**114**	Choi and Paik 2018	South Korea	24 patients<br>**Age range:**<br>Experimental group=61.0±15.2<br>Control group= 72.1±9.9<br>**PSD:**<br>Experimental=15.75 ± 11.35 (3–47)<br>Control=14.75 ± 9.59 (8–44)<br>TOS= ischaemic stroke	Game-based upper extremity virtual reality program for patients with stroke (MoU-Rehab)	Conventional therapy	-Fugl-Meyer Assessment of the upper extremity (FMA-UE) was used to evaluate arm and hand function and activities<br>-Brunnström stage (Bstage) was used to evaluate the upper extremity recovery stages	FMA-UE , experimental=24.5 ± 22.2 (4–63), control= 21.5 ± 20.6 (4–57) , p-value=0.735<br><br>Brunnstrom stage (arm) , experimental=2.7 ± 1.5 (1–5) , control=2.7 ± 1.5 (1–5) , p-value=1.00	MoU-Rehab was not inferior to the conventional therapy that is delivered one-on-one by an occupational therapist.	1
**115**	Aşkın et al. (2018)	Turkey	-Forty stroke patients<br>-Age range:<br>Experimental group= 53.27 ± 11.19yrs<br>Control group=56.55 ± 9.85yrs<br>-**PSD**= after 6 months<br>**TOS** n (%): (ischemic/hemorrhagic),<br>Experimental group=16/2 (89/11)<br>Control group=19/1 (95/5)	physical therapy (PT) + Kinect-based virtual reality (VR) training	Physical training	-Fugl-Meyer Assessment (FMA) was used to measure movement, coordination, and reflex action about the shoulder, elbow, forearm, wrist, hand, hip, etc.<br>-Brunnstrom Recovery Stages (BRS)<br>-Modified Ashworth Scale (MAS used for the primary clinical measure of muscle spasticity<br>-Box and Block test (BBT) was used to	Differences from baseline of FMA, MI, and AROM<br>(except adduction of shoulder and extension of elbow) were greater in the experimental group ($p < 0.05$). Others were not significant.	The adjunct use of Kinect-based VR training may contribute to the improvement of UE motor function and AROM in chronic stroke patients	3

						measure unilateral gross manual dexterity.<br>-Motricity index (MI) provided a brief assessment of the motor function of the arm and total arm score is obtained by evaluation of pinch grip<br>Range of motion (AROM) measurement			
**116**	Calabrò et al, (2017)	Italy	24 patients<br>**Age** range:<br>RAGT + VR= 60 ± 4yrs<br>Lokomat= 63 ± 6yrs<br>**PSD:**<br>RAGT + VR= 8 ± 2months<br>Lokomat= 8 ± 2months<br>**TOS**= ischaemic	Lokomat with VR (RAGT + VR)	Lokomat	Clinical, kinematic, and EEG<br><br>[N/S]	There was a significant correlation between RMI and POM A score improvement and central β -ERS (r = 0.895,<br>p = 0.001 and r = 0.570, p = 0.04, respectively) and frontal<br>Hγ-ERD magnitude (r = −0.831, p = 0.003 and r = −0.615,<br>p = 0.04, respectively)	VR feedback during RAGT elicits stronger cortical activations within the fronto-parietooccipital areas potentially belonging to the<br>MNS, and involved in motor intention and planning.	4
117	Faria et al, (2016)	Portugal	18 patients<br>Age range (years):<br>Experimental group=58 (48–71)<br>Control group= 53 (50.5–65.5)<br>PSD(months):<br>Experimental group=7 (4–49)<br>Control group=4	Virtual Reality-based simulation using Reh@City	Time-matched cognitive training	-Addenbrooke Cognitive Examination to assess the global cognitive functioning<br>-Trail Making Test A and B was used to assess attention<br><br>Picture Arrangement from WAIS III was	The experimental group improved, significantly more than the control group, in terms of general cognitive functioning, as assessed by ACE (U = 13.500, Z = −2.388, p = .014,<br>r = .56) and MMSE (U = 18.000, Z = −1.996, p = .050,<br>r = .47). The experimental group presented also significantly higher scores in the attention domain (U =	Cognitive rehabilitation through the Reh@City, an ecologically valid VR system for the training of ADL's,	**4**

			(3–11.5)<br>TOS= both ischaemic and haemorrhagic [N/S]			used to assess executive functions<br>-Stroke Impact Scale 3.0 was used to assess the subjective general health status	17.000, Z = −2.066, p = .040, r = .49).	has more impact than conventional methods.	
118	In et al, (2016)	South Korea	Twenty-five patients<br>**Age** range(year) :<br>Experimental group=57.31±10.53<br>Control group=54.42±11.44<br>**PSD** (month):<br>Experimental group=12.54±4.14<br>Control group=13.58±5.28<br>**TOS** (ischaemic /haemorrhagic):<br>Experimental group=8/5<br>Control group=8/4	Virtual reality reflection therapy (VRRT) plus conventional rehabilitation program (Rhb)	Placebo VRRT program plus conventional rehabilitation program	Berg Balance Scale (BBS), The Functional Reaching Test (FRT)<br><br>The Timed Up and Go (TUG) test were all used to compare dynamic balance ability before and after the intervention<br><br>10 meter walking velocity (10 mWV) was used to assess gait ability and velocity	FRT, TUG, and 10 m WV showed signifiant improvements compared to baseline in the VRRT group (p<0.05), but not in the control group	VRRT + Rhb program for patients with chronic stroke might be even more benefiial than Rhb alone in improving affected lower limb function.	**4**
**119**	Lee et al, (2016)	Korea	Eighteen participants<br>**Age** range, year:<br>VRBT =69.20 (5.514)<br>BT = 73.13 (8.983)<br>**PSD**, days:<br>VRBT =16.20 (6.477)<br>BT = 17.00 (6.437)<br>**TOS:** (ischaemic/ haemorrhagic)<br>VRBT = 5/3<br>BT =4/4	Virtual reality-based bilateral upper extremity training (VRBT)	Bilateral upper limb training (BT)	Upper extremity function was measured using the Jebsen–Taylor Hand Function Test (JHFT), the Box and Block test (BBT)<br>and the Grooved Pegboard Test (GPT).	VRBT group exhibited significant improvements in upper extremity function and muscle strength (p < 0.05) after the 6-week training programme	VRBT is a feasible and beneficial means of improving upper extremity function and muscle strength in individuals following stroke	4
**120**	Choi et al. (201	South Kor	24 patients<br>**Age** range (years):<br>Experimental	Mobile upper extremity rehabilitation	Conventional occupatio	Fugl−Meyer<br>-FMA-UE (arm and hand function and	FMA-UE: 24.5 ± 22.2 (4–63), 21.5 ± 20.6 (4–57) p=0.735	Mobile game-based VR	4

	6)	ea	group= 61.0 ± 15.2<br>Control group= 72.1 ± 9.9<br>**PSD**,months: Experimental group=15.75 ± 11.35<br>Control group=14.75 ± 9.59<br>**TOS**= ischaemic stroke	program using a smartphone and a tablet PC (MoU-Rehab)	nal therapy (OT)	activities)<br>-Brunnstrom stage [B-stage] (arm and the hand, manual muscle testing¨ [MMT])<br>Modified Barthel index [mBI] (activity limitations)<br>-EuroQol-5 Dimension [EQ-5D] participant restriction and QoL<br>-Beck Depression Inventory [BDI]) psychology test	Brunnstrom stage (arm) 2.7 ± 1.5 (1–5), 2.7 ± 1.5 (1–5)p= 1.00<br>Brunnstrom stage (hand) 1.9 ± 1.4 (1–5), 2.1 ± 1.4 (1–4) p=0.775<br>MMT (shoulder) 2.7 ± 1.1 (1–4), 2.2 ± 1.2 (0–4) p=0.292<br>MMT (elbow) 1.9 ± 1.4 (1–5). 2.1 ± 1.4 (1–4)p= 0.775<br>MMT (wrist) 2.7 ± 1.1 (1–4), 2.2 ± 1.2 (0–4)p= 0.292	rehabilitation program appears to be feasible and effective for promoting upper limb recovery after ischemic stroke.	
**121**	Kong et al, (2016)	Singapore	105 subjects<br>**Age** range, yr:<br>E1=58.1 ± 9.1<br>E2=59.0 ± 13.6<br>C=55.8 ± 11.5<br>**PSD** days:<br>E1= 14.2 ± 8.9<br>E2=14.2 ± 9.4<br>C=13.1 ± 8.6<br>**TOS**(Ischaemic/haemorrhagic):<br>E1= 25/8<br>E2= 28/7<br>C=27/8	Commercial gaming device, Nintendo wii (NW)<br><br>Conventional therapy	Occupational therapy	-Fugl-Meyer assessment (FMA) to measure upper limb impairment<br>-Action Research Arm Test measures arm and hand function<br>-Functional Independence Measure to measure ability to perform selfcare activities<br>-Stroke Impact Scale evaluates use of the affected upper limb in activities of daily living	Subjects with significant upper limb pain had lower FMA scores than those without pain at week 7 and 15. (26.1 ± 18.1vs. 38.4 ± 20.4, p = 0.015 at week 7, 29.8 ± 16.9 vs.40.8 ± 20.3, p = 0.001 at week 15).	12 week Augmented upper limb exercises via NW gaming or conventional therapy over a 3-week period was not effective in enhancing upper limb motor recovery compared to control.	3
**122**	Cho et al, 2015	Korea	P:chronic stroke patients , Sample size=22 (E=11, C=11),<br>**Mean age**=(E=60, C=58);<br>**PSD**: E=6months, C=263days;	virtual reality training with cognitive load (VRTCL) + Standard rehabilitation	VRTT, (virtual reality treadmill training) + Standard rehabilitat	GAITRite walkway System (Walking function)	-No significant differences in baseline values were observed between the experimental and control groups.<br>-<br>- in the dual task condition, greater improvement on walking function was observed in the VRTCL	Beneficial effect of VRTCL on walking function under single and dual task conditions in	3

			**TOS**(ischemic/hemorrhagic):<br>E = 9/2;C =6/5		ion		group, compared with the control group (P < 0.05)	chronic stroke patients was demonstrated	
**123**	McEwen, et al, 2014	Canada	**Sample size:** I= 30 C= 29<br>**Age Range:** NR<br>**Mean Age:**<br>I=62.2±14.1<br>C= 66.0±15.8<br>**PSD(days):**<br>I=30.1±18.9<br>C=39.6±17.8<br>**TOS(ischemic/hemorrhagic):**<br>I = 23/7<br>C = 25/4	Standard stroke rehabilitation therapy (SRhb) + a program of virtual reality (VR) exercises that challenged balance (eg, soccer goaltending, snowboarding) performed while standing	SRhb + exposure to identical VR environments but whose games did not challenge balance (performed in sitting)	Primary: balance (Timed Up and Go test (TUG));<br><br>Secondary: mobility (Two-Minute Walk Test (TMWT)) and lower extremity impairment (the Chedoke McMaster Stroke Assessment Scale Leg domain)	Both groups showed significant improvement in the TUG and the TMWT with the intervention group showing higher improvement. More individuals in the treatment than the control group showed improvements on the Chedoke McMaster Stroke Assessment Scale Leg domain at post intervention (*P*=0.04) and 1 month follow-up (*P*=0.02).	This VR exercise intervention that challenges balance for inpatient stroke rehabilitation improved mobility-related outcomes	3
**124**	Cho et al, 2012	Korea	**Sample size:** I=11 C=11<br>**Age Range:** I=NR C=NR<br>**Mean Age**:<br>I= 65.26±8.35<br>C=63.13±6.87<br>**PSD** (months):<br>I=12.54±2.58<br>C=12.63±2.54<br>**TOS: (**Ischemic/ hemorrhagic):<br>I= 7/4; C = 6/5	Virtual reality balance training (VRBT) + physical and occupation therapy (PT/OT)	Physical and occupational therapy (PT/OT)	Static balance [SB] (postural sway velocity),<br><br>Dynamic balance [DB] (Berg balance scale (BBS) and Time Up and Go Test (TUG))	There was a significant within group changes in both groups in the two measures of dynamic balance (TUG & BBS), however, the degrees of changes in the both outcomes were higher in the intervention group. There was no significant change both within groups and between groups in static balance	Both VRBT+ PT/OT and PT/OT alone has a signify-cant improving effect on DB. VRBT provides a higher effect on DB. None of the interventions has a significant effect on SB	2
125	Subramanian,	Canada	**Sample size:** I=16 C=16	Neurorehabilitation therapy in a virtual	Neurorehabilitation therapy in	Primary outcome:<br>-Motor functions (FMA) and	For the intervention, participants in the moderate to severe group increased elbow extension during a reach-to-grasp	VE training led to more changes in	2

	et al., 2013		**Age Range:** NR<br><br>**Mean Age:**<br>I = 60.0 ±11.0<br>C = 62.0 ±9.7<br><br>**PSD:**<br>I = 3.0 ±1.9<br>C = 3.7 ±2.2<br><br>**TOS: Ischemic/hemorrhagic:**<br>I = 10/6 C = 12/4	environment (VE) Group subdivided into mild and moderate-severe impairment	a physical environment (PE) Group subdivided into mild and moderate-severe impairment	Reaching Performance Scale for Stroke (RPSS)<br>-Arm motor activity (Wolf Motor Function Test-Functional Assessment Scale (WMFT-FAS)).<br>-ADL (The Motor Activity Log [MAL-AS])<br>-Motivation levels (the 13-item self-report Intrinsic Motivation Task Evaluation Questionnaire)	task (RPSS elbow close target: $P < .02$, far target: $P < .05$) whilie the mild group, extension range increased only in VE ($P < .01$). There was no other statistically significant result.	the mild group and a motor recovery pattern in the moderate-to-severe group indicative of less compensation, possibly because of a better use of feedback.	
**126**	Kiper et al, 2011	Poland, Italy	**Sample size:** I : 40 C = 40<br>**Age Range:** I=NR C=NR<br>**Mean Age** (Years): 64.0 ± 16.4<br>**PSD**: NR<br>**TOS**: ischemic/ hemorrhagic<br>I= 24/16 C= 24/16	Reinforced feedback in virtual environment (RFVE) therapy and Traditional neuromotor rehabilitation (TNR)	Traditional neuromotor rehabilitation (TNR) only	-Motor deficit and the functional activities of the upper extremity (Fugl-Meyer scale for the upper extremity (FMA-UE));<br>-spasticity (modified Ashworth scale [mAS]);<br>-Functional assessment (Functional Independence Measure (FIM)).	A statistically significant difference between RFVE and TNR groups was observed in the clinical outcomes of FMA-UE and FIM (both $p < 0.001$), but not Ashworth ($p = 0.053$). while no statistical significant difference in mean mAS was seen between the groups.	The RFVE therapy in combination with TNR showed better improvements in functional abilities compared to the TNR treatment only.	2
**127**	Yang et al, 2011	Taiwan,	**Sample size:** I = 7 C= 7<br>**Age Range:** NR<br>**Mean Age** (Years):<br>I = 56.3 ± 10.2<br>C= 65.7 ± 5.9<br>**PSD** (month):<br>I = 17.0 ± 8.6<br>C= 16.3 ± 10.4<br>**TOS**: NR	Virtual reality (VR-TDx) treadmill training	Traditional treadmill training (T-TDx).	-Static standing balance skills [Center of pressure (COP)],<br>-Gait parameters (pressure mat system)	-No significant improvements in COP during the quiet stance, either in the VR-TDx or T-TDx ($P > 0.05$), but there was a significant between group difference after training in COP maximum sway in medial-lateral direction during the quiet stance ($P = 0.038$).<br>-T-TDx failed to improve sit-to-stand performance, whereas VR-TDx improved symmetric index ($P = 0.028$) and sway	Neither T-TDx nor VR-TDx had any effect on balance skill during quiet stance, but VR-TDx improved balance skill	1

							excursion (P = 0.046) significantly during sit-to-stand transfer. The changes of symmetric index between groups were markedly different (P = 0.045).<br>- Both groups improved significantly in stance time, but only VR-TDx increased contact (P = 0.034) after training during level walking. The difference between groups during level walking was not significant.	in the medial-lateral direction better than T-TDx. VR-TDx also improved balance skill during sit-to-stand transfers	
**128**	Kim, et al, 2009	Korea	**Sample size:** I = 16 C= 17<br>**Age Range:** I=NR C=NR<br>**Mean Age** (Years):<br>I = 52.42 + 10.09<br>C= 51.75 + 7.09<br>**PSD** (days):<br>I = 25.91 + 9.96<br>C = 24.25 + 8.87<br>**TOS(ischemic/ hemorrhagic)**: NR	Conventional therapy with additional 30 mins of virtual reality therapy (CT+VR)	Conventional therapy only (CT)	-Balance (Balance Performance Monitor [BPM] and Berg Balance Scale [BBS] tests).<br>-Gait performance (10-m walking test and Modified Motor Assessment Scale [mMAS]), and<br>-Spatiotemporal parameters (GAITRite)	-There was a significant mean difference between the groups favouring the intervention group in BBS, 10 meters walk test and mMAS (P= 0.05).<br>-For gait parameters, a significant mean difference was found between the groups in cadence (P = 0.010) , step time (P = 0.012) and step length (P = 0.013) all favouring the intervention group	VR has an augmented effect on balance and associated locomotor recovery in adults with hemiparetic stroke when added to CT	4
**129**	Broeren et al, 2008	Norway	**Sample size:** I=11 C=11<br>**Age Range:**<br>I=32.5-78.3<br>C= 26.5-77.3<br>**Mean Age:**<br>I=67.0 8+ 12.5<br>C=68.0 8 + 1.5<br>**PSD(months):**<br>I=62.3 8+ 28.4<br>C=72.0 8+35.9<br>**TOS(ischemic/others):** I = 10/1;C = 9/2	Virtual reality training (VR)	Conventional therapy (CT)	-Subject's perception of the VR system (Semi-structured interview);<br>-Gross movements of the hand/arm (box and block test [BBT]);<br>-Participants' experience of problems in performing ADL (ABILHAND);<br>-Kinematics (upper extremity test [reaching])	-Significant improvement was shown in BBT but not in ABILHAND in the intervention group but none in the control group.<br>-For kinematics: movement time (seconds) improved considerably (p < 0.05) in the intervention group but not in the controls. HPR improved in the intervention group, but there were no differences between before and after training for the intervention and control groups with respect to max velocity) and max acceleration	Virtual reality shows better improvement in motor function compared to conventional therapy in stroke patients	2
**130**	You	Rep	**Sample size:** I= 5	Virtual reality	No	-locomotor function	VR practice produced a greater increase	VR could	2

	et al, 2005	ublic of Korea	C= 5<br>**Mean Age:**<br>I= 54.60±3.01<br>C= 54.60±3.44<br>**PSD:**<br>I= 18.20±2.27<br>C= 19.40±4.27<br>**TOS(ischemic/hemorrhagic):**NR	(VR), where patient were provided with interactive real-life practice environments	intervention	(FAC and walking item of mMAS).<br>-Laterality index (LI) in the regions of interests (ROIs) using fMRI and<br>-locomotor recovery (Standardized Locomotor Tests)	in LI than the control group. (*P*<0.05). However, the interval changes in the other ROIs were not significantly different (*P*>0.05). Motor function was significantly improved after VR (*P*<0.05).	improve motor function and enhanced cortical reorganization in patients with chronic stroke.	

Video Games

Ref	Study (Authors, year	Country	Participants	Interventions/ Model of care	Control/ comparison	Outcomes assessed/ outcome measures	Results	Conclusion	Quality score
**131**	Rand et al, (2018)	Israel	8 stroke patients (number of cases and control N/S)<br>**Age** range: 18 to 80yrs<br>**PSD**= at least 6 months<br>**TOS**= both ischaemic =6 and haemorrhagic=2	Video Game intervention	Traditional intervention	Semistructured interviews on:<br><br>Whole - body stimulation<br><br>Improving balance and gait.	N/A no statistics supplied	Playing video games was perceived not as treatment but as a motivating tool to facilitate whole-body movement. Therefore, this intervention might be suitable to be used in the community for ongoing intervention.	2
**132**	Dalal et al, (2018)	India	32 subjects<br>**Age** range: 40-80 yrs for both groups<br>**PSD**: N/A<br>**TOS**= both	Prowling along with proprioceptive training	Routine physiotherapy	Wisconsin gait scale (WGS) for spatio-temporal gait parameters	The experimental group had significant improvement for mean knee hyperextension (p= 0.001), ankle dorsiflexion (p=0.005), WGS score (p=0.001)	Prowling along with proprioceptive training is effective in reducing knee hyperextension, increasing	2

			ischaemic and haemorrhagic; [N/S]	plus routine physiotherapy				dorsiflexion range and improving spatio-temporal gait parameters.	
133	Emmerson et al, (2017)	Australia	62 participants<br>**Age** (years),:<br>E=68±15<br>C=63±18<br>**PSD**(days), median (IQR):<br>E=122(77–193)<br>C=133(58-228)<br>**TOS**(ischaemic/haemorrhagic):E=22/6; C=27/3	Home exercise programme filmed on an electronic tablet, with an automated reminder	Paper-based home exercise programme	Wolf Motor Function Test (WMFT)used as an index of upper limb motor ability, measured through a series of 15 timed functional tasks, and two strength based tasks	-There were no differences between the groups for measures of adherence (MD 2%, 95% CI −12 to 17) or change in the WMFT log transformed time (MD 0.02 seconds, 95% CI −0.1 to 0.1).<br>-There were no between-group differences in how participants found instructions (p = 0.452), whether they remembered to do their exercises (p = 0.485), or whether they enjoyed doing their exercises (p = 0.864).	The use of smart technology was not superior to standard paper-based home exercise programmes for patients recovering from stroke.	**3**

Body Weight Supported Treadmill Training (BWSTT)

**Ref**	**Study (Authors, year**	**Country**	**Participants**	**Interventions/ Model of care**	**Control/ comparison**	**Outcomes assessed/ outcome measures**	**Results**	**Conclusion**	**Quality score**
134	Gama et al,. (2017)	Brazil	28 participants<br>**Age** range: 58.2±9.1 years<br>**PSD**(months)<br>E=60.2±55.4<br>C=53.8±42.2<br>TOS: (ischaemic / haemorrhagic)<br>E =12/2<br>C=10/4	E: Partial body weight support systems (BWS) on a tread mill	C: Overground training (OGG)	6 minutes-walk test<br>Motor domain of FIM<br>Lower extremity domain of Fugl-Meyer movement assessment<br>Step length symmetry ratio and single limb support duration<br><br>*[uses were not ststed]*	Only participants from OGG improved step-288 length symmetry ratio (p<.01, d=1.08), and both groups increased single limb support 289 duration for the paretic limb only (p=.006, BWS, d=.14, OGG, d=.79)	Only overground training led to significant improvements in step length s	**4**
**1**	Sriva	Ind	45 patients	E1:Bodyweigh	C:Overgro	Scandinavian Stroke	In the outcome measures, there was	BWSTT	3

35	stava et al, (2016)	ia	**Age** range (years):<br>E1=44.40±12.31<br>E2=47.93± 9.95<br>C=44.20± 11.70<br>**PSD**, (days):<br>E1= 652.20± 579.04;<br>E2=442.07± 295.13<br>C=391.80± 431.10<br>TOS (ischaemic/ haemorrhagic)<br>E1=13/02, E2 =11/04<br>C =12/03	t-supported treadmill training (BWSTT)<br><br>E2:Treadmill training without bodyweight support	und gait training	Scale (SSS) was used to measure impairment after stroke.<br><br>Functional Ambulation Category (FAC) was used to classify walking handicap after stroke.	significant improvement (P < 0.05) in the 3 groups at the end of training, which was sustained at 3-month follow-up (other than walking endurance in group I). Outcomes were better with<br>BWSTT but not significantly (P > 0.05).	offers improvement in gait but has no significant advantage over conventional gait training strategies for chronic stroke survivors.	
136	MacKay-Lyons et al, 2013	Canada	**Sample size:** I= 24<br>C= 26<br>**Age Range:**<br>**I=** 29-88<br>C= 41-78<br>**Mean Age: I=** 61.5 ± 15.4<br>C= 59.0 ± 12.7<br>**PSD(days):**<br>I= 23.3 ± 5.7<br>C=23.1 ± 4.4<br>**TOS:** NR	Body-weight-supported treadmill training (BWSTT)	Usual care (UC)	Primary: Cardiovascular Fitness (CVF): VO_{peak} (open-circuit spirometry); Walking function (6MWT)<br><br>Secondary: functional balance (BBS); motor impairment (ChedokeMcMaster Stages of Recovery (CMSR) Leg and Foot)	There was significant Group × Time interactions for VO_{peak} (*P* = .004). The intervention group showed significant improvement in VO_{peak} at posttraining, 6-month follow-up, and 12-month follow-up (*P* < .001). There was no significant Group × Time interaction for walking function; however, within-group analyses revealed that both groups improved from baseline across tests (*P* < .001). Group × Time interactions were not significant for BBS and CMSR Leg. Both roups also showed significant improvement in BBS and CMSR	BWSTT elicits greater improvements in CVF and walking endurance than UC in the subacute poststroke period. These gains are largely sustained for 1 year	3
137	Nadeau et al., 2013	USA	**Sample size:**<br>I:G1 = 139<br>G2=126 C=143<br>**Age Range:** I=NR<br>C= NR<br>**Mean Age:**<br>I:G1= 60.1 ± 12.3<br>G2=62.6 ± 13.3<br>C=63.3 ± 12.5<br>**PSD(days):**<br>I:G1 = 64.1 ± 8.3<br>G2=62.9 ± 8.0<br>C=64.2 ± 9.0<br>**TOS:**	G1 = Usual care plus 36 therapist-provided sessions of walking training on a treadmill using body-weight support and practice overground at clinics (locomotor	Usual care (UC)	Primary; The proportion of participants who improved their functional level of walking at 6 months poststroke.<br><br>Secondary: walking speed, distance walked in 6 minutes, and number of steps taken per day measured with a step activity monitor.	compared with UC a significantly higher functional walking level was achieved using LTP (*P* = .010) and HEP (*P* = .007). There was no<br>difference in gains between LTP and HEP, all 3 groups improved their walking speed, 6-minute walk distance, and number of community steps taken per day (*P*< .0001). However, the intervention groups had greater improvement in walking speed than the control group (*P*< .0001). All 3 groups also significantly improved in ADL/IADL, physical mobility and social participation participation, motor recovery (FM),	Progressive physical therapy using either walking training on a treadmill and overground, conducted in a clinic, or strength and balance exercises conducted at	3

			**ischemic/hemorrhagic : NR**	training program [LTP])<br><br>G2 = Usual care plus 36 therapist-provided sessions of impairment-based strength and balance exercise at home (home exercise program [HEP]		Motor impairment (Fugl-Meyer assessment of lower-extremity); Balance (Berg Balance Scale (BBS) and Activities-Specific Balance Confidence (ABC) Scale); ADL the ADL/Instrumental ADL (IADL) Scale); overall disability (physical mobility and participation domains of the Stroke Impact Scale, and the modified Rankin Scale)	balance score (BBS), balance confidence (ABC Scale), and modified Rankin score (*P* < .0001). With Bonferroni adjustment for multiple testing, the LTP and HEP groups improved more than the UC group in BBS score, ABC Scale score, and physical mobility (*P* < .0014). In addition, there were trends toward more improvement in the LTP and HEP groups, relative to the UC group, in community steps taken, ADL/IADL, social participation, motor recovery (FM-LE), and modified Rankin scale.	home, was superior to usual care in improving walking in post stroke patients	
**138**	Høyer, et al, 2012	Norway	**Sample size:** I = 30 C = 30<br>**Age Range:**<br>I=24 – 68<br>C=18 – 69<br>**Mean Age** (Years):<br>I = 52.3 + 10.4<br>C = 52.0 + 13.1<br>**PSD** (days):<br>I= 96 + 42.0<br>C= 99 + 39.4<br>**TOS ischemic/hemorrhagic**: I = 15/15<br>C = 17/13	30 sessions of Treadmill training with body weight support (TTBWS) plus traditional training	Traditional training alone	Primary: Walking ability (Functional Ambulation Categories (FAC 0-5)).<br>Secondary: walking speed (10 m walk test); waking endurance (6 minutes walk test (6MWT)), ability to walk over ground after TTBWS (EU Walking (0–5)), transfer ability (Functional Independence Measure (FIM 1-7))	Substantial improvements in walking and transfer were shown within both groups after 5 and 11 weeks of intervention. Overall, no statistical significant differences were found between the groups in any of the measured outcomes, but 12 of 17 physical measures tended to show improvements in favour of the treadmill approach.	Both training strategies provided significant improvements in the tested activities, suggesting that similar outcomes can be obtained in the two modalities by systematic, intensive and goal directed training	3
**139**	Duncan et al., (2011)	USA	**Sample size:**<br>I: G1= 139<br>G2 =143<br>C= 126<br>**Mean Age:**<br>G1= 60.1±12.3	G1 = training on a treadmill with the use of body-weight support 2 months after	Exercise program at home managed by a physical	Walking speed (10MWT & 6MWT); Motor Recovery in legs (FMA); Balance (the Berg Balance Scale); Activities-Specific	All the groups showed significant improvement in all the measured outcomes with no significant difference in any of the outcomes reported	Locomotor training, including the use of body-weight support in	2

			G2 = 63.3±12.5<br>C= 62.6±13.3<br>**PSD(days):**<br>G1= 64.1±8.3<br>G2 = 64.18±9.0<br>C = 62.9±8.0<br>**TOS:** Large vessel: G1=55; G2=60; C=47<br>Lacunae:G1=40; G2=45; C =43<br>Hemorrhage G1=27;G2=22;C=21<br>Undefined G1=17; G2=16;C = 15	the stroke<br><br>G2 = training on a treadmill with the use of body-weight support 6 months after the stroke	therapist 2 months after the stroke	Balance ADL(Confidence Scale, the Activities of Daily Living- Instrumental activities of Daily Living (ADL–IADL) Scale); functional ability and quality of life (the physical mobility and participation domains of the Stroke Impact Scale).		stepping on a treadmill, was not shown to be superior to progressive exercise at home managed by a physical therapist.	
140	Daly et al, (2011)	USA	**Sample size: I=** 20 C= 24<br><br>**Age Range:** NR<br><br>**Mean Age: I=** 59 C= 62<br><br>**PSD(months):** NR<br><br>**TOS(ischemic/hemorrhagic):** I = 20/0 C = 19/5	coordination exercises, body weight–supported treadmill training (BWSTT), and over-ground walking, provided with functional electrical stimulation (FES)with intramuscular (IM) electrodes (FES-IM)	coordination exercises, body weight–supported treadmill training (BWSTT), and over-ground walking, without FES	Primary outcome: gait parameters (Gait Assessment and Intervention Tool (G.A.I.T.) of coordinated movement components);<br>Secondary outcome: leg muscle strength (manual muscle testing [MMT] on a 5-point scale); isolated leg joint movement cordination (Fugl-Meyer scale); walking endurance (6-Minute Walk Test); ADL (Functional Independence Measure subscales for Locomotion and Mobility (FIM-L&M))	The G.A.I.T. showed an additive advantage with FES-IM versus No-FES (*P* = .045) at the end of training. Both groups showed a significant gain in all the measured outcomes (*P* < .05), and a continued benefit from mid- to posttreatment (*P* < .05) was present. For FES-IM, recovered coordinated gait persisted at 6-month followup but not for No-FES. A significant between group difference in mean gait paramenters were found between the groups; no other between group changes were found in the measured outcomes.	Improved gait coordination and function were produced by the multimodal Gait Training Protocol. FES-IM added significant gains that were maintained for 6 months after the completion of training.	3
141	Dean et al, 2010	Australia	**Sample size: I**=64 C=62<br><br>**Age Range:** NR	Treadmill walking with body weight support via an overhead	Overground walking	PO: Quality of walking: speed and stride length (10 meter walk test). Waking capacity (6mintues walk test),	In terms of walking quality and capacity, the experimental group walked with a mean speed that was 0.10m/s faster than the control, but there was no statistically significant difference between the two	Treadmill walking with body weight support provides post	2

			**Mean Age: I=**70+ 9<br>C=71+ 9<br><br>**PSD(days):**<br>I=18+ 8<br>C=18+ 7<br><br>**TOS:** NR<br>NR	harness		walking perception(numerical scale[1-10])),<br>SO: falls(quantified) and community participation (Adelaide Activity Profile [AAP])	groups. A significant difference was found in walking capacity between the groups favouring the intervention group. At 6 months, the experimental group rated their walking 1 out of 10 points higher than the control group. However, both groups scored low on the AAP with no significant difference. Although more (10%) of the experimental group fell, on the average, they had 0.1 few falls than the control group, neither which are statistically significant.	stroke patients with a better walking capacity and perception of walking but no effect on walking quality	
**142**	Ada, et al, 2010	Australia	**Sample size: I=**64<br>C=62<br>**Age Range:** NR<br>**Mean Age:**<br>I=70 + 9<br>C=71 + 9<br>**PSD(days):**<br>I=18 + 8<br>C=18+7<br>**TOS:** NR	treadmill walking with body weight support via an overhead harness (TBWS)	Assisted overground walking.	Proportion of participants walking independently within 6 months.	43 of 60 experimental participants achieved independent walking compared with 36 of 60 control participants. experimental group walked 2 weeks earlier, with a median time of 5 weeks compared to 7 weeks for the control group.	TBWS is feasible, safe, and tends to result in more people walking independently and earlier after stroke.	2
**143**	Franceschin, et al, 2009	Italy	**Sample size:** I=52<br>C=45<br><br>**Age Range:**<br>I=43.6–79<br>C= 53.8–84<br><br>**Mean Age:** I=65.5+ 12.2<br>C=70.9 + 11.8<br><br>**PSD(days):**<br>I=28.9 + 12<br>C=26.1 + 10.9<br><br>**TOS:** I = 39/13<br>C = 36/9	conventional therapy with 20 minutes of gait training on a treadmill with Body weight support system [BWS]	Conventional therapy only	Motor functions (Motricity Index at the paretic upper and lower extremity); Trunk mobility (Trunk Control Test); modified Rankin Scale; ADL (Barthel Index); Ambulatory capacity (Functional Ambulation Categories); spasticity (Mordified Ashworth scale [MAS]); oral comprehension (Token Test); unilateral spatial neglect (Albert test); proprioceptive sensibility at the lower	. Each group showed significant improvement from baseline to 2 weeks and6 months post intervention follow-up in all outcome variables; comparison between the 2 groups did not reveal any significant differences	adding 20 minutes of treadmill training with BWS to conventional overground gait training is not more effective than conventional treatment in the early phase after stroke	3

						limb (An ordinal scale); walking speed (10MWT); walking capacity (6MWTt); dyspnea (Borg Scale) & Walking Handicap Scale.			
**144**	Nilsson et al., 2001	Swedes	**Sample size:** I= 36 C= 37 **Age Range:** I= 24–67 C= 24–66 **Median Age:** I= 54 C= 56 **PSD(years):** I = 22 C = 17 **TOS(ischemic/hemorrhagic):**NR	walking training on a treadmill with body weight support (BWS)	walking training according to the Motor Relearning Programme (MRP) on the ground	ADL (FIM); locomotor function and control (Fugl-Meyer Scale [FMA]); ambulation (Fugl-Meyer Stroke Assessment); walking speed (10 MWT); balance (BBS); ambulatory ability (Functional Ambulatory Category).	Both the treatment group and the control group had improved significantly between admission and discharge with respect to FIM, Fugl-Meyer Stroke Assessment, FAC, walking speed and Berg's Balance Scale. The results of these improvement showed a trend favouring the intervention group but not significant	Treadmill training with BWS at an early stage of rehabilitation did not provide any between effect compared with walking training on the ground	3

Occupational Therapy Intervention

Ref	Study (Authors, year	Country	Participants	Interventions/ Model of care	Control/ comparison	Outcomes assessed/ outcome measures	Results	Conclusion	Quality score
**56**	Ilić et al, (2016)	Serbia	26 stroke patients **Age** range (years), mean (SD): Experimental	Anodal transcranial direct stimulation (tDCS) with occupational	Sham stimulation with occupational therapy	Modified Jebsen Taylor Hand Function Test (mJTHFT) was used for functional assessment that is scored by using the	mJTHFT showed significant differences between active and sham group i.e. main effect of group $F[1,23] = 18.978$, $p < 0.001$, $p2 = 0.452$,as well as the effect of covariate $F[1,23] = 88.191$, $p < 0.001$, $p2 = 0.793$;	Fine motor skill deficits in chronic stroke survivors can be improved	4

			group=58.3 (7.7)<br>Control group=62.0 (3.9)<br>PSD (months): Experimental group= 41.0 (24.4)<br>Control group= 37.3 (20.9) 0.68<br>TOS: ischaemic	therapy		summed times required to complete seven individual tasks.<br><br>Upper limb Fugl-Meyer (ULFM) [N/S]	and no within-subject effect of time F[2,46] = 0.709, p = 0.497, p2 = 0.030 i.e. between-group differences were preserved across time without change of slope	when intensive OT is primed with anodal tDCS over the ipsilesional hemisphere.	
**145**	Shin et al, 2014	Korea	**Sample size:** I= 9 C= 7<br><br>**Age Range:** NR<br><br>**Mean Age:**<br>I=52.0 ± 11.9<br>C= 46.6 ± 5.8<br>**PSD(days):**<br>I=67.1 ± 45.3<br>C=76.6 ± 28.5<br>**TOS(ischemic/ hemorrhagic):** NR	conventional occupational therapy plus 20 minutes of RehabMaster intervention (RehabMaster + OT group).	conventional occupational therapy only (OT-only group)	Primary: Fugl-Meyer Assessment score (FMA),<br>Secondary: Modified Barthel Index (MBI), MBI, Medical Research Council Score, and passive range of motion of the affected upper extremity.	The improvement in the FMA was greater in the RehabMaster + OT group than in the OT only group, although this trend did not reach statistical significance (P = .07). Although the improvement in the MBI did not differ significantly between the groups (P = .16), the change in the MBI was greater in the RehabMaster + OT group (11.6 ± 6.5) than in the OT-only group (7.7 ± 4.6). The Medical Research Council Score and the painless passive range of motion of the affected upper extremity did not differ significantly between the two groups.	RehabMaster intervention did not provide any statistically significant improvement in hand function and ADL	2
**81**	Abo et al., 2013	Japan	**Sample size:** I= 44 C=22<br><br>**Age Range:** NR<br><br>**Mean Age:**<br>I= 57·7 ± 12·7<br>C= 60·3 ± 10·6<br><br>**PSD(months):**<br>I= 62·1 ± 47·7<br>C=68·0 ± 53·1	Low-frequency repetitive transcranial magnetic stimulation Plus occupational therapy (LF-rTMS-OT)	Constraint induced movement therapy (CIMT)	Impairment (FMA); motor functions (WMFT)	FMA score increased significantly in both groups (LF-rTMS-OT: *P* < 0·001; CIMT: *P* < 0·005). Similarly, the decrease in WMFT log performance time was significant in both groups (LF-rTMS-OT, *P* < 0·001; CIMT, *P* < 0·005). In addition, FAS of WMFT also increase significantly in both groups (LF-rTMS-OT *P* < 0·001; CIMT, P < 0·005). A significantly larger increase in FAS of WMFT was significantly improved in the intervention group compared with the CIMT group (*P* < 0·05).	both intervention provided improved motor and functional outcomes, with Low-frequency repetitive transcranial magnetic stimulation	3

			**TOS(ischemic/ hemorrhagic):** I = 18/26 C =11/11					plus occupational therapy (LF-rTMS-OT) providing better effect	
**124**	Cho, and Song, 2012	Korea	**Sample size:** I=11 C=11 **Age Range:** I=NR C=NR **Mean Age**: I= 65.26±8.35 C=63.13±6.87 **PSD** (months): I=12.54±2.58 C=12.63±2.54 **TOS:** Ischemic/hemorrhagic: I= 7/4 C = 6/5	Virtual reality balance training (VRBT) with physical and occupation therapy	Physical and occupational therapy	PO: static balance (postural sway velocity), dynamic balance (Berg balance scale (BBS) and Time Up and Go Test (TUG))	There is a significant within group changes in both groups in the two measures of dynamic balance (TUG & BBS, however, the degrees of changes in the both outcomes were higher in the intervention group. There was no significant change both within groups and between groups in static balance	Both VRBT+PT/OT and PT/OT alone has a significant effect on the improvement of dynamic balance. VRBT provides a higher effect on dynamic balance. None of the interventions has a significant effect on static balance	2
**146**	Liang et al., 2012	Taiwan	**Sample size:** I=15, C=15 **Age Range:** **Mean Age**: I= 56.1±11.9 C=59.73±11.6 **PSD (Days):** I=10.9±5.4 C=13.6±6.4 **TOS:** Ischemic/hemorrhagic: I = 6/9 C = 7/8	Physical and occupational therapy plus 5 Thermal stimulations per week for 6 wks	Physical and occupation therapy with 3 consultations per week for 6 weeks	PO: level of impairment (FMA-LE), muscle strength (the (MRC-lower extremity) and gait ability (the Functional Ambulation Classification (FAC)), SO: Balance (Berg Balance Scale (BBS), motor functions (Modified Motor Assessment Scale (MMAS) and ADL	The result showed a significant difference between the groups in FMA-LE from the 4th wk lasting till the 12 months follow-up ($P<0.05$). Also the intervention group performed better in the MRC-LE, FAC and MMAS relative to the control group at the 4 and 6 weeks and 3 months follow-up ($P<0.05$) but effect disappeared at the 12th month. In addition, BBS and BI showed a significant between group difference favouring the intervention group at the 3 months follow-up (P = 0.038 and P = 0.031, respectively)	There is a long term benefit of thermal stimulation therapy for patients with acute stroke that may be sustained for 3 months but disappear by the 6 month and 1 year	3

						(Barthel Index (BI))		follow-up	
**147**	Nilsen, et al, 2012	New York	**Sample size:**<br>I: G1 =5<br>G2 = 6<br>C=6<br>**Age Range:** NR<br>**Mean Age**:<br>I: G = 46.6 ±5.2<br>G2 = 62.0 ±5.7<br>C= 66.2 ±2.6<br>**PSD** (month):<br>**I: G1=** 33.5 ±15.1<br>G2 =43.2 ±15.4<br>C = 20.3 ±8.8<br>**TOS:** NR	G1:Occupational therapy (OT) with mental practice (MP) training in functional tasks (FT) using aninternal Perspective<br>-G2 = OT with MP training in FT using an external perspective	Occupational therapy with relaxation imagery training	PO: impairment ((FMA-UE)<br>Hand function in ADL (Jebsen–Taylor Test of Hand Function [JTTHF]).<br>SO: self-perception of occupational performance and satisfaction with performance (The Canadian Occupational Performance Measure (COPM))	Mean FMA scores increased from pretest to posttest across all the groups, but significantly only for G1 and there was a group x time interaction (p = .042). G1 also has a higher improvement in the JTTHF compared to the other groups (p = .036). COPM scores increased significantly across all the groups (p < .001)	MP using either the internal or the external imagery perspectives, combined with OT, reduced impairments and improved function above a control condition.	3
**33**	Wu, et al., 2012	Taiwan	**Sample size:** I: G1=14 G2 = 14 C=14<br>**Age Range:** NR<br>**Mean Age** (Years):<br>I: G1= 55.13 ±12.72<br>G2 = 57.04 ±8.78<br>C=51.30 ±6.23<br>**PSD** (months):<br>I: G1=18.00±8.65<br>G2 = 17.29 ±13.29<br>C=17.57 ±9.80<br>**TOS:**Ischemic/hemorrhagic: NR	G1 = Occupational therapy with robot-assisted bilateral arm training(RBAT)<br><br>G2 = Occupational therapy with therapist-based bilateral arm training(TBAT)	Occupation and physical therapy	**PO:** motor impairment (The Upper Limb subscale of the Fugl-Mayer Assessment), daily functions (The Motor Activity Log [MAL]), quality of life (The Stroke Impact Scale [SIS] version3).<br>Kinematics: temporal efficiency and smoothness, trunk motion, trunk compensation, shoulder flexion (Kinematic analysis)	Large and significant effects were found in the kinematic variables, distal part of upper-limb motor impairment, and certain aspects of quality of life in favor of TBAT or RBAT. Specifically, the TBAT group demonstrated significantly better temporal efficiency and smoothness, straighter trunk motion, and less trunk compensation compared with the CT and RBAT groups. The RBAT group had increased shoulder flexion compared with the CT and TBAT groups. No group effect on the overall FMA score, proximal part score of the FMA, and AOU and QOM of the MAL was documented. However, on the FMA, the TBAT group showed higher distal part scores than the CT group. On the SIS, the RBAT group had better strength subscale, physical function domain, and total scores than the CT group.	Compared with CT, TBAT and RBAT exhibited differential effects on outcome measures. TBAT may improve temporal efficiency, smoothness, trunk control, and motor impairment of the distal upper limb. RBAT may improve shoulder	2

								flexion and QoL	
148	Lin et al, (2011)	China	**Sample size:** I= 19 C= 18 **Age Range:** I= NR C= NR **Mean Age:** I= 62.2 + 8.7 C= 66.0 + 9.6 **PSD(days)**: I= 43.5+ 25.2 C= 41.3 + 26.5 **TOS(ischemic/ hemorrhagic)**: I = 13/6 C = 12/6	neuromuscular electrical stimulation with standard treatment, including physical therapy and occupational therapy,	Standard treatment only, including physical therapy and occupational therapy,	The Modified Ashworth Scale for spasticity, the upper extremity section of the Fugl-Meyer motor assessment for upper limb function, and the Modified Barthel Index for ADL	Significant improvements were found in both groups in terms of Fugl-Meyer motor assessment, and Modified Ashworth Scale scores after the 3rd week of treatment. The significant improvements persisted 1 month after treatment had been discontinued. At 3 and 6 months after treatment was discontinued the average scores in the neuromuscular electrical stimulation group were ignificantly better than those in the control group. Both group showed no significant within and between group significant change in ADL	Three weeks of neuromuscular electrical stimulation to the affected upper extremity of patients with stroke improves motor recovery. The effect persists for at least 6 months.	3
149	Hayner et als, (2010)	USA	**Sample size: I=** 6 C= 6 **Age Range:** NR **Mean Age:** **I=** 54.00 + 11.628 C= 59.50 + 11.777 **PSD(days):** I= 642.33 + 421.121 C= 2039.00 + 925.328 **TOS(thromboemboli/hemorrhagic):** I = NR C = NR	occupational therapy and additional home practice while wearing a mitt on the unimpaired upper extremity (UE) (constraint induced therapy)	Occupational therapy and additional home practice while being intrusively and repetitively cued to use both UEs.	Motor function using The Wolf Motor Function Test (WMFT) and the therapy goals self-rating using the Canadian Occupational Performance Measure (COPM)	Significant improvements were found in WMFT and COPM scores across time in both groups. No significant between-group differences were found on the WMFT.	Both groups of interventions are comparable in improving UE function in people with chronic UE dysfunction after CVA. Treatment intensity rather than restraint may be the critical therapeutic factor.	2

150	Logan et al, 2004	UK	**Sample size:** I= 86<br>C= 82<br><br>**Age Range:** NR<br><br>**Mean Age:**<br>I= 74 ±8.4<br>C= 74 ±8.6<br><br>**PSD(months):**<br>I= 11±8.4<br>C= 10 ±9.0<br><br>**TOS(ischemic/ hemorrhagic):** NR	Use of leaflets with assessment and up to seven intervention sessions by an occupational therapist	Use of Leaflets describing local transport services for disabled people	The response to the query “do you get out of the house as much as you would like?” response to the query “how many journeys outdoors have you taken in the last month?” and scores on the Nottingham extended activities of daily living scale, Nottingham leisure questionnaire, and the 12 item version of the general health questionnaire	Participants in the treatment group were more likely to get out of the house as often as they wanted at both four months (relative risk 1.72, 95% confidence interval 1.25 to 2.37) and 10 months (1.74, 1.24 to 2.44). The treatment group reported more journeys outdoors in the month before assessment at both four months (P < 0.01) and 10 months (P < 0.01). At four Months, the mobility scores on the Nottingham extended activities of daily living scale were significantly higher in the intervention group, but there were no significant differences in the other secondary outcomes. No significant differences were observed in these measures at 10 months.	A targeted occupational therapy intervention at home increases outdoor mobility in people after stroke	3
151	Parker et al., 2001	UK	**Sample size:**<br>I:G1= 156<br>G2 = 153<br>C= 157<br><br>**Age Range:**<br>I: G1 = 66–78<br>G2 = 65–79<br>C= 65–78<br><br>**Median Age:**<br>I: G1 = 75<br>G2 = 72<br>C= 72<br><br>**PSD:** NR<br><br>**TOS(ischemic/ hemorrhagic):** NR	G1 = occupational therapy with activity of daily living training, aimed at improving independence in self-care tasks<br><br>G2 = occupational therapy with leisure therapy aimed at practicing the leisure tasks as well as any ADL tasks necessary to achieve the leisure	no occupational therapy treatment	Mood (the General Health Questionnaire [GHQ]); leisure activity (the Nottingham Leisure Questionnaire [NLQ]); independent in ADL (the Nottingham Extended ADL Scale [NEADL]);	In all the outcomes, no significant improvement was showed in the three groups and no significant difference in mean of outcomes were noted between the groups	All the interventions showed no significant benefit to the measured outcomes in this study.	1

				objective.					
**152**	Nelson et al., 1996	USA	**Sample size:** 26<br>**Age Range:** NR<br>**Mean Age:** NR<br>**PSD:** NR<br>**TOS(ischemic/ hemorrhagic):** NR	Occupationally embedded exercise, involving a simple dice game with bilaterally assisted supination	Rote exercise with bilaterally assisted supination	Degrees of rotation of the handle that was grasped during the exercise (a pen recorder)	There was an improved but not significant rotation of the handle in the intervention group ($p < 0.05$); but none in the control group	Occupationally embedded exercise may provide an improvement in hand rotation in stroke survivals.	2

Constraint-Induced Movement Therapy (CIMT)

	Study (Authors, year)	Country	Participants	Interventions/ Model of care	Control/ comparison	Outcomes assessed/ outcome measures	Results	Conclusion	Quality score
**153**	Stock et al,. (2018)	Norway	47 patients<br>**Age** range(SD):<br>Early CIMT= 65.3 (8.9) yrs<br>Delayed CIMT=61.0 (14.8)yrs<br>**PSD**(SD):<br>Early CIMT =16.6 (7.2)days<br>Delayed= CIMT 18.0 (6.5)days	Constraint-induced movement therapy (CIMT) plus standard rehabilitation	Standard rehabilitation	Wolf Motor Function Test (WMFT); the secondary measures were Nine-Hole Peg Test (NHPT), the Fugl-Meyer Assessment (FMA) of the upper extremity, Stroke Impact Scale, and Modified Rankin Scale (MRS)	No significant differences in the primary outcome measure between the early and delayed CIMT groups (p = .91).<br><br>The early CIMT group showed significantly better results in the MRS (p = .02) at T2 than the delayed intervention group	the early CIMT intervention group showed a faster recovery curve than the delayed intervention group, which can be a clinically	4

			**TOS(** ischaemic /haemorrhagic) Early CIMT =23/1 Delayed= CIMT 20 /3			[uses not ststed]		important finding for patients in the acute phase	
154	Doussoulin et al, (2017)	Chile	36 subjects **Age** (years): E: 58.33±10.38 C:48.75±18.60 **PSD**: N/A **TOS** ischaemic & haemorrhagic [N/S]	E: Modified Constrined-Induced Movement Therapy (CIMTm) done collectively	C: CIMTm done individually	Functional Indipendence Measure (FIM) Scale to measure functional independence in everyday activities	Significant differences ($F_{1.31} = 42.78$, $p < 0.001$, $\eta 2p = 0.72$) in favour of the collective intervention modality.	Both modalities of CIMTm intervention promote functional independence.	**4**
**155**	Liu et al, (2016)	China	86 **Age** (years), C=67.87±9.42 E=65.07±6.70 **PSD** (days): C= 9.69 (1.00) E= 8.44 (0.62) **TOS:** ischaemic	E: Self-regulated modified constraint-induced movement therapy (SR-mCIMT)	C: Modified constraint-induced movement therapy (mCIMT)	- Action Research Arm Test (ARAT)<br>- Fugl-Meyer Assessment (FMA)<br>- Lawton Instrumental ADL Scale (Lawton IADL)<br>- Motor Activity Log (MAL)<br>*[uses not stated]*	-Significant differences found, SR-mCIMT outperforming the other groups after the intervention (ARAT, P = 0.006; FMA, Lawton IADL and MAL, all Ps < 0.001).<br>-In terms of the carry-over effect, the SR-mCIMT group outperformed in the hand and coordination subscales of ARAT and FMA (P = 0.012–0.013) and the self-perceived quality of arm use (P = 0.002)	SR-mCIMT could produce an added effect in functional regain in patients post stroke	4
**156**	Thrane et al, 2015	United kingdom	P: Patients in early stages of stroke recovery Sample size:47 (E:24, C:23); **Mean Age** (Years): E= 65.3±8.0, C=61.0±14.8; **PSD:** E=16.6±7.2, C=18.0±6.5 **TOS**(ischemic/hemorrhagic): E=23/1; C=20/3	Constraints induced movement therapy (CIMT)	Treatment based on the Norwegian guide-lines	- Arm motor function (WMFT);<br><br>- UE function (UE-FMA);<br><br>- Dexterity (NHPT)<br><br>- Arm use ratio (SIS).	After treatment, the mean timed WMFT score was significantly better in the CIMT group compared with the control group.<br><br>NHPT, was significantly better in the CIMT group, whereas the other test results were similar in both groups.<br><br>At the 6-month follow-up, the 2 groups showed no significant difference in arm impairment, function, or use in daily activities.	Despite a favorable effect of CIMT on timed movement measures immediately after treatment, significant effects were not found after 6 months.	3

**157**	Bang et al, 2015	Korea	P: Subacute stroke patients with moderate motor impairment; Sample size= 18 (E=9, C=9); **Mean age** (yrs): E= 60.22±5.76, C= 59.33±8.2; **PSD** (months): E= 3±1.12, C = 3.56±1.01; **TOS**(ischemic/hemorrhagic): E=6/3; C=5/4	(modified Constraint Induced Movement Therapy) mCIMT + Trunk Restraint (TR)	mCIMT only	- UL Function (ARAT),<br>- UL Impairment (UE-FMA),<br>- ADL Capacity (mBI),<br>- UL Activity (MAL),<br>- MEER (Dartfish motion analysis software)	- The mCIMT + TR group exhibited more improvement in the ARAT, FMA, mBI, MAL and MEEAR compared with the mCIMT group.<br><br>- Statistical analyses showed significantly different in ARAT (P = 0.046), FMA (P = 0.008), MBI (P = 0.001), MAL-AOU (P = 0.024), MAL-QOM (P = 0.010) and MEEAR (P = 0.001) between groups	mCIMT+TR is more helpful to improve upper-extremity function than mCIMT only in subacute stroke patients with moderate motor impairment	5
**158**	van Delden, et al, 2015	Netherlands & UK	**Sample size:**<br>I: G = 22<br>G2 = 19<br>C= 19<br>**Age Range:** NR<br>**Mean Age:**<br>I: G =59.8 +13.8<br>G2 = 62.6 +9.8<br>C= 56.9+12.7<br>**PSD(weeks):**<br>I: G =9.2 +6.8<br>G2 = 7.8 +4.9<br>C= 11.1 +6.8<br>**TOS(ischemic/hemorrhagic):**NR	G1 = Unilateral upper limb training: modified constraint-induced movement therapy (mCIMT).<br><br>G2 = Bilateral upper limb training: modified Bilateral Arm Training with Rhythmic Auditory Cueing (mBATRAC)	Dose matched conventional treatment (DMCT)	Limb coupling (rhythmic bimanual coordination tasks); Relative phase (RP) shift (Unimanual Motor task), range of motion and movement harmonicity (Bimanual Coordination and Unimanual Reference),	There were no significant between-group differences in change scores from baseline to postintervention and from postintervention to follow-up with regard to interlimb coupling. However, the mBATRAC group showed greater movement harmonicity and larger amplitudes with the paretic hand after training than the mCIMT and DMCT groups.	The degree of coupling between both hands was not significantly higher after bilateral than after unilateral training and control treatment. Although improvements in movement harmonicity and amplitude following mBATRAC may indicate a beneficial influence of the interlimb coupling	3

**23**	Hsieh et al., 2014	Taiwan	**Sample size:** G1=16, G2= 16; C= 16 **Mean Age:** G =54.41±7.77 G2=52.34±13.20 C= 54.12 ±9.98 **PSD(months):** G1=20.56±13.95 G2=23.56±15.43 C=27.81 ±19.07 **TOS(ischemic/ hemorrhagic):** G1=7/9;G2=12/4 ; C= 8/8	G1 = Robot assisted therapy (RT) with the distributed form of constraint induced therapy (CIT) (RT + dCIT)<br><br>G2 = Robot assisted therapy (RT)	C = Conventional rehabilitation (CR)	Primary: motor impairment (FMA) and motor function (Wolf Motor Function Test (WMFT) with subscales: Functonial Ability subscale [WMFT-FAS] and performance time subscales [WMFT-TIME]).<br><br>Secondary: ADL (Motor Activity Log (MAL)) and amount of arm movement (accelerometers)	- Significant within groups improvements in FMA total, distal, & proximal (P<0.05).<br>- Significant between groups difference in FMA (p<0.01): RT+dCIT > RT (P = 0.05) and CR (P<0.01); RT > CR (P=0.04).<br>-Significant within-groups improvements on the WMFT (P <0.05).<br>- Significant between-group difference in WMFT-FAS (P=0.01): RT+dCIT > RT and CR groups on WMFT-FAS (P=0.01), but no significant between-group differences in WMFT-TIME (P = 0.28).<br>-Significant within-groups improvements over time on the MAL-AOU and MAL-QOM (P<0.05)<br>-Improvements on the MAL and accelerometers were not significantly different among the three groups.	RT in sequential combination with CIT led to additive effects on participants' motor ability and functional ability to perform motor tasks after stroke.	3
**159**	van Delden et al., 2013	Netherlands	**Sample size:** G1=22; G2=19; C=19 **Mean Age:** G1= 59.8 + 13.8 G2= 62.6 + 9.8 C= 56.9 + 12.7 **PSD(weeks):** G1 = 9.2 + 6.8 G2 = 7.8 + 4.9 C = 11.1 + 6.8 **TOS(ischemic/hemorrhagic):**NR	G1 = modified constraint-induced movement therapy (mCIMT)<br><br>G2 = modified bilateral arm training with rhythmic auditory cueing (mBATRAC)	A dose-matched conventional treatment (DMCT)	Upper limb motor functions (ARAT)	No significant difference in change scores was obtained on ARAT scores between the groups at posttest and follow-up. Both groups showed significant improvement in the ARAT test which lasted up to the 6 weeks follow-up	None of the treatment groups are superior in improving upper limb function 1 to 6 months after stroke	3
**160**	Fritz al, 2013	Germany	**Sample size:** I= 50; C= 50 **Age Range (Yrs):** I = 41–81; C = 34–84 **Mean Age (yrs):** I= 60.7; C= 60.2 **PSD(days):** I= 36.7; C= 32.9	I: Modified version Constraint-induced aphasia therapy (CIAT)	C: Standard aphasia therapy	Language (Aachener Aphasia Test [AAT]) and Communication (Communicative Activity Log [CAL]);	Significant within-groups improvement in language skills (p<0.05); but no significant between-group in: token test, repetition, written language and comprehension (p>0.05).<br>-Significant within-group improvement in the amount of communication (p<0.05) and language comprehension (p<0.05).<br>-There were no significant differences	Both CIAT and conventional therapy performed with equal intensity are efficacious methods for	3

			**TOS(ischemic/ hemorrhagic):** I = 43/7;C = 42/8				concerning the CAL results between both groups. The improvements remained stable over a 1-year follow-up period.	patients with sub-acute aphasia	
**161**	Lang, et al, 2013	USA	**Sample size:** I= 106 C= 116<br><br>**Age Range:** NR<br><br>**Mean Age:** NR<br>**PSD:** NR<br><br>**TOS:** NR	Early constraint-induced movement therapy (E-CIMT)	Late constraint-induced movement therapy (L-CIMT)	Motor functions (WMFT tasks);	E-CIMT showed significant within-group improvements in 3fine-movement tasks & in total noncompleted tasks (P<u><</u>0.0036), whereas the L-CIMT did not.<br>E-CIMT>L-CIMT for the lifting pencil task and total noncompletes. During the year following CIMT, neither group showed significant changes in completion of WMFT tasks.<br>Over all time intervals, only E-CIMT displayed significant improvement in several tasks and total noncompletes.<br>Between groups, there were significant and almost significant differences between the improvements of the 2 groups in 3 tasks requiring fine distal movement.	Receiving CIMT earlier appears to improve reacquisition and retention of WMFT tasks, especially those requiring fine motor skills.	2
162	Treger, et al, 2012	Israel	**Sample size:** I=9 C=19<br>**Age Range:** NR<br>**Mean Age:**<br>I = 62.0 ± 10.4<br>C = 61.5 ± 8.4<br>**PSD (Days):**<br>I= 39.8 ± 28.4<br>C = 23.3 ± 24.1<br>**TOS:** NR	Physical rehabilitation with Constraint-induced movement therapy (CIMT)	Physical rehabilitation without Constraint (PRhb)	PO: hand function (MFT); impairment (National Institute of Health Stroke Scale (NIHSS)); disability (functional independent measure (FIM))	There is no significant difference in all the outcomes measured between the groups (P >0.05). a significant pre-post intervention change was found in some of the MFT domains such as: peg transfer, ball grasping and eating with a spoon in both groups (P<0.05) but there is no significant change in peg transfer in the control group	There is an improvement in limb function with CIMT & PRhb with no statistically significant difference between the 2interventions	2
**163**	Krawczyk et al., 2012	Poland	**Sample size:**<br>I=24; C=23<br>**Age (Yrs)**:<br>I= 48±14;<br>C=46±13<br>**PSD**: NR<br>**TOS (ischemic/ haemorrhagic):**<br>I =19/5; C=20/3	Physiotherapy and Constraint-induced movement therapy (CIMT): the sling-constraint (S-CIMT)	PT + CIMT: the voluntary constraint (V-CIMT)	Performance (Rivermead Motor Assessment (RMA) Arm scale), ADL (Motor Activity Log–Quality of Movement (MAL-QOM))	There were no statistically significant differences between the groups in all the measured outcomes	V-CIMT in the intact arm is equivalent to S-CIMT during massed practice of paretic arm.	3
**1**	Bru	N	**Sample size:**	Modified	Bimanual	PO: arm motor	No significant between group difference	Bimanual	3

**64**	nner, et al, 2012	orway	I=14; C=16<br>**Age (Years)**:<br>I= 61.0 ±10.0<br>C=64.8±12.8<br>**PSD** (Days):<br>I=48.43 ±39.3<br>C=36.9 ±25.1<br>**TOS:**Ischemic/hemorrhagic:<br>I =13/1; C=12/4	constraint-induced movement therapy (mCIMT) with an emphasis on unimanual tasks,	task-related training, emphasizing bimanual tasks	function (Action Research Arm Test), SO: dexterity of the hand (Nine-Hole Peg Test) and amount and quality of use of the affected arm in daily life activities (the Motor Activity Log).	was found in all the outcomes. But significant improvement in all the outcomes were noticed in both groups	training was as effective as modified constraint-induced movement therapy in improving arm motor function.	
**165**	Huseyinsinoglu, et al, 2012	Turkey	**Sample size**: I = 11 C= 11<br><br>**Age Range:** NR<br><br>**Mean Age** (Years): I= 49.1 ± 13.7<br>C= 48.2 ± 15.4<br><br>**PSD**: NR<br><br>**TOS(**Ischemic/hemorrhagic) I = 7/4<br>C = 10/1	constraint-induced movement therapy for 3 hours per day during 10 consecutive weekdays (CIMT)	Bobath Concept group was treated for 1 hour (Bobath)	Primary outcome: motor ability (Wolf Motor Function Test) Secondary outcomes: ADL (Motor Activity Log-28), arm performance (Motor Evaluation Scale for Arm in Stroke Patients (MESUPES)).	Significant improvements were seen after treatment only in the 'Amount of use (AOU)' and 'Quality of movement (QOM)' subscales of the Motor Activity Log-28 in the intervention group over the the Bobath Concept group (*P* = 0.003; *P* = 0.01respectively). There were no significant differences in Wolf Motor Function Test Motor Evaluation Scale for Arm in Stroke Patients (*P* > 0.05) and Functional Independence Measure scores (*P* = 0.259) between the two intervention groups.	CIMT & Bobath have similar efficiencies in improving functional ability, speed and QOM in the paretic arm among stroke patients with a high level of function. CIMT seems to be more efficient than the Bobath t in improving the AOU & QOM of affected arm .	3
**166**	Wu, et al, 2012	Taiwan	**Sample size:**<br>G1=20, G2=19, C=18<br>**Mean Age**:<br>G1=54.0 ± 9.7,<br>G2=56.3 ± 12.2<br>C=58.6 ± 11.6<br>**PSD** (Months)	G1: Distributed constraint-induced therapy (dCIT),<br><br>G2:distributed constraint-induced therapy	usual and customary care: treatment based on neurodevelopmental principles	Motor function (The Action Research Arm Test (ARAT)); daily function (Motor Activity Log); perception of instrumental ADL participation (Frenchay	-dCIT-TR and dCIT exhibited higher overall scores on the ARAT, FAI, and hand function domain of the SIS and better quality of movement and larger amount of use (of the affected arm) on the MAL than NDT.<br>-dCIT-TR, demonstrated greater improvements on the ARAT grip subscale	dCIT & dCIT+TR provided better improvement in motor function, ADL and	2

			G1=15.7 ± 13.5<br>G2 = 13.7 ± 7.3<br>C= 17.7 ± 13.4<br>**TOS**: NR	combined with trunk resistance dCIT-TR	(NDT)	Activities Index (FAI)), QOL (Stroke Impact Scale (SIS)).	and FAI outdoor activities scale than dCIT & NDT. However, dCIT showed greater improvements on the strength domain of the SIS after training than participants receiving dCIT-TR & NDT	quality of life than the usual care	
**167**	Wang et al., (2011)	China	**Sample size:**<br>G1=10; G2=10; C= 10<br><br>**Age (yrs):**<br>**G1=**63.5±9.63<br>G2 = 59.4±10.89<br>C= 67±7.45<br>**PSD(weeks):**<br>G1 = 12.7±9.72<br>G2 = 11.9± 9.59<br>C = 9.4±5.38<br>**TOS (ischemic/ hemorrhagic):**<br>G1=8/2;G2= 7/3; C = 8/2	G1 = Intensive conventional rehabilitation (ICR)<br>G2 = modified constraint-induced movement therapy (mCIMT)	conventional rehabilitation (CR)	Motor function assessed using the Wolf Motor Function Test (WMFT)	mCIMT & ICR improved their function ability scores in WFMT significantly more than CR after 2 weeks of treatment ($p < 0.05$), but all groups reached comparable levels at the end of 4 weeks of intervention. However, only mCIMT proved to have robust and systematic effects on the functional ability scores, as revealed by the large, positive and significant correlation between the initial scores and the scores 2 and 4 weeks after the intervention. The median performance time of the WMFT decreased significantly in all groups after 4 weeks of treatment ($p < 0.05$), but only mCIMT showed significant improvements at both 2 and 4 weeks after the initiation of treatment.	Compared with classical intervention, mCIMT showed an apparent advantage over both conventional intervention and intensive conventional rehabilitation for patients after stroke.	3
**168**	Wu, et al., (2011)	Taiwan	**Sample size:**<br>**I**: G1 = 22 G= 22 C=22<br>**Age Range:** NR<br>**Mean Age:**<br>**I: G1=** 51.91±11.93<br>G2 = 52.22 ± 10.72<br>C = 55.19 ± 2.50<br>**PSD(months):**<br>I: G1= 14.91±12.04<br>G2 = 15.92 ± 13.74 C = 17.77 ± 12.45<br>**TOS(ischemic/h**	G1 = distributed constraint-induced therapy (dCIT),<br><br>G2 = bilateral arm training (BAT),	Standard therapy (75% neurodevelopmental treatment &<br>25% compensatory practice on functional tasks with the unaffected UE or	Motor function using Wolf Motor Function Test (WMFT), and the Motor Activity Log (MAL); kinematics (a 7-camera motion analysis system)	The dCIT and BAT groups had smoother reaching trajectories in unilateral and bilateral tasks than the CT group. The BAT group, but not the dCIT group, generated greater force at movement initiation than the CT group during the unilateral and bilateral tasks. The dCIT patients had decreased WMFT time and higher functional ability scores than the CT patients. MAL results pointed to better performance in the amount and quality of use of the affected arm by the dCIT group than BAT and CT patients.	BAT and dCIT exhibited similar beneficial effects on movement smoothness but differential effects on force at movement initiation and functional performance.	3

			**emorrhagic):** NR		both UEs.				
149	Hayner, etal, (2010)	USA	**Sample size:** **I=** 6, C= 6 **Age Range:** NR **Mean Age:** **I=** 54.00 ±11.628 C=59.50±11.777 **PSD(days):** I= 642.33 ± 421.121 C= 2039.00 ± 925.328 **TOS(thromboemboli/hemorrhagic):** NR	occupational therapy and additional home practice while wearing a mitt on the unimpaired upper extremity (UE) (constraint induced therapy)	Occupational therapy and additional home practice while being intrusively and repetitively cued to use both UEs.	Motor function using The Wolf Motor Function Test (WMFT) and the therapy goals self-rating using the Canadian Occupational Performance Measure (COPM)	Significant improvements were found in WMFT and COPM scores across time in both groups. No significant between-group differences were found on the WMFT.	Both groups of interventions are comparable in improving UE function in people with chronic UE dysfunction after CVA. Treatment intensity rather than restraint may be the critical therapeutic factor.	2
169	Wolf, et al., (2010)	USA	**Sample size: I=** 106 C= 86 **Age Range:** NR **Mean Age:** **I=** NR, C= NR **PSD(days):** I = 178±64 C = 187±67 **TOS(thromboemboli/hemorrhagic):** NR	Constraint-induced movement therapy (CIMT) delivered immediately after randomization	Constraint-induced movement therapy (CIMT) delivered 1 year after randomization	Primary: functional ability using Wolf Motor Function Test and ADL using Motor Activity Log<br><br>Secondary: health status using Stroke Impact Scale	Although both groups showed significant improvements from pretreatment to 12 months after treatment, the earlier CIMT group showed greater improvement than the delayed CIMT group in Wolf Motor Function Test Performance Time and the Motor Activity Log (*P*<0.0001), as well as in Stroke Impact Scale Hand and Activities domains (*P*<0.0009 and 0.0214, respectively). Early and delayed group comparison of scores on these measures 24 months after enrollment showed no statistically significant differences between groups.	CIMT can be delivered to eligible patients 3 to 9 months or 15 to 21 months after stroke. Both patient groups achieved approximately the same level of significant arm motor function 24 months after enrollment.	2
**170**	Lin, et al.,	Tai	**Sample size: I=**5 C=8 **Age Range:** NR	Functional training with distributed form	traditional rehabilitation	PO: motor impairment recovery (The UL subscale of the Fugl-	dCIT group showed a significantly greater increase than the control group on the FMA (*P* = 0.04) and the MAL [*P*=0.02],	Brain adaptation may be	2

	2010	wan	**Mean Age:**<br>**I=**46.4 ± 26.0<br>C= 51.6 ± 12.4<br>**PSD:**<br>I=21.5 ± 12.3<br>C=16.3 ± 18.3<br>**TOS:** NR	of constraint-induced therapy (dCIT)	matched to the dCIT in duration and intensity	Mayer Assessement [FMA]), ADL (Motor Activity Log [MAL]), and hemistpheric changes (functional magnetic resonance imaging examination)	quality of movement domain of MAL [*P*= 0.02]). The functional magnetic resonance imaging data showed that distributed form of constraint induced therapy significantly increased activation in the contralesional hemisphere during movement of the affected and unaffected hand. The control intervention group showed a decrease in primary sensorimotor cortex activation of the ipsilesional hemisphere during movement of the affected hand	modulated by specific rehabilitation practices, such as dCIT which provides higher motor and functional iimprovement in post stroke patients.	
**171**	Azab, et al., 2009	Jordan	**Sample size:** I = 27, C= 17<br>**Age Range:** NR<br>**Mean Age**: NR<br>**PSD**: NR<br>**TOS(ischemic/hemorrhagic)**: NR	traditional therapy with the constraint-induced movement therapy (CIMT)	traditional therapy only	ADL (Barthel Index[BI])	Intervention group showed statistically significant improvement in the BI compared to the control group, and this effect was maintained up to 6 months follow-up.	There is an overall significant improvement in ADL status for patients who received CIMT	1
**172**	Dromerick, 2009	USA	**Sample size:** I: G1=16; G2=19 C=17<br>**Mean Age:**<br>G1=64.5±15.5<br>G2 = 62.8±12.8<br>C=64.7±14.6<br>**PSD(days):**<br>G1= 9.94±4.8<br>G2=8.8±3.1<br>C=10.4 ± 5.7<br>**TOS (% Ischemic)**<br>G1= 81<br>G2 = 74<br>C=76	G1 = High intensity Constraint induced movement therapy (CIMT)<br><br>G2 = low intensity Constraint induced movement therapy (CIMT)	C = traditional occupational therapy	Motor impairment (Action Research Arm Test [ARAT]; Stroke severity (National Institute of Health Stroke Scale (NIHSS); disability(Functional Independence Measure [FIM]); Hand strength, dexterity, and fine and gross motor ability (The Stroke Impact Scale [SIS]: hand function subscale); Pain (The Wong-Baker Faces Scale); Depression (The Geriatric Depression–15 Scale)	A significant improvement in ARAT scores were seen in all the groups (p <0.0001) with G1 having the lowest gains; There were no significant differences between G1 and control; a significant group x time interaction (p < 0.01) was observed for the total ARAT when all three groups were compared; no significant differences across the three treatment groups for the FIM Upper Extremity Scale; with SIS, G1 and control has significant improvement while G2 had significantly lower scores; There were no significant differences in mean pain rate and depression among the three treatment groups.	Constraint-induced movement therapy (CIMT) was equally as effective but not superior to an equal dose of traditional therapy during inpatient stroke rehabilitation.	1
**17**	Brogår	De	**Sample size:**<br>**I=**12, C=12	Traditional neurorehabilitati	Traditional	Upper extremity functions (mMAS);	Large improvements in arm and hand function were found in both the mitt group	In this study, no effect of	3

**3**	dh et al, 2009	nmark	**Mean Age:** I=58.5 +6.2 C=56.7+10.5 **PSD(weeks):** I=6.7+2.3 C=7.3+3.1 **TOS:** NR	on and constraint induced movt therapy (CIMT) with mitt use over the less affected hand for 90% of waking hours	neurorehabilitation and CIMT without the use of mitt	Daily Hand Activities (Sollerman Hand Function Test); Tactile Somatosensation (2-Point Discrimination Test); ADL (Motor Activity Log [MAL])	and the non-mitt group on the Sollerman hand function test (p < 0.0001), on the MAS test (p < 0.0003) and on the MAL test (p < 0.0002), respectively but not on tactile somatosensation.. However, no statistically significant differences between the groups were found in any measures at any point in time.	using a restraint in patients with subacute stroke was found.	
**174**	Lin, et al, 2009a	Taiwan	**Sample size:** G1=20; G2=20; C=20 **Mean Age:** G1=55.28±9.34 G2=51.58 ±8.67 C=50.70 ±13.93 **PSD(months):** G1=21.25±21.59 G2=18.50±17.40 C=21.90±20.51 **TOS:** NR	G1 = distributed Constraint-Induced Therapy (dCIT)<br><br>G2 = Bilateral Arm Training (BAT)	Conventional therapy	Motor impairment (Fugl-Meyer Assessment [FMA] scale); daily function (Functional Independent Measure [FIM]); use of the affected arm in real world situations (Motor Activity Log[MAL]); and quality of life (Stroke Impact Scale [SIS])	The result showed a significant difference in mean FMA and SIS between the groups. Post hoc analyses revealed that, in comparison with the control group, dCIT (P = .001) and BAT (P < .001) groups each produced greater improvements in the overall FMA score. The dCIT also showed better improvement than the BAT group in MAL (P = .011), quality of life in the overall SIS (P = .003), and the domains of ADL/IADL (P = .024) and social participation (P = .009) of the SIS. dCIT also showed better improvement than the control group in MAL (P < .001), quality of life in the overall SIS (P = .007), and the domains of ADL/IADL (P = .004) and daily function (P = .015) of SIS	BAT may uniquely improve proximal UL motor impairment. In contrast, distributed CIT may produce greater functional gains for the affected UL in subjects with mild to moderate chronic hemiparesis	1
**175**	Lin, et al, 2009b	Taiwan	**Sample size:** I=33; C=30 **Age Range (Yrs):** I=30-79; C= 36-78 **Mean Age** (Yrs): I=54.14 +11 C=57.38 +12.78 **PSD(months):** I=16.93 +11.13 C=13.23 +8.84	constraint-induced movement therapy (CIT)	Conventional rehabilitation	Motor impairment (FMA); ADL (Functional Independence Measure [FIM]); self-perceived functional use of the affected limb (Motor Activity Log [MAL]); extended ADL (Nottingham Extended Activities of Daily	The CIT group performed significantly higher than the control group in all the measured outcomes; also, all, Except MAL, showed a significant mean difference between the groups.	Constraint induced movement therapy improves motor functions, ADL, functional outcome and quality of life	2

			**TOS(ischemic/hemorrhagic):** I=26/7; C=21/9			Living [NEADL] Scale); and Health Related Quality of Life (Stroke Impact Scale)		in stroke patients	
**176**	Sawaki et al., 2008	USA	**Sample size:** I=17; C=13 **Age Range (Yrs):**I=30-79; C= 36-78 **Mean Age (Yrs)** I=54.4 ± 3.8 C=58.5 ± 3.5 **PSD(days):** I=28.9 ± 12 C=26.1 ± 10.9 **TOS(ischemic/hemorrhagic):** NR	Early Constraint Induced Movement Therapy (CIMT); receiving CIMT immediately after baseline evaluation	Late Constraint Induced Movement Therapy (CIMT); receiving CIMT after 4 months	Upper extremity function (Wolf Motor Function Test (WMFT)); cortical reorganization (transcranial magnetic stimulation [TMS])	Both experimental and control groups demonstrated improved upper extremity function 2 weeks after baseline. The experimental group showed significantly greater improvement in grip force domain of WMFT after the intervention and at follow-up (P = .049). The experimental group also had a borderline significance increase in the TMS motor map area compared with the control group over a 4-month period with (P = .053)	CIMT produced statistically significant and clinically relevant improvements in arm motor function with effect lasting up to 4months. The corresponding enlargement of TMS motor maps appears to play an important role in CIMT dependent plasticity	1
**177**	Lin, et al, 2008	Taiwan	**Sample size:** I=12; C=10 **Mean Age:** NR **PSD:** NR **TOS(ischemic/hemorrhagic):** NR	constraint-induced movement therapy (CIMT) r	traditional intervention	motor impairments (FMA); changes in ADL function (FIM); perceived functional use of the affected limb including amount of use in the affected limb and quality of movement (MAL); extended ADL abilities (The NEADL) .	There was a significant and moderate-to-large effects in favor of the CIMT group on the FMA and two domains of the FIM (i.e., self-care and locomotion); Nonsignificant and small-to-moderate effects were found in the subtests (AOU and QOM) of the MAL. A significant and moderate effect was found in the domain of mobility on the NEADL in favor of CIMT but not in other domains.	CIMT provides improvement in motor performance and some aspects of basic and extended activities of daily living.	2
**178**	Gauthier et	UK	**Sample size:** I= 20 C= 16	constraint-induced movement	comparison therapy	ADL (The Quality of Movement scale of the Motor Activity Log	Both group showed improvement in MAL at both post intervention and follow-up (P < 0.0001) with the intervention group	Both CIMT and comparison	2

	al, 2008		**Age Range:** NR<br><br>**Mean Age:** NR<br><br>**PSD:** NR<br><br>**TOS(ischemic/hemorrhagic):**<br>I = NR<br>C =NR	therapy (CIMT)		[MAL]); and motor ability (the Wolf Motor Function test); changes in grey mater (longitudinal voxel-based morphometry)	showing higher improvement. Both groups also showed improvement in Wolf Motor Function Test but no difference between the two groups. There was a significant deference in ADL between the two groups. CI therapy group exhibited profuse increases in gray matter in sensory and motor areas both contralateral (*P*= 0.002) and ipsilateral (*P*= 0.023) to the affected arm, as well as in bilateral hippocampi (*P*= 0.033). There was a significant grey mater increase in the intervention group (p<0.05) but not in the control group. increase in gray matter from pretreatment to posttreatment differed significantly between groups for the ipsilateral sensorimotor cluster (*P*=0.041) and the hippocampus (*P*=0.006) but not for contralateral cluster (*P*=0.087),	therapy showed similar improvement in motor functions, however, the intervention group provided more effect in grey matter changes.	
**179**	Page, et al, 2008	USA	**Sample size:**<br>G1=13; G2=12; C=10<br>**Mean Age:** NR<br>**PSD:** NR<br>**TOS(ischemic/hemorrhagic):**<br>NR	G1 = Modified constraint induced therapy (mCIT)<br><br>G2 = Traditional rehabilitation (TR)	No therapy	Primary: Motor impairment (ARAT); upper limb function (upper-extremity section of the FM); ADL (MAL);	After intervention, the mCIT group's MAL, FM and ARAT scores increased significantly; only small but non significant changes were noticed in TR group respectively. Caregiver MAL showed similar trend. The control groups showed a negative changes in both the FM and ARAT scores. A significant difference in mean ARAT score were found between the groups (*P*<.0001) but not in FM	Modified constraint induced therapy increases the improvement of the affected arm function in stroke survivors	2
**180**	Wolf et al., 2008	USA	**Sample size:**<br>I= 106; C=116<br>**Mean Age:** NR<br>**PSD:** NR<br>**TOS:** NR	Constraint induced movement therapy (CIMT)	Usual care	Primary: arm motor activity (Wolf motor function test [WMFT] and motor activity log [MAL]); quality of movement (functional ability scale (FAS)); health status and quality of life (SIS)	The intervention group showed significant improvement in WMFT, MAL and SIS and there is a significant difference in mean WMFT between the groups but not in SIS, MAL nor FAS.	CIMT showed effective improvement in motor function more than usual care.	2
**1**	Lin,	T	**Sample size:**	modified	traditional	Independence in the	For the intervention group, there was a	In addition to	3

**81**	Wei, Lee **&** Liu**, 2007**	aiwan	I= 17; C=15<br>**Mean Age:**<br>I= 57.11 ±18.30<br>C= 58.77 ±15.15<br>**PSD(months):**<br>I=15.97±3.46<br>C= 16.61±2.89<br><br>**TOS(ischemic/hemorrhagic):**<br>1 = NR<br>C = NR	constraint-induced movement therapy	rehabilitation	ADL (Motor Activity Log [MAL] and Functional Independence measure [FIM]); Kinematic variables for reaching and grasping: reaction time, movement time, the percentage of movement time where peak velocity occurs, movement units, maximum grip aperture, and the percentage of movement time where maximum grip aperture occurs (An analysis programme coded by LabVIEW (National Instruments, Inc.) language)	significant main effect of group (P=0.041) for reaching and grasping kinematics. A significant improvement in reaction time. and significant moderate effect for group was also shown for reaching strategy (P=0.024) and the percentage of reach where peak velocity occurs (P=0.046) but not on movement units (P=0.18). There was also a significant improvement in the intervention group in ADL at post intervention (P=0.012) and follow up (P<0.0001), in quality of movement subscale of MAL (P<0.0001) and a significant and moderate effect on Functional Independence Measure (P=0.016). The group showed greater improvements than the traditional rehabilitation group in amount of use and quality of movement of the affected arms during daily activities. The intervention group also showed higher improvement in FIM than in the traditional rehabilitation group.	improving functional use of the affected arm and daily functioning, modified constraint-induced movement therapy improved motor control strategy during goal-directed reaching, a possible mechanism for the improved movement performance of stroke patients undergoing this therapy.	
**182**	Wu, et al, 2007	Taiwan	**Sample size:** I= 24 C= 23<br><br>**Age Range:** I= NR C= NR<br><br>**Mean Age:**<br>I=53.93±11.20<br>C= 56.77±12.90<br><br>**PSD(months):**<br>I=12.51±9.64<br>C= 11.98±11.72	constraint-induced movement therapy (CIMT)	traditional intervention	kinematic variables of reaching movement used to describe the control strategies for reaching (a 6-camera motion analysis system with a personal computer); motor-impairment severity (Fugl-Meyer Assessment (FMA)); and functional ability of the upper extremity	For kinematic variables, the CIMT group compared with control group had: shorter reaction time (*P*=.005), shorter normalized movement time (*P*=.021) smaller normalized total displacement (*P*=.021) and fewer normalized movement units (*P*=.010) reaching trajectories of the more affected arm. There was a significant and moderate to large effects in favor of the CIMT group on the FMA and MAL. There was less motor impairment for the CIMT group compared to the traditional intervention group (*P*=.019). Subjects in	In addition to improving motor performance at the impairment and functional levels, CIMT conferred therapeutic benefits on control strategies	3

			**TOS:NR**			[AOU and QOM] (Motor Activity Log (MAL))	the CIMT group reported better performance in the MAL subsets: AOU (*P*=.001) and QOM (*P*=.001) of their more affected UE during daily activities.	determined by kinematic analysis.	
**183**	Wu, et al, 2007	Taiwan	**Sample size:** I= 13 C= 13<br><br>**Age Range:** NR<br><br>**Mean Age:**<br>I= 71.44+6.42<br>C= 71.94+6.79<br><br>**PSD:**<br>I= 6.70+8.99<br>C= 8.32+7.97<br><br>**TOS:**	modified constraintinduced movement therapy (mCIMT)	traditional rehabilitation	Motor impairment (FMA); changes in activity performance through performance observation (FIM); changes in activity performance through self-report (MAL); self perceived health related quality of life [HRQOL] and participation (SIS);	The results showed significant and moderate-to-large effects in favor of the mCIMT group on FMA (p = 0.008), FIM (p = 0.018), and MAL: AOU (p = 0.003), QO (p<0.001) and the strength (p = 0.015), ADL (p = 0.045) and stroke recovery section of SIS (p = 0.001). The result also showed significant and moderate effects of mCIMT on the overall SIS.	mCIMT is a promising intervention for improving motor function, daily function, and physical aspects of HRQOL in elderly patients with stroke	3
**184**	Wolf et al., 2006	USA	**Sample size:** I= 106 C= 116<br><br>**Age Range:** NR<br><br>**Mean Age:**<br>I= 61.0 + 13.5<br>C= 63.3 + 12.6<br><br>**PSD(days):**<br>I= 179.8 + 66.1<br>C= 187.7 + 70.8<br><br>**TOS(ischemic/ hemorrhagic):**<br>1 = 96/10<br>C = 98/18	multisite program of CIMT	customary care	Upper extremity motor function (the Wolf Motor Function Test [WMFT]) and a structured participant interview of real world arm use (MAL).	The CIMT group showed larger improvements than the control group on all measures of paretic upper-extremity function at posttreatment (*P*<.05), with the exception of the 2 WMFT strength items. The CIMT group, however, showed larger improvement than controls on these strength items at 12-month follow-up (*P*<.001). The CIMT group also showed larger improvement than the control group on WMFT Performance Time and both participant MAL scales at all follow-up testing occasions (ie, 4-, 8-, and 12-month follow-up; *P*<.01)	Among patients who had a stroke within the previous 3 to 9 months, CIMT produced statistically significant and clinically relevant improvements in arm motor function that persisted for at least 1 year	1
**185**	Page, et al, 2004	USA	**Sample size:** NR<br><br>**Age Range:** NR<br><br>**Mean Age:** NR	G1 = modified constraint induced movement therapy	No treatment	Impairment (FMA), dexterity (ARA Test), and ADL (MAL: AOU and QOU)	After intervention, the mCIMT group displayed a mean improvement on the FMA and ARA, patients receiving traditional rehabilitation exhibited less improvement on the FMA and ARA,	mCIMT may be an efficacious method of improving	2

			**PSD:** NR<br><br>**TOS(ischemic/hemorrhagic):**NR	(mCIMT)<br><br>G2 = traditional rehabilitation			whereas control patients exhibited negative changes on the FMAand ARA. MAL scores, both in terms of AOU and QOU, also increased between pre- and posttesting sessions for the mCIMT group a significant difference was seen between the three groups for the FMA (*P*=.002) and the ARA (*P*=.001). On the FMA, mCIMT differed significantly from both traditional rehabilitation and control while the difference between traditional rehabilitation and control therapy was not significant. On the ARA, subjects in both mCIMT and traditional rehabilitation exhibited significantly greater gains than did control subjects, but no difference was found between both groups.	function and use of the more affected arms of chronic stroke patients	
**186**	Van der Lee et al., 1999	Netherlands	**Sample size:** I= 31<br>C= 31<br><br>**Age Range:** NR<br><br>**Median Age:**<br>I= 59<br>C= 62<br><br>**PSD(median year):**<br>I= 3.4<br>C= 2.7<br><br>**TOS(ischemic/hemorrhagic):**NR	forced use therapy (immobilization of the unaffected arm combined with intensive training)	reference therapy of equally intensive bimanual training	Primary: activities (Personal Care and Occupation of the Rehabilitation Activities Profile (RAP)); Dexterity (ARAT)<br><br>Secondary: impairment level (UE section of ARA test); Amount of use (AOU) and quality of movement (QOM) of the affected arm (Motor Activity Log (MAL));	A significant main effect of treatment on ARAT score was found between the groups in favour of the intervention group. For the FMA, no significant differences between the 2 treatment conditions or any within-subject changes over time during any of the study periods, indicating no treatment effect on this scale. The experimental group showed significantly more improvement than patients in the reference group in the quality of movement subscore of MAL, while no significant effect of intervention both within group and between groups were found for the quality of movement subscore of MAL.<br><br>***Intention-to-Treat Analysis***<br>This analysis revealed no statistically significant treatment effects between the 2 groups	Forced use therapy provided a small but lasting effect of forced use therapy on the dexterity of the affected arm (ARAT) and a temporary clinically relevant effect on the amount of use of the affected arm during activities of daily living (MAL amount of use).	3

Mirror Therapy

Ref	Study (Authors, year	Country	Participants	Interventions/ Model of care	Control/ comparison	Outcomes assessed/ outcome measures	Results	Conclusion	Quality score
187	Arya et al, (2018)	India	31 subjects (E=17;C=14), **Age range (yrs)**: E=44.12±9.08 C=47.93±9.10 **PSD (month):** E=13.35±10.12 C=17.00±13.28 **TOS** (Ischaemic /Haemorharrgic) : E: 08/9; C:6/8	Sensory stimulus such as tactile perception and motor tasks on the less-affected hand using mirror box.	Dose-matched conventional program	-Semmes Weinstein Monofilament(cutaneous threshold), used to measure diminished cutaneous sensation. -2-Point discrimination test (touch discrimination) and Fugl-Meyer Assessment (hand motor recovery). Used to assess the poststroke motor recovery	Post intervention, there was a significant (P <0.004) increase up to 30% positive touch-response for the hand quadrants among the experimental group in comparison to only 13.5% rise for the same among the controls. The cutaneous threshold of the less-affected palm also improved significantly among the experimental subjects in comparison to the controls (P = .04).	MT may be considered as a feasible and effective regime for enhancing cutaneous sensibility among chronic stroke survivors.	3
69	Kim and Yim (2018)	Republic of Korea	20 stroke patients **Age** range: E=51+/-2.98 C= 74.11+/-2.88 PSD= <3 months TOS(ischaemic /haemorrhagic) E=5/3; C=7/5	high-frequency repetitive transcranial magnetic stimulation (HF-rTMS) combined with task-oriented mirror therapy (TOMT)	HF-rTMS	- Motor evoked potential (MEP) used to prognosticate corticospinal and motor capacity recovery after brain stroke. - Pinch grip and pich grip tests of both the paretic and nonparetic hands (JAMAR® Hand Evaluation Kit) - Hand motor function (Box and block tests)	Significant improvements in the MEP and hand function variables were observed in both groups (p<0.05). In particular, hand functions (pinch grip and box and block test) were significantly different between the 2 groups (p<0.05).	HF-rTMS + TOMT had a positive effect on hand function and can be used for the rehabilitation of precisehand movements in acute stroke patients.	1

188	Schick et al, (2017)	Germany	33 subjects<br><br>Age range:<br><br>Intervention group=62 (19.6 ± SD) yrs<br>Control group=63 (11.5 ± SD)yrs<br>PSD:<br>Intervention group=51 (36.4 ± SD) days<br>Control group=49 (32.8 ± SD)days<br>TOS(ischaemic /haemorrhagic)<br>Intervention group= 12/3<br>Control group=15/2	EMG-triggered electrostimulation and mirror therapy (EMG-MES and MT)	EMG-triggered electrostimulation (EMG-MES)	Fugl-Meyer Assessment for motor function<br>German language version of the Rivermead Assessment of Somatosensory Performance (RASP-DT)<br>• Box and Block Test (BBT) to assess manual dexterity of arm and hand in object manipulation<br>• Goal Attainment Scaling (GAS) to assess attain-¨<br>ment of a therapy goal previously defined with<br>the subject.<br>• Barthel Index (BI) to record and<br>assess basic everyday activities	Fugl-Meyer Assessment (p = 0.017) at a medium effect size (Cohen) of d = 0.7, due to a significant recovery of shoulder and elbow function<br>(p = 0.003) in the Fugl-Meyer Assessment Part A subtest	Additional mirror therapy in combination with EMG-triggered multi-channel electrostimulation is therapeutically beneficial for post-acute stroke patients with very severe arm/hand paresis.	**4**
**189**	Harmsen et al, 2015	Netherland	P: Home dwelling chronic stroke survivors<br>Sample size: 37 (E=18, C=19)<br>Age (Years):<br>E= 57±10.4,<br>C= 60±8.8;<br>PSD (months):<br>E=46±37,<br>C=38±25;<br>TOS(ischemic/hemorrhagic):	Mirror therapy (Action Observation – AO)	Controlled observation (CO) of static photographs of landscape.	Movement time<br><br>- 2D acceleration sensors (biaxial piezo-resistive)<br><br>- Digital recording system)	- Movement time decreased significantly in both groups: 18.3% in the AO and 9.1% in the CO group.<br><br>- Decrease in movement time was significantly more in the AO compared with the CO group (MD = 0.14 s, 95% CI=0.02-0.26; p= 0.026).	The study showed that a mirror therapy–based AO protocol contributes to motor learning after stroke.	2

			E=13/5; C=12/7						
**190**	Selles et al., 2014	Netherlands	**Sample size:**<br>G1 =17, G2 =21<br>G3 =20, G4 =18<br>G5 =17<br>**Age Range:** NR<br><br>**Mean Age:**<br>G1 = 57 ±11<br>G2 =60 ±9<br>G3 = 58 ±12<br>G4 =58 ±11<br>G5 = 57 ±11<br>**PSD(months):**<br>I**:**G1 =36 ±31<br>G2 =41 ±39<br>G3 =35±35<br>G4 =24 ±18<br>G5 =22 ±15<br>**TOS(ischemic/ hemorrhagic):**<br>NR	G1 = Reaching Exercise using Mirrow Therapy (REMT) with the paretic arm with direct view (Paretic-No Mirror); G2 = REMT with the nonparetic arm with direct view (Nonparetic-No Mirror); G3 = REMT with the nonparetic arm with mirror reflection (Non-paretic Mirror)<br>G4 = REMT with both sides and with a nontransparent screenpreventing visual control of paretic side (Bilateral Screen)	G5 = REMT with both sides with mirror reflection of the nonparetic arm (Bilateral -Mirror)	Primary: the movement time of the reaching task<br><br>Secondary: reaction time,<br>peak movement velocity, number of peaks in the velocity profile as a measure of the smoothness of the trajectory, and<br>the precision of reaching the target	All the groups showed significant improvement following the reaching exercises (p < 0.001). The largest improvement in movement time was seen in G1..<br>In contrast, the improvements in the G3 were not significantly different from G1. No significant difference was found between the groups the mirror conditions with the group with no mirror conditions. The comparison of the combined Mirror conditions (G3+G5) versus the combined no-mirror condition (G2+G4) was not significantly different (*P*= 0.102) nor was the comparison between the unimanual mirror condition (G3) and the G5 (*P* = 0.334). When comparing the change scores of the G1 and the other 4 conditions, there was no significant differences except for the peak velocity, where both bimanual conditions (G4,G5) showed significantly less improvement in peak velocity than G1	Using a mirror reflection can facilitate motor learning	2
**191**	Lin, et al, 2014	Taiwan	**Sample size:**<br>G1=14;G2=14; C=15<br>**Mean Age:**<br>G1=55.79±14.59<br>G2=56.01±12.53<br>C=53.34 ±10.12<br><br>**PSD(months):**<br>G1=22.71±13.6	G1: Mirror therapy (MT) with mesh glove (MG) afferent stimulation (MT + MG)<br><br>G2: Mirror therapy (MT) only	Conventional rehabilitation	Primary: Motor impairment (UE-FMA); muscle tone (Myoton-3 device); manual dexterity (BBT); mobility function (10MWT)<br>Secondary: ADL (MAL); self-report assessment Of UE function (ABILHAND questionnaire);	There was a significant difference between the groups in mean total FMA (*P* = .045), BBT (p = 0.019), 10MWT (and the MT groups showing significantly higher scores than the CT group for the FMA total score (MT + MG: *P* = .0032 and MT only: P = .0031). Group differences in the muscle tone were not significant. Performance on the BBT and the 10 MWT were improved after MT + MG compared with the other groups.	MT+MG improved BBT and ambulation greater than MT only. However, it showed no significant higher effect on motor impairment	3

			2<br>G2=18.50±11.61<br>C=17.80 ±10.56<br>**TOS(ischemic/ hemorrhagic):** NR			kinematic variables (7-camera motion capture system)		and muscle tone compared with MT	
**192**	Stinear, et al, 2014	New Zealand	**Sample size:** I= 29 C= 28<br><br>**Age Range:**<br>I= 33–97<br>C= 31–90<br><br>**Mean Age:**<br>I=68<br>C= 71<br><br>**PSD:** subacute stage<br><br>**TOS(ischemic/ hemorrhagic):** NR	Made device-assisted mirror symmetrical bimanual movements before upper limb physiotherapy (PRIMED)	Intermittent cutaneous electric stimulation of the paretic forearm before physiotherapy	Upper limb function (ARAT); impairment (FMA), and stroke severity (NIHSS); disability (mRS) and quality of life (SIS); Corticomotor excitability in each hemisphere and interhemispheric inhibition (TMS); Motor evoked potentials (standard surface EMG techniques); Rest motor threshold (by recording blocks of 12 MEPs)	A greater proportion of participants achieved a plateau of upper limb function by 12 weeks in the PRIMED group than the CONTROL group for both the intention-to-treat (*P*=0.039) per-protocol analyses (*P*=0.046). At 26 weeks, there were no between-group differences in median modified Rankin Scale score (*P*>0.4) or mean Stroke Impact Scale score (*P*>0.2). PRIMED participants had greater rebalancing of corticomotor excitability than controls at 12 and 26 weeks and interhemispheric inhibition at 26 weeks (all *P*<0.05).	Bilateral priming accelerated recovery of upper limb function in the initial weeks after stroke.	3
**193**	Wu et al, 2013	Taiwan	**Sample size:** I=16; C=17<br>**Mean Age:**<br>**I=**54.77±11.66<br>C =53.59±10.21<br>**PSD(months):**<br>I= 19.31±12.57<br>C = 21.88±15.55<br>**TOS:ischemic/ hemorrhagic :**<br>I=10/6; C=10/7	Upper extremity training with mirror therapy (MT)	task-oriented upper extremity training	Motor performance (FMA and kinematic analysis); ADL (MAL and ABILHAND); sensory function (Revised Nottingham Sensory assessment). Reaction time, normalized total displacement, maximum cross correlation of the shoulder and elbow (kinematic analysis)	The results of the FMA and kinematic variables showed significant group effects favoring the MT group on the FMA Total (p = 0.009) and distal part (P=.041). Kinematic results displayed significant and large effects favoring MT on reaction time (p = 0.037), normalized total displacement (P=.042), and maximum cross-correlation between the shoulder and elbow (P=.029). No significant group differences were found on the MAL and ABILHAND in posttreatment and follow-up	MT might improve movement performance, motor control, and temperature sense, but may not translate into daily functions in stroke survuvors.	3
**1**	Thi	Swizerl	**Sample size:**	G1 = Individual	Therapy	PO: impairment level	ARAT and FMA scores increased	There is a	3

**94**	eme et al., 2013	and & Germany	G1=18;G2=21; C=21<br>**Mean Age**:<br>G1 = 63.8+12.1<br>G2 = 69.1+10.2<br>C=68.3+8.9<br>**PSD** (Days):<br>G1=47.6+25.8<br>G2 = 36.2+21.1<br>C=51.4+22.5<br>**TOS(**Ischemic/hemorrhagic):<br>G1= 14/1<br>G2 = 11/8<br>C = 11/10	mirror therapy<br><br>G2 = Group mirror therapy	with Restricted view on the affected arm	(motor score of the Fugl-Meyer Test-arm section(FMA)), functional level (Action Research Arm Test(ARAT))<br><br>SO: ADL(Barthel Index (BI)), self reported influence of stroke related function on quality of life (Stroke Impact Scale (SIS)), somatosensory, pain and ROM (Fugle-Meyer Test), Spasticity (mAS), visiospatial neglect (Star Cancellation Test)	significantly in all patients over time but there were no significant group differences. No significant interaction between groups were detected in the FMA scores for the arm, hand and fingers. There were a significant change in BI, SIS and somatosensory subscore of FM for all participants but no significant between group changes. The PROM and pain scores significantly decreased for all participants. There is a significant group interaction for the visiospatial measure. ROM of both groups improved significantly over time for the finger flexors (*P* < 0.001) but not for wrist flexors (*P* = 0.08). A significant group difference in ROM was also seen between both intervention groups indicating higher scores for individual mirror therapy after the intervention (*P*< 0.05). No significant group interaction was found for wrist flexors (*P* = 0.82)	positive effect on visiospatial neglect with mirror therapy but no effect on seonsorimotor function of the arm, ADL, QOL, MAS and ROM	
**39**	Burgar et al, (2011)	USA	**Sample size: I:** G1=19; G2=17; C=18<br>**Mean Age I:**<br>G1= 62.5 ± 2.0<br>G2 = 58.6 ± 2.3<br>C=68.1 ± 3.3<br>**PSD(days): I:**<br>G1= 17.3 ± 2.7<br>G2 = 16.6 ± 2.4<br>C=10.6 ± 1.2<br>**TOS:** I =NR<br>C = NR	G1 = 15 hours robot-assisted (RA) upper-limb therapy with the Mirror Image Movement Enabler (MIME)<br>G2 = 30 hours robot-assisted (RA) upper-limb therapy with the Mirror Image Movement Enabler (MIME)	15 hours of additional conventional therapy in addition to usual care	The primary outcome: upper extremity functions (Fugl-Meyer Assessment (FMA)). Secondary outcomes: ADL (Functional Independence Measure (FIM)), spasticity (Modified Ashworth Scale), and upper limb functions (Wolf Motor Test)	Gains in the primary outcome measure were not significantly different between groups at 6 months. The high-dose group had greater FIM gains than controls at discharge and greater tone but no significantdifference in FIM changes compared with low-dose subjects at 6 months. As used during acute rehabilitation, motor-control changes at follow-up were no less with MIME than with additional conventional therapy.	Improved shoulder and elbow function and impairment measures following RA training can be at least equivalent to conventional, labor-intensive methods.	2

**195**	Michielsen et al, (2011)	Netherland	**Sample size: I=** 20 C= 20<br><br>**Age Range:** I= NR C= NR<br>**Mean Age:**<br>**I=** 55.3 ± 12.0<br>C= 58.7 ± 13.5<br><br>**PSD(years):**<br>I= 4.7 ± 3.6 C= 4.5 ± 2.6<br><br>**TOS(ischemic/ hemorrhagic):**<br>I = 14/6 C = 14/6	Homebased mirrow therapy with the affected hand positioned behind the mirror while they looked at the reflection of the unaffected hand in the mirror.	Home based mirrow therapy with a direct view of both hands,	The primary outcome: was motor function using the Fugl-Meyer motor assessment (FMA). Secondary: grip force with a Jamar handheld dynamometer, spasticity with the Tardieu scale, and pain with a visual analog scale ranging from 0 to 100 mm, motor capacity with the Action Research Arm Test, self-perceived performance with the ABILHAND questionnaire, actual performance in daily life during 24 hours with the Stroke-ULAM and quality of life with the EQ-5D.	Posttreatment, the FMA improved more in the mirror than in the control group ($P < .05$), but this improvement did not persist at follow-up. No other ithin nor between group changes were found on the other outcome measures (all $P > .05$). fMRI results showed a shift in activation balance within the primary motor cortex toward the affected hemisphere in the mirror group only	This trial showed some effectiveness for mirror therapy in improving the motor function of chronic stroke patients and is the first to associate mirror therapy with cortical reorganization.	3
**196**	Cacchio et al, 2009	Italy	**Sample size:**<br>I=24<br>C=24<br><br>**Age Range:** NR<br><br>**Mean Age:**<br>I=57.9 ± 9.9<br>C=58.8 ± 9.4<br><br>**PSD(months):**<br>I=5.1 ± 2.5<br>C=4.9 ± 2.8<br><br>**TOS:**<br>I = 18/6 C	conventional stroke rehabilitation program with additional mirror therapy program consisting of unaffected upper limb movements	conventional stroke rehabilitation program only	**Primary outome**: Pain (VAS); Tactile allodynia (brush movement);<br>**Secondary outcomes**: Functional Ability (FA) and mean performance time (mPT) value (both are WMFT items]); Quality of Movement (MAL]); ADL (Motor Activity Log [MAL]).	Only the intervention group showed significant improvement in pain both at rest and movement and in tactile allodynia at post intervention and at 6 months follow up ($p < 0.001$). There was significant between group difference in pain and allondynia at post intervention but only the difference in pain was maintained at follow-up. The intervention group showed significant improvement in FA, PT and MAL at post intervention and follow-up; control group also showed similar improvements except for FA post intervention and MAL. There was also significant between group difference in FA, PT and	The results indicate that mirror therapy effectively reduces pain and enhances upper limb motor function in stroke patients with upper limb Complex regional pain syndrome	2

			=17/7				MAL at post intervention and follow up (p <0.001)	type 1	
**197**	Dohle et al, 2009	Germany	**Sample size:** I=18; C=18 **Mean Age:** I=54.9 +13.8 C=58.0 +14.0 **PSD:** NR **TOS(ischemic/ hemorrhagic):** NR	Standard therapy with Mirrow therapy	Standard therapy with direct view of the affected arm (no mirror)	Upper extremity motor functions (Fugl-Meyer subscores for the upper extremity); hemineglect (several subtests of the Behavioral Inattention test [BIT]); motor impairment (Action Research Arm test [ARAT]); ADL (Functional Independence Measure (FIM))	Regarding motor function, there was no significant therapy effect in both groups; however, Regarding nonmotor symptoms of Fugl-Meyer Scale, improvement of surface sensibility (light touch) was significantly different between the 2 treatment groups. hemineglect score was significantly greater in the MT group than in the CT group (P = .005). there was no withing group nor between group difference in ADL.	MT early after stroke is a promising method to improve sensory and attentional deficits and to support motor recovery in a distal plegic limb	5
**198**	Yavuzer et al, 2008	Turkey	**Sample size:** I= 17; C=19 **Age Range(yrs):** I= 49–80 C= 43–79 **Mean Age (yrs):** I=63.2+9.2 C= 63.3+9.5 **PSD:** I=5.4+2.9 C=5.5+2.5 **TOS(ischemic/ hemorrhagic):** I = 14/3; C =15/4	Rehabilitation with mirror therapy (MT)	Conventional rehabilitation with sham morrow (i.e. nonreflecting side of the mirror)	motor recovery (Brunnstrom stages); spasticity (Modified Ashworth Scale [MAS]), and hand-related functioning (the self-care items of the FIM instrument)	Between-group differences were significant for motor recovery and functioning scores not only at posttreatment but at the 6-month follow-up as well with the intervention group showing significant more improvement in both outcomes (p = < 0.001) than the control group. No significant differences were found between the groups for the MAS (*P*=.904).	MT was more beneficial in terms of motor recovery and hand-related functioning than a similar treatment without mirroring.	3
**199**	Sütbeyaz, et al, 2007	Turkey & Netherlands	**Sample size:** I= 20; C= 20 **Mean Age (yrs):** I=62.7+9.7 C= 64.7+7.7 **PSD(months):**	Traditional rehabilitation with Mirror therapy Program (MT)	Traditional rehabilitation with Sham mirror therapy	Motor recovery (Brunnstrom stages), spasticity (Modified Ashworth Scale [MAS]), walking ability (Functional Ambulation	All assessed outcome parameters improved significantly in both groups after the treatment and continued to improve at follow-up. At follow up, the intervention group showed greater improvement than the control in Brumstorm stage (*P*=.002), as well as the	MT enhances lower-extremity motor recovery and motor	3

			I=3.5±1.3<br>C= 3.9±1.9<br>**TOS(ischemic/ hemorrhagic):**<br>1=16/4; C = 17/3		Program	Categories [FAC]), and motor functioning (motor items of FIM instrument).	FIM motor score (*P*=.001). Neither MAS (*P*=.102) nor FAC (*P*=.610) showed significant differences between the groups.	functioning in subacute stroke patients.	

Mental Practice

Ref	Study (Authors, year	Country	Participants	Interventions/ Model of care	Control/ comparison	Outcomes assessed/ outcome measures	Results	Conclusion	Quality score
**200**	Aben, et al, 2014	Netherlands	**Sample size:** I= 57 C= 58<br><br>**Age Range:** NR<br><br>**Mean Age:** NR<br><br>**PSD(months):** NR<br><br>**TOS(ischemic/hemorrhagic):**NR	Enrolled in a Memory Self-efficacy (MSE) training Program with a strong focus on psychoeducation	Enrolled in a peer support group program: were educated about causes and consequences of stroke and shared their problems with peers.	Memory Self-efficacy (MSE) (3 subscales of the Metamemory in Adulthoodquestionnaire); Depression (CES-D); QoL (WhoQol-Bref & the VAS of the Euroqol-5D); Social participation (Social Support List); verbal memory capacity (the Dutch version of the Auditory Verbal Learning Test (AVLT) and Story Recall of the Rivermead Behavioural Memory Test (RBMT)); Problem experience (abbreviated version of the Impact on Participation and Autonomy)	MSE improved significantly over the intervention period in the experimental group compared with the control group (*P* = .010), Depression scores declined in both groups, but group difference did not reach significance (*P* = .148). Psychological QoL improved more in the experimental than in the control group, but this result was not statistically significant either (*P* = .077). Other quality of-life measures did not differ between groups. Participants in both groups reported a decrease of problem experience (*P* = .014) over the follow-up period of 12months, but no differences between the experimental and control groups were found. Self-reported strategy use remained unchanged in either group (*P* = .934). No differences in social participation were found between the experimental and control groups over time (*P* = .341)	An MSE training program improved MSE and psychological quality of life in stroke patients	3
**201**	Timmermans et al., 2013	Netherlands	**Sample size:** I=8 C=10<br><br>**Age Range:** I=NR C= NR<br><br>**Mean Age:** **I=**59.7 ±7.3 C= 58.7 ±9.6	Performed video instructed mental practice (MP)	neurodevelopmental therapy based exercise therapy	ADL (Barthel index [BI] and the Frenchay activities index [FAI]); Cognitive functioning (Cognitive Log [CogLog]); ability to imagine motor acts (Vividness of Movement Imagery Questionnaire [VMIQ]); Upper extremity functioning at the ICF activity level (arm motor	Both the experimental and control groups showed significant improvements in BI over time at 6- and 12-month follow-up, respectively, as compared with baseline [control: P = .034 (6 months) and P = .018 (12 months); experimental: P = .011 (6 months) and P = .014 (12 months). For both the BI and the FAI, no differences between groups were Found. There is a significant within-group improvement over time in both the control (P = .000) and the experimental group (P = .000) on FMT. Patients improved significantly over time on the results of the FAT, in both the control	Both video instructed mental practice (MP) and neurodevelopmental therapy based exercise Therapy	3

			**PSD(days):**<br>I= 36.1 ±27.4<br>C= 32.3 ±17.9<br><br>**TOS: ischemic/hemorrhagic :**<br>NR			function section of the Fugl-Meyer test<br>(FMT), the Frenchay arm test [FAT], and accelerometry [CC])	group (P = .037) and the experimental group (Friedman test P = .000). However, only in the experimental group, a significant improvement on the FAT was found after Training (P = .004) and between cessation of the training and the 12-month follow-up measurement (P = .026). For the accelerometer data, no significant improvements over time were found, apart from the increment of the ratio affected/nonaffected arm in the control group between baseline and after training (P = .045). No significant differences were found between the improvements of the control and the experimental group on the FAT, or on any of the subdomains (FAS, PT, and two strength-based tasks) of the WMFT. No between groupdiference was foun in any of the measured outcomes.	provide equal improvement in motor and functional abilities post stroke.	
**149**	Nilsen, et al, 2012	New York	**Sample size:** G1=5;G2=6; C=6<br>**Age(yrs)**:<br>G = 46.6 ±5.2<br>G2=62.0 ±5.7<br>C= 66.2 ±2.6<br>**PSD** (month):<br>**G1=**33.5±15.1<br>G2=43.2 ±15.4<br>C = 20.3 ±8.8<br>**TOS:**Ischemic/hemorrhagic: NR	G1 = occupational therapy with mental practice training in functional tasks (OT+MP) using an internal Perspective<br>G2 = OT+MP using an external perspective	Occupational therapy with relaxation imagery training	PO: impairment (The upper extremity section of the Fugl-Meyer Assessment (FMA) of Motor Recovery), hand function in ADL (Jebsen–Taylor Test of Hand Function [JTTHF]).<br>SO: self-perception of occupational performance and satisfaction with performance (The Canadian Occupational Performance Measure (COPM))	Mean FMA scores increased from pretest to posttest across all the groups, but significantly only for G1 and there was a group x time interaction (p = 0.042). G1 also has a higher improvement in the JTTHF compared to the other groups (p = .036). COPM scores increased significantly across all the groups (p < .001)	OT+MP using either the internal or the external imagery perspectives, reduced impairments and improved function above a control condition.	3

**202**	Rico et al, 2010	Italy	**Sample size:** I=18 C=18<br><br>**Age Range:** I= 32 – 75 C= 34 – 75 **Mean Age:** I= 60.06± 11.68C= 60.17 ± 11.67<br><br>**PSD(weeks):** I= 7.44 ± 2.41 C= 7.33 ± 2.38<br><br>**TOS:** ischemic	Mental practice then conventional neurorehabilitation (MP-NR)<br><br>NB: randomized crossover trial	Conventional neuro-rehabilitation then mental practice (NR-MP)	Motor strength (upper limb of the motoricity index [MI]) Motor ability [MA] (Arm Functional Test). Quality of movement [QoM] (Functional Ability Scale [FAS]); Speed of Execution [SoE]	After three weeks of treatment before the crossover, there was a significant difference in MA between the groups favoring MP-NR. At six weeks after the crossover interventions, a significant difference in mean MI was seen between the two groups, favoring MP-NR (p = 0.002). At the 3 weeks also, in favor of MP-NR; there was a significant difference between the two groups in the QoM (p = 0.001) while a significant difference was seen in the SoE (p = 0.002) favoring NR-MP. Also there was a significant improvement in strength, QoM and SoE in both groups, more in MP-NR at week three and in the control group at week 6, which both coincides with the periods both groups were receiving mental practice therapy.	Three weeks mental practice coupled with conventional neurorehabilitation in the post-acute setting can significantly contribute to the recovery of motor performance of upper limbs in stroke patients.	2
**203**	Page et al, 2005	USA	**Sample size:** I= 6 C= 5 **Mean Age:** NR **PSD:** NR **TOS:** NR	Conventional therapy plus mental practice (MP) of the ADLs	Conventional therapy only	ADL (MAL: AOU and QOM); fine motor functions (ARAT);	The intervention group showed significant improvement in the AOU and QOM domain of MAL aand also in ARAT scores. In contrast, the controls showed nominal increases in the amount they used their affected limb and in limb function. the intervention group also showed significantly greater improvement in ARAT score (*P*=.004)	MP may increase stroke patient's use of more affected limb and motor functions.	2
**204**	Liu, et al, 2004	Hong Kong	**Sample size:** I= 26; C= 20 **Age (yrs):** I= 71.0±6.0 C= 72.7±9.4 **PSD(months):** I= 12.3±5.3 C= 15.4±12.2 **TOS (ischemic/he**	mental imagery program (MIP)	conventional functional training (FT)	Performance on new task (7-point Likert scale); sustained visual attention and visual scanning (Color Trails Test23 (CTT)); upper-extremity motor function, lower-extremity motor function, and sensation (3 subtests of the Fugl-Meyer)	MIP reached a significantly higher level of performance on the trained tasks FT after completing both weeks 2 and 3 of the training. MIP also reached a significantly higher level of performance on the untrained tasks tested at the end of the training program (p<001). MIP also reached a significantly higher level of performance on the trained tasks at the 1-month follow-up (*P*<.001). For the cognitive and sensorimotor abilities, the repeated-measure MANOVAs showed no significant differences in CTT and FMA subscale scores between the 2 groups	Mental imagery can be used as a training strategy to promote the relearning of daily tasks for people after an acute stroke.	3

			**morrhagic):** NR				($P<.28$). However, MIP showed significantly greater improvement in CTT subscale scores across time than FT		

Task-Oriented Training (TOT)

Ref	Study (Authors, year	Country	Participants	Interventions/ Model of care	Control/ comparison	Outcomes assessed/ outcome measures	Results	Conclusion	Quality score
205	Khumsapsiri et al, (2018)	Thailand	16 stroke patients<br>Age (yrs):<br>E=61.00 ± 10.59<br>C=57.50 ± 9.79<br>PSD (yrs):<br>E= 1.43 ± 1.00<br>C=2.46 ± 2.56<br>TOS (ischaemic/ haemorrhagic)<br>E=5/3; C= 6/2	multidirectional reach training	Conventional physical therapy	-Limits of stability (LOS), was used as a laboratory measurement of balance using the Balance Master (NeuroCom International Inc., Clackamas, OR, USA)<br>-Weight-bearing squat was used to determine weight distribution of participants.<br>-Fullerton Advanced Balance scale was used as a clinical balance measurement tool	At 1month follow-up, weight-bearing squat of affected side at 0°, mean difference (95% CI) = 0.14 (0.22 to 0.04), p = .006, and 90°, mean difference (95% CI) = 0.13 (0.23 to 0.03), p = .008, was significantly increased as compared to control<br>group. There were no statistically significant differences in the other outcome measures between the two groups.	training using a new multidirectional reach tool is effective for improving balance in individuals with stroke	1
206	Jonsdottir et al. (2017)	Italy	68 patients<br>Age range:<br>E= 36.5–84.8yrs<br>C =38.0–88.7yrs<br>PSD: NR	Experimental (MeCFES assisted TOT (M-	Conventional rehabilitation care including	-Action Research Arm Test (ARAT), [N/S]<br>- Upper Extremity Fugl-Meyer Assessment (FMA-UE)	Considering subacute subjects (time since stroke < 6 months), there was a trend for a larger proportion of improved patients in the M-TOT group following rehabilitation (57.9%) than in	MeCFES can be a safe adjunct to rehabilitation that could promote recovery of upper	3

			TOS (haemorrhagic /ischaemic) E =5/26; C=7/29	TOT)	TOT (CTOT).	-Disability of the Arm Shoulder and Hand questionnaire *[Scale NS]*	the C-TOT group (33.2%) (difference in proportion improved 24.7%; 95% CI -4.0; 48.6),	limb function after stroke,.	
207	Jung et al. (2017)	Korea	46 stroke survivors<br>Age (years):<br>E=54.8 ± 8.13<br>C=57.3 ± 9.06<br>PSD (month):<br>E=13.3 ± 5.95<br>C=14.5 ± 6.32<br>TOS(ischaemic /haemorrhagic)<br>E=12/11;C=15/8	Task-Related Training (TRT) + Transcutaneous Electrical Nerve Stimulation (TENS)	TRT+ placebo TENS (TRT+PLBO)	-Integrated EMG (IEMG),<br>- A digital manual muscle tester for muscle strength, active range of motion (AROM)<br>-FuglMeyer Assessment of the upper extremity (FMA-UE) used to assess upper extremities function	Significantly greater improvement in muscle activation (wrist extensors, P = 0.045; elbow extensors, P = 0.004), muscle strength (wrist extensors, P = 0.044; elbow extensors, P = 0.012), AROM (wrist extension, P = 0.042; elbow extensors, P = 0.040) and FMAUE (total, P < 0.001; shoulder/elbow/forearm, P = 0.001; wrist, P = 0.002; coordination, P = 0.008) at the end of intervention.	TRT Combined with TENS can improve paretic muscle activity in upper limb paresis, highlighting the benefits of somatosensory stimulation from TENS	**4**
208	Folkerts et al. (2017)	Netherlands	11 participants<br><br>Age range:35 to 73 years<br><br>PSD: N/S<br><br>TOS= both ischaemic and haemorrhagic combined [N/S]	Eccentric strength training and then four weeks of task-oriented strength training (EST-TOST)	Vice versa (TOST-EST).	-Strength and upper limb function were administered with a handheld dynamometer (HHD)<br>-Action Research Arm Test (ARAT) was used to measure upper limb function<br>-Feasibility was evaluated with the Intrinsic Motivation Inventory (IMI)	Significant increases were found in ARAT score (mean difference 7.3; p < 0.05) and in shoulder and elbow strength (mean difference respectively 23.96 N; p < 0.001 and 27.41 N; p < 0.003).	A combination of eccentric and task-oriented strength training is an effective and feasible training method to increase function and strength in individuals with chronic stroke.	**3**
**209**	Willigenburg et al. (2017)	USA.	34 subjects<br>**Age range**:<br>Both groups= 53.5 ± 5.35yrs<br>**PSD**:<br>Both groups=61.7 months<br>**TOS**= ischaemic	Combined repetitive task-specific practice (RTP) with a myoelectric brace (RTP	RTP only	Stroke Impact Scale (SIS)<br><br>Upper Extremeties reaching kinematics<br><br>[the uses were not supplied]	Subjects in the RTP CMyomo group showed greater improvements on all SIS subscales, with the recovery scale reaching statistical significance (p = .03). Subjects in the RTP-only group showed a greater increase in hand velocity in the reach up task (p = .02	RTP CMYomo may be more beneficial than RTP only in improving self-reported function and perceptions of overall recovery	3

				CMyomo)					
**210**	Carrico et al, (2016)	USA	36 subjects<br>Age range,yeras:<br>E=58.7±12.1<br>C=65.4±10.8<br>PSD, months:<br>E=39.2±34.6<br>C=25.7±17.7<br>TOS(I/H)<br>E=11/6; C=15/3	Peripheral Nerve Stimulation (PNS)	Sham stimulation	-Fugl–Meyer Assessment was used to assess upper extremity movement function<br>-Wolf Motor Function Test (WMFT)<br>-Action Research Arm Test (ARAT)	Sign btwn-grp diff favored PNS on Fugl–Meyer at post intervention (95% CI, 1.1–6.9;P=0.008) and 1-month follow-up (95% CI, 0.6–8.3; P=0.025),<br>WMFT at post intervention (95% CI, −0.21 to −0.02; P=0.020), and ARAT at post intervention (95% CI, 0.8–7.3; P=0.015) and 1-month follow-up (95% CI, 0.6–8.4; P=0.025).	PNS paired with intensive task–oriented training can effect significant improvement in impaired upper extremity movement function.	4
**211**	Kim et al, 2016	South Korea	P: Chronic Stroke;<br>Sample size = E/C=10/10<br>Mean age (yrs): E 48.90 ± 10.12, C= 47.50±14.43;<br>PSD (months): E= 10.90 ± 4.80, C= 15.40 ± 4.69;<br>TOS(I/H):<br>E = 4/6, C =5/5	Task Oriented training (TOT) + EMG-stim	Electromyogram-triggered neuromuscular stimulation (EMG-stim) only	Muscle Activity(EMG)<br>Dexterity<br>(Box and block Test)<br>Muscle Recovery (Fugl-Meyer assessment (FMA)	- TOT + EMG-stim exhibited significant improvement relative to the EMG-stim only in muscle activation, motor recovery and dexterity (p>0.05).	-EMG-stim + TOT may provide more positive effects on the affected arm in chronic stroke patients than EMG-stim alone.<br>-TOT could enhance the therapeutic effects of EMG-stim by active task-induced motor learning	2
**212**	Hubbard et.al, 2015	Australia	P: Individuals with acute first ever ischaemic stroke<br>Sample size=23 (E=11, C=12);<br>Mean age (Years): E= 61.7±13.4, C= 69.3±9.4;<br>PSD: ≤1 week<br>TOS(ischemic/hemorrhagic): Ischaemic only	Intensive Training (Additional 30 hours of task-specific UL training to Standard Therapy (IT-UL)	Standard Therapy (Averages of 6 sessions, 31.5mins of OT & PT for 11.5 days) (ST)	- Change in brain activation (FMRI), - UL motor function (UL-MAS & mRS)	- When compared with the standard-care group, the intensive-training group had increased brain activation in the anterior cingulate and ipsilesional supplementary motor areas and a greater reduction in the extent of activation (P =0.02) in the contralesional cerebellum,<br>- Intensive training was associated with a smaller deviation from mean recovery (UL-MAS) at 1 month (Pr>F0 = 0.017) and 3 months (Pr>F = 0.006), indicating more consistent and predictable improvement in motor outcomes	Early, IT-UL was associated with greater changes in activation in putative motor(supplementary motor area and cerebellum) and attention (anterior cingulate) regions, providing support for the role of these regions and functions in early recovery poststroke.	3
213	Gharib et al,	Egypt	Sample size= 40, (E=20, C=20);<br>Mean age (yrs):	E= Repetitive task	C= Repetitive task	- ROM of fingers abduction and extension (3D	- E showed a significant improvement as compared with C. MAS score was 4.25±0.63 for E and 3.35±0.74 for C (*t*=-	Repetitive task practice therapy combined with	3

	2015		E=54.85±6.41, C= 54±6.23; Age range (yrs): 45-65 years; PSD (months): E= 10.95±4.81, C= 11.40±4.60; TOS(ischemic/hemorrhagic): E =14/6; C=15/5	practice + Electrical Stimulation	practice + sham Electrical Stimulation	Motion Analysis)<br><br>- Hand motor function (MAS) and<br><br>- Hand skills (Jebsen Taylor Test)	3.50, *p*= 0.0001).<br><br>- Time to complete Jebsen Taylor Test was 180.90±7.04 for E and 192.80±6.87 for C (*t*=4.50 and *p*= 0.0001).<br><br>Significant improvement in fingers abduction and extension in both groups (in favor to E).	electrical stimulation can improve skilled hand performance in terms of hand motor function, skills and range of motion in stroke patients	
214	Cruz et al., 2014	Portugal	**Sample size:** I=22; C=21 **Age (yrs):** I=64.9±12.0 C= 68.2±14.3 **PSD (days):** I=7.5 ±7.9 C= 6.2 ± 6.8 **TOS:** ischemic	Received RMT first and then, active comparator -3D motor characterization (3D-MC) *[Randomizedcross-over trial]*	Received 3D-MC first, and then repetitive motor task under vibratory feedback (RMT)	Number of correct movements per minute (a hand-to-mouth task)	The intervention session produced greater significant improvement in the number of movements made on the patient's affected side compared to the comparator session (P< 0.001). This difference corresponds to a relative increase average of 2.8 more correct movements per minute in the experimental session (P< 0.001)	Repetitive motor task under vibratory feedback may improve the efficacy of training on motor re-learning processes after stroke.	2
**20**	Lemmens et al., 2014	Belgium & Netherland	**Sample size:** I= 8; C= 8 **Age (yrs):** I=63.5; C= 55.0 **PSD(months):** I=12.5; C=25.5 **TOS (ischemic/ hemorrhagic):** NR	task-oriented robot-supported arm-hand training	task-oriented arm-hand training without robot support	Amount of arm use (Fugl-Meyer Motor Assessment [FMMA], Action Research Arm Test [ARAT] and Motor Activity Log [MAL])	Both groups showed no significant improvement in the amount of arm use (p > 0.05) from all the measured outcomes	There is no significant changes in actual amount of arm-hand use after task-oriented training, with or without robot-support	3
**24**	Timmermans et al., 2014	Netherlands	**Sample size:** I= 11; C= 11 **Mean Age yrs:** I=61.8±6.8 C= 56.8±6.4 **PSD(years):** I=2.8±2.9 C= 3.7±3.0	task-oriented robot-assisted arm-hand training (TORAT)	task-oriented non-robotic arm-hand training (TONAT)	Arm hand function (Fugl Meyer Motor Assessment (FMMA)), upper limb activity (Action Research Arm Test (ARAT)); arm hand activity (Motor Activity	No between group differences were found in FMMA, ARAT, and MAL. Both groups did not show any significant improvement in FMMA, but. a significant improvement in ARAT occurred in TORAT (p = 0.008); there was a significant improvement in MAL for TONAT (p = 0.008), and TORAT (p =	Arm hand performance improved in chronic stroke patients, after eight weeks of task oriented training with or without robot assistance.	3

			**TOS(ischemic/ hemorrhagic):** NR			Log (MAL)); quality of life (EuroQol-5D and SF-36).	0.005). A significant difference was found between TONAT and TORAT with regard to the delta physical health scores of the SF-36 T2-T3 (p = 0.002).		
**215**	Kim, et al, 2013	USA & South Korea	**Sample size:** I=15; C= 15 **Mean Age Yrs:** I= 63.3 + 8.30 C= 60.9 + 10.64 **PSD(years):** I=13.6 + 4.36 C=12.3 + 5.26 **TOS (ischemic/ hemorrhagic):** NR	Task related training (TRT) with transcuteno us electrical nerve stimulation (TENS)	TRT+ placebo TENS	Upper extremity motor recovery (FMA); motor function (manual function test [MFT]); promptness in upper limb and hand coordination (Box and Block Test[BBT]); spasticity (MAS)	Both groups significantly improved in the three arm function measures: FMA, MFT and BBT (p < 0.05). There was also significant difference between the two groups favoring the intervention group in FMA (p = 0.034), MFT (0.037) and BBT (p = 0.042). The intervention group also showed reduced spasticity (p = 0.011) while the control group showed no improvement.	TRT +TENS can reduce motor impairment and improve motor function in stroke survivals with chronic upper limb impairment	2
**216**	Shaughness, et al, 2012	USA	**Sample size:** I : 57; C=56 **Mean Age** (Years): NR **PSD** : NR **TOS**: NR	6-month task oriented treadmill training program	Supervised stretching movements of major muscle groups on a raised mat table.	Attitudes and beliefs regarding exercise (Short Self-efficacy and Outcomes Expectations for Exercise); Physical activity (Yale Physical Activity Survey) participation (Stroke Impact Scale)	Regardless of group, all study participants experienced increased self efficacy (p=0.09) and outcome expectations for exercise (p<0.001), and improvements in activities of daily living as reported on the Stroke Impact Scale (p=0 .002). No statistically significant between-group differences were noted in any of the outcomes.	Theoretically based interventions should be tested to clarify the role of motivation and potential influence on exercise and physical activity in the post-stroke population.	2
**217**	Verma et al, 2011	India	**Sample size:** I = 15; C = 15 **Age** (Years): I = 53.27 ± 8.53 C = 55.07 ± 6.80 **PSD** (weeks): I = 6.60 ± 3.20 C = 6.07 ± 3.30 **TOS**:**(**ischemic /hemorrhagic): I =12/3; C=11/4	Task-oriented circuit class training (TOCCT) with motor imagery (MI)	Dose-matched standard training program based on the Bobath's neurodevelopmental technique,	Walking ability (Functional Ambulation Classification [FAC]); Arm movement during walking (the Rivermead Visual Gait Assessment (RVGA)); Gait parameters (in print on a paper walkway); walking speed (10 meters walk tests); and walking capacity (6-minute walk test (6MWT)).	Statistically significant differences were observed in changes between the groups at post and follow-up assessment for FAC, RVGA, walking speed, and 6MWT (*P* < 0.05).	TOCCT with MI produced statistically significant and clinically relevant improvements in the gait and the gait-related activities.	3
**84**	Wang et al.,	Taiw	**Sample size:** I = 12; C = 12	Repetitive transcranial	Sham rTMS	PO:: Corticomotor excitability symmetry	Decreased interhemispheric asymmetry of the amplitude of the MEP was noted after	rTMS enhances the effect of task-	5

	2012	an	**Age** (Years):<br>I =64.90 ± 12.37<br>C=62.98 ± 10.88<br>**PSD** (days):<br>I= 1.84 ± 1.16<br>C= 2.00 ± 1.23<br>**TOS**: NR	magnetic stimulation (rTMS) followed by Task-Oriented Training (TOT)	followed by TOT	(Motor evoke potential, MEP) and gait symmetry.<br>SO: Motor control (FMA) and spatial and temporal parameters of gait (GAITRite system)	rTMS and taskoriented training. Improvement in spatial asymmetry of gait was comparable with increased symmetry in interhemispheric excitability in the intervention group. Motor control and walking ability were also significantly improved after rTMS and task-oriented training.	oriented training in those with chronic stroke, especially by increasing gait spatial symmetry and corticomotor excitability symmetry.	
**2 1 8**	Yang, et al, 2007	Ta iw an	**Sample size:**<br>I= 13; C= 12<br>**Age Range yrs:**<br>I= 47-76;<br>C=45-80<br>**Mean Age Yrs:**<br>I=59.46±11.83<br>C= 59.17±11.98<br>**PSD(years):**<br>I=4.08±3.13<br>C= 4.68±7.40<br>**TOS(ischemic/hemorrhagic):**<br>1 = NR; C = NR	Dual-task based ball exercise program	No therapy	Gait parameters: walking speed, cadence, stride time, stride length, and temporal symmetry index [TSI] (GAITRite system)	After the ball exercise training, significant improvement was found in all selected gait variables except for TMI under both task conditions. However, improved gait performance was not shown in the control group. The between-group comparisons also revealed significant differences btwn the 2groups for all selected gait variables except for TMI under both task conditions	The dual-task– based exercise program is feasible and beneficial for improving walking ability in subjects with chronic stroke.	3
**2 1 9**	Higgins et al., 2006	Ca na da	**Sample size:**<br>I=47; C= 44<br>**Mean Age:**<br>I=73±8<br>C= 71±12<br>**PSD(months):**<br>I=217±73<br>C= 239±83<br>**TOS(ischemic/hemorrhagic):**<br>1 = 36/11<br>C = 40/4	Practice of functional, unilateral and bilateral tasks designed to improve gross and fine manual dexterity	Walking tasks.	PO: Walking capacity (6MWT); gross manual dexterity (The Box and Block Test);<br>SO: fine manual dexterity(The Nine-Hole Peg Test); activity performance of the arms (TEMPA);<br>Grip strength (dynamometer);<br>Mobility (UE-STREAM);<br>performance in self-care and mobility (BI);<br>IADL (OARS-IADL);<br>Health-related QoL (SF-36); Depression	The intervention group improved in grip strength by an average of 0.5 kg more than the mobility group members. However, this improvement was not significant. No difference was also seen in all the measured outcomes between the groups.	A task-oriented intervention did not improve voluntary movement or manual dexterity of the affected arm in people with chronic stroke	3

						(GDS)			
**220**	Michaelsen et al, 2006	Canada	**Sample size:** I= 15 C= 15<br><br>**Age Range:** NR<br><br>**Mean Age:**<br>I= 68.9 ±10.3<br>C= 69.4±10.8<br><br>**PSD(months):**<br>I=16.7±9.1<br>C= 18.2±10.7<br><br>**TOS:** NR	progressive object-related reach-to-grasp training with prevention of trunk movements	progressive object-related reach-to-grasp training with no restraint on trunk movements	PO: Motor impairment (FMA); motor function (UE- Performance Test (TEMPA)),<br>SO: Isometric force and manual dexterity (BBT); Isometric force (dynamometer); kinematics: trunk displacement and elbow extension ranges; also, range of shoulder flexion, peak arm tangential velocity, smoothness and hand trajectory straightness (Optotrak 3010 system)	Both groups improved in FMA and TEMPA with the intervention group showing greater significant decreases in impairment (*P*<0.035) and improvements in function (*P*<0.05); no significant between-group differences in these outcomes. Kinematic analysis revealed that the intervention group showed decreased mean trunk displacement, however, Changes in trunk displacement between groups were not significantly different. Unlike the control group, increase in trunk restraint elbow extension was significant (*P*<0.02). There were no between-group differences, but both groups improved elbow strength (*P*<0.002), BBT (*P*<0.01), peak velocity (*P*<0.002), trajectory smoothness (*P*<0.001) and straightness (*P*<0.01).	Treatment should be tailored to arm impairment severity with particular attention to controlling excessive trunk movements if the goal is to improve arm movement quality and function	3
**221**	Chen et al., 2005	Canada	**Sample size:**<br>I= 41; C=42<br>**Mean Age:**<br>NR<br>**PSD:** NR<br>**TOS (ischemic/ hemorrhagic):**<br>I= 37/4; C= 36/6	Task-oriented interventions targeting walking or UE function	practice of functional UE tasks while sitting	Primary: Balance self-efficacy (16-item Activities-specific Balance Confidence (ABC) scale)	The walking intervention was associated with a significantly greater average proportional change in balance self-efficacy than the control.	Task-oriented walking retraining enhances balance self-efficacy in community-dwelling individuals with chronic stroke	3
**222**	Thielman, et al, 2004	NY & Australia	**Sample size:**<br>I=6<br>C= 6<br><br>**Age Range:** NR<br><br>**Mean Age:**<br>I = 72.83<br>C = 72.83<br><br>**PSD(months):**<br>I = 9.33	Task-related training (TRT) involving reaching to objects placed across the workspace.	Progressive Resistive Exercise (PRE) involving whole-arm pulling against resistive therapeuti	Forward reaching (A Panasonic video camcordera (60Hz)). Kinematic analysis of arm trajectory and trunk motion (the Peak Performance System); muscle tone (MAS); motor functions (Rivermead Motor Assessment (RMA))	For low-level groups, TRT resulted in increased substitutive trunk use at the target ipsilateral to the moving arm and for midline and contralateral targets after PRE. Only low-level, TRT subjects straightened hand paths, which suggested better coordination of elbow-shoulder motion, and improved on the RMA. High-level subjects decreased trunk use at ipsilateral target after PRE, which was not observed after TRT. No significant differences between training conditions	Training benefits appear to depend on initial level of functioning. Although compensatory trunk use was evident, low-level subjects seemed to benefit most from TRT. High-level subjects, whose kinematics showed	2

			C = 14.33<br><br>**TOS(ischemic/hemorrhagic):**<br>I = 5/1<br>C = 5/1		c tubing in planes and distances similar to that in TRT		were found for other kinematic variables. Analysis of RMA scores yielded significant effects for function level (*P*<.01); pretest and posttest (*P*<.013), group by pretest/posttest (*P*<.013), and the 3-way interaction of group by level by pretest/posttest (*P*<.03). Significant improvement was found only for low-level, TRT subjects. There were no statistically significant training effects for the MAS.	fairly normal movement organization, demonstrated less compensatory movement after PRE.	

TEMPA: Test d'Evaluation des Membres superieurs des Personnes Agees

UE: Upper Extremity, LE: Lower Extremity, STREAM :Stroke Rehabilitation Assessment of Movement

BI: Barthel Index

OARS-IADL: Older Americans Resources and Services Scale - Instrumental Activities of Daily Living

SF-36: The Medical Outcomes Study 36-Item Short form Questionnaire

GDS: Geriatric Depression Scale

BBT: Box and Blocks Test

FMA: Fugyl Meyer Assessment Scale

Muscle Strengthening Exercise

	Study (Authors, year	Country	Participants	Interventions/ Model of care	Control/ comparison	Outcomes assessed/ outcome measures	Results	Conclusion	Quality score
**223**	Eom et al, (2017)	Korea	42 stroke patients **Age (**year), E=69.2 ± 4.1 C=70.2 ± 3.6 **PSD**= less than three months **TOS**= both ischaemic and haemorrhagic [N/S]	Expiratory muscle strength training (EMST)	Sham EMST	-Videofluoroscopic dysphagia scale (VDS) was used to assess oropharyngeal swallowing -Penetration aspiration scale (PAS) reflects pharyngeal penetration and aspiration	The experimental group showed more improvement in pharyngeal phase of the VDS (p = 0.018 and 0.006, respectively) and PAS compared to the placebo group (p = 0.014)	EMST could improve the effects of dysphagia observed in post-stroke elderly patients based on swallowing function.	1
**224**	Rose et al, (2017)	USA	347 participants **Age** (Yrs): All=61.4±13.0 PSD ≤45 days TOS=both ischaemic and haemorrhagic [N/S]	E1:Task-specific Locomotor Training Programme (LTP):Early-LTP;Late-LTP E2: Home exercise program (HEP)	C: Usual care physical therapy	-Stroke severity(mRS) -National Institutes of Health Stroke Scale -Depression (Patient Health Questionnaire score ≥10) -FMA -Berg Balance Scale -ABC Scale [N/S]	There was a statistically significant difference in all training parameters between sessions 13 to 24 and sessions 25 to 36 (P < .05)	There was improvement in walking recovery after up to 24 sessions of locomotor training or strength and balance exercises at both 2 and 6 months after stroke.	4
74	Cha and Kim (2017)	Korea	30 subacute stroke **Age** (yrs): E1=55.80±16.40 E 2=53.80±13.28 C=53.70±9.67 **PSD** (Months)**:** E1= 4.20±1.22	E1: Ankle strengthening exercise group E2: Ankle strengthening exercise integrated group rTMS	C: rTMS group (control group .	Motor-evoked potential testing Peak torque at the ankle joint 10 m walk test	Subjects in group II showed significantly higher amplitude of MEP, plantarflexor and dorsiflexor of peak torque, 10 m walk test than groups I and CG (p < 0.05). Subjects in groups I and II differed significantly in the pre- and post-test for all variables, (p < 0.05). In the CG group, the pre- and post-test scores for	Strengthening exercise integrated rTMS has positive effects on motor function recovery in subacute stroke patients.	**4**

			E2=3.90±1.59<br>C= 4.10±1.19<br>**TOS**: [N/S]			[Uses were not stated]	the amplitude of MEP, dorsiflexor, and 10-walk test differed significantly ($p < 0.05$).		
225	Ivey et al, (2017)	USA	30 participants<br>**Age** (years)<br>E=57 ± 14<br>C=55 ± 9<br>**PSD** (years):<br>E=5 ± 4<br>C= 6 ± 5<br>**TOS**= both ischaemic and hemorrhagic [N/S]	Strength training (ST)	Standardized stretching	One repetition maximum<br><br>6-minute walk distance<br><br>Ankle foot orthosis;<br><br>Self-selected walking speed<br>*[uses not stated]*	ST participants (N = 14) had significantly greater SME gains compared with SC participants (N = 16) in both the paretic (178% versus 12%,<br>$P < .01$) and non-paretic legs (161% versus 12%, $P < .01$). These gains were accompanied by group differences for 6MWD ($P < .05$) and VO2 peak ($P < .05$)	ST regimen had a large impact on the capacity to sustain submaximal muscle contraction, a metric that may carry more practical significance for stroke than the often reported measures of maximum strength.	**4**
**226**	Guillén-Solà et al, (2017)	Spain	62 patients<br>Age (years):<br>C= 68.9±7.0<br>E1=67.9±10.6<br>E2=70.3±8.4<br>PSD (days):<br>C=9.3±5.1<br>E1=10.8±8.7<br>E2=11.0 (5.5)<br>TOS: ischaemic	E2: Sham inspiratory/expiratory muscle training IEMT+ neuromuscular electrical stimulation (NMES)<br>E1: SST+ IEMT	C: Standard swallow therapy (SST)	Penetration-Aspiration Scale<br><br>N/S<br><br>Maximal inspiratory and expiratory pressures<br><br>N/S	Maximal respiratory pressures were most improved in Group II: treatment effect was 12.9<br>(95% confidence interval 4.5-21.2) and 19.3 (95% confidence interval 8.5-30.3) for maximal inspiratory and expiratory pressures, respectively.	Adding IEMT to SST was an effective, feasible, and safe approach that improved respiratory muscle strength	3
**227**	Aidar et al, (2016)	Brazil	24 subjects<br>**Age** (year):<br>E=51.7 ± 8.0<br>C= 52.5 ± 7.7<br>PSD: N/A<br>TOS= ischaemic stroke	Strength training ST)	Normal daily activities (NDA)	Generic Questionnaire for the Assessment of Quality of Life"-SF 36 "	There was a significant increase in quality of life from pre-test to post-test (Δ% = 21.47%; $p = 0.021$) in EG. There were significant differences in all indicators of quality of life between groups at 12 weeks. There were greater gains in strength in EG than in CG ($p \leq 0.05$).	Improvement in the measures of strength in ST, and that there was a correlation between improvements in strength and quality of life in these patients who had previously suffered a.	4
**228**	Shin et.al , 2016	korea	P: Female poststroke patients;<br>Sample size=31 (E=16, C=15),<br>**Age** (Years):<br>E= 62.08 ±3.32,<br>C= 62.92 ±4.93;	PFMT + General Rehabilitation Exercise (GRE)	GRE	- MVSP (Perineometer),<br>- PFMA (intra-vaginal electromyography), - LUTS (Bristol Female Lower Urinary Tract	- MVSP for the PFMT and control groups were 18.35 ±5.24 and 8.46 ±3.50 mmHg, respectively.<br>-PFMA of the PFMT and control groups was 12.09 ±2.2) µVand 9.33 ±3.40 µV, respectively.<br>- After intervention, the changes of scores for inconvenience in the ADL	PFMT is beneficial for the management of urinary incontinence in female stroke patients.	3

			PSD (months):<br>E= 6.17 ±1.53,<br>C= 5.17 ±2.08;<br>TOS: NA			Symptom questionnaire)	of the PFMT and control groups were −15.00 ±6.25and −0.17 ±1.59, respectively. In addition, the changes of score for LUTS was improved more in the PFMT group -4.17 ±4.00 than in the control group -0.25 ±1.29 (P<0.05).		
**229**	Clark & Patten, 2013	USA	**Sample size:**<br>I=18; C=16<br>**Mean Age (yrs):**<br>**I=**63.2 ± 10.6<br>C= 59.7 ± 10.9<br>**PSD(months):**<br>I= 13.3 ± 4.9<br>C= 12.8 ± 4.7<br>**TOS: ischemic/ hemorrhagic :**<br>NR	Eccentric resistance training (ECC)	concentric resistance training (CON)	Neuromuscular activation (surface electromyography) and strength of the knee extensors (isokinetic dynamometer)	Both groups showed significant improvement in muscle strength (p <0.0001). This improvement was significantly greater in the eccentric group compared with the concentric (p <0.0001). Both groups also showed a significant effect of Time for leg activation (*P* < .0001). the ECC group experienced larger improvements in neuromuscular activation of paretic leg muscles, rectus femoris and vastus medialis (*P* < .005)	ECC resistance training was more effective for improving bilateral neuromuscular activation, strength, and walking speed following stroke.	2
**230**	Flansbjer, et al, 2012	Sweden	**Sample size:**<br>I=11 C=7<br><br>**Age Range:** NR<br><br>**Mean Age:** NR<br><br>**PSD** (Days):<br>I=60<br>C=60<br><br>**TOS: (**Ischemic/hemorrhagic) NR	Progressive resistance training of the knee extensors and flexors (PRE)	Usual daily activities. (UDA)	Leg Extension/Curl; Muscle strength (dynamometer); spasticity (mAS); gait speed (Fast Gait Speed [FGS] test); balance (TUG), wlking capacity (6MWT); and perceived participation (participation domain of Stroke Impact Scale [SIS]).	PRE had Sig ↑ in isotonic muscle strength (*p* < 0.001) and isokinetic muscle strength (*p* < 0.05); no ↓ in strength in UDA. The Δ in muscle tone were negligible in both groups. Both groups had Sig ↑ in TUG, FGS & 6MWT and were maintained at the 4-year follow-up. In UDA, the FGS was slower (*p*< 0.05) and the distance for the 6MWT shorter (*p* < 0.05). No sig Δ in perceived participation at the 4-year follow-up. Sig btwn-grp diff for the isotonic muscle strength (*p* < 0.01) and isokinetic knee extensor strength (*p*< 0.05) at the 4-year follow-up compared with baseline, but no other sig btwn-grp diffs.	There are long-term benefits of progressive resistance training in chronic stroke. However, apart from in muscle strength, these benefits are comparable with usual daily activities.	2
**231**	Cooke, et	Uni	**Sample size:**<br>G1:35, G2:36, C: 38	G1 = Conventional Physiotherapy	Conventional physiothe	Primary: Walking speed (10 Meter Walk Test),	At post intervention, G1 showed a significantly higher improvement in 10 meter walk test (*P* = .031) and	The result of this study shows that adequate (increased) conventional	3

	al, 2010	ted Kingdom	**Age (Yrs)**: G1=67.46 ±11.3 G2=71.17 ±10.6 C= 66.37±13.7 **PSD**: G1=32.43±21.29 G2=33.86±16.50 C=36.76 ±22.41 **TOS(ischemic/ hemorrhagic)**: NR	plus additional conventional physiotherapy (CPT + CPT)<br><br>G2 = conventional physiotherapy plus additional functional strength training (CPT + FST)	rapy [CPT]	community mobility (ability to walk at 0.8 m/s or more) , walking, torque around the paretic knee (isokinetic dynamometer), mobility functions (the modified Rivermead mobility index) temporal-spatial gait parameters (footsteps symmetry), the health status and health-related quality of life (EuroQuol)	community mobility (*P* = .038) compared to control; whereas, G2 showed no better improvement than control. No significant improvement was shown in G2 and control at post intervention and none in all the groups at follow-up. All groups showed improved but none significant peak torque about the paretic knee at outcome and follow-up, but this was most evident in the 2 experimental groups with G1 showing a statistically significant at outcome (*P* = .016). None of the groups showed a significant improvement in footstep symmetry, modified Rivermead index and EuroQuol.	therapy shows a greater improvement in walking speed and community mobility in stroke patients	
**232**	Donaldson et al., 2009	UK	**Sample size:** G1=10, G2=10, C=10 **Age (yrs):** G1:73.3, G2:72.6 C: 72.6 **PSD(days):** G1=21.7, G2=25.6 C= 13.4 **TOS:** NR	G1 = conventional physical therapy plus extra conventional physical therapy (CPT + CPT)<br><br>G2 = conventional physical therapy plus Functional strength training (CPT + FST)	Convention physical therapy CPT (no extra therapy),	primary outcome: Upper limb function (Action Research Arm Test (ARAT));<br><br>Secondary outcome: Dexterity (Nine Hole Peg Test (9HPT)); Upper limb strength: hand grip and pinch grip force (myometer)	CPT + FST group showed the largest increase in ARAT score and this was above the clinically important level of 5.7 points. For The Nine Hole Peg Test, the CPT + FST group shows higher significant improvement compared to control. For Hand grip force, the median change was found to be highest for the CPT group, and lowest for the CPT + CPT group. for Pinch grip force, the largest median change was found for the CPT+ FST group, and the smallest for the CPT + CPT group, but there was no significant difference between the two highest scoring groups. For Isometric elbow flexion force, the smallest change was found for the CPT group	The study found a trend for enhanced motor recovery for the CPT □□FST group for all measures except hand grip force. The improvements found achieved set clinical importance for ARAT, 9HPT, and isometric elbow flexion force.	1
**233**	Lee et al., 2008	Austr	**Sample size:** G1=12, G2=12, G3=12, C=12 **Mean Age:** G1 = 67.2	G1 = Real AEROBIC (cycling) and sham progressive	Sham aerobic cycling and sham progressi	Walking Ability (6-minute walk test); Cardiorespiratory Fitness and Associated Measures	Neither of the real AEROBIC nor STRENGTH improved walking ability significantly more than sham exercise, although STRENGTH significantly improved participants'	Single-modality exercises targeted at existing impairments do not optimally address the	3

		a l i a	+10.6<br>G2 = 62.9 + 9.3<br>G3= 60.5 + 10.6<br>C= 65.3 + 6<br>**PSD(months):**<br>G1 = 52.4 + 2.2<br>G2 = 44.2 + 63.9<br>G3= 63.2 + 40.5<br>C= 65.8 + 42.3<br>**TOS(ischemic/ hemorrhagic/others):**<br>I: G1 = 9/2/1<br>G2 = 8/2/2<br>G3= 7/4/1<br>C= 9/1/2	resistance training<br>G2 = real STRENGTH (real progressive resistance training) and Sham aerobic training<br>G3 = Real AEROBIC and STRENGTH (real cycling and real progressive resistance training).	ve resistanc e training	(progressive-intensity maximal-effort cycling test); Dynamic muscle strength (1RM) or maximal isometric Force (Dynamometer); health-related quality of life, psychological function, and general health (36-item Short Form Medical Outcomes Survey [SF-36]);	stair climbing power by 17% (P=.009), as well as their muscle strength, muscle force, and endurance; cycling peak power output; and self-efficacy. Conversely, AEROBIC improved indicators of cardiorespiratory fitness only. Cycling plus progressive resistance training produced larger effects than either single modality for mobility and impairment outcomes.	functional deficits of walking. A combined AEROBIC and STRENGHT training shows promising trend.	
**234**	Flansbjer et al, 2008	UK, Sweden & Lund	**Sample size:** I= 15 C= 9<br><br>**Age Range:** NR<br><br>**Mean Age:**<br>I= 61 + 5<br>C= 60 + 5<br><br>**PSD(months):**<br>I= 18.9 + 7.9<br>C= 20.0 + 11.6<br><br>**TOS(ischemic/ hemorrhagic):**<br>1 = 12/3<br>C = 6/3	Supervised progressive resistance training of the knee muscles (TG)	Continued their usual daily activities (CG)	Knee extension and knee flexion dynamic strength (Knee extension and knee flexion dynamic strength); gait performance (Timed “Up & Go” (TUG), Fast Gait Speed (FGS), and 6-Minute Walk Tests [6MWT]); Perceived participation (Stroke Impact Scale 3.0 (SIS))	TG: Dynamic strength sig ↑ (*p*<0.001) for both the paretic and non-paretic LL, but in CG, increase was only in the non-paretic LL ($p < 0.05$) after intervention and at fllow-up. Sig diff in Dynamic strenghht after the intervention ($p < 0.001$) and at follow-up ($p < 0.001$). Muscle tone was sig ↓ after the intervention in the TG ($p < 0.01$) and CG ($p = 0.02$). At follow-up, no sigΔ from baseline. For the TG, all gait performance tests improved sig ($p < 0.05$) after the intervention and for TUG and 6MWT at follow-up. For the CG, only TUG improved sig ($p < 0.05$) after the intervention. No sig btwn-grp diffs after the intervention; but at follow-up for TUG ($p < 0.05$). No sig btwn-grp diff for perceived participation after the intervention but at follow-up ($p < 0.05$). SIS at follow-up as 1% higher in TG, 19% lower than at	Progressive resistance training is an effective intervention to improve muscle strength in chronic stroke and there appear to be long-term benefits	2

							baseline.		

ABC: Activities-Specific Balance Confidence
TUG: Timed Up and Go test
6MWT: Six Minutes Walk Test
FMA: Fugyl Meyer Assessment Scale
mAS: modified Ashworth Scale

Muscle Stretching Exercises.

	**Study (Authors, year**	**Country**	**Participants**	**Interventions/ Model of care**	**Control/ comparison**	**Outcomes assessed/ outcome measures**	**Results**	**Conclusion**	**Quality score**
**235**	Ghasemi et al, (2018)	Iran	45 patients<br>**Age** (Yrs):<br>E=54.37±12.38<br>C=58.13±12.91<br>**PSD** (month)<br>E=24.60±19.76<br>C=18.07±13.53<br>TOS:I&H [N/A]	Functional stretching program	Routine physical therapy program	Ultrasonography and electrophysiology test to evaluate pennation angle; muscle thickness; and fascicle length. Dorsiflexion passive range of movement (PROM)	Pennation angle (P=0.006), and in muscle thickness (P=0.030) was sig.<br>H-reflex latency (P=0.006), pennation angle (P < .001), and muscle thickness (P=0.001) were altered after stretching training program and these changes were at sig level after 2-month follow-up<br>Nevertheless, no sig btwn-grp diff (F = 1.18, P = .28)	The use of functional stretching exercises can cause significant differences in neural and mechanical properties of spastic medial gastrocnemius<br>muscle in patients with chronic stroke	3
**236**	Sahin et al, 2012	Turkey	**Sample size:**<br>I = 21, C = 21<br>**Age** (Years):<br>I = 60.2 ± 6.2<br>C= 59.3 ± 9.3<br>**PSD** (months):<br>I= 25.0 ± 14.6<br>C= 35.1 ± 24.4<br>**TOS**: NR	Stretching with PNF after hot treatment with infrared and neuromuscular electrical stimulation (NMES) to the wrist extensors	Stretching and PNF alone after hot treatment with infrared. No NMES	Spasticity (mAS); ROM (goniometer); *Fmax/Mmax ratio, Hmax/Mmax amplitude ratio* (electrodes); Motor evaluation (Brunnstrom method); Functional	Both groups revealed a significant recovery after the treatment based on the MAS, the electrophysiological evaluation results, wrist ROM, FIM and Brunnstrom motor staging. The group receiving the combined treatment showed a better recovery in terms of MAS, wrist ROM, FIM and Brunnstrom motor staging compared to	The results of this study showed that NMES given together with stretching of the wrist extensor muscles was more effective than stretching of the wrist extensor muscles alone	3

						status (FIM)	the group doing the stretching alone.		
**2 3 7**	Gustafsson & McKenna, 2006	Australia	**Sample size:** I=15; C= 17 **Mean Age Yrs:** I= 67.1+13.9 C= 65.9+15.6 **PSD(days):** I=19.7+9.6 C= 16.5+12.3 **TOS(ischemic/ hemorrhagic):** 1=11/4, C=12/5	Conventional rehabilitation with static positional stretches of the stroke-affected shoulder	Conventional rehabilitation with static positional stretches of the stroke-affected shoulder	Pain-free passive ROM (a universal goniometer); hemiplegic shoulder pain (RAI & VAS)); motor recovery (MAS) and functional independence (BI)	Both groups showed significant reduction in mean pain-free passive range of motion into external rotation (P = 0.005) but no significant group differences was shown. No significant change were found in pain for all participants (p =0.47) or between groups (P = 0.78). All participants improved significantly with respect to motor recovery (P <0.01) and functional independence (P < 0.01). There were no significant group differences	Participation in static positional stretches of the stroke-affected shoulder did not result in higher improved outcomes.	3

ROM: Range of Motion

FIM: Functional Independent Measurement

mAS: modified Ashworth Scale

RAI: Ritchie Articular Index

VAS: Visual Analogue Scale

MAS: Motor Assessment Scale

BI: Barthel Index

Cognitive Therapy

	Study (Authors, year	Country	Participants	Interventions/ Model of care	Control/ comparison	Outcomes assessed/ outcome measures	Results	Conclusion	Quality score
**238**	Cheng et al, 2018	China.	168 post stroke patients. **Age** (Yrs): E= 63.36 ± 9.08 C= 63.11 ±11.95 PSD= within 7 days of stroke. TOS= both ischaemic and haemorrhagic combined [N/S]	Comprehensive Rehabilitation Training (CRT)	Conventional Treatment (CT)	-Cognitive Status (Montreal cognitive assessment score [MCAS]; Mini mental state examination score [MMSES]) –Depression/Anxiety (Hospital anxiety and depression [HAD] scale) -Anxiety (Self-rating anxiety scale [SAS])	Both MCAS change (Month12 [M12]-baseline; P=0.001) and MMSES change (M12-baseline) were higher in CRT than CT (P = .004), and the percentage of cognitive impairment by MCAS was lower (P = .003) in CRT then CT at M12. The HADS anxiety score change (M12-baseline; P = .002) and the SAS score change (M12-baseline; P = .006) HADS depression score change (M12-baseline; P<0.001) and the SDS score change (M12-baseline; P=0.002) were reduced in CRT compared to CT	CRT contributes to the recovery of cognitiveimpairment , and decreasesanxiety and depression in poststrokepatients.	2
**239**	Fotakopoulos and Kotlia, 2018	Greece.	65 stroke survivor **Age** (yrs) E=73.29±4 C=76.02±3 **TOS**(ischaemic/ haemorrhagic) E=23/18; C=14/10 **PSD** = NR	daily listening to experiential/traditional music (ETM)	standard care only (SC)	BI, Barthel Index; CBF, cerebral blood flow, mMT, mini Mental test, [N/S]	Overall, cognitive and motor recovery was 40% (26 of 65 patients). Recovery was significantly higher in ETM (26.2%) compared to SC (13.8%) (P =0.001) Multivariate analysis revealed that only Group and Lesion size were independent predictors for Recovery (odd ratio [OR] [95% CI) 0.11(.001-.133) and .798(.668-.954) respectively	ETM has a positive effect on mood profi le in stroke patients and Recovery rate is higher when exercise rehabilitation program was accompanied by an enriched sound environment with experiential music.	2
**240**	Tang et al, (2016)	Canada	47 participants Age, years: median (IQR) E=66 (62–71) C= 64 (62–75) PSD years, median (IQR)	Aerobic exercise	Balance and flxibility	Verbal Digit Span was used for executive function performance Trail Making Test B was used to assess Set shifting	There was an improvement in verbal item memory in both groups (time effect p = 0.04), and no between-group differences in improvement in the other outcomes (p > 0.27).	6 months of high or low intensity exercise was not effective in improving cognitive function, specifically executive functions.	3

			E= 3.5 (2.2–6.7)<br>C= 2.3 (1.8–5.1)<br>TOS:Lacunar/Ischemic/Hemorrhagic/Unknown<br>E= 3 /7 /9/6<br>C= 4 /12 /7 /2			Color–Word<br>Stroop Test was used for selective attention and conflict resolution			
**241**	Wentink et al(2016)	Netherlands	110 participants<br>**Age** range (years):<br>E=(46–74)<br>C=(46–73)<br>PSD (months):<br>E= 26 ± 9.1<br>C= 25 ± 7.4<br>TOS,(infarction/haemorrhagic/unknown)<br>E=29/21/3;<br>C=44/13/0	8-week brain training programme (Lumosity Inc.®)	General information about the brain weekly.	-Trail Making Test [TMT] was used to assess attention and flexibility<br>-Block Span Task and Digit Span Task were used to assess working memory with two subtests: blocks/digits forward (sequential order) and blocks/digits backward (reversed order)<br>-Eriksen Flanker Task were used to assess Speed and flexibility<br>-Raven Standard Progressive Matrices[SPM] for Fluid intelligence<br>-Cognitive Failures Questionnaire was used to measure self-perceived cognitive failure.<br>-Stroke Specific Qualit y of Life Scale (SS-QoL -12) was used to measure health-related QoL (HRQoL)<br>-General Self-Effi cacy Scale (GSES) was used to assess participants' belief in the ability to respond to and cope with novel or difficult situations and unexpected setbacks or obstacles.	TMT A Spearman , r= .180 (p = .07), and TMT B, r = .308 (p < .01), Block Span forward, r= −.181 (p = .06), and backward, r = −.079 (p = .42), Digit Span forward, r =−.184 (p = .06), and backward, r = −.295 (p < .01), Flanker incongruent, r = .168 (p= .09), and congruent, r= .158 (p = .11), and the Raven SPM, r = −.158 (p = .15).	No effect of the training was found on cognitive functioning, QoL or self-efficacy when compared to the control condition, except for very limited effects on working memory and speed.	4
**242**	Lund, et al, 2011	Norwa	**Sample size:** I : 39 C = 47<br>**Age Range:**<br>I=NR	A lifestyle course in combination with	Physical activity alone	Perceived health and well-being (The Medical Outcomes Study 36-item Short Form questionnaire (SF-36)); change in self-reported occupational performance and	At 9M follow-up, the SF-36 subscales – mental health, vitality, social functioning, physical functioning, role physical and role emotional showed improvements both	Improvements were seen in both groups, but no statistically significant differences were	3

		y	C=NR<br><br>**Mean Age** (Years):<br>I = 75 + 7.2<br>C = 79 + 6.5<br><br>**PSD** (days):<br>I= 161<br>C= 137<br><br>**TOS**: I = NR<br>C = NR	physical activity		Satisfaction(Canadian Occupational Performance Measure [COPM]); depression (The Hospital Anxiety and anxiety and Depression Scale [HADS]); mobility (the Timed Up and Go test [TUG]) and cognitive function (Trail Making Test [TMT] A and B)	groups. No significant differences between the groups at 9M follow-up in any of the SF-36 subscales. Both groups significantly iproved in the HADS and depression scale but no significant difference was seen between the groups. Significant improvements in both groups were observed in COPM but no difference between the groups. TUG showed no significant changes in both groups and between them. TMT A showed significant improvements after 9M in the control group. However, TMT A and B did not demonstrate any significant differences between the groups.	found in the intervention group compared to controls. An intervention comprising regular group-based activity with peers may be sufficient in the long-term rehabilitation after stroke.	
**243**	Ryan et al, (2011)	Canada	**Sample size:** I= 22 C= 22<br><br>**Age Range:** NR<br><br>**Mean Age:**<br>I= 62.8+12.8<br>C= 60.7+10.3<br><br>**PSD:** NR<br><br>**TOS(ischemic/hemorrhagic):** I = 21/1 C = 18/4	Standard rehabilitation with continuous Positive airway pressure (CPAP)	standard rehabilitation alone (SR)	Primary: Stroke severity (Canadian Neurological scale); motor function (6-minute walk test [6MWT]); neurocognitive function (sustained attention to response test: a measure of vigilance and the digit or spatial span-backward: a measure of executive function).<br>Secondary: ADL (Functional Independence measure (FIM)), Chedoke-McMaster Stroke assessment of upper and lower limb motor recovery test [CMSA], hand-grip strength, Berg Balance scale, Epworth Sleepiness scale (ESS), Stanford Sleepiness scale (SSS), the Purdue<br>Pegboard test and the Beck depression inventory-1 (BDI).	CPAP experienced higher improvement in stroke severity (P<0.001) but not in 6MWT, sustained attention response test, or digit or spatial span-backward. SR showed no significant improvement in 6MWT. CPAP did not experience a significant improvement in sustained attention/vigilance on the sustained attention to response test but did show a significant improvement in digit span and visual spatial span-backward, not seen in the control group, CPAP experienced improvements in the ESS (P<0.001), motor component of FIM (P=0.05), CMSA of the leg (P=0.001), and the affective component of depression<br>(P=0.006), but not neurocognitive function. There were significant reductions in the ESS and SSS in CPAP compared to SR. There were also significant improvements in the FIM and BBS within, but not between both groups	Treatment with CPAP in stroke patients undergoing rehabilitation improved functional, motor and neurocognitive outcomes	3
**244**	Johnston et al,	UK	**Sample size:** I= 103 C= 100	a workbook-based	Normal care (NC)	Primary: Recovery from disability (Observer Assessed Disability (OAD))	WBI had significantly better OAD than NC (p=0.019); BI over three interviews showed no group by time interaction. There were no	Thee study showed that a workbook-based intervention,	2

	2007		**Age Range:** I= NR C= NR<br><br>**Mean Age:** I= 68.96+12.64 C= 68.79+12.02<br><br>**PSD:** NR<br><br>**TOS:** NR	intervention, designed to change cognitions about control (WBI)		Secondary: Disability/activity limitations (Barthel Index [BI]); Emotional distress (Hospital Anxiety and Depression Scale (HADS)); Satisfaction with treatment and advice (scales from 0 (totally unsatisfied) to 10 (totally satisfied)); Perceived control over recovery (Recovery Locus of Control Scale (RLOC)); Confidence in recovery (scale from 0 (not at all confident) to 10 (totally confident)	significant effects of group by time interactions on the total HADS for patient, however, there was a significant time effect, (p<0.001).. No significant effects of group or group by time interactions for Satisfaction with Care among patients. There was no significant group by time interaction between patients in the control and intervention groups for RLOC. There was a significant group by time interaction effect for patients' Confidence in Recovery (p=0.001).	designed to change cognitions about control improved recovery from disability better than normal care in stroke patients at 6 months after discharge from hospital	

LUTS = Lower Urinary Tract Symptoms

PFMT = Pelvic Floor Muscle Training

PSD - poststroke duration; TOS. Quality score: assessed with Jadad quality assessment scale for rating randomized controlled trials; N/A = Not Available or Applicable PFMT = Pelvic Floor Muscle Training; LUTS = Lower Urinary Tract Symptoms; E = Experimental; C = Comparison/Control; I = Ischaemic Stroke; H = Haemorrhagic Stroke; ES = Electrical Stimulation; VDS = Videofluoroscopy Dysphagia Scale; PAS = Penetration–Aspiration Scale; CT = Conventional Treatment; NFT = Neuromuscular Faccilitation; BMD = Bone Mineral Density, ALP = Alkaline phosphatase; BGP = Bone gla protein; IL-6 = Interleukin-6; PNS = Peripheral Nerve Stimulation; mCIT = modified Constraint Induced Movement Therapy; WMFT = Wolf Motor Function Test; FMA = Fugl-Meyer Assessment Scale; ARAT = Action Reach ArmTest; UE = Upper Extremity; mAS = Modified Ashworth Scale; MBEST = Mini Balance Evaluation Systems Test, TUG = Time up and Go; 6MWT = Six minutes walk test;ABC =Activity Specific Balance; FAI = Frenchai Activity Index; CHIEF = Craig Hospital Inventory of Environmental Factors; SIS- = Stroke Impact Scale ; mCIMT = Modified Contraint Induced Movement Therapy; rTMS = Repetitive transcranial magnetic stimulation; BI = Barthel Index; HADS-Thai = Thai version of the Hospital Anxiety and Depression Scale; DEXA = Dual energy-ray Absorptometry; LWBV = Low intensity Whole Body Vibration; HWBV = High intensity Whole Body Vibration; MFT = Manual Function Test; VD = Vertical Displacement (Shoulder Subluxation); UL = Upper Limb; MAL = Motor Activity Log; VO2 peak, 6MWD = 6-minute walk distance, 30WT = 30-ft Walk Times; 48SC = 48-hr step counts; TDx = Treadmill; SAM = Step Activity Monitoring; BCI-FES = Brain–Computer Interface controlled Functional Electrical Stimulation; MAL = Motor Activity Log; mBI = modified Berthel Index; AOT = Action Observational Training; SMA = Stride Management Assist; FTST = Functional Task Specific Training; m = Median;

Speech Therapy

	Study (Authors, year	Country	Participants	Interventions/ Model of care	Control/ comparison	Outcomes assessed/ outcome measures	Results	Conclusion	Quality score
**245**	Raglio et.al, 2016	Italy	Sample size=20 (E=10, C=10),<br>Age (years): E:61.3(42-89), C:70.9(63–89)<br>PSD (Yrs): E=3.4±4.1 C=3.8±3.3<br>TOS: NA	Music Therapy (MT) + Speech Language Therapy (SLT)	SLT	- Disability (mRS),<br>- Speech/language assessment (Milan protocol, & AAT),<br>- QoL (SF-36 short form),<br>- Personality (BFO)<br>- Psychological aspects (BDI),<br>- Speech language [Token test (comprehension), BNT, AAT]	- significant improvement in spontaneous speech in MT+SLT group (Aachener Aphasie subtest: p = 0.020; Cohen's d = 0.35);<br><br>- the 50% of the MT+SLT group showed also an improvement in vitality component of SF-36 (X2 = 4.114; p = 0.043)	The combined use of MT and SLT can lead to a better result in the rehabilitation of patients with aphasia than SLT alone.	5
**85**	Waldowski et al, 2013	Poland	**Sample size:** I=8 , C=10<br>**Mean Age:** **I=**63 ±12.07 C=58.7±11.38<br>**PSD(years):** I= 13.3 ±4.06 C= 11.1 ±2.08<br>**TOS: ischemic/hemorrhagic :** NR	Speech and language therapy (SLT) + real low-frequency repetitive transcranial magnetic stimulation (rTMS)	SLT + sham rTMS	Naming assessment: accuracy of naming and reaction time (RT) (Computerized Picture Naming Test (CPNT)); language abilities (Boston Diagnostic Aphasia Test (BDAE)); aphasia severity (Aphasia Severity Rating Scale (ASRS))	Both groups showed significant improvement in their naming abilities, however, there was no significant difference in average test scores between groups at any time. SLT+rTMS showed better scores in average RT immediately after rTMS treatment (p = 0.048). no other significant between group difference were seen in all the other measured outcomes.	SLT + rTMS do not appear to be more effective in improving naming in early-stroke aphasia patients than SLT + sham rTMS	5
**246**	Bowen et al., 201	UK	**Sample size:** I=85 C=85<br><br>**Age Range:**	Enhanced, agreed best practice, communication	A similar frequency on amount	Primary outcome: functional communicative ability (Therapy Outcome Measure (TOM) activity subscale). Secondary outcomes: participants' perceptions on the	Both groups showed statistically significant improvement in TOM and no significant iprovemnet in any of the secondary otucomess.	There was no evidence of added benefit of the SLT over and above SCV on	3

	2		I= 40–92<br>C = 32–97<br><br>**Mean Age**:<br>I= 70<br>C=70<br><br>**PSD** (Days):<br>NR<br><br>**TOS:** NR	therapy specific to aphasia or dysarthria, offered by speech and language therapists according to participants' needs (SLT)	of social contact by employed visitors (not therapists). (SCV)	outcomes (Communication Outcomes After Stroke<br>scale (COAST)); carers' perceptions of participants from part of the Carer COAST, carers' wellbeing on (Carers of Older People in Europe Index and quality of life items from Carer COAST).	No significant difference was seen in all the outcomes between the groups.	participant or carer perceptions of the participant's communication, nor on carer perceptions of impact on themselves in terms of their own quality of life or wellbeing.	
**247**	Lincoln et al., 1984	Nottingham	**Sample size:**<br>I= 104 C= 87<br>**Mean Age:**<br>NR<br>**PSD:** NR<br>**TOS(ischemic/hemorrhagic):**NR	Speech Therapy	No treatment	-Quantifying language and measuring slight changes in language behaviour. (Porch index of communicative ability (PICA)); -Functional language (Functional communication profile (FCP));<br>-Classification of aphasia (Boston Diagnostic Aphasia Examination (BDAE));<br>-Relatives observations of the patient's expressive speech and understanding (speech questionnaire)	There were no significant differences between the groups at either 10th, 22nd, or 34th weeks on the PICA and FCP. There was no significant difference between the groups in aphasia types at 10 weeks post stroke (p>0.05).	Speech therapy did not improve language abilities more than was achieved by spontaneous recovery.	3

Aerobic Exercise/Physical Activity Training

Ref	Study (Author s, year	Coun try	Participants	Interventions/ Model of care	Control/ comparison	Outcomes assessed/ outcome measures	Results	Conclusion	Quality score
**239**	Fotakopoulos & Kotlia, 2018	Greece.	65strokesurvivor<br>Age (Yrs)<br>E=73.29±4<br>Ce=76.02±3<br>TOS(ischaemic/haemorrhagic)<br>E=23/18;<br>C=14/10<br>PSD = NR	Exercise rehab with daily listening to experiential/traditional music),	Standard exercise care	BI, Barthel Index;<br><br>CBF, cerebral blood flow,<br><br>mMT, mini Mental test,<br><br>[N/S]	-Overall, cognitive and motor recovery was 40% (26 of 65 patients). Recovery was significantly higher in Group MG (26.2%) compared to CG Group (13.8%)<br>(P = .001<br><br>Multivariate analysis revealed thatonly Group and Lesion size were independent predictors for Recovery (odd ratio [OR][95%confidence interval]) .11(.001-.133) and .798(.668-.954) respectively	Music-based exercise program has a positive effect on mood profi le in stroke patients and Recovery rate is higher when exercise rehabilitation program was accompanied by an enriched sound environment with experiential music.	2
**248**	Kim & Kang (2018)	Korea	27 patients<br>(C=13; E=1 4).<br>**AGE** (Yrs)<br>C=51.54±2.88<br>E=51.43±2.62<br>**PSD**=1-2years<br>**TOS** (ischaemic/haemorrhagic)<br>E=11/3; C=11/2	Proprioceptive neuromuscular facilitation treadmill training (TT) using PNF lower-leg t aping (PNFLT) (PNFLT-TT)	Treadmill training (TT)	-6MWT (6-min walk test)<br>1-0MWT (10m walking test)<br>TUG(timed up and go test)<br><br>*[uses were not supplied]*	After intervention, the PNFLT-TT group showed significantly more effective changes than the TT group in 6MWT, 10MWT, and TUG ($P < 0.05$ and < 0.01).	The intervention of PNFLT-TT was more effective in improving walking and balance ability in patients with stroke than treadmill training.	1
**249**	Eyvaz et al, 2018	Turkey	60 patients<br>Age (years):<br>E=**58.5 ± 6.27**<br>**C=58.3 ± 5.43**	water based exercise (WBE) program	land-based exercises (LBE) alone	FIM (self care, sphincter control,mobility,locomotion,communication and social cognition)	The improvement in the vitality parameter of SF-36 was higher in the study group ($p < 0.05$), and improvement	Applying WBE together with the LBE (except SF-36 vitality sub-	1

			PSD**(months)**<br>**E= 23.4 ± 15.1**<br>**C=24.2 ± 15.1**<br>TOS= ischaemic/ haemorrhagic<br>**E=27/3; C=23/7**	applied in combination with the land-based exercises (LBE)		-BBS (Balance/RoF)<br>- TUG (dynamic stability/ambulation)<br>- SF -36 (physical and mental health)	in the BBS was significantly higher in the LBE group than the WBE group ($p < 0.05$)<br>For the Functional Independence Measure= 107 ± 6.3 110 ± 6.2 0.003 99.9 ± 10.4 103.8 ± 10.7 0.002	parameter) in patients with hemiplegia did not make any additional contribution to the application of LBE alone.	
**250**	Ekechukwu et al, 2017	Nigeria	69 patients<br>Age (years): 59.33±8.80<br>PSD**: N/A**<br>TOS= ischaemic/ haemorrhagic<br>**52/17**	E1: Continuous Aerobic Training (CT),<br>E2: Interval Aerobic Training (IT)	E3: Combined Aerobic Training (CmbT)	Haematological Indices (WBC, RBC, Hg, PC, MPV)	Sig within-group improvement in all the variables ($p<0.05$) except WBC in CT. IT was the most effective in decreasing WBC and MPV and also the most effective in increasing Hg and PC.	IT mode is the most effective aerobic training mode for improving haematologica indices post stroke	3
**251**	Rozental-Iluz et al, 2016	Israel	39 patients (E:20, C:19)<br>Age (years): N/A<br>PSD**: N/A**<br>TOS= N/A	E= interactive video-game group intervention (VG)	C=Taditional Group Interaction (TG)	Executive function: TMT<br>Performance: the Bill Paying Task (BPT)	BPTBill ↓ed by 27.5% and 36.6% for VG & TG respectively (F=17.3, P<0.000) and continued to ↓ in VG with small effect sizes. Effect size was small to medium for the TMT without any sig diff	VG provide combined cognitive-motor stimulation and therefore have potential to improve executive functioning of stroke patients	3
**252**	van de Ven et al, 2017	Netherlands	97 stroke patients (E:38, C1:35, C2:24)<br>Age (years): E: 55.0 ±9.1, C1: 62.0±7.5, C2: 60.5 ±9.0<br>PSD (Months): E=28.3, C1:28.3, C2:29.1<br>TOS= N/A	E: computer-based cognitive Training(n = 38),	C1:mock training; n = 35), or a<br><br>C2: waiting list control group (n = 24)	Cognition: Cognitive Failure Questionnaire (CFQ),<br>-Executive function: Dysexecutive Functioning Questionnaire (DFQ)<br>-QoL: SF-36, ADL: IADL scale,<br>-Participation Utrecht Scale	All groups: ↑ in CFQ & DFQ onlyHowever, the intervention group did not improve more than the two control groups ($p>0.05$).	computer-based cognitive flexibility training did not improve subjective cognitive functioning or quality of life after stroke	3
**253**	Stahl et al, 2016	Germany	18 chronic aphasic stroke survivors<br>Age (Yrs): E= 47.8±10.2; C=	E: Intensive Language-Action Therapy (ILAT) -	C: NT-ILAT	Language Performance (standardized aphasia test battery)	Sig ↑ in language performance with ILAT, independent of when this method was administeredbut NT tended to benefit language	Language for communication and social interaction increases the	3

			54.4±12.0	Naming Therapy (NT)			performance only when given at the onset of the treatment.	efficacy of intensive aphasia therapy.	
**254**	Crotty et al (2018)	Australia	24 patients **Mean age** 65±4.3 yrs<br>**PSD**<br>51±52.3 days,<br>**TOS**= both ischaemic and haemorrhagic combined<br>[N/S]	Combined standardized scanning and mobility (S&M)	Individualized therapy (IT)	National Eye Institute Visual functioning Questionnaire (NEI VFQ25) for the assessment of scanning ability.	3 months significant differences were found for the QOL- NEI VFQ25 total score (P = 0.03) and dependency sub-score (P = 0.03) measures. No significant differences were found between intervention groups for the primary outcome measure of scanning ability whilst walking at 7 weeks and at 3 months (P > 0.05	S&M improved QOL. Allocation of resources to visual rehabilitation services point towards the implementation of more mobility practice over a longer period of time.	1
**255**	De Luca,et al. (2018)	Italy	32 patients<br>**Age** (Yrs):<br>E= 52.7 ± 15.2<br>C= 50.5 ± 14.3<br>**PSD**= at least after 6 months<br>**TOS**= both ischaemic and haemorrhagic<br>[N/A]	computerized rehabilitation training using software Power-Afa?	Conventional speech therapy.	-Attentive matrices test to evaluate the visual selective attention<br>-Aphasia Rating Scale For Depression was used to better evaluate patient's mood and behavior .<br>-Constructional apraxia and ideomotor apraxia test for the evaluation of praxis abilities<br>-Functional Independence Measure was administered to caregivers to measure patient's autonomy in everyday life	The experimental group had a significant improvement from T0 to T1 in all the outcomes, whereas for the control group patients such an improvement was significant only concerning Functional Independence Measure and ideomotor praxis. There was statistically significant difference between the two groups	The software Power-Afa can be considered a valuable tool in improving the linguistic and cognitive recovery in patients affected by post stroke aphasia in the chronic phase	1
**51**	Klomjai, et al. (2018)	Thailand	19 stroke patients<br>**Age range** of all patients: 20–74 years<br>**PSD**= less than 6 months<br>**TOS**= ischaemic	Transcranial direct current stimulation (tDCS)	Conventional physical therapy (CPT)	-Lower-limb performance [Timed Up and Go (TUG) and Five-Times-Sit-To-Stand (FTSTS)] tests and<br>-Muscle strength (peak knee torque of extension).	Significant within-group difference in TUG (p = 0.004) for only tDCS. For FTSTS, paired t-test revealed a significant within-group difference in FTSTSfor only tDCS (p = 0.003). No	A single session of dual-tDCS before PT in people with sub-acute stroke immediately improved lower-	2

			stroke				significant difference between the two groups.	limb function but not strength.	
**256**	Ten et al. (2017)	Nethe rlands	34 patients<br>**Age**, yrs m(IQR)<br>E=59.31 (14.45)<br>C= 61.48 (13.37)<br>**PSD** days m(IQR)<br>E=41.50 (39.00)<br>C= 37.00 (37.00)<br>TOS(%) :I/ICH/SAH<br>E=68/29/4<br>C=76/17/7	Prism adaptation (PA)	Sham adaptation (SA)	Change of Neglect [Catherine Bergego Scale (CBS)]<br>Change in Performance [Mobility Assessment Course (MAC)]<br>Static paper-and-pencil task [Shape cancellation task (SC)] used to assess	Significant time-dependent improvements in CBS, MAC, and SC (all F > 15.57; P < .001).<br>No significant difference in magnitude of improvement between groups on the CBS, MAC, and SC (all F < 2.54; P > .113]	No beneficial effects of PA over SA in the subacute phase poststroke were observed	4
**257**	Kerr et al, (2017)	Glasg ow, Uk	61 patients<br>**Age** (Yrs):<br>E= 66.89±13.40<br>C=71.50±12.58<br>**PSD** (days):<br>E= 8.74±7.53<br>C= 9.33±11.47<br>TOS(ischaemic /haemorrhagic)<br>E=32/8; C=19/3	Wearing of physical activity monitor (PAM)	No PAM	-Modifid Rivermead Mobility Index (mRMI)<br>-Five times sit to stand test (FTSTS).<br>*[uses were not stated]*	There was an overall improvement in mobility (p = 0.002) but not FTSTS (p = 0.053) neither outcome was affected by group allocation (p=0.158).	Compared to the control, PAM did not appear to influ-ence FTSTS outcomes.	2
258	Hammer beck et al, (2017)	UK	36 stroke patients<br>**Mean Age** (yrs)<br>57.5 ± 11.5<br>PSD: N/A<br>TOS=N/S	Fast training	Slow training	-Spatial accuracy at movement end.<br>-Fugl-Meyer scores (FMA)<br>[N/S]	FMA scores were significant for only FT (p=0.004). Neither of these changes are clinically meaningful (↓hypertonus mAS=0.21±0.85, & ↑FMA=1.84±2.27)	Performance improvement can be achieved without the use of compensatory strategies	**2**
259	Jung et al.(2017)	Repu blic of Korea	40 stroke patients<br>**Age** range (Yrs):<br>E=56.2 ± 10.4<br>C=56.3± 10.2<br>**PSD**:<br>E=6.5 ± 2.7<br>C=6.6 ± 2.5<br>**TOS** (ischaemic/ haemorrhagic)<br>E=8/12; C=9/11	Transcutaneou s Electrical-Stimulation (TENS)	Sham stimulation	-Composite Spasticity-Score was used to evaluate the spasticity of the ankle plantar flexor<br>-Isometric strength in the extensor of hip, knee and ankle	Spasticity in the TENS group improved significantly > sham group (0.7± 0.8, p < 0.05). The muscle strength of hip extensor in the TENS group was significantly > sham group (1.0 ± 0.8 kg, p < 0.05). Significant improvement in postural-sway was observed in the TENS group compared to the sham group (p < 0.05).	Sit-to-stand training combined with TENS may be used to improve the spasticity, balance function and muscle strength in stroke patients.	**4**

**260**	Ballester etal, (2016)	Spain	18 patients<br>**Age** range,(years)<br>E=[57.80–68.50]<br>C=[50.80–63.30]<br>**PSD**, days<br>E=269.25–373.00<br>C=493.50–826.00<br>**TOS**:ischaemic/ haemorrhagic<br>E= 8/1; C= 6/3	VR based traing + Reinforcement -Induced Movement Therapy (RIMT)	VR based traing only	-Motor Function UE-FMA<br>- Bi-manual motor function (CAHAI-7)<br>- Functional independence (BI)<br>- Mood (Hamilton scale)	The experimental group continued to exhibit further gains in UE-FM at 12-weeks follow-up (p < .05)	RIMT seems beneficial for inducing significant improvement in chronic stroke patients	4
**261**	Pomeroy et.al, 2016	Norwich	P: Individuals with poststroke<br>Sample size=105 (E=51, C=54),<br>**Age** (Years)=<br>E=64.2±15.3,<br>C= 69.1±13.6;<br>**PSD** (Days):<br>E= 19.5±11.3 ,<br>C= 22.2±26.7;<br>**TOS**(ischemic/hemorrhagic):<br>E = 41/8; C =45/8	SWIFT cast + Conventional Physical Therapy (CPT)	CPT	- Walking speed (3MWT)<br>- Ability to Walk (FAC),<br>- Motor Activity (mRMI);<br>-Gait Assesment (Peak angular velocity of the knee,- Gait symmetry, and angle of the tibia)	- SWIFT Cast utilization during CPT sessions was significantly higher (P< .001) for the SWIFT Cast (55%) than the CPT group (3%).<br>-The CPT group used an AFO in 26% of CPT sessions, compared with 11% for the SWIFT Cast group (P= .005).<br>-Differences in walking speed, after accounting for minimization factors, were insignificant at outcome (P= .345) and follow-up (P= .360).	-SWIFT Cast did not enhance the benefit of CPT, but the control group had greater use of another AFO.<br>However, SWIFT remains a clinical option because of its low cost & custom-madeby therapists	3
**262**	Mansfield et.al, 2015	Canada	P: Individuals with subacute stroke<br>Sample size=60 (E=29, C=31),<br>**Mean Age** (yrs)<br>E=64,C=61.5<br>Age range=E=22-92yrs,C=24-81yrs<br>PSD (days): E= 26±22 (11-114),<br>C= 23±20 (12-72)<br>TOS(ischemic/hemorrhagic):<br>E = 24/5; C =22/6	Accelerometers + daily feedback about walking activity	Accelerometers around both ankles (no feedback)	- Self Efficacy (SEQ),<br>- Change in walking activity(Accelerometer),<br>- Spatiotemporal features (4-m-long pressure sensitive mat)	- no significant increase in walking time, number of steps, longest bout duration, or number of long walking bouts for the feedback group compared with the control group (P >0.20); except cadence of walking that was significantly higher in the feedback group (P = 0.013).<br>- For the laboratory gait assessment, feedback had a greater increase in walking speed and decrease in step time variability than the control group (P < 0.030).	Feedback did not increase the amount of walking completed by individuals with stroke. However, there was a significant increase in cadence	3

**263**	Kim et.al, 2014a	South Korea	P: First unilateral stroke ≤ 6mnths Sample size=20 (E=10, C=10), **Age** (yrs) E= 54.10±11.69, C= 53.90±5.82 **PSD** (months): E= 13.76±4.02, C= 13.50±2.76 **TOS**: NA	Respiratory training using RESPIFIT S® +Conventional exercise treat-ment + Automated full-body work -out machine	Conventiona l exercise treatment + Automated full-body workout machine	- Pulmonary function (FVC, FEV1, FEV1/FVC) - Functional walking ability & endurance (6MWT) -Level of breathless-ness (SBMBDS)	A significant intergroup difference was observed in the FVC, FEV1, PEF, 6MWT, and SBMBDS scores (p<0.05) with the intervention group recording statistically significant improvement in these variables	RESPIFITS had a positive effect on pulmonary function and exercise capacity and may be used for breathing rehabilitation in stroke patients	3
**264**	Langha mmer et all, 2014	Norw ay	**Sample size:** I= 19 C= 18 **Age Range:** NR **Mean Age:** I=72.3 +14.2 C= 77.7 +8.9) **PSD(year):** I = <1 C = <1 **TOS(ischemic/he morrhagic):**NR	intensive exercise report of the 4 years follow-up	regular exercise	ADL (BI); IAD (Duke OARS); motor function (MAS); Walking capacity (6MWT); balance (BBS); functional mobility (TUG); grip strength (vigorimeter,); muscle tone (mAS); The Health-Related Quality of Life [HRQoL] (Nottingham Health Profile (NHP)	There was no significant differences in total scores on the BI (p=0.3), MAS (p=0.4), BBS (p=0.1), TUG (p=0.08), 6MWT (p=0.1), bilateral grip strength (Affected hand, p=0.8; non-affected hand, p=0.9) nor in the items of NHP (p>0.05).	This longitudinal study shows that persons with stroke in the two groups with different exercise regimes during the first year after stroke did not differ in long-term outcomes. Both groups maintained function and had a relatively active life style 4 years after the acute incident	2
**265**	Logan et al., 2014	UK	**Sample size:** I= 287, C= 281 **Age Range** (Yrs)**:** I=64–81 C= 63–81 **Mean Age** (Yrs)**:** I=71.7 +12.1 C= 71.5 +12.1 **PSD(months)** I=43.2 +60.1 C= 37 +43.8 **TOS(ischemic/he**	Verbal advice and transport and outdoor mobility leaflets	Outdoor mobility rehabilitatio n programme	Primary: health-related quality of life (the Short Form questionnaire-36 items, version 2 (Social Function domain). Secondary: functional ability, mobility, satisfaction with outdoor mobility (SWOM), psychological well-being and resource use (health care and Personal Social	There was no significant difference between the groups on health-related quality of life (social function). There were also no significant differences between groups in functional ability, psychological well-being or SWOM at 6- or 12-month follow-ups.	The outdoor mobility intervention provided in this study to these stroke patients was not clinically effective in relation to the measured outcomes	3

			**morrhagic):**NR			Services [SSS])			
266	Van Nunen et al, 2014	Netherland & UK	**Sample size:** I= 16; C= 14 **Mean Age:** I=50.0 + 9.6 C= 56.0 + 8.7 **PSD(days):** I=61.6 + 28.7 C=67.1 + 49.1 **TOS (ischemic/ hemorrhagic):** I=9/7; C=10/4	Conventional overground therapy. with Lokomat therapy (LOKOMAT)	Conventional assisted overground therapy only (Control)	PO: Walking speed [WS] (10MWT)<br><br>SO: Ambulation (FAC); Balance (BBS); Motricity index (MI), Motor impairment (FMA); mobility (RMI). Dynamic balance (TUG); Maximal Voluntary Isometric Muscle Torque	Significant within-group ↑in WS. Also, both groups significantly ↑ in all the secondary variables. Significant between-group difference in MI favouring LOKOMAT ($p < 0.05$); but no significant btwn-grop differences in FAC, BBS, RMI, FMA-leg and TUG ($p > 0.05$). No significant btwn-grp differences during the follow-up period from wk 10-24 or wk 10-36 for FAC, BBS, RMI, FMA leg and TUG	substituting Lokomat therapy for some of conventional therapy is as effective in recovering walking ability in non-ambulatory stroke patients as conventional therapy alone	3
**267**	Monticone, et al, 2013	Italy	**Sample size:** I=30, C=30 **Mean Age yrs:** **I=**62.1 ±9.7 C=60.2 ±6.1 **PSD(days):** I= 21.0 ±7.8 C= 21.2 ±8.9 **TOS: ischemic/hemorrhagic :** NR	20 sessions of neuromotor exercises (e.g. sit-to-stand, balance, gait training) wearing the 'Regent Suit'. (REGENT)	20 sessions of neuromotor exercises (e.g. sit-to-stand, balance, gait training) without the 'Regent Suit' (CG)	Gait speed (6 minutest walk test); balance (Berg Balance Scale); ADL (BI and FIM); Global satisfaction with treatment (Global Perceived Effect scale)	REGENT showed ↑er significant ↑ in GS & balance. Significant effect of time, group, and time-by-group interaction on GS in favour of REGENT. Significant effect of time and time-by-group interaction on BBS favouring REGENT, while no significant group effect was found. REGENT also showed ↑er significant ↑ than CG in ADL: there was a significant effect of time, group, and time-by-group interaction for both BI & FMI. In both groups.	Neuromotor exercise using the 'Regent Suit' is more effective than neuromotor exercise without the 'Regent Suit' in improving locomotion and daily living activities in patients with subacute stroke	3
**268**	Hsu et al., 2013	Taiwan	**Sample size:** I=11; C=12 **Mean Age** (Yrs)**:** I=51.1+14.0 C= 52.6+13.3 **PSD(months):** I= 5.8+4.2 C= 9.4+7.1	30-minute noxious thermal stimulation (TS) protocol with physical therapy	30-minute non-noxious thermal stimulation (NTS) protocol with physical	PO: Lower extremely [LE] impairment (LE-STREAM), Mobility(Mob-STREAM) Ability to walk without using devices (FAC). SO: ADL (BI), Balance (PASS), and	Significant time effect improvement in TS in all the outcomes except balance and spasticity whereas there was no measurable change in NTS at post intervention and follow-up. No significant change in outcome measures between the	8-week program of noxious TS combined with a traditional stroke rehabilitation program can improve the LE-related movement	5

			**TOS: ischemic/ hemorrhagic :** I = 7/4; C = 4/8		therapy	Spasticity (mAS).	groups at post intervention, but during the 1 month follow-up in LE-ESTREAM, Mob-STREAM, BI & mAS (P<0.05)	and function in patients with stroke for more than 3 months.	
**269**	Morris, & Wijck, 2012	UK	**Sample size:** I=56 C=50 **Mean Age** (Yrs)**:** I= 67.9+11.7 C= 67.9+11.7 **PSD** (weeks)**:** I=2-4, C=2-4 **TOS:** NR	Training of the ipsilesional and contralesional arms	Training involving the contralesional arm only	Upper limb activity limitation (Action Research Arm Test [ARAT]); and dexterity: (Nine-Hole Peg Test [9HPT])	There was no significant difference in mean ARAT score change between the groups at 0 to 6 weeks nor 0 to 18 weeks (*P*>.05) Intervention group demonstrated significantly greater change in dexterity (*P*=.03) compared with the control group.	Bilateral limb training may lead to clinically small improvements in ipsilesional performance of fine, rapid dexterity tasks.	3
**270**	Zedlitz, et al, 2012	Netherlands.	**Sample size:** I= 45, C= 38 **Mean Age** (Yrs): I= 54.8 + 9.1 C= 55.6 + 8.8 **PSD**: NR **TOS(**Ischemic/hemorrhagic/SAH/others) I = 31/3/7/4 C = 30/3/2/3	Cognitive therapy (CO) with graded activity training (GRAT), CO+GRAT	Cognitive therapy (CO)	Fatigue (CIS-f & SOL-f) Depression and anxiety (HADS), Functional Health Status (SA-SIP 30), Physical Endurance (6MWT)), Patients satisfaction (VAS)	Both groups showed significant beneficial effects on fatigue (*P*<0.001) and other outcomes (except pain and anxiety) with ITT analyses. Gains for the intervention group exceeded those in the Control group on number of individuals showing clinical improvement on the CIS-f (>8 points: 58% versus 24%) and on physical endurance (η_p^2=0.20, p<0.001).	A 12-week cognitive therapy program can alleviate persistent fatigue after stroke. The best results are obtained when cognitive therapy is augmented with graded activity training	3
**271**	Tilson et al., 2011	Los Angeles, CA	Sample size = NR **Age Range:** NR **Mean Age**: NR **PSD**: NR **TOS:** NR	G1 = early specialized locomotor training program (LTP) G2 = late a specialized locomotor training program (LTP)	progressive strength and balance exercise program	Fall incidence (monthly fall calendar), Nature of fall (standardized questionnaire)	Most falls occurred at home in the first 3 months after assessment. Falls incidence was highest for those with severe walking impairment who received early-LTP (*P*=0.025)	As individuals with stroke improve in walking capacity, risk for injurious falls remains high. Individuals with severe walking impairement who received early-LTP training are at higher risk of falls.	2
**272**	Braun et	Nethe	**Sample size:**	Multi	Multi-	PO: Patient-perceived	There was no significant	No effect in favor	3

	al., (2012)	rland	I= 18, C= 18<br>**Mean Age:**<br>I= 77.7 + 7.2<br>C= 77.9 + 7.4<br>**PSD:**<br>I= 4.8 + 3.3<br>C= 6.1 + 2.7<br>**TOS:** NR	professional therapy with instruction on **mental practice** with a 4-step framework embedded in regular therapy time.	professional therapy only	performance of daily activities (10-point NRS).<br>SO: Voluntary activity and strength (MI)<br>Independent performance of daily activities (BI),<br>Fine hand coordination (NHPT),<br>Balance (BBS),<br>Mobility disability (RMI),<br>Walking speed (10MWT)	between group differences detected in all the measured outcomes.	of the mental practice intervention on any outcome measure could be detected at either measuring points.	
**273**	Erel, et al (2011)	Turkey	**Sample size:**<br>**I=** 14, C= 14<br>**Mean Age** (yrs)**:**<br>I= 42.50 +14.89<br>C= 50.64+ 9.22<br>**PSD(months):**<br>I= 30.21 +13.84<br>C = 25.36 +13.44<br>**TOS (ischemic/ hemorrhagic):**<br>I = 3/11, C = 1/13	wore tennis shoes with **dynamic ankle foot orthosis** for three months	Wore only tennis shoes for three months	limits of stability (Functional Reach test);<br>Mobility (TUG);<br>Gait velocity (Timed-Up-Stairs, Timed-Down-Stairs & 100MWT) ;<br>Increased heart rate required for walking (Physiological Cost Index)	There was a significant between groupdifference in favour of the study group for gait velocity and Physiological Cost Index (P<0.05). No difference was found between the groups for Functional Reach, Timed Up and Go, Timed Down Stairs (P>0.05).	Chronic hemiparetic patients may benefit from using dynamic ankle-foot orthosis.	3
**274**	Britto et al., (2011)	Brazil	**Sample size:**<br>I= 9, C= 9<br>**Mean Age:**<br>I= 56.66+5.56<br>C = 51.44+15.98<br>**PSD:** NR<br>**TOS(ischemic/hemorrhagic):** NR	Maximal inspiratory pressure [MIP] with resistance adjusted biweekly to 30% (MVP+R)	MIP without the threshold resistance valve. (MVP)	Maximal inspiratory pressure [MIP] (a manovacuometer);<br>Inspiratory muscular endurance [IME] (The Threshold); functional performances (cycle ergometer test and HAP) and QoL (Brazilian version of the NHP)	There were significant between-group differences for the MIP and IME measures (p < 0.05). Significant changes were observed for only the experimental group for MIP and IME (p < 0.05). No statistically significant between nor within groupdifferences were observed for measures of functional performance and QOL.	MVP+R may benefit people with stroke, and it is feasible to be included in rehabilitation interventions with this population	2
**275**	Bovend' Eerdt, et l., 2010	Netherlands	**Sample size:**<br>**I=**15, C=15<br>**Mean Age (**Yrs**):**<br>I=51.2+11.75<br>C= 50.6 +16.48	Motor Imagery embedded in Usual Therapy (MI+UT)	Usual Therapy (UT)	PO: Set Goals (Goal Attainment Scale [GTS]).<br>SO: ADL (BIl)<br>-Mobility (RMI), Funct-ional mobility (TUG),	GTS significantly ↑ed at post intervention and follow-up (P<.001) in both groups, but no significant betwn-grp diff (P=.845). The interaction effect	Both interventions provided a comparable improvement in the measured	3

			**PSD(weeks):**<br>I= 15.9±17.25<br>C=21.8±15.17<br>**TOS:** NR			Arm Motor Function (ARAT)<br>ADL (Nottingham Extended ADL scale) , Self efficacy & perceived effort in relation to imagine performing tasks (A custom-developed questionnaire)	(group X time) was not significant (P=0.919). There were ↑ in the entire SO in both groups; The between group change in TUG was not significant (P=0.691). Imagery Questionnaire and has similar results as the other outcome measures.	outcomes..	
**276**	Tung, et al 2010	Taiwa n	**Sample size:**<br>**I=**16, C=16<br>**Age Range** (Yrs):<br>I=27–74<br>C= 27–73<br>**Mean Age** (Yrs):<br>**I=**51.0 ±12.1<br>C=52.7 ±14.1<br>**PSD(months):**<br>I=26.9 ±16.0<br>C=12.8 ±12.3<br>**TOS:** NR	General physical therapy plus additional 15 minutes of sit-to-stand training per session.(SST)	General physical therapy (CG)	Standing balance ability (Balance Master System[BMS]), dynamic balance (limit of stability testing[LST]), static and dynamic balance during ADL (Berg Balance Scale [BBS])	SST recorded a significant improvement in all the outcome measures (p <0.05) CG recorded significant improvement in only the BBS (p<0.001). A significant between group change was found only in the dynamic balance: dynamic balance of the affected part (p = 0.028) and sit-to-stand (p = 0.032)	Additional sit-to-stand training is encouraged due to effects on dynamic balance and extensor muscles strength in subjects with stroke.	3
**277**	Yang, et al., 2010	Taiwa n	**Sample size:**<br>E1=5, E2=5,<br>C1=9, C2 =9<br>**Mean Age** (Yrs):<br>**E1 =** 56.8±1.3<br>E2 = 57.5±6.1<br>C1 = 61.8±3.8<br>C2 = 48.1±3.7<br>**PSD(years):**<br>**E1 =** 0.3±0.1<br>E2 = 2.1±0.3<br>C1 = 0.3±0.0<br>C2 = 2.9±0.5<br>**TOS(ischemic/hemorrhagic):**<br>**E1 =** 4/1, E2= 3/2<br>C1=3/12, C2=1/3	E: 30-minute BWSTT followed by the 20-minute general exercise program<br>*Subgroups*:<br>E1:short duration and E2:long duration post stroke	C: General exercise program<br>*Subgroups:*<br>C1:short duration and C2:long duration post stroke	Corticomotor activity (motor threshold [MT] and map size of AH elicited by Trans Magnetic Stimulation), Motor control (Fugl-Meyer Assessment Scale [FMA])	↓MT: E1>E2, C1>C2<br>↓MT: E1>C1, E2>C2<br>Sig ↓MT for E1 (*P*=.025), but not in E2.<br><br>**Motor Control**<br>↑MC: E>Cl,<br>Sig ↑FMA for E1 (*P*=.001) and E2 (*P*=.001). However, the FMA scores remained unchanged over time for subjects in control group	The BWSTT results in higher improvement in motor control and also in different patterns of treatment-induced cortical reorganization in subjects with different poststroke durations.	3
**278**	Devos,et	Georg	**Sample size:**	Simulator-	cognitive	On-road driving	SD sig> CD in the overall on-	Training in a	2

	al., 2009	ia	I=42 C=41<br><br>**Age Range:** NR<br><br>**Mean Age:** NR<br><br>**PSD:** NR<br><br>**TOS(Ischemic/hemorrhagic):** NR	based driving training (SD)	based driving training (CD)	performance(Test Ride for Investigating Practical fitness to drive (TRIP)–Belgian version)	road score and the items of anticipation and perception of signs, visual behavior and communication, quality of traffic participation, and turning left. There were significant mean difference in driving performance between the groups; most of the differences in were observed at 6 months poststroke.	driving simulator appeared to be superior to cognitive training to treat impaired on-road driving skills after stroke.	
**279**	Langhammer, et al, 2009	Norway/Sweden	**Sample size:** I=35, C=40<br>**Mean Age** (Yrs)**:** I=76 ±12.7<br>C=72 ±13.6<br>**PSD(days):** NR<br>**TOS:** NR	Intensive exercise (I-Ex)	Self-initiated Exercise (S-Ex)	IADL [OARS]; Motor function [MAS]; Walking capacity [6MWT]; Balance [BBS]; dynamic balance, gait, and transfers [TUG]; grip strength (Martin vigorimeter); resistance to passive movement (mAS); Pulse rate (pulse monitor, Sport Testert); subjective impression of life after stroke (oral interview)	Both groups reported a higher level of activity in all the items. However, Only item 1 (can you use the telephone) was significantly different between the groups; all other items were not. functional activities as measured by MAS, 6MWT, BBS, TUG, and grip strength improved significantly in both groups from admission to 12 months after stroke. No sig btwn-grp diff	Intensive exercise and self initiated exercise equally show individual improvement in motor functions, pulse rate, MAS but do not differ significantly	3
**280**	Langhammer et al, 2008	Norway & Sweden	**Sample size:** I= 35, C= 40<br>**Mean Age (yrs):** I= 76 ±12.7<br>C= 72 ± 13.6<br>**PSD(days):** I=22 ± 13<br>C= 16 ± 10<br>**TOS(ischemic/hemorrhagic):** NR	Intensive exercise	Regular exercise	Motor function (MAS); balance (BBS); mobility; walking speed and length (6MWT); personal ADL (BI); IADL (the Duke OARS); health related quality of life HRQoL](Nottingham Health Profile [NHP])	No significant difference in mean NHP was seen between the groups. Significant difference in mean physical mobility subscales was found between the two groups in favour of the control group ($p < 0.05$). no other within nor bee\tween group differences were seen in the rest of the outcomes.	Regular exercise with self-initiated training seemed to enhance HRQoL more than intensive exercise	3

**281**	Mead et al., 2007	UK	**Sample size:** I= 32 C= 34<br><br>**Age Range:** NR<br><br>**Mean Age:**<br>I=72.0 ± 10.4<br>C= 71.7 ± 9.6<br><br>**PSD(days):**<br>I=171<br>C= 147.5<br><br>**TOS(ischemic/ hemorrhagic):**<br>1 = 28/4<br>C = 32/2	Exercise training (including progressive endurance and resistance training) (ExT)	Relaxation (Rlx)	ADL (FIM & NEADLs); mobility function (RMI); functional reach; (TUG); sit-to stand time (EMS); ambulation (FAC); QoL (SF-36), mood (HADS). Comfortable walking velocity (10MWT); walking economy; Leg extensor explosive power (NPR)	ExT: Sig within-grp ↑for SF-36 role-physical, EMS, NPR paretic, and 10MWT (all P<.001); SF-36 general health, SF-36 mental health, TUG, and HADS anxiety (all P<.01); SF-36 vitality, SF-36 role-emotional, functional reach, NPR nonparetic, and walking economy (all P<.05).<br>At 7 months, sig↑s were maintained only in NPR of both lower limbs (P<.01),<br>Rlx: Significant within-grp ↑ in 10MWT (P<.001), SF-36 mental health and NPR nonparetic (both P<.05). Sig↑ was maintained in all of these outcomes at 7 months (P<.05, P<.01, and P<.001, respectively), but SF-36 bodily pain was sig worse (P<.01)	Exercise training for ambulatory stroke patients was feasible and led to significantly greater benefits in aspects of physical function and perceived effect of physical health on daily life.	3
**282**	Pang, et al, 2005	Canada	**Sample size:**<br>**I=** 32, C= 21<br>**Mean Age** (Yrs):<br>**I=** 65.8 ± 9.1<br>C= 64.7 ± 8.4<br><br>**PSD(years):**<br>I = 5.2 ± 5.0<br>C = 5.1± 3.6<br>**TOS:** Ischaemic/ Haemorrhagic<br>I=18/14, C=19/2	Fitness and mobility exercise program (FAME)	Seated upper extremity program	VO2max (cycle ergometer); mobility (6MWT); muscle strength (hand held dynamometry) ; functional balance BBS); activity /participation (physical activity scale for individuals with physical disabilities [PASIPD]);	Significant group x time interaction (p = 0.004) indicating that overall, the FAME program produced more gains than the control. Post hoc analysis revealed that the intervention group has significantly more improvement in VO2max, 6MWT distance, and paretic leg muscle strength than control. There were no group x time interaction for balance, activity and participation, and non-paretic leg muscle strength	FAME program is feasible and beneficial in improving some of the secondary complications resulting from physical inactivity in older adults with stroke.	3
**283**	Wang, et al, 2005	Taiwan	**Sample size:** NR<br><br>**Age Range:** NR	Bobath programme (Bobath)	orthopaedic treatment programme	Impairment levels (Stroke Impairment Assessment Set (SIAS); functional	Spasticity: both groups, sig ↑ in SIAS motor control of LE (p < 0.01), MAS (p <0.001) &	Both treatments rulted in improvements in	3

			**Mean Age:** NR<br><br>**PSD:** NR<br>**TOS(ischemic/hemorrhagic):**NR		(Ortho)	limitation levels (Motor Assessment Scale (MAS)); balance (Berg Balance Scale (BBS)) stroke-specific outcome (Stroke Impact Scale (SIS)).	BBS (p <0.001). Bobath: sig ↑ in SIAS tone (p = 0.005) & SIS (p = 0.005), unlike Ortho..<br>Bobath: sig >↑in tone control (p=0.006), MAS (p= 0.01), and SIS (p =0.023. No sig btw-grp diff in BBS, LE-motor control (SIAS)<br>Relative recovery: both groups, sig ↑ in BBS score (p <0.001) & SIS (p <0.01). Bobath: sig↑ in MAS (p 0.001), not found in Ortho.	impairment and functional levels for patient with stroke.<br>Patients benefited more from the Bobath in MAS and SIS scores than from Ortho regardless of their motor recovery stages.	
**284**	Blennerhassett & Dite, 2004	Australia	**Sample size:**<br>I= 15, C= 15<br>**Mean Age** (yrs)**:**<br>I= 53.9 ±19.8<br>C= 56.3±10.5<br>**PSD(days):**<br>I= 36.0±25.1<br>C= 50.1 ±49.2<br>**TOS(ischemic/hemorrhagic):**<br>I= 11/4, C= 11/4	Conventional rehabilitation with Mobility activities (Mob)	Conventional rehabilitation with upper limb activities (UL)	For locomotor performance: (6MWT, step test, TUGT),.<br><br>For upper limb activities: (MASand the combined time for three sub-tests from the Jebsen Taylor Hand Function Test (JTHFT))	Mob: Sig↑ 6MWT, Step Test, and TUGT (all $p < 0.001$).<br>UL: Sig ↑ 6MWT ($p < 0.001$), Step Test ($p = 0.001$), TUGT ($p = 0.006$), and JTHFT ($p = 0.005$).<br>Sig (group x time) for the 6MWT ($p = 0.001$), and TUGT ($p = 0.009$).	Additional task-related practice improves mobility and upper limb function early after stroke.	3
**285**	The Glasgow Augmented Physiotherapy Study (GAPS) group, 2004	UK	**Sample size:**<br>I= 35, C= 35<br>**Mean Age** (Yrs)**:**<br>I= 31±11<br>C= 67±10<br>**PSD(days):**<br>I= 22±14<br>C= 25±18<br>**TOS(ischemic/hemorrhagic):**NR	Augmented physiotherapy (double the amount of control) (A-PT)	Conventional physiotherapy (C-PT)	Patient activity (an automated system; Recovery of impairment (Trunk Control Test and MI); Recovery of walking: [RMI] (BI and Nottingham Extended ADL); length of stay in the hospital and the presence or absence of various complication.	Both groups did not show any significant improvement in all the outcomes measured; there was also no significant difference between the groups in all the outcomes	Augmented physiotherapy did not provide any better improvement in mobility of post stroke patients	1
**286**	Byl et al., 2003	San Francisco	**Sample size:** NR<br><br>**Age Range:** NR<br><br>**Mean Age:** NR	First 4 weeks (F4w) sensory training, then Second 4 weeks (S4w)	F4w motr training, then S4w sensory training	sensory discrimination [SD] (kinesthesia, graphesthesia, and stereognosis), fine motor control [FMC] (digital	Sig ↑ for functional independence [FI] and performance parameters [PP] of UE ($P < 0.05$), FI ($P < 0.002$, motor UE ($P < 0.001$), sensory	There is significant improvement in function in the late poststroke	2

			**PSD:** NR<br><br>**TOS(ischemic/hemorrhagic):**NR	motor training (S-M)<br><br>NB: longitudinal crossover design	(M-S)	reaction time and performance time on the Purdue Peg Board), musculoskeletal performance [MP] for the upper and lower limb (strength and ROM), functional independence (WMFT] and the California Functional Evaluation), and gait speed	UE (*P* < 0.002). During sensory training, sig↑ in SD (*P* ≤ 0.005), FMC (*P* < 0.025), and musculoskeletal measurements of the UE (*P* < 0.05). During motor training, sig↑ in FMC (*P* < 0.02), MP of the UE (*P* < 0.05), and FI (*P* < 0.02). S-M showed sig↑ in SD (*P* < 0.025) and MP of the UE in the S4w (*P* < 0.005). In F4w, M-S made sig↑ in FMC (*P* < 0.005); sig↑d in FMC (*P* < 0.025) and SD (*P* < 0.05) in S4w. Over 8 weeks, M-S ↑> S-M in FMC (*P* < 0.025).	recovery period following 12 hours of supervised learning based sensory motor training	
**287**	Partridge et al., 2000	UK	**Sample size:** I= 49<br>C= 44<br><br>**Age Range:** NR<br><br>**Mean Age:** NR<br><br>**PSD:** NR<br><br>**TOS(ischemic/hemorrhagic):**NR	Double the standard amount of traditional physiotherapy	Traditional physiotherapy (standard amount)	PO: Gross Body Movements underlying function (Profiles of Recovery (POR) scale); Balance (functional reach test); step:time ratio (five metre timed walk test) and time taken to stand up from sitting in a chair (sit-to-stand test).<br>SO: psychological variables (HAD) and perceived personal control over recovery (Recovery Locus of Control Scale (RLOC))	Comparison of initial to six-week difference scores in the control and intervention groups of the whole sample did not show a significant difference	doubling the physiotherapy time available for patients in a stroke unit will not provide a measurable benefit for all patients	3
**288**	Rønning and Guldvog, 1998	Norway	**Sample size:**<br>I= 271, C= 279<br>**Mean Age** (Yrs):<br>I= 76.8±7.4<br>C= 76.1±7.0<br>**PSD:** NR<br>**TOS(ischemic/hemorrhagic):** NR	Allocated to a stroke unit (SU)	Allocated to a general medical ward (GMW)	Primary: death(collected through the National Register), need for long-term care, and number of patients who improved, deteriorated, or died<br><br>secondary: neurological	The improvement in all the outcomes from admission to follow-up at 7 months after stroke was highly significant within both groups but improvement in neurological score was significantly better among patients treated in the	The effects of stroke unit on the most reliable clinical outcomes were modest but didn't show any better effect compared to the	1

						impairment (the SSS); Disability (BI)	SU than in the GMW (*P*=.036). There was no significant difference in change in any of the other outcomes	general medical ward except in neurological improvement	
**289**	Kjendah at al, 1997	Norway	**Sample size:** I= 21<br>C= 20<br><br>**Age Range:** NR<br><br>**Mean Age:** NR<br><br>**PSD:** NR<br><br>**TOS(ischemic/hemorrhagic):** NR	acupuncture group [Accu]	Traditional neurorehabilitation [TNR]	ADL (Motor Assessment Scale for stroke patients [MAS]and Sunnaas Index of Activity of Daily Living (ADL)); subjective health status (Nottingham Health Profile (NHP))	NHP was sig ↑ed in both groups ($p \leq 0.0002$). Both Groups: sig within-grp diff in NPH with regard to changes over time from baseline to one year after (p = 0.0003), and also a sig diff one year after discharge ($p \leq 0.0001$). Also, sig btwn-grp diff in ADL in favour of Accu, both with regard to changes over time from first to third evaluation (p = 0.001), and with regard to the status after one year (p = 0.0002). ADL was sig ↑ed in Accu ($p \leq 0.0001$) while TNR remained almost unchanged (p = 0.50).	Acupuncture provides a positive effect to post troke patients in the subacute stage and these effect seems to be long-term	2
**290**	Hui, et al, 1995	Hong Kong	**Sample size:**<br>I= 59, C= 61<br>**Mean Age** (Yrs)**:**<br>I= 73.1±5.42<br>C= 74.1±5.89<br>**PSD (days):**<br>I= 6.47±3.62<br>C= 5.45±2.66<br>**TOS (ischemic/ hemorrhagic):**<br>NR	Care under the geriatricians (CuG)	conventional inpatient rehabilitation under a neurology team (CuN)	Details of hospital services received (number of visits to the Geriatric Day Hospital [GDH] where applicable, new hospital admissions, and outpatient visits); use of general practitioner services; use of community services (eg, community nurse, health visitor, and home help); functional evaluation (BI); information on well-being problems with sleeping; and assessment of mood (GDS); degree of patients' and	At 3M, both groups ↑ed in BI; but, no sig btwn-grp diff in BI at each assessment; By 6M, no sig diff in the number of cases in each BI category for the two groups. The BI overall CuG↑ed sig (*P*<.0005). At 3M, the cost per course of treatment for CuG was > CuN but not sig (*P*=.055). No sig btwn-grp diff in number of outpatient attendances and cost. Other parameters assessed, such as patient well-being (self-perceived health, sleep problems, GDS score), use of community services, and financial support, were all	Compared with conventional medical management, care in the geriatric day hospital hastened functional recovery with an increased cost per course	2

						caregivers' satisfaction with services received on a scale of 1 (not satisfied) to 4 (very satisfied)).	comparable between the two groups at each follow-up		
**291**	DePippo et al, 1994	UK	**Sample size:**<br>I= 124, C= 121<br>**Mean Age** (Yrs)**:**<br>I= 77.8±11.4<br>C= 78.6±12.2<br>**PSD:** NR<br>**TOS (ischemic/ hemorrhagic):**<br>NR	Managed on a stroke rehabilitation unit	General medical wards	destination of discharge, functional abilities at discharge (median discharge Barthel ADL scores); length of hospital stay	There was sig ↑er length of hospital stay by individual who were managed on a rehabilitation unit compared with general wards (P<.001). No sig diff in functional abilities at discharge between the groups. Functional abilities at discharge, destination of discharge, and length of hospital stay in patients with good prognosis were comparable in both settings.	At discharge, the functional abilities of the patients in both groups remain comparable. Those managed on a stroke rehabilitation unit however showed higher length of hospital stay.	2
**292**	Sunderland et al., 1992	UK	**Sample size:**<br>1=35, C=36<br>**Median Age:**<br>I =60, C = 65<br>**PSD(days):**<br>I=10, C=8;<br>**TOS(I/H):** NR	Enhanced physical therapy[ET]	Orthodox physical therapy [OT]	Motor functions (EMI); Active ROM (MCbA); Passive Movt & Pain (present/absent);Dexterity (FAI); Sensory loss (light touch); Functional independence (BI)	ET&OT: sig (grp x time) on the EMI (p=0006). No sigeffect on BI (therapyxtime, p > 0 2). At 6M: ET arm function > OT patients; ET>OT on all measures except the FAI.	There are gains in patients gains in recovery due to enhanced physical therapy.	3

FIM: Functional Independence Measure
BBS: Berg's Balance Scale
RoF: Risk of Fall
TUG: Time Up and Go test
SF-36: The Medical Outcomes Study 36-Item Short form Questionnaire
CAHAI-7: Chedoke Arm and Hand Activity Inventory
UE: Upper Extremity
FMA: Fugl Meyer Motor Assessment Scale
mMAS: modifies Ashworth Scale
BI: Barthel Index
FAC:
OARS: Older Americans Resources and Service Procedures
RMI: Rivermead Mobility Index
PASS: Postural Assessment Scale for Stroke Patients
CIS-f: Checklist Individual Strength–subscale Fatigue

SOL-f: Self Observation List – Fatigue subscale
HADS: Hospital Anxiety and Depression Scale
SA-SIP: Stroke-Adapted Sickness Impact Profile 30
NRS: Numeric Rating Scale
MI: Motricity Index
NHPT: Nine Hole Peg Test
10MWT: Ten Meters Walking Time
ARAT: Action Research Arm Test
BWSTT: Body Weight Supported Treadmill Training
EMI: Extended Motricity Index
MCbA: Motor Club Assessment
FAI: Frenchay Arm Index
SBMBDS: Shortness of Breadth Modified Borg Dyspnea Scale

Participation Based Therapy

Ref	Study (Authors, year	Country	Participants	Interventions/ Model of care	Control/ comparison	Outcomes assessed/ outcome measures	Results	Conclusion	Quality score
**293**	Graven et al, (2016)	Australia	110 participants<br>Age range (years):<br>C= 70.9 (13.2)<br>I= 68.9 (15.1)<br>PSD: ≥ 6 months<br>TOS (ischaemic /haemorrhagic):<br>C=39/16, I=30/24	A multimodal approach, including telephone contacts, screening for adverse sequelae, written information, home visits, review of goal achievement, and further referral to relevant health services.	Standard management	Geriatric Depression Scale (15 item; GDS-15)<br><br>Functional Independence Measure (FIM-Motor)<br><br>[uses were not stated]	There was a significant difference between the 2 groups with respect to the severity of depressive symptoms at 12 months post stroke ($R2=0.366$; $F(6, 89)=8.57$; $P<0.005$), with the intervention group recording lower depressive scores.	Community-based rehabilitation proved effective in reducing poststroke depressive symptoms.<br>An integrated approach using pursuit of patient-identifid activities should form part of routine poststroke management	3
**294**	Sabariego et al., 2013	Germany, UK & Switzerland	**Sample size:**<br>**I=110,** C=103<br>**Mean Age:**<br>**I=** 55.31 ±12.56<br>C=59.31 ±12.67<br>**PSD(weeks):**<br>I=151.10 ± 399.30<br>C=149.48±634.74<br>**TOS: (ischemic/ hemorrhagic):**<br>NR	Received ICF-based patient education programme	Received attention placebo: consisting of standardized lectures about stroke,	Primary: Self-efficacy regarding functioning (Liverpool Self-Efficacy Scale) Secondary: Life satisfaction (five items of the WHOQOL); self-perceived impact of stroke (SIS)	Over time, both groups showed improvement in self-efficacy ($p< 0.01$) and participation ($p<0.01$), while emotional functioning ($p < .01$) deteriorated, although no significant between-group differences were observed in those outccomes. And no significant within or between group change in SIS was observed.	There was no significant benefit of the ICF-based patient education in comparison with an attention-placebo control group	3
**295**	Gill & Sullivan, 2011		**Sample size:**<br>I = 14, C = 8<br>**Mean Age** (Yrs):<br>I = 60.75 ± 19.30<br>C= 67.75 ± 19.30<br>**PSD** (days):	Participated in the enhanced version of Stay Active and Stop Stroke (SASS) sessions,	Did not participate	Exercise believes (Cerebrovascular Attitudes and Beliefs Scale–Revised (CABS-R)) and participants stage of change (Stages	Selected beliefs changed significantly over time but not differentially across conditions. Beliefs that changed were (1) perceived susceptibility to stroke, and (2) perceived	Participated in the enhanced version of Stay Active and Stop Stroke (SASS) sessions did not provide	1

			I= 31.7 +24.56<br>C= 95.92 +47.21<br>**TOS**: NR			of Exercise Questionnaire (SOEQ))	benefit of exercise to reduce risk.	better improvement in exercise believes.	
**296**	Strasser et al., 2008	USA	**Sample size:** I=233,C=346<br>**Mean Age:** I=67.6+11.1<br>C= 66.9+11.9<br>**PSD:** NR<br>**TOS(ischemic/hemorrhagic):** NR	Treated by staff who participated in a team training program (TTP)	Treated by staff receiving information only (RIO)	Primary: motor skills (FIM). Secondary: length of stay on admission [LOS] and discharge disposition (discharged to home or discharged to other location).	Significant↑ FIM scores in the intervention group than in the control group (*P*=.029), with no corresponding intervention effect on the other 2 outcomes. There was a significant difference in improvement in motor FIM score between the groups (*P*=.038). There was no significant difference for the other 2 outcome	Stroke patients treated by staff who participated in a team training program were more likely to make functional gains than those treated by staff receiving information only	3
**297**	Olney et al., 2006	Canada	**Sample size:** I= 37 C= 35<br><br>**Age Range:** NR<br><br>**Mean Age:** I=63.5+12.0<br>C= 65.8+11.6<br><br>**PSD:** NR<br><br>**TOS:** NR	A supervised physical conditioning program 3 times a week for 10 weeks (SPC)	an instructional physical conditioning program with supervision 3 times a week for the first week followed by an unsupervised home training program for 9 weeks (UHP)	PO: Walking speed (6MWT).<br>SO: activity participation (Human Activity Profile [HAP]); Health Status(SF-36); muscle strength (hand-held dynamometry); Physiological Cost Index [PCI] (calculated as the difference between the walking heart rate and the resting heart rate divided by the average walking speed, is expressed in beats per meter)	6MWT ↑ed sig in both groups ($p < 0.05$) no sig btwn-grp diff. HAP ↑ed sig in SPC but not with UHP. SF-36 ↑ed sig but the UHP did not; btwn-grp diff was not sig. The Mental Component scale followed a similar trend. Strength did not change significantly over time in both groups, and PCI was significantly improved only at 1 year for the UHP.	Supervised exercise programs and unsupervised programs after initial supervised instruction both provided physical benefits that were retained for 1 year, although supervised programs showed trends to greater improvements in self-reported gains	3

Quality of Life Centred Therapy

Ref	Study (Authors, year	Country	Participants	Interventions/ Model of care	Control/ comparison	Outcomes assessed/ outcome measures	Results	Conclusion	Quality score
**298**	Pan et al.(2017)	China	120 participants<br>**Age** range (Yrs):<br>E= 63.50yrs<br>C= 64.50yrs<br>**PSD** (Months):<br>E= 1.98, C= 1.91<br>**TOS**: ischaemic/ haemorrhagic: N/S	Modified wheelchair arm support plus routine services	Routine services	-Visual Analogue Pain Scale or Numeric Pain Rating Scale used for the intensity of pain<br>-Upper Extremity Fugl-Meyer Assessment scale used to measure active movement at each joint of the upper extremity<br>-Modified Barthel Index and Quality of Life Index used to measure activities of daily life.	At four weeks, the median of pain intensity was higher in the control group (median, interquartile range = 3, 5.75 vs. 2, 3.75; P= 0.059). At 12 weeks, the median of pain intensity was higher in the control group (median, interquartile range = 3, 5.00 vs. 0, 1.00; P< 0.001). At 12 weeks, patients with shoulder pain were higher in the control group (6 vs. 1; P< 0.05).	The modified wheelchair arm-support could lead to the mitigation of hemiplegic shoulder pain and reduction in pain incidence in stroke patients	4
**299**	Ada et al (2016)	Australia<br><br>&<br><br>Norway	46 participants<br>Age range:<br>50 -85 years for both groups<br>PSD: N/A<br>TOS= both ischaemic and haemorrhagic [N/S]	Modified lap-tray while sitting and a triangular sling	Hemi-sling	Shoulder subluxation measured on x-ray<br><br>10-cm visual analogue scale was used to measure pain	There was a trend for the experimental group to develop less pain at rest (MD -0.7 out of 10, 95% CI -2.2 to 0.8) and during shoulder external rotation (MD -1.7 out of 10, 95% CI -3.7 to 0.3) and a trend towards having less contracture of shoulder external rotation (MD -10 deg, 95% CI -22 to 2)	A lap-tray during sitting combined with a triangular sling during standing is no more effective than a hemi-sling in preventing subluxation, pain, contracture and activity limitation in acute stroke survivors at risk of shoulder subluxation.	2
**227**	Aidar et al, (2016)	Brazil	24 subjects<br>Age range (ys):<br>E=51.7 ± 8.0<br>C= 52.5 ± 7.7	Strength training (ST)	Normal daily activities (NDA)	Generic Questionnaire for the Assessment of Quality of Life"-SF 36 "	There was a significant increase in quality of life from pre-test to post-test (Δ% = 21.47%; p = 0.021) in	There was an improvement in the measures of strength in ST, & a correlation	4

			PSD: N/A<br>TOS= ischaemic stroke				EG. There were significant differences in all indicators of quality of life between groups at 12 weeks. There were greater gains in strength in EG than in CG ($p \leq 0.05$).	btwn improvements in strength & QoL in patients who had previously suffered a stroke at least one year prior to study.	
**300**	Tibaek et al, 2017	Denmark	P: men with post stroke LUTS<br>Sample size=31;<br>Age (yrs):<br>E=68 (57–73);<br>C = 70(64-75);<br>PSD days m(IQR):<br>E= 60(50-87),<br>C= 55 (50-63);<br>TOS(ischemic/hemorrhagic):<br>E = 12/0<br>C =9/2	PFMT	Standard Rehab Prog (SRP)	Generic QoL (SF-36), Norctural QoL (N-QoL Questionnaire)	- Sig within-group ↑ in SF-36 [emotional role] (p=0.03) & [vitality] (p=0.03) in PFMT but not in SRP;<br>- No sig btwn-grp diff in SF36<br>-Sig within-group ↑ in N-QoL domains for bother/concern for both groups, & for sleep/ energy control in SRP<br>- No sig btwn-grp ↑ in N-QoL	PFMT may increase the emotional health &vitality dimensions of QoL	3
**301**	Wong et al, 2015	China	P: Stroke patients discharged home<br>Sample Size= 108 (E=54, C=54);<br>Mean age (yrs):E= 67.5±11.6, C= 71.5±11.6<br>PSD:NA<br>TOS(ischemic/hemorrhagic):<br>E = 47/7, C = 47/7	Transitional Care programme (TCP)<br>+<br>Routine hospital-based physical training programme (RPT)	Routine hospital-based physical training programme (RPT)	- QoL (SF-36 & WHO-QOL-SRPB);<br><br>- General Satisfaction (PSQ-HK)<br><br>Functional Performance (mBI);<br><br>Depressive symptom (CES-D)	- Sig btwn-grp diffs in QoL in both physical (F=10.15,P =0.002) and mental (F = 8.41, P=0.005) domains of SF-36. But, only the physical domain achieved a significant time intervention interaction effect (F = 7.73, P = 0.006). TCP+RPT had better WHO-QOL-SRPF, PSQ, mBI but lower CES-D scores than RPT	The TCP shares common features that have been proved to be effective when applied to chronically ill patients.	3
**302**	Ntsiea et al, 2014	South Africa	P: Pre-stroke workers;<br>Sample size= 80 (E=40, C=40);<br>Age (yrs): 45±8.7,<br>E= 45±8.5,<br>C= 44±8.9;<br>Age range 26-	Workplace intervention programme tailored according to functional ability and workplace	Usual stroke care which took into consideration job requireme	- Return to work rate (Return to Work Questionnaire),<br>- ADLs (BI),<br>- Mobility (mRMI),<br>- Global cognitive function (Montreal cognitive assessment)	- At six months follow-up 60% (n = 24) of stroke survivors in the intervention group returned to work compared to 20% (n = 8) in the control group (P <0.001).<br>- The odds ratio for return to work for stroke survivors in	A workplace intervention consisting of workability assessments and workplace visits was effective in facilitating return to	3

			60yr<br>PSD (wks): 4.6±1.8, E=4.4±1.9<br>C = 4.7±1.5;<br>TOS: NA	challenges of each stroke survivor	nts but without workplace intervention	and<br>- QoL (Stroke Specific QoL Scale)	the intervention group was 5.2.<br>- Participants that returned to work had better quality of life than those who did not return to work (P = 0.05).	work for stroke survivors	
303	Immink, et al, 2014	Australia	**Sample size:**<br>I=11, C=11<br>**Age Range (Yrs):**<br>I=32–85, C=24–91<br>**Mean Age (Yrs):**<br>I=56.1±13.6<br>C= 63.2±17.4<br>**PSD(months):**<br>I=81.6 ±77.5<br>C= 23.3±12.5<br>**TOS:**NR	Yoga therapy (YT)	No therapy (NT)	Manual dexterity (9HPT); Motor function (MAS); mobility (2MWD) ; Gait Speed (CGS);Static and dynamic balance (BBS); Depression (GDS); state anxiety (STAI-Y1) and trait anxiety (STAI-Y2); QoL<br>(SIS)	MAS and BBS: no sig main or interactions effects ($p > 0.05$). Both groups ↑ed sig in 2MWD; A sig main effect of time was evident for 2MWD ($P = .046$). No sigt within-grp Δ in 2MWD. No sig main effects/interactions STAI-Y1; (ICC = 0.11), but a sig main effect of time (P =0 .045) for STAI-Y2. A sig ↑ in SIS-Physical (P =0 .001) for YT but not for NT ($P = .663$)	Yoga therpy showed greater efficacy only in the physical domain score of SIS compared with no therapy. Both therapies appear to have eqiovocal effect on the other measured outcomes	3
**304**	Rochette et al, 2013	Canada	**Sample size:**<br>I= 92, C= 94<br>**Age Range (Yrs):**<br>I= 31–92,C=34–88<br>**Mean Age (Yrs):**<br>I=61.7±12.7<br>C= 63.2±12.4<br>**PSD(days):**<br>I=6.5±5.0<br>C=5.2±3.8<br>**TOS(I/H):**<br>I=85/5/2,<br>C=87/5/2	a multimodal intervention WE CALL (study initiated phone support/information provision)	a passive intervention YOU CALL (participant can contact a resource person)	PO: unplanned use of health services for an adverse event (frequency calendar); QoL (Quality of Life Index and Euroquol-5D)<br>SO: Planned use of health services (frequency calendar); depressive symptoms (BDI-II); participation (LIFE-H 3.1)	No significant differences were seen between groups at 6 months for any outcomes with both groups improving from baseline on all measures ($p < 0.05$). The only significant change for both groups from 6 months to 1 year was in the social domains of the LIFE-H	The 2 groups improved equally over time on the outcomes measured	3
**305**	Beinotti et al, 2013	Brazil	**Sample size:**<br>I= 10, C= 10<br>**Mean Age (Yrs):**<br>I= 59, C= 52<br>**PSD(months):**<br>I= 79, C= 62<br>**TOS(ischemic/he**	Conventional physiotherapy plus horseback riding therapy (HBRT)	Conventional physiotherapy only	Quality of life (Medical Outcomes Study 36-item Short-Form health survey (SF-36).)	HBRT: sig ↑QoL ($P = .004$). Sig ↑ functional capacity ($P = .02$), physical aspects ($P = .001$), and mental health ($P = .04$). The subdomains of pain ($P = .58$), general health state ($P = .11$), vitality ($P = .33$),	Supplementation of conventional physiotherapy with HBRT, applied in different contexts, may yield positive QOL outcomes for	2

			**morrhagic):**<br>I = 9/1, C = 8/2				and emotional aspects (*P* = .32) did not show significant differences.	people with stroke	
**242**	Lund, et al, 2011	Norway	**Sample size:**<br>I=39, C=47<br>**Mean Age** (Yrs):<br>I = 75 ± 7.2<br>C = 79 ± 6.5<br>**PSD** (days):<br>I= 161, C= 137<br>**TOS**: NR	A lifestyle course in combination with physical activity (LC+PT)	Physical activity alone (PT)	Perceived health and well-being (SF-36)); change in self-reported occupational performance and Satisfaction(COPM); depression (HAD); mobility (TUGt) and cognitive function (Trail Making Test A and B)	9M FU: SF-36 (all domains) ↑ed in both grps, but no sig btwn-grp diff.. Both groups sig ↑ed in HAD and depression scale but no sig btwn-grp diff. Sig ↑ in both groups were observed using the COMP, but there was no difference between the groups. No sig btwn-grp change in TUG. The Trail Making Test A showed significant ↑after 9M in PT. However, the Trail Making Test A and B did not demonstrate any sig btwn-grp diff.	Improvements were seen in both groups, but no statistically significant differences were found in the intervention group compared to controls. An intervention comprising regular group-based activity with peers may be sufficient in the long-term rehabilitation after stroke.	3
**306**	Markle-Reid et al., (2011)	Canada	**Sample size:**<br>I= 75.8, C= 70.6<br>**Mean Age** (yrs):<br>**I=** 75.8, C= 70.6<br>**PSD:** NR<br>**TOS (ischemic/ hemorrhagic):**<br>NR	12-month specialized, evidence-based rehabilitation strategy involving an interprofessional team	Usual home care services.	PO: HRQoL (SF-36).<br>SO*:* Physical Function (SIS-16); Perceived Social Support (PRO85-Part 2); Depression (CES-D); Anxiety and depression (Kessler-10); Cognitive Function (SPMSQ); Community Reintegration (RNLI); and no of strokes in 12 months after randomization	Compared with the usual care group, stroke survivors in the intervention group showed clinically important (although not statistically significant) greater improvements from baseline in mean SF-36 physical functioning score (p=0.24) and social functioning score (p=0.28). The groups did not differ for any of the secondary outcomes.	A 12-month specialized, interprofessional team is a feasible and acceptable approach to community-based stroke rehabilitation that produced greater improvements in quality of life compared to usual home care	2
**307**	Claiborne, 2006	New York	**Sample size:**<br>I= 16, C= 12<br>**Mean Age (Yrs):**<br>**I=** 70±13.97<br>C= 65±11.99<br>**PSD(days):**<br>I=21.25±10.41	Integrated biopsychosocial interventions with coordinated delivery of	Usual care	QoL (SF-36); Depression (GDS). Psychosocial functioning (a one-time psychosocial assessment consisting of five sections and a	There were no effects for SF-36 physical component summary (PCS) of SF-36. There was a significant interaction effect between the condition and time for the mental component summary	care coordination provided by social workers significantly improves quality of care	1

			C= 23.67±15.19 **TOS(ischemic/ hemorrhagic):**NR	care		total score); patient adherence to self-care(self reported questionnaire)	(MCS) of SF-36 ($p \leq .001$), GDS ($p \leq .001$), and adherence ($p \leq .05$)		
**308**	Boter, 2004	Netherlands	**Sample size:** I=**263**, C= 273 **Age Range (Yrs):** I= 52 - 76 C= 51 - 74 **Median Age (Yrs):** I= 66; C= 63 **PSD:NR** **TOS(ischemic/hemorrhagic):**NR	standard care plus outreach care (SC+OC)	standard care (SC)	PO: Dissatisfaction with stroke care (SASC-19) and QoL (SF-36) SO: Anxiety/Depression (HADS) and readmiss-ion since discharge. Others: ADL (BI); overall degree of independence (mRS); use of health care services since discharge (eg, general practitioner and therapy), and use of secondary prevention drugs since discharge	In both groups, 1/5th of the patients were dissatisfied with care received in the hospital, and half were dissatisfied with care received after discharge. Apart from better scores in “Role limitations due to emotional health” SC+OC vs SC = (60.1±43.2vs52.3±43.8), no other sig diffs. SC+OC had less use of rehabilitation services & ↓er anxiety scores than controls, but on other outcomes, no statistically significant differences were found.	outreach nursing stroke care was not effective in improving quality of life and dissatisfaction with care of recently discharged patients.	2
**309**	Fjaortoft et al, 2004	Norway.	**Sample size:** I= 133, C= 125 **Mean Age:** NR **PSD(weeks):** 1-2 **TOS(ischemic/hemorrhagic):** NR	Acute stroke unit care with extended stroke unit service (ESUS)	ordinary stroke unit service (OSUS)	PO: quality of life ((NHP). SO: complex physical activities and social functioning (FAI); mood (MADS1) and cognitive mental status((MMSE).	No sig diffs btwn the groups in all 6dimensions ofNHP: Sig↑ global NHP, ESUS> OSUS group (p=0.048). FAI, MADS, MMSE & Caregivers Strain Index, there were non-sig diff btwn the groups (p > 0.05), but ESUS>OSUS	Stroke unit treatment combined with early supported discharge in addition to reducing the length of hospital stay can improve long-term QoL.	2
**310**	Tibaek, et al, 2004	Denmark	**Sample size:** I= 12, C= 12 **Age Range:** I=56-72; C=52-75 **Mean Age:** I= 59; C=62 **PSD:** NR **TOS:**NR	standardised pelvic floor muscle training	Conventional treatment	Health related quality of life (Short Form 36 (SF-36) Health Survey Questionnaire); urinary incontinence (Incontinence Impact Questionnaire (IIQ))	The SF-36 and IIQ did not show significant difference between the two groups. No significant improvement was also seen among the two groups in the measured outcomes.	The two interventions showed no efficacy in the improvement of quality of life and urinary incontinence. This could be attributed to the small sample size	3
**31**	Sulch, et	U	**Sample size:**	Integrated	conventio	ADL (BI), anxiety and	No btwn-grp diff in mortality	ICP management	3

**1**	al 2000	K	I= 76, C= 76<br>**Mean Age:**<br>I= 75 +11<br>C= 74 +10<br>**PSD:** NR<br>**TOS(ischemic/hemorrhagic):**NR	Care Pathway (ICP) coordinated by an experienced nurse ICP)	nal multidisciplinary care (CMC)	depression (HADs); QoLe (Rankin Score21 and Euroqol); The total therapy time for physiotherapy and occupational therapy, mortality, cause of death, and discharge destination	rates, institutionalization,or length of hospital. CMC ↑ed in functional recovery sig faster btwn 4&12 wks (*P*<0.01) & had ↑er QoL at 12wks (p = 0.07) & 6M (p<0.005). No sig btwn-grp diffs in the duration of PT or OT received. The median BI & Rankin were comparable btwn the 2 groups at all points. CMC ↑sig faster btwn 4&12 wks (*P*<0.01)	offered no benefit over conventional multidisciplinary care on a stroke rehabilitation unit. Functional recovery was faster and Quality of Life outcomes better in patients receiving conventional multidisciplinary care.	
**312**	Mant, et al, 1998	UK	**Sample size:** I= 37<br>C= 34<br><br>**Age Range:** NR<br><br>**Mean Age:**<br>I= 7 0+1 1<br>C= 7 6+1 0<br><br>**PSD(months):** NR<br><br>**TOS(ischemic/hemorrhagic):**NR	Received, in addition to Traditional rehabilitation, an information pack containing various Stroke Association publications one month after their stroke, or at discharge from hospital	Traditional rehabilitation only	Knowledge and satisfaction with information received, patient behaviour in terms of access to community services and benefits and health status and QoL (interviewer-administered questionnaires developed specifically for this study); impairment (LHS)), mood (HAD-S); and the Dartmouth Coop Chart for the patient, and burden of care of the carers (CSI); and QoL of carers (SF-36)); disability (BI)	Carers in the intervention group were found to have significantly better mental health (*p* = 0.04). no other significant between group difference was found in all the measured outcomes.	Information about stroke improved the mental health of the carers in the intervention group. However, it did not provide any further and better beneficial effect in the outcomes compared to traditional rehabilitation only.	3
**313**	Indredavik, et al, 1998	Norway	**Sample size:**<br>I= 110<br>C= 110<br><br>**Age Range:** NR	Allocated to stroke unit (SU)	Allocated to general ward (GW)	Primary: ADL (BI); QoL [the NHP] and the FAI)<br><br>Secondary: QOL (VAS)	For NHP, Sig btwn-grp diff (SU>GW) for the dimensions of energy, emotional reactions, social isolation, physical mobility, and sleep (all *P<0.05)*; but not in the	stroke unit care improves different aspects of long-term quality of life for stroke patients	2

			**Mean Age:** NR<br><br>**PSD:** NR<br><br>**TOS(ischemic/hemorrhagic):** NR				category of pain (*P*=0.3186). SU had a sig ↑er FAI (*P*=0.0142). 5yrs Post-stroke, QoL was sig better in SU than GW when VAS results were used as an indication of QoL. No sig btwn-grp diff in BI		
**314**	Rønning & Guldvog, 1998	Norway	**Sample size:**<br>I= 127; C= 124<br>**Mean Age:**<br>I= 75.5±6.7<br>C= 76.5±6.4<br>**PSD(day):**<br>I= 9.4±5.7<br>C= 10.4±7.0<br>**TOS (ischemic/ hemorrhagic):**<br>NR	subacute rehabilitation in a hospital rehabilitation unit (HRU)	health services in the municipality (Mun)	Primary: death, need of long-term care, and number of patients disabled (BI index score of <75).<br><br>Secondary: neurological deficits (SSS), ADL (BI); and HRQoL (The Medical Outcomes Study Short Form (SF-36) physical and mental health summary scales)	Sig btwn-grp diff no of death/dependence (*P*=.01). No btwn-group diff in 7M survival rates (*P*=.11), Dependency in ADL (*P*=.07). Patients with BI <50 before rehabilitation had sig better outcome in HRU, with fewer patients becoming dependent (*P*=.005) and patients having higher SSS(*P*=.026) and BI scores (*P*=.005). No sig diff in HRQoL..	Subacute rehabilitation of stroke patients in a hospital-based rehabilitation unit improves outcome. Patients with moderate or severe stroke appear to benefit most.	2

9HPT- Nine Hole Peg Test
2MWD: Two-Minute Walk Distance
CGS: Comfortable Gait Speed
BBS: Berg Balance Scale
STAI: State Trait Anxiety Inventory
SIS: Stroke Impact Scale
BDI: Beck Depression Inventory II
LIFE-H: Assessment of Life Habits
PRO-85: Personal Resource Questionnaire
CES-D: Centre for Epidemiological Studies in Depression Scale
GDS: Geriatric Depression Scale
SASC: Satisfaction-With-Stroke-Care questionnaire
NHP: Nottingham Health Profile
FAI: Frenchay Activity Index
MAD: Montgomery-
Asberg Depression Scale
LHS: London Handicap Scale
CSI: Carer Strain Index
BI: Barthel Index

SF-36: The Medical Outcomes Study Short Form-36
SSS: Scandinavian Stroke Scale
HRQoL: Health Related Quality of Life

PNS = Peripheral Nerve Stimulation; mCIT = modified Constraint Induced Movement Therapy; WMFT = Wolf Motor Function Test; FMA = Fugl-Meyer Assessment Scale; ARAT = Action Reach ArmTest; UE = Upper Extremity; mAS = Modified Ashworth Scale; MBEST = Mini Balance Evaluation Systems Test, TUG = Time up and Go; 6MWT = Six minutes walk test;ABC =Activity Specific Balance; FAI = Frenchai Activity Index; CHIEF = Craig Hospital Inventory of Environmental Factors; SIS- = Stroke Impact Scale ; mCIMT = Modified Contraint Induced Movement Therapy; rTMS = Repetitive transcranial magnetic stimulation; BI = Barthel Index; HADS-Thai = Thai version of the Hospital Anxiety and Depression Scale; DEXA = Dual energy-ray Absorptometry; LWBV = Low intensity Whole Body Vibration; HWBV = High intensity Whole Body Vibration; MFT = Manual Function Test; VD = Vertical Displacement (Shoulder Subluxation); UL = Upper Limb; MAL = Motor Activity Log; VO2 peak, 6MWD = 6-minute walk distance, 30WT = 30-ft Walk Times; 48SC = 48-hr step counts; TDx = Treadmill; SAM = Step Activity Monitoring; BCI-FES = Brain–Computer Interface controlled Functional Electrical Stimulation; MAL = Motor Activity Log; mBI = modified Berthel Index; AOT = Action Observational Training; SMA = Stride Management Assist; FTST = Functional Task Specific Training; m = Median;

Community-based rehab

Ref	Study (Authors, year	Country	Participants	Interventions/ Model of care	Control/ comparison	Outcomes assessed/ outcome measures	Results	Conclusion	Quality score
315	Khan et al.(2016)	Australia	103Participants<br>Age (years):<br>E= 61.0±18.4<br>C= 64.1±18.6<br>PSD,(years)<br>E=2.4 (7.5)<br>=3.2 (5.7)<br>TOS: both ischaemic and haemorrhagic combined N/A	Enriched environmental activities programme	Usual ward activity	-Depression, Anxiety & Stress (DASS)<br>-Personal Mastery Beliefs (NIS&;MHLC)<br>Worth/Value (RSES)<br>Cognition (MoCA)<br>Function & dependency (FIM)<br>Health dimensions [mobility, self-care, daily activity, pain/discomfort, and anxiety/depression] (Euro-QoL-5D)	Compared with controls, the intervention group showed signifiant improvement at discharge in: DASS: "total", "depression", and "stress" subscales ($p < 0.05$ for all, with small effect sizes (η2) = 0.04–0.05); MoCA (p = 0.048, η2 = 0.04) and FIM motor (total and "selfcare", "mobility" subscales ($p < 0.05$ for all, with moderate effect sizes, η2 = 0.0–0.08).	An enriched environmental programme can produce signifiant improvements in functional and cognitive ability in inpatient neurological cohorts compared with routine ward activity programmes	4
316	Askim, et al., 2010	Norway	**Sample size:**<br>**I**=30, C=32<br>**Age** (Yrs)**:**<br>**I=**75.4+7.9<br>C=77.6 +9.6<br>**PSD(days):**<br>I=14.4 +7.4<br>C=14.8 +6.6<br>**TOS:** NR	4-week community-based intensive motor training program combined with early supported discharge after initial treatment	standard treatment	Primary: balance (Berg Balance Scale).<br>Secondary: ADL (Barthel Index), motor functions (Motor Assessment Scale), mobility (Step Test), walking speed (5-meter Walk Test), self reported influence of stroke related function on quality of life (Stroke Impact Scale)	At 12 weeks follow-up, there was no significant difference found between the groups on all measured outcomes. Mean (SD) score improved significantly (*P*<0.000) from baseline to 26-week follow-up in both groups on all reported outcome measures	Intensive motor training program did not show significant improvement of balance or any other functional outcomes.	3
317	Lincoln, et al, 2004	UK	**Sample size:**<br>I=189, C=232<br>**Age Range:**<br>I= 22–92<br>C= 25–101<br>**Mean Age:**	rehabilitation by a community stroke team (Comm)	routine care (RC)	Functional independence in personal activities of daily living (Barthel Index); independence in instrumental activities of daily living. (Extended	No sig diffs btwn both groups in: BI/E-ADL, GHQ, EuroQol or knowledge of stroke. Comm were sig more satisfied with the emotional support they had received (*p*	Comm were more satisfied with the emotional support they received and had equivalent outcomes in terms	1

			I= 72.8+11.4<br>C= 71.2+11.5<br>**PSD:NR**<br>**TOS (ischemic /hemorrhagic) :**NR			ADL); mood (General Health Questionnaire 12 (GHQ-12)); quality of life (European Quality of Life [Euroquo]) Satisfaction with care(4-point Likert sc)	< 0.01). No sig diff btwn the groups in satisfaction with practical help or overall satisfaction. Carers of Comm were under significantly less strain than carers in RC (*p* < 0.04). Carers of Comm were sig more satisfied with their knowledge of stroke (*p* < 0.01) and were more satisfied overall (*p* < 0.01).	of independence in ADL, QoL, knowledge of stroke and mood. Their carers were under less strain and were more satisfied with their knowledge of stroke recovery	
**318**	Donnelly, et al, 2004	UK	**Sample size:**<br>I= 54, C= 59<br>**Mean Age:**<br>NR<br>**PSD:** NR<br>**TOS(ischemic/ hemorrhagic):**<br>NR	community-based multidisciplinary stroke team (CST)	hospital-based rehabilitation (usual inpatient rehabilitation)	ADL (BI & the Nottingham ADL measure); QoL (SF-36); patient and carer satisfaction and the Carer Strain Index (Service Use Questionnaire); cost of hospital care (Financial Accounts); home-based rehabilitation and community health and social services (SUQ, UCHSC)	The intervention group experienced higher satisfaction with services (p<0.01) and spent 202 fewer inpatient days than the control group. No other significant between group differences was seen in the rest of the outcomes. There was a significant improvement in quality of life for both groups.	Apart from higher satisfaction of service, community based multidicoplinary stroke team management provided no better improvement in quality of life than the usual in patient care to stroke patients	3
**319**	Green, et al, 2002	UK	**Sample size:**<br>I= 85, C= 85<br>**Mean Age:**<br>I= 71·5 ±8·7<br>C= 73·5 ± 8·3<br>**PSD:** NR<br>**TOS (ischemic/ hemorrhagic):**<br>NR	community physiotherapy treatment	No treatment	Mobility (Rivermead mobility index); walking speed (10 minutes walk test); ADL (Barthel index); social activity (the Frenchay activities index); mood (hospital anxiety and depression scale)	Apart from gait speed which showed significant difference between the groups at 3 months, (p=0·027). No significant difference in any of the outcomes measured were found between the groups	Community physiotherapy treatment provided only higher gait speed compared with no treatment.	3

DASS: Depression, Anxiety Stress Scale, NIS: Neurological Impairment Scale, MHLC: Multidimensional Health Locus of Control
RSES: Rosenberg Self-Esteem Scale, MoCA: Montreal Cognitive Assessment, FIM: Functional Independence Measure
SUQ: Service Use Questionnaire , UCHSC: Unit Costs of Health and Social Care

Home-Based Rehab

Ref	Study (Authors, year	Country	Participants	Interventions / Model of care	Control/ comparison	Outcomes assessed/ outcome measures	Results	Conclusion	Quality score
133	Emmerson et al, (2017)	Australia	62 Participants<br><br>Age (years), E=68±15 C=63±18 PSDdays, median (IQR): E=122(77-193) C=133(58–228) TOS(ischaemic/ haemorrhagic): E=22/6,C=27/3	Home exercise programme filmed on an electronic tablet, with an automated reminder	Paper-based home exercise programme	Wolf Motor Function Test used as an index of upper limb motor ability, measured through a series of 15 timed functional tasks, and two strength based tasks	No sig diff btwn the groups for measures of adherence (MD:2%, 95% CI −12 to 17) or change in the WMFT (MD: 0.02secs, 95% CI −0.1 to 0.1). No sig btwn-grp diffs in how participants found instructions (p = 0.452), whether they remembered to do their exercises(p = 0.485), or enjoyed doing so (p = 0.864).	The use of smart technology was not superior to standard paper-based home exercise programmes for patients recovering from stroke.	**3**
**320**	Zondervan et al, (2016)	California	**17 participants Mean Age, yr C=59 (35–74) E= 60 (45–74) PSD, yr C=3.17 ± 1.66 E= 5.33 ± 4.14 TOS=both ischaemic and haemorrhagic [N/S]**	Music Glove therapy	Conventional therapy	QoM, AoU, MAL, NHPT, Ability to manipulate various sized objects (ARAT), GDS Impairment (FMA), NIHSS and mAS for the wrist	The MusicGlove group did exhibit significantly greater improvements than the conventional exercise group in Motor Activity Log Quality of Movement and Amount of Use scores 1 mo post therapy (p = 0.007 and p = 0.04, respectively)	MusicGlove is viable and effective for home therapy, and it motivated users to complete a high number of therapeutic gripping movements.	4
**321**	Chen et al (2017)	China.	**97 partcipants Age (yrs): E=66.52±12.08 C=66.15±12.33 PSD (day): E=16.41 ± 3.74 C=16.93 ± 3.25 TOS: (I/H)**	Home-based telesupervising rehabilitation group (HTR)	Conventional rehabilitation (COR)	Disability/ADL (mBI) Balance (BBS) Functional outcome (mRS) Career stress (CSI)	Both groups showed sig ↑ in mBI (55.56–67.31 in the HTR groups, 54.26–66.04 in the COR group; F = 138.965; P < 0.001)	HTR is most likely as effective as the COR for ↑ing functional recovery in stroke survivors & could ease caregivers' burden	4

			**E=24/3, C=22/5**						
**322**	Rasmussen et.al, 2016	Denmark	**Sample size = E=38, C=33**<br>**Age (Yrs): E=78 (72-84) C=79 (71-85)**<br>**PSD: E= 18 (16–21), C= 16 (12–21)**<br>**TOS: NA**	HBR during hospitalization and for up to four weeks after discharge (HBR)	Treatment and rehabilitation following usual Care (UC)	- Stroke disability rate (mRS),<br>- Daily Activities (mBI),<br>- Cognitive function (CT-50CT Cognitive Test),<br>- QoL (EuroQol-5D™)	- HBR had > mRS (P=0.04); EuroQol-5 (P=0.03).<br>- The total amount of HBR training in minutes highly correlated with mRS, BI, MAS & EuroQol-5D™ (P<0.00001 to P=0.01).<br>- Economical estimations of HBR costs were 0.2% lower than total costs of UC	Early home-based rehabilitation reduced disability and increased QoL, and was more cost-effective	3
**323**	Zonderva n et al, 2015	USA	P: Persons living with severe arm impairment after stroke;<br>Sample size= 16 (E=8, C=8);<br>Mean age (yrs): E= 61 ± 17, C= 54 ± 14;<br>PSD (months): E = 39 ± 46, C = 19 ± 9;<br>TOS: NA	Resonating Arm Exerciser (RAE)	Conventional Therapy	- UL Motor Impairment (UE-FMA),<br>- Spasticity (mAS),<br>- Pain Intensity (VAS),<br>- Motor Activity (MAL)<br>- fine motor skill (BBT)<br>- Shoulder & Elbow ROM (Goniometer)	Sig ↑ in UE-FMA in RAE (p=0.008) & CT (p=0.016), These improvements were not significant at 1 month;<br>UE-FMA was sig > in RAE than CT at the 1-month follow-up (P = 0.02)	Home-based training with the RAE was feasible and significantly reduced impairment without increasing pain or spasticity in Stroke patients with severe arm impairment.	2
**324**	Wang et al., 2015	Taiwan	**Sample size:** I= 25 C= 26<br><br>**Age Range:** NR<br><br>**Mean Age:** I=62.0 ± 9.5.0 C= 65.4 ± 10.6<br><br>**PSD(days):** I = 8 C = 8.5<br><br>**TOS(ischemic/hemorrhagic):**NR	Received weekly personalized caregiver-mediated, home-based intervention (CHI) trainings	Received visits from the therapist without intervention (VT)	Balane (Berg balance Scale); walking speed (10-Meter Walk Test); walking capacity (6-Minute Walk Test (6MWT)); participation (Stroke Impact Scale (SIS); ADL (Barthel Index); subjective caregiver burden (Caregiver Burden Scale)	CHI: Sig ↑ in SIS domains of strength (*P* < .001), mobility (*P* < .001), ADL/IADL (*P* = .002), composite physical (*P* < .001), communication (*P* = .018), social participation (*P* = .004), and general recovery (*P* < .001) while VT showed no improvement. Sig btwn-grp diff favouring CHI in the SIS domain of strength, mobility, composite physical, and general recovery domain of the SIS (p<0.05). Sig within-group (time) effects on free-walking velocity (p=0.005), maximal walking velocity (p=0 .01), BBS (p=0 .005), BI (p=0 .001), & 6MWT (p=0.030)	CHI can improve physical functional recovery and, possibly, social participation in patients with chronic stroke.	3

							were observed in CHI but not VT. CHI did not sig ↑ caregiver burden at endpoint.		
**325**	Chaiyawat & Kulkantrakorn, 2012a	Thailand	**Sample size:** I=30, C=30 **Age** (Yrs): I= 67±10 C=66±11 **PSD** (Days): I=10±1 1.7 C=10.9±2.3 **TOS:** Ischemic	Home-based Physical therapy, education support, counseling and audiovisual materials (HBR)	Rehabilitation as prescribed by a physician and educational materials. (PPR)	Balance (Barthel Index (BI)), depression (Hospital anxiety and depression scale (HADS)), dementia (Thai-mini mental state examination [TMSE])	BI was significantly more improved in the intervention group (97.2± 2.8) than in the control group (76.4± 9.4) (95% confidence interval: 42.0–85.0). TMSE and HADS score did not significantly differ between the two groups (p=0.24; p= 0.87) respectively.	HBR in the first 6 months after stroke can lead to a faster recovery than PPR and reducing disability and depression, but not dementia.	3
**326**	Dean, et al., 2012	Australia	**Sample size:** I=76, C=75 **Age Range** Yrs: I=31-91 C=40-85 **Mean Age** (Yrs): I= 66.7± 14.3 C=67.5 ± 10.2 **PSD** (years): I=6.7 ±6.7 C=5.2 ± 5.4 **TOS:**Ischemic/hemorrhagic: NR	Exercise classes, advice, and a home program with aim to improve walking, prevent falls and increase physical activity for 12 months (WFT)	Exercise classes, advice, and a home program for 12 months with aim to improve upper-limb and cognitive functions (ULC)	PO: Walking capacity (6MWT). Walking speed (10MWTt). Falls were monitored for 1 year with monthly fall calendars. SO: falls risk score (Short-form Physiological Profile Assessment), habitual physical activity (Digimax pedometer), QoL (SF-12) Version 2), community participation(the Adelaide Activities Profile), and health system contact(falls calendars)	After 12M, WFT walked 34 m further in 6mins ($p<0.001$) & walked 0.07m/s faster over 10m during the fast walk ($p=0.03$) than ULC after controlling baseline performance. No sig diff btwn the groups for walking at a comfortable speed. No sig diff in the proportion of fallers (RR = 1.22; 95% CI = 0.91-1.62; *P* =.19) or the rate of falls between groups (IRR = 0.96; 95% CI = 0.59-1.51; *P* = .88). No sig diff btwn the groups in falls risk score, physical activity, or QoL.	The experimental intervention delivered through stroke clubs enhanced aspects of mobility but had no effect on falls, physical activity and quality of life.	3
**327**	Chaiyawat &Kulkantrakorn, 2012b	Thailand	**Sample size:** I=30 C=30 **Age Range:** NR **Mean Age**: I=67 ± 10 C= 66 ± 11 **PSD** (Days): I= 10 ± 1.7	Home-based physical therapy once a month for 6 months, along with educational support, counselling and audiovisual	Rehabilitation as prescribed by a physician and educational materials upon discharge from	ADL (BI); depression (Hospital Anxiety and Depression Scale [HADS]); dementia (Thai Mini-Mental State Examination [TMSE])	Over 2 years, the mean Barthel Index and Hospital Anxiety and Depression Scale were significantly improved in the intervention group compared to the control group (Barthel Index: $P < 0.001$; Hospital Anxiety and Depression Scale: $P = 0.003$). However, the Thai Mini-Mental State Examination in both groups did not significantly differ ($P = 0.068$).	At 2 years follow-up, it was evident that a 6-month home rehabilitation programme after ischemic stroke improved functional outcome and reduced incidence of depression, but not	3

			C=10.9 ± 2.3<br><br>**TOS:** NR	materials	hospital			dementia	
**195**	Michielsen et al., (2011)	Netherland	**Sample size: I=** 20 C= 20<br>**Age Range:** I= NR C= NR<br>**Mean Age:**<br>**I=** 55.3 ± 12.0 C= 58.7 ± 13.5<br>**PSD(years):** I= 4.7 ± 3.6 C= 4.5 ± 2.6<br>**TOS(ischemic/hemorrhagic):** I = 14/6 C = 14/6	Homebased mirrow therapy with the affected hand positioned behind the mirror while they looked at the reflection of the unaffected hand in the mirror.	Home based mirrow therapy with a direct view of both hands,	PO: Motor function (FMA). SO: :grip force with a Jamar handheld dynamometer, spasticity with the Tardieu scale, and pain with a visual analog scale ranging from 0 to 100 mm, motor capacity with the ARAT, self-perceived performance with the ABILHAND questionnaire, actual performance in daily life during 24 hours with the Stroke-ULAM and quality of life with the EQ-5D.	Posttreatment, the FMA improved more in the mirror than in the control group (*P* < .05), but this improvement did not persist at follow-up. No other ithin nor between group changes were found on the other outcome measures (all *P* >.05). fMRI results showed a shift in activation balance within the primary motor cortex toward the affected hemisphere in the mirror group only	This trial showed some effectiveness for mirror therapy in improving the motor function of chronic stroke patients and is the first to associate mirror therapy with cortical reorganization.	3
**328**	Mayo, et al, 2009	Canada	**Sample size:** I=96, C=94<br>**Mean Age:** I=70.0±14.5 C=71.6±13<br>**PSD(days):** I=12.7±11.8 C=24.1±15.5<br>**TOS:** NR	Case management: through home visits and telephone contacts for a period of 6 weeks	Usual Care	Rate of self-perceived health (Euroquol visual analog scale (EQ-VAS).	No difference in self perceived health was found between the two groups	Case management intervention showed no better improvement than the usual care.	2
**329**	Crotty, et al, 2008	Australia	**Sample size:** I=113, C=116<br>**Age** (Yrs): I=71.2±13.4 C=72.2±14.8<br>**PSD (days):** I=15.3±16.5 C=13.9±10.6<br>**TOS:** NR	hospital-based day rehabilitation programme (Hosp)	home-based rehabilitation programme. (Home)	Functional competence in ADL (AMPS); QoL (SF-36). Dynamic balance (TUG) ADL FIM). Self Perceived Quality of life (SF-36), Muscle strength (Dynamometer); Carer Stress (CSI).	Sig within-grp ↑ in AMPS (baseline-3M follow-up), but no btwn-grp diff. No sig btwn-grp diff in SF-36, TUG and quadriceps strength. While the FIM at 3M follow-up (p=0.01) and the change scores from baseline to 3 months (p=0.03) were higher for Hosp. Hosp Carers had higher CSI than Home Carers (p=0.047).	Hosp>Home in ADL. However, both programmes are ccomparable in providing improvement in functional abilities, QoL, balance and strength in post stroke patients	3
**330**	Page, et al, 2008	USA	**Sample size:** 7	a resistance-based,	A home excs prog	motor impairments (FMA); balance (Berg Balance Scale	After HEP participation, subjects showed no changes on any of the	Impairment reductions and	3

			**Age Range:** NR<br><br>**Mean Age:** NR<br><br>**PSD:** NR<br><br>**TOS =** NR	reciprocal, affected leg locomotor training protocol using the NuStep apparatus, then HEP<br><br>NB: Randomized crossover design	consisting of self-supervised practice with fractionated joint movements of the lower limb (HEP), then NuStep (HEP–NuStep)	[BBS])	outcome measures. After NuStep participation, patients in both treatment groups showed impairment reductions and increased balance. These trends were exhibited regardless of group assignment.	balance gains may be achieved using aresistance-based, reciprocal upper and lower limb locomotor training protocol.	
**331**	Desrosiers et al., 2007	Canada	**Sample size:** I=29; C=27<br>**Age (Yrs):** I=70.0±10.2 C= 70.0±12.0<br>**PSD(months):** I=24.5±25.7 C= 32.7±37.8<br>**TOS(ischemic/hemorrhagic):** 1=26/3; C=23/4	Leisure Education Program at home once a week for 8 to 12 weeks (LEP)	Visited at home at a similar frequency (HV)	perceived well-being and symptoms of distress (General Well-Being Schedule); Depressive symptoms (Center for Epidemiological Studies Depression Scale (CES-D)); health related quality of life [HRQOL] (Stroke-Adapted Sickness Impact Profile (SA-SIP30))	LEP had more active than passive leisure activities. Sig btwn-grp diff for active leisure activity duration as well as for the number of activities (p= 0.01). No sig btwn-grp diff in the duration of passive leisure activities (p>0.05). Satisfaction with leisure ↑ed only in LEP and the btwn-grp diffs were sig (p=0.003), except for satisfaction with the use of spare time. Well-being in LEP ↑ed but the bywn-grp diff was not sig. Both groups ↑ed their HRQOL but no sig diff btwn them. Only LEP sig ↓ed their depressive symptoms; with sig btwn-grp diff (*P*=0.01).	Leisure education program is effective for improving participation in leisure activities, improving satisfaction with leisure and reducing depression in people with stroke	3
**332**	Studenski et al., 2005	USA	**Sample size:** I= 44, C= 49<br>**Age** (Yrs)**:** I= 68.5±9.0 C= 70.4±11.3<br>**PSD(months):** I= 25.2±18.4 C= 28.7±16.7<br>**TOS(ischemic/h**	home-based exercise program with target on strength, balance, endurance and encouraged use of the	Usual care as prescribed by their personal physician (UC-P)	Stroke severity (OPS); ADL (FIM, BI, , Lawton and Brody IADL); Community Ambulation (gait speed thresholds (0.8 m/s)); QOL(subscales of SIS and SF-36).	The intervention and usual care groups demonstrated recovery of function. Each group increased the proportion of participants who were independent in basic ADLs (Barthel score) and who were independent in community ambulation. Both groups demonstrated increasing scores in	home-based exercise program provided better significant improvement in function and quality of life than the usual care in post stroke	3

			**emorrhagic):**<br>1=39/5, C=44/5	affected upper extremity (HBEx)			motor FIM, SF-36, and SIS scales. For SIS, gains in the intervention group were greater than in usual care in every domain and were significantly greater for SF-36 social function, SIS strength, emotion, social participation, and physical function and SIS upper extremity function. There were no detectable effects on the motor or cognition scales of the FIM or on instrumental ADL (IADL) score	individuals	
**333**	McClellan &Ada, 2004	Australia	**Sample size:**<br>I= 13, C= 10<br>**Age** (Yrs)**:**<br>I= 69 ±13<br>C= 72 ± 9<br>**PSD(months):**<br>I= 6.5 ±5.5<br>C= 4.5 ± 3.0<br>**TOS (ischemic/ hemorrhagic):**<br>NR	participated in a six-week, home-based mobility program, aimed at improving mobility in standing and walking (HBMP)	participated in a six-week, home-based program of upper-limb exercises (HBUE)	Standing (Functional Reach Test (FR)); walking (Item 5 of the MAS). Quality of life (stroke-adapted 30-item version of the Sickness Impact Profile (SA-SIP30))	FR ↑ed sig: HBMP>HBUE btwn wks 0 & 6 (*p*=0.01) and btwn wks 0 & 14 (*p*=0.04). Btwn wks 0 & 6, Item 5 of MAS ↑ed slightly in both groups, with no sigbtwn-grp diff (*p*=0.50). Btwn wks 0 & 14, Item 5 of MAS & SA-SIP30 remained relatively unchanged in both groups, with no sig btwn-grp diff (*p*>0.60). Betwn wks 0 & 6, SA-SIP30 was unchanged in both groups, with no sig btwn-grp diff (*p*=0.70)	A resource-efficient, mobility program was effective in improving standing ability after discharge from physiotherapy services in people affected by stroke	3
**334**	Askim, et al, 2004	Norway.	**Sample size:**<br>I= 31, C= 31<br>**Age** (Yrs)**:**<br>I=76.9, C=76.3<br>**TOS (ischemic/ hemorrhagic):**<br>NR	newly constructed extended service (ES)	ordinary stroke unit service: (OS)	Disability (mRS & BI); subjective health status (NHP); burden of the patient's illness on the caregiver (CSI))	No sig btwn-grp diff for any of the 6dimensions of the NHP at 6 or 52 week follow-up, but a sig diff in favour of ES in the dimension social isolation (*p*=0.046) at 26 weeks. No sig btwn-grp diff or within-grp diff in the rest of the measured outcomes.	An extended stroke unit service with early supported discharge seems to have no positive effect on functional outcome for patients living in rural communities, but might give a trend toward better quality of life.	3
**335**	Duncan et al., 2003	USA	**Sample size:**<br>I=44, C= 48<br>**Age** (Yrs)**:**<br>I=68.5 ±9.0	structured, progressive, physiologically based,	usual care (UC).	Stroke severity (the Orpington Prognostic Scale); stroke motor recovery (Fugl-Meyer	The overall effect of SHP compared with UC on the combined outcomes was highly significant (*P*=0.0056) by a single MANOVA. Both groups	3-month structured, physiologically based, progressive, supervised home-	3

			C= 70.2 +11.4 **PSD(days):** I= 77.5 +28.7 C= 73.5 ±27.1 **TOS (ischemic/ hemorrhagic):** I=39/5, C=44/4	therapist-supervised, in-home program (SHP)		Motor Score); gait velocity (10-Meter Walk); walking distsance (The Six-Minute Walk); balance (The Berg Balance Scale)	↑ed from baseline to 3M in strength, balance, UE & LE motor control, UE function, and gait velocity. UC did not show gains in endurance. SHP > UC in measures of endurance, balance, 6MWD, and gait velocity. Both groups showed no improvement in WMFT.	based exercise program improved stroke recovery compared with usual care	
**336**	Andersen, et al, 2002	Denmark	**Sample size:** G1=54, G2=53 C=48 **Age** (Yrs)**:** G1 = 69.8 ± 9.9 G2 **=** 74.1 ± 11.4 C = 68.3 ± 12.3 **PSD (days):** G1**:**87.6,G2:83.0 C=98.3 **TOS (ischemic/ hemorrhagic):** NR	G1 = follow-up home visits by a physician (HVP) G2 = PT instruction in the patient's home, or (PI)	standard aftercare	Neurological impairments at discharge (SSS, BMRC-MSV); mobility (FQoM)); cognitive functions (a detailed test battery); Functional limitations (BI);	No statistically significant differences were demonstrated in all the outcomes; However all used measurements showed a tendency towards higher scores, indicating better function in both intervention groups compared with the control group	Physician and physiotherapist follow-up services after stroke may be a way of improving functional outcome	3
**337**	Roderick et al., 2001	UK	**Sample size:** I=66, C=74 **Age Range:** I**=** 78.3 (62 – 91) C= 79.6 (60-95) **PSD:** NR **TOS(I/H):** NR	Domiciliary rehabilitation service for elderly patients with stroke	Geriatric day-hospital care	Functional status (BI), mobility (RMI); , Mental state (PGCM); social activity (FAI); and geriatric QoL (SF-36); Health service and local authority social service cost (Economic evaluations)	No significant difference in outcomes were detected and both groups showed no significant improvement in any of the measured outcomes	None of the interventions provided improvement in patients mental health, social activity, quality of life, and total costs	2
**338**	Widen Holmqvist et al., 1998	Swedes & Spain	**Sample size:** I=41, C=40 **Age** (Yrs)**:** I= 70.86+7.6 C= 72.66+8.9 **PSD:**NR **TOS (ischemic/ hemorrhagic):** NR	early supported discharge with continuity of rehabilitation at home for 3 to 4 months (HRG)	Routine rehab service in a hospital, day care, and/or out-patient care (RRH)	ADL (BI); Walking capacity (10 MWT); manual dexterity (peg test); Neurological function (rated by Senior Neurologist); Coping capacities of the patients and their spouses (SCS)	Overall there were no statistical significant differences between the group in all outcomes	HRG during the first 3-month period after acute stroke is not less beneficial than RRH	3
**339**	Gladman et al,	UK	**Sample size:** I=162, C=165	Treated by domiciliary	Treated by routine	Functional recovery (Extended ADL scale); and	Overall there were no differences between the groups in all the	Overall, there was no difference in the	3

	1993		**Age** (Yrs)**:** I=70, C=70 **PSD(days):** I=21, C=18 **TOS(ischemic/hemorrhagic):**NR	rehabilitation team after discharge	hospital-based Services after discharge	perceived health status (NHP); Informal carers (the Brief Assessment of Social Engagement and the Nottingham version of the Life Satisfaction Index).	measured outcomes	effectiveness of the domiciliary and hospital-based services	

QoM: Quality of Movement
AoU: Amount of Use
MAL: Motor Activity Log
NHPT: Motor Activity Log
ARAT: Action Research Arm Test
GDS: Geriatric Depression Scale
FMA: Fugl Meyer Assessment Scale
NIHSS: National Institutes of Health stroke Scale
mBI: modified Barthel Index
BBS: Berg's Balance Scale
mRS: modified Rankin Scale
CSI: Caregiver Strain Index
AMPS: Assessment of Motor and Process Skills
FIM: Functional Independence Measure
TUG: Time-up and Go test
ADL: Activities of Daily Living
QoL: Quality of Life
OPS: Orpington Prognostic Score
SIS: Stroke Impact Scale
SSS: Scandinavian Stroke Scale.
BMRC-MSV: British Medical Research Council Muscle Strength Assessment and visual field
FQoM: Functional Quality of Movement Scale
RMI: Rivermead Mobility Index
PGCM: Philadelphia Geriatric Center Morale Scale
FAI: Frenchay activity index
NHP: Nottingham Health Profile
SCS: Sense of Coherence Scale

Self Management

Ref	Study (Authors, year	Country	Participants	Interventions/ Model of care	Control/ comparison	Outcomes assessed/ outcome measures	Results	Conclusion	Quality score
**340**	Lo et al, (2018)	Australia	128adult stroke survivors<br>Age (Yrs): 67.46 ±11.95<br>PSD (days): 45± 26.16<br>TOS:ischaemic /haemorrhagic: N/S	stroke self-management programs (SESSMP)	usual stroke care	Self-effiacy: (SSEQ)<br>Expectation of performing self management behaviors (SSMOE)<br>Satisfaction with performance of self-management behaviors (SSMBP)	The intervention group improved signifiantly in self-effiacy (95% confience interval, 2.55–12.45; P<0.01), outcome expectation (95% confience interval, 5.47–14.01; P<0.01), and satisfaction with performance of self-management behaviors (95% confience interval, 3.38–13.87; P<0.01) compared with the control	The stroke self-management program improved survivors' self-effiacy, outcome expectation, and satisfaction with performance of self-management behaviors	2
**341**	Sit et al.(2016)	USA	210 stroke patients<br>Age (years): C=70.7±13.9<br>E= 67.8±14.2<br>PSD: N/A<br>TOS (haemorrhagic/ ischaemic)<br>C=27/98,E=27/76	Health Empowerment Intervention for Stroke Self-management [HEISS] plus Usual ambulatory rehabilitation	Usual ambulatory rehabilitation	Modified Barthel index (BI) was used to assess basic ADL performance on a personal level.<br><br>Chinese Lawton instrumental ADL (IADL) was used to assess Self-efficacy and self-management behaviour	Those in Intervention Group (IG) reported better self-efficacy in illness management 3-month (P□0.011) and 6-month (P□0.012) post intervention, along with better self-management behaviors at all follow-up time points (all P,0.05), apart from medication adherence (P.0.05).<br>Those in IG had signifiantly better functional recovery (Barthel, all P,0.05; Lawton, all P,0.001), compared to Control Group.	Patient empowerment intervention (HEISS) may influence self-efficacy in illness management and improve self-management behavior and functional recovery of stroke survivors.	4

SSEQ: Stroke Self Effiacy Questionnaire
SSMOE: Stroke Self-Management Outcome Expectation Scale
SSMBP: Stroke Self-Management Behaviors Performance Scale

Family or Caregiver-led Training

Ref	Study (Authors, year	Country	Participants	Interventions/ Model of care	Control/ comparison	Outcomes assessed/ outcome measures	Results	Conclusion	Quality score
**342**	Forster et al., 2013	UK	**Sample size:** I= 450, C= 478 **Mean Age:** I= 71.0 ±12.76 C= 71.3 ±12.18 **PSD:** NR **TOS (ischemic/ hemorrhagic):** I = 380/62/8 C = 401/77/0	competency-based training programme for caregivers [the London Stroke Carer Training Course (LSCTC)]	Usual care (UC) *(wrt: with respect to)*	PO: functional independence (NE-ADL) and caregiver burden (CBS) SO: FA & HRQoL (SIS); Mood (HADS); Health state (EQ-5D); ADL (BI); Death, Hospital readmission & Institutionalization	No differences in primary outcomes were found between the groups at 6 months (p=0.866). Furthermore, no differences were detected in any of the secondary outcomes. Health and social care costs, societal costs and outcomes are similar for the intervention and control groups at 6 months, 12 months and over 1 year.	No diff btwn LSCTC & UC wrt ↑recovery, ↓caregivers' burden, or ↑ing other physical & psycholog-ical outcomes, nor was it cost-effective wrt UC	3
343	Harris et al, (2010)	Canada/ Colombia	**Sample size:** **I=** 29, C= 21 **Mean Age:** **I=** 71.8±9.6 C= 65.5±14.5 **PSD(days):** I= 20.5±2.0 C= 20.2±2.3 **TOS:** NR	Intervention with caregiver support	Intervention without caregiver support	Motor function using Fugl-Meyer Upper-Limb Motor Impairment Scale	Group comparisons revealed that participants with caregiver support had improved upper-limb function compared with those without caregiver support	Involvement of caregivers was a determinant of improved upperlimb function	1
**344**	Torres-Arreola, et al., 2009	Mexico	**Sample size:** I = 124, C= 119 **Age** (Years): I = 71.0±10.5 C= 70.0 ± 10.2 **PSD**: NR **TOS**(ischemic/hemorrhagic): NR	physiotherapy plus caregiver education in rehabilitation (strategy 1, S1)	Education alone (strategy 2, S2).	Primary: Basic ADL (Barthel index[BI]) and social ADL (Frenchay activities index [FAI])	There was a significant improvement in BI in both groups ($P < 0.05$) and improvement in FAI in both groups at 3 months follow up. However, there was no statistically significant difference between the groups for both outcomes.	Both interventions can be used to improve the ADL of stroke patients. However, they do not show any sig diff in effectiveness	3
**345**	Tilling et	UK	**Sample size:** I= 170 C=170	family support	Usual care (UC)	PO: Patient satisfaction with services (PSS),	Fewer FSO were satisfied with community services (p = 0.02) but More FSO were	Family support appears to	3

	al, 2005		**Age Range:** I= NR C= NR<br><br>**Mean Age:**<br>**I=** 78 ±11.6<br>C= 77 ±10.6<br><br>**PSD(months):**<br>I= 25.2±18.4<br>C= 28.7±16.7<br><br>**TOS(ischemic/hemorrhagic):**NR	organiser (FSO) service		Depression (HADS); Stroke Impact (modified RNLI); Home Adaptations & Stroke care Satisfaction (PoSS); ADL (BI)<br>For carers: CSI & HADS; Satisfaction with stroke care (carer version of PoSS)	satisfied with information about recovery (p = 0.001) & advice about prevention (p = 0.09). More FSO were satisfied with the item of care for 7/10 items in PSS, but the overall diffs in satisfaction not sig diff (p = 0.8]. RNLI lower in FSO (p = 0.05), More carers in FSO were satisfied with the item of care for 6/10 items in their PoSS, but, overall no sig btwn-grp diff (p = 0.54). FSO>UC thought that the stroke still had a negative effect on their life (p < 0.001). At 3 months, more carers FSO (p = 0.08) felt that some good had come out of the stroke, fewer FSO were satisfied with equipment they had at home and more were satisfied with information about recovery (p = 0.07), advice about prevention (p = 0.007), knowing who to contact and feeling that someone had listened to them (p = 0.04).	benefit stroke patients and their carers in several aspects of life after stroke at both 3 months and 1 year	
**346**	Dey et al., 2005	UK	**Sample size:** I= 10 C= 10<br><br>**Age Range:** NR<br><br>**Mean Age:** NR<br><br>**PSD:** NR<br><br>**TOS(ischemic/hemorrhagic):**NR	Visited by members of a mobile stroke team who advised clinical staff on appropriate and timely investigation and management;	were not visited by the mobile stroke team.	Primary: all-cause mortality<br><br>Secondary: death or dependency, defined as a score of 18 or less on the BI, and death or institutionalized care, defined as hospital, hospice, nursing or residential home	There was no statistically significant difference observed between study groups in mortality at 6 weeks (*P* =0.87), nor at 12 months (*P* =0.25). Compared with the control group, there was a non-significant increase in the risk of death in the intervention group. There were no significant differences between the two groups in the percentage of patients at 12 months who died or were in institutional care (*P*=0.45) or were dead or dependent (*P*=0.27). Nor were any statistically significant differences observed between study groups in other functional outcomes or quality of life measures at 12 months	The trial was terminated before the necessary sample size was collected but findings suggest that the mobile stroke team failed to confer sigt long-term mortality benefit compared with general ward-based care alone	3
**347**	Mant et al, 2000	UK	**Sample size:** I= 156<br>C= 167<br><br>**Age Range:** NR	family support	Normal care	knowledge about stroke and use of services (Self developed questionnaire) Social activities of patients and carers (FAI); Disability and handicap	Carers in the intervention group had significantly better Frenchay activities indices (p=0·03), SF-36 scores (energy p=0·02, mental health p=0·004, pain p=0·03, physical function p=0·025, and general health perception p=0·02), quality of life on	Family support significantly increased social activities and improved quality of life for carers,	3

			**Mean Age:**<br>I= 74·7 + 10·0<br>C= 73·1 + 11·4<br><br>**PSD(months):**NR<br><br>**TOS(ischemic/hemorrhagic):**NR			(BI,RMI & LHS); emotional health of patient (HADS); emotional health of carers (GHQ-28 & CSI); QoL patient/Carer: (DCC & SF-36 carers only); and satisfaction with services and stroke understanding (Self-developed scales)	the Dartmouth co-op chart (p=0·01), and satisfaction with understanding of stroke (p=0·04) than those in the control group. Patients' knowledge about stroke, disability, handicap, quality of life, and satisfaction with services and understanding of stroke did not differ between groups. Fewer patients in the intervention group than in the control group saw a physiotherapist after discharge (p=0·04), but use of other services was similar.	with no significant effects on patients.	

NE-ADL: Nottingham Extended Activities of Daily Living
CBS: Caregiver Burden Scale
SIS: Stroke Impact Scale
FA: Functional Ability
HRQoL: Health Related Quality of Life
HADS: Hospital Anxiety and Depression Scale
EQ-5D: European Quality of Life-5 Dimensions
BI: Barthel Index
PSS: Patient Satisfaction Scale
RNLI: Reintegration to Normal Living Index
PoSS: Pound Satisfaction Scale
CSI: Caregiver Strain Index
FAI: Frenchay Activities Index
RMI: Rivermead Mobility Index
LHS: London Handicap Scale
GHQ: General Health Questionnaire
DCC: Dartmouth Coop Charts

Telerehabilitation

Ref	Study (Authors, year	Country	Participants	Interventions/ Model of care	Control/ comparison	Outcomes assessed/ outcome measures	Results	Conclusion	Quality score
**321**	Chen et al.(2017)	China.	97 partcipants<br>Age (yrs):<br>E=66.52±12.08<br>C=66.15±12.33<br>PSD (day):<br>E=16.41±3.74<br>C=16.93 ± 3.25<br>TOS: (I/H)<br>E=24/3, C=22/5	Home-based telesupervising rehabilitation group (HBT)	Conventional rehabilitation (CR)	ADL (mBI), Balance (BBS)<br>Functional Outcome (mRS)<br>Carer Stress (CSI)	For the MBI score that was mainly used to evaluate the basic activities of daily life, compared with the baseline values,<br>both groups showed signif icant improvements after treatment (55.56–67.31 in the HTR groups, 54.26–66.04 in the COR group; F = 138.965; P < 0.001)	HBT is most likely as effective as CR for improving functional recovery in stroke survivors and could ease the burden of caregivers as CR	4
**348**	Wan et al, (2016)	China	99 patients<br>Age, (years):<br>C= 60.24±12.57<br>E=59.07 ± 12.36<br>PSD: within one month<br>TOS= Ischaemic	Predischarge education and 3 goal-setting follow-up sessions conducted by phone	Usual stroke education	modified Rankin Scale (mRS) used for functional outcome	Medication adherence 1.461 (.230) 14.274 (<.001) 5.243 (.025*)<br>①Baseline 3.31 (.65) 3.19 (.80) .729 (.468)<br>②3-month follow-up 3.55 (.75) 3.69 (.74)− .864 (.390)<br>③6-month follow-up 3.48 (.80) 3.86 (.57)− 2.46 (.016)	Goal-setting telephone follow-up intervention for ischemic stroke patients is feasible and leads to improved medication adherence	4
**349**	Chumbler et.al, 2015	USA	P: Persons with experience of either previous ischemic or heamorrhagic stroke<br>Sample size = 52 (E=23, C=29),<br>PSD: 24 months	Stroke Telerehabilitation - STeleR: (home visit, five telephone calls, and an in-home	Usual Veteran Affairs (VA) or non-VA care, such as home health care.	Fall related self efficacy(falls efficacy scale), patients satisfaction(SSPS)	-STeleR group compared with Usual Care, showed statistically significant improvements in one of the two SSPSC scales<br>(satisfaction with hospital care, p=0.029) and approached significance in the second SSPSC scale (satisfaction with home care, p =0.077).	-The STeleR intervention improved satisfaction with care, especially as it relates to care following their experience from the hospital.	3

			Mean age NA TOS(ischemic/hemorrhagic): NA	messaging device)			-There were no improvements in fall-related self-efficacy		
**350**	Carey et al., 2007	USA	**Sample size:** I= 10 C= 10<br><br>**Age Range:** I= NR C= NR<br><br>**Mean Age:** I=65.9 ± 7.4 C= 67.4 ± 11.8 **PSD(months):** I=42.5 ± 24.3 C= 35.6 ± 26.1<br><br>**TOS(ischemic/hemorrhagic):**NR	computerized finger and wrist tracking training via telerehabilitation involving temperospatial processing to achieve accuracy (TRwA)	movement training telerehabilitation with no attention to accuracy (TRwo)	Dexterity (Box and Block test); gross movement (Jebsen Taylor test); range of motion (F-ROM); Accuracy of tracking movements of the index finger (F-MTT). Brain activation (fMRI)	BBT:,Sig ↑ in TRwA (*P* = 0.03) while TRwoA had >↑ (*P* < 0.001). JTT:, Sig ↑ in TRwA (*P* = 0.01) & TRwoA (*P* = 0.03). F-ROM: , sig effects for test (*P* = 0.02) & interact-tion (*P* = 0.03); TRwA sig ↑ed (*P*=0.004), but Δ in TRwoA was not sigF-MTT: No sig interaction effect (*P* = 0.07); but, TRwA ↑ed sig (*P* = 0.02), unlike TRwoA. For the relative volume of activation, laterality index data, and intensity index in the ipsilesional, and controlateral hemisphere, a sig time & interactin effect was found favoring TRwA	Telerehabilitation may be effective in improving performance in subjects with chronic stroke. Tracking training with reinforcement to enhance learning, however, did not produce a clear advantage over the same amount of practice of random movements	2

mBI: modified Barthel Index
BBS: Berg Balance Scale
mRS: modified Rankin Scale
CSI: Caregiver Strain Index
JTT: Jebsen Tylor test
F-ROM: Finger range of motion test
F-MTT: Finger movement tracking test
SSPS =

Early Therapy/Rehab

Ref	Study (Authors, year	Country	Participants	Interventions/ Model of care	Control/ comparison	Outcomes assessed/ outcome measures	Results	Conclusion	Quality score
**351**	Pan (2018)	China	86 patients with acute CVA<br>Age range:<br>E= 62.15± 7.9<br>C= 61.89± 8.4<br>PSD= N/A<br>TOS(ishcaemic / haemorrhagic/ mixed)<br>E= 16/ 23/ 6<br>C= 14/ 22 /5	Rehabilitation based on the basic therapy(RBT)	Routine primary therapy (RPT)	-Simplified Fugl–Meyer assessment (FMA),<br>-Neurologic deficit scale (NDS),<br>-Barthel index (BI)<br>-All used to evaluate the recovery of hemiplegic limb movement ability and the improvement of daily living ability before and after treatment	FMA, NDS, and BI of patients in both groups were distinctly ameliorated after treatment (P< .05). (RBT sig > RPT) (P< .05). Total FMA in RBT sig ↑er than RPT (P< .05).	Early RBT can sig ameliorate the movement ability of hemiplegic LE in patients with acute CVA. Its therapeutic effect is remarkable.	1
**352**	Faulkner et al,.(2017)	North Carolina	47 participants<br>Age range(years):<br>E=66 ± 12<br>C= 68 ± 10<br>PSD: [N/S]<br>TOS= Ischaemic	Early exercise engagement (EEE)	Usual care (UC)	Peripheral and central hemodynamic variables: cPP, cSBP, DP, MAP, pDP, pDBP, PI, pPP, pSBP.<br>AIx	No sig btwn-grp diff in all baseline variables (P>0.05). Sig interaction for cSBP, pSBP and AIx (P <0.05), with EEE presenting lower values than UC post intervention.	EEE post stroke may elicit sigt beneficial ∆s to cSBP AIx&.	4
**353**	Liu et al, 2014	China	Sample size= 243 (E=122, C=121);<br>Mean age (yrs):<br>E= 58.5±12.3,<br>C= 59.1±15.5;<br>PSD: E<48hrs,<br>C = 1 week;<br>TOSHaemorrhagic	Very Early (<48hrs) Rehabilitation (VER)	Standard Rehabilitation (SR)	- Survival,<br>- HRQoL (SF-36),<br>- Function (mBI), and<br>- Anxiety (ZungSRAS)	@ 6M SC were more likely to have died (AHR: 4.44; 95%CI: 1.24–15.87). 6-point diff in SF-36 Physical (CI=4.2–8.7), 7-point diff in SF-38 Mental (CI=4.5–9.5).13-point diff in mBI (CI=6.8–18.3), & 6-point diff in SRAS (CI= 4.4–8.3) was reported in favor VER.	VER improves survival and functional outcomes at 6 months after stroke in hospitalized patients	3
**35**	Lin	Chi	**Sample size:**	Training on	No training	Memory (Wechsler	TES sig ↑scores in 5/7 WMS	computer-	3

**4**	et al., 2014	na	I= 16, C= 18<br>**Mean Age:**<br>I=62.4 ±6.0<br>C= 63.2 ±5.7<br>**PSD(days):**<br>I=2227.5 ±24.0<br>C= 228.1 ±18.4<br>**TOS:** NR	early sitting (TES)	(NT)	Memory Scale [WMS] memory quotient [MQ] and Trail Making Test [TMT])). Brain activity (fMRI)	subtests, MQ and TMT-A (P<0.05). TES ↑ FC of the hippocampus with the frontal lobe & left parietal lobe NT ↓ed FC of the left hippocampus–right occipital gyrus, and right hippocampus–right posterior lobe of cerebellum and left superior temporal gyrus..	assisted cognitive training is an effective therapy for the improvement of memory in post stroke patients	
**355**	Tang et al., 2014	China	**Sample size:**<br>I= 24, C= 24<br>**Mean Age:**<br>I=68.2 ±4.1<br>C= 66.9 ±4.1<br>**PSD(days):**<br>I=16.6 ±5.05<br>C= 16.8 ±4.9<br>**TOS:**NR	early sitting, standing, and walking in conjunction with the CBA (ECBA)	Contemporary Bobath approach (CBA)	Balance (BBS); mobility (STREAM);	ECBA sig ↑ed STREAM than CBA in the LE (*P* < .001) and basic mobility (*P*< .001) domains and also on the overall STREAM Scores (*P* < .01) However, there was no difference in UE mobility between the 2 groups. BBS of the ECBA was sig ↑er than CBA (*P* < .001).	ECBA is a valuable intervention to improve LE mobility, basic mobility, and balance ability post stroke	3
**356**	Cumming et al., 2011	Australia	**Sample size:**<br>**I=** 38, C= 33<br>**Mean Age:**<br>I= 74.6 ±14.6<br>C= 74.9 ±9.8<br>**PSD:** NR<br>**TOS:** NR	very early and intensive mobilization: within 24 hours of stroke (VEIM)	Standard stroke unit care. (SSC)	PO: the number of days required to return to walking 50 meters unassisted.<br>SO: ADL (BI) and motor activity (RMA)	VEIM returned to walking sig faster than SSC (*P*=0.032). no sig btwn-grp diff were noted in any of the secondary outcomes.	VEIM after stroke may fast-track return to unassisted walking & functional recovery.	2
**357**	Sorbello, et al., 2009	Australia	**Sample size:**<br>I = 38, C= 33<br>**Mean Age** (Years):<br>I = 74.6 ±14.6<br>C= 74.9 ±9.8)<br>**PSD**: NR<br>**TOS** NR	Very early and frequent mobilization (VEM) with Standard care	Standard care only	Number of complications	There was no difference in the total number of complications by group (p = 0.61)	VEM showed no better improvement in complications.	2
**176**	Sawaki et al., 2008	USA	**S. size:** I=17, C=13<br>**Age** Mean(Range) yrs<br>I=54.4±3.8 (30-79)<br>C=58.5±3.5 (36-78)<br>**PSD(days):**I=28.9±12<br>C=26.1±10.9<br>**TOS:** NR	Early CIMT immediately after baseline Evaluation (E-CIMT)	Late CIMT receiving CIMT after 4 months (L-CIMT)	Upper extremity function (Wolf Motor Function Test (WMFT)); cortical reorganization (transcranial magnetic stimulation [TMS])	Both grps ↑ed WMFT 2wks after baseline. E-CIMT>L-CIMT sig ↑ing grip force (WMFT) (P = .049). E-CIMT had a borderline sig ↑TMS motor map area compared with L-CIMT over a 4M period (P = .053)	CIMT sig & clin relevant ↑ in arm motor function with effect lasting up to 4 months.	1
**358**	Draper et	Austra	**Sample size:**<br>I= 107, C= 83	immediate treatment	3 months delayed	Cargiver Psychological distress (GHQ); Caregiver	Caregivers IT had sigt ↓in GHQ s (P= 0.006) unlike DT. Improvement	Stroke caregiver support,	2

	al., 2007	lia	**Mean Age:** I= 62.83±10.25 C= 64.02±12.03 **PSD:** NR **TOS:** NR	(IT)	treatment (DT)	burden (RSS); caregiver's use (ComA) and perceived effectiveness (ComB) of eight functional communication strategies (A specifically designed communication questionnaire)	was not maintained at 3M follow-up. No sig effects of the programme on communication skills or on caregiver burden.	education & training programmes have short-term effects on caregiver stress levels	

cPP: central Pulse Pressure;
cSBP: central Systolic Blood Pressure;
DP: Double Product;
MAP: Mean Arterial Blood Pressure;
pDP: peripheral Double Product;
pDBP:,peripheral Diastolic Blood Pressure;
PI: Post-Intervention;
pPP, peripheral Pulse Pressure;
pSBP, peripheral Systolic Blood Pressure
AIx: Augmentation Index
AHR: Adjusted Hazard Ratio
SRAS: Self-Rated Anxiety Scale
FC: Functional Connectivity
RMA: Rivermead Motor Assessment
CIMT: Constraint Induced Movement Therapy
GHQ: General Health Questionnaire
RSS: Relatives' Stress Scale
